# Radical-Induced Cascade Annulation/Hydrocarbonylation for Construction of 2-Aryl-4*H*-chromen-4-ones

**DOI:** 10.3390/molecules27217412

**Published:** 2022-11-01

**Authors:** Xinwei He, Keke Xu, Yanan Liu, Demao Wang, Qiang Tang, Wenjie Hui, Haoyu Chen, Yongjia Shang

**Affiliations:** Key Laboratory of Functional Molecular Solids, Ministry of Education, Anhui Laboratory of Molecule-Based Materials (State Key Laboratory Cultivation Base), College of Chemistry and Materials Science, Anhui Normal University, Wuhu 241000, China

**Keywords:** cascade reaction, chromen-4-ones, hydrocarbonylation, propargylamines

## Abstract

A robust metal- and solvent-free cascade radical-induced C-N cleavage/intramolecular 6-*endo-dig* annulation/hydrocarbonylation for the synthesis of the valuable 2-aryl-4*H*-chromen-4-ones is described. This practical synthesis strategy utilizes propargylamines and air as the oxygen source and green carbonylation reagent, in which propargylamines are activated by the inexpensive and available dimethyl 2,2′-azobis(2-methylpropionate) (AIBME) and (PhSe)_2_ as the radical initiators. This simple and green protocol features wide substrate adaptability, good functional group tolerance, and amenability to scaling up and derivatizations.

## 1. Introduction

Chromone frameworks are frequently found in bioactive natural products, including natural flavone and isoflavone products [1,2,3,4]; biologically and therapeutically active drugs [5,6,7,8], such as anti-inflammatory, antiviral, antimicrobial, antioxidative, and anticancer agents; and drug candidates for neurodegenerative diseases and adenosine receptors [9,10,11,12]. The importance of their structures greatly promoted the development of diverse procedures for their formation. Typically, chromones can be prepared by the classical synthetic routes, including Claisen condensation [13,14], Baker–Venkataraman [15], Kostanecki–Robinson reaction [16,17], benzopyrylium salts [18,19,20], and Vilsmeier–Haack reaction [21,22], utilizing *ortho*-hydroxyarylalkylketones as starting materials. In addition, phenols, salicylic acid, and derivatives have also been used to synthesize chromones via the Simonis [23,24] and Ruhemann [25] reactions and others [26,27,28,29,30,31].

**Scheme 1:**
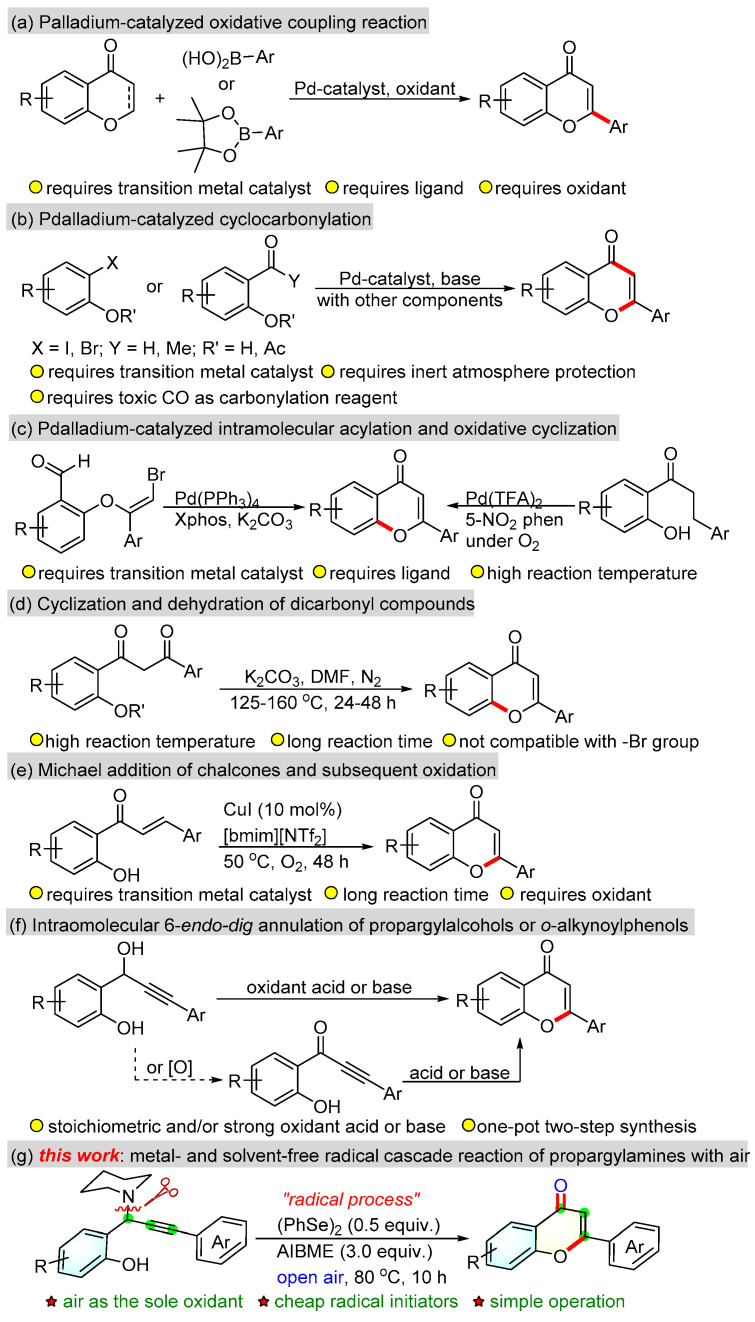
Modern strategies for accessing 2-aryl-4*H*-chromen-4-ones.

Given the promising potential of 2-aryl-4*H*-chromen-4-ones in drug discovery and pharmaceutical applications, consequently, much effort has been focused on the development of new synthetic methods. Several practical and convenient transition-metal-catalyzed coupling methods have been used for the formation of 2-aryl-4*H*-chromen-4-ones due to their importance [32,33,34]. During the last decades, transition-metal-catalyzed coupling reactions have provided one of the most attractive methodologies for C−C bond formation. The application of palladium-catalyzed protocols, including oxidative arylation of chromones with phenylboronic acids (Scheme 1a) [35,36,37], and carbonylative cyclization using CO gas as a carbonyl source (Scheme 1b) [38,39,40,41,42,43,44], for the construction of chromones has attracted significant attention for a long time. Additionally, the Pd-catalyzed intramolecular acylation and oxidative cyclization can be used to synthesize 2-aryl-4*H*-chromen-4-ones (Scheme 1c) [45,46]. Recently, other methods involving intramolecular annulation of 2-alkoxyphenylacetophenones (Scheme 1d) [47,48] and 2′-hydroxychalcones (Scheme 1e) [49,50,51,52] via C–O bond formations have also been reported. Moreover, the synthesis of 2-aryl-4*H*-chromen-4-ones can be accomplished through the regioselectivite 6-*endo-dig* cyclization of *ortho*-hydroxyphenyl propagylic alcohols and *ortho*-hydroxyphenyl alkynones (Scheme 1f) [53,54,55,56,57]. Thus, these methods are good supplements to classical methods, but they generally have drawbacks of requiring harsh reaction conditions such as strong acids or bases, stoichiometric oxidants, and a long reaction time. In addition, transition-metal-catalyzed annulations often require the use of highly toxic carbon monoxide or expensive palladium catalyst and ligand at high temperatures. Inspired by our pioneering results, in which multireactive propargylamines displayed unique feature of cyclization, and in continuation of our interest in developing new synthetic protocols to build valuable heterocyclic frameworks [58,59,60,61,62,63,64], we herein disclose a cascade annulation of propargylamines for the synthesis of 2-aryl-4*H*-chromen-4-ones under green and operationally simple metal- and solvent-free conditions, which is unprecedented in previous works (Scheme 1g). This cascade process presumably involves a sequence of radical-induced C-N cleavage, followed by C-O coupling, intramolecular 6-*endo-dig* annulation, thermal hemolytic cleavage, and oxidative hydrocarbonylation.

## 2. Results and Discussion

We commenced with an investigation of propargylamine **1aa** as a model substrate with air as the oxygen and carbonyl source to identify the reaction conditions (Table 1). The initial test of **1aa** in the presence of diphenyl diselenide and dimethyl 2,2′-azobis(2-methylpropionate) (AIBME) in 1,2-dichloroethane (DCE) under an air atmosphere gave the desired product **2aa** in a 63% isolated yield (entry 1). The influence of the solvents in this model reaction was then examined, and inferior results were obtained (entries 2–6). The following screening of the amount of diphenyl diselenide and AIBME (entries 7–12) showed that the 0.5 equiv. of diphenyl diselenide and 3.0 equiv. of AIBME was the best choice (entry 8). Changing the radical initiator from AIBME to AIBN (azodiisobutyronitrile) decreased the yield to 37% (entry 13). Interestingly, the yield of **2aa** increased to 85% and 78% when the reaction was performed under solvent-free and blue LED light conditions, respectively (entries 14, 15). Furthermore, the effect of the reaction temperature and time was investigated, and the results revealed that these attempts did not show any improvement in the obtainable yield (entries 16–19). After extensive experimentation, we selected the conditions used in entry 14 as the optimal ones for the further investigations.

**Scheme 2:**
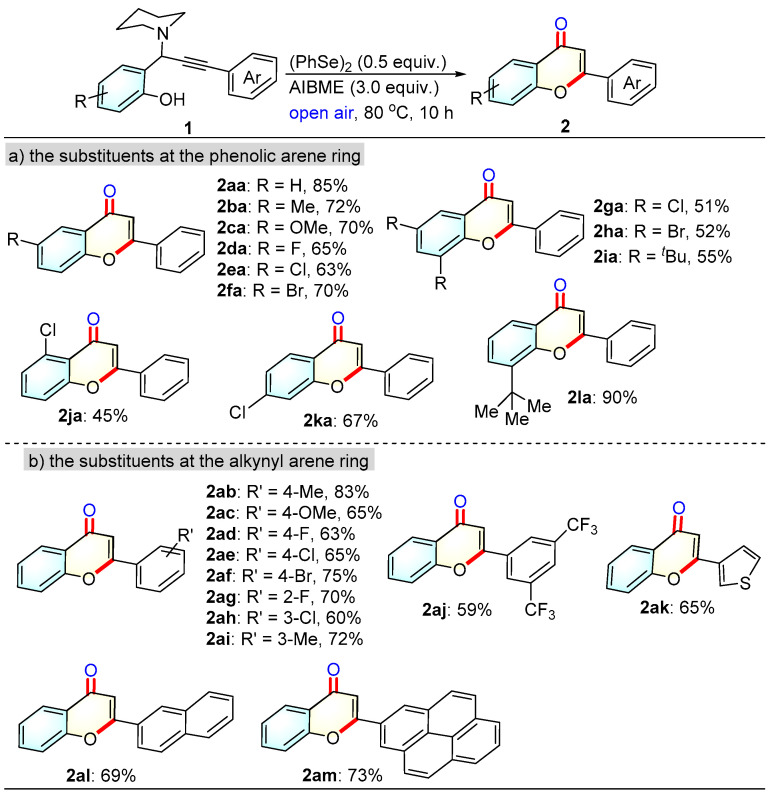
Substituent effect at the phenolic and alkynyl arene rings. Reaction conditions: propargylamines **1** (0.2 mmol), (PhSe)_2_ (0.1 mmol), and AIBME (0.6 mmol) at 80 °C for 10 h under air atmosphere. Isolated yields were reported.

With the optimized conditions in hand, the influence of the substituents at the phenolic or alkynyl arene rings was first evaluated (Scheme 2). Generally, electron-donating (e.g., –Me, –OMe) and electron-withdrawing R groups (e.g., –F, –Cl, –Br) were well tolerated, giving the desired products **2ba**–**2fa** in 63–72% yields. Substrates with multiple halo substituents and a bulky *tert*-butyl group at the *ortho*- and *para*-phenolic position were compatible under this reaction system with slightly lower yields (products **2ga**, **2ha**, and **2ia**). Moreover, substituents at the *meta*-phenolic position were well tolerated, affording the desired products **2ja** and **2ka** in 45% and 67% yields, respectively. Subsequently, the scope and generality of the substituents on the alkynyl arene rings were explored. Substituents with electron-donating groups (–OMe, –Me) and electron-withdrawing groups (–F, –Cl, –Br) at the 2-, 3-, and 4-positions of the benzene rings were well tolerated, affording the corresponding products **2ab–2ai** in 60–83% yields. In particular, trifluoromethyl as a strong electron-withdrawing substituent afforded the desired product **2aj** in a 59% yield. Moreover, reactions with alkenyl-, thienyl-, and pyrenyl-containing substrates proceeded smoothly as well, giving the products **2ka**, **2la**, and **2ma** in 65%, 69%, and 73% yields, respectively. It is notable that the halo moiety, e.g., –F, –Cl, and –Br, located at either the phenolic or alkynyl arene rings, remained intact (products **2da**–**2ha**, **2ja**–**2ka**, **2ad**–**2ah**). These results exhibit an excellent opportunity for further arene functionalization by transition-metal-catalyzed cross-couplings.

**Scheme 3:**
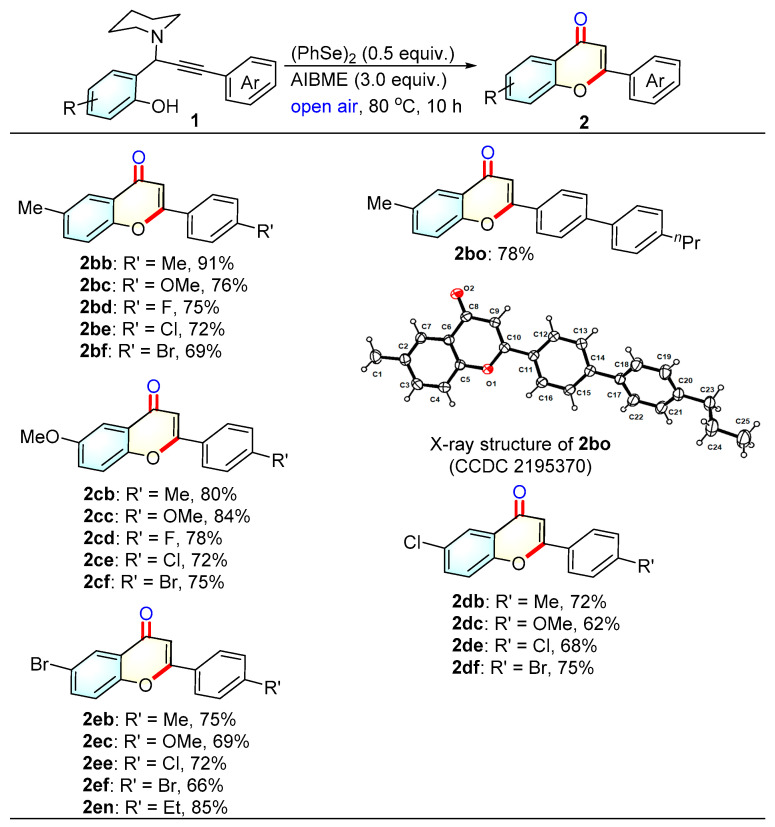
Substrate scope of various propargylamines. Reaction conditions: propargylamines **1** (0.2 mmol), (PhSe)_2_ (0.1 mmol), and AIBME (0.6 mmol) at 80 °C for 10 h under air atmosphere. Isolated yields were reported.

Furthermore, we investigated various propargylamines bearing with different substituents on both the phenolic and alkynyl arene rings to showcase the prospective utility of this protocol (Scheme 3). Substituents with electron-rich (e.g., –Me, –Et, –OMe) groups and electron-deficient (e.g., –F, –Cl, –Br) groups at the phenolic and alkynyl arene rings were well-tolerated. The corresponding products **2bb**–**2en** were obtained in good-to-excellent yields (62–91%). Moreover, the extended π structure did not show an influence, and the desired product **2bo** was successfully obtained in a 78% yield. In addition, the structure of compound **2bo** was unambiguously characterized via single crystal X-ray crystallographic analysis (details appear in Supplementary Materials).

**Scheme 4:**
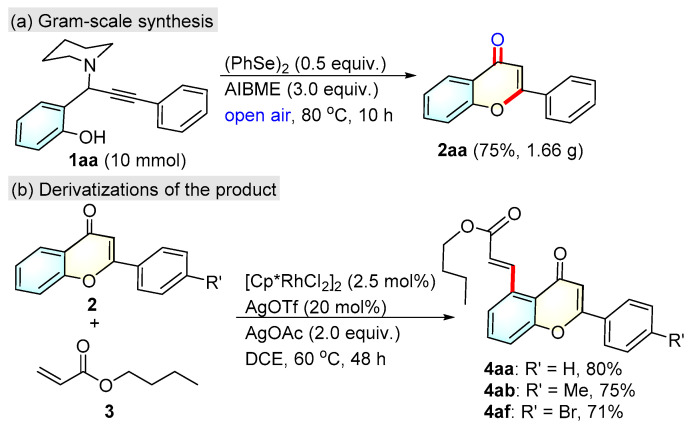
Synthetic applications.

To further prove the robustness and the general utility of this protocol, we carried out the reaction of propargylamine **1aa** on the gram scale under the standard condition. When the reaction was amplified to a large scale (scaled up to 50 times), the protocol worked well, and the corresponding product **2aa** was isolated in a 75% yield (Scheme 4a), which showed promise for this synthetic strategy as a useful tool in practical synthetic terms. Taking advantage of the flavones, we then explored their reactivity in further synthetic transformations. Rhodium-catalyzed oxidative C-H functionalization at the C-5 position of chromones successfully realized the formation of alkenyl flavones **4aa**, **4ab**, and **4af** in 80%, 75%, and 71% yields, respectively (Scheme 4b).

**Scheme 5:**
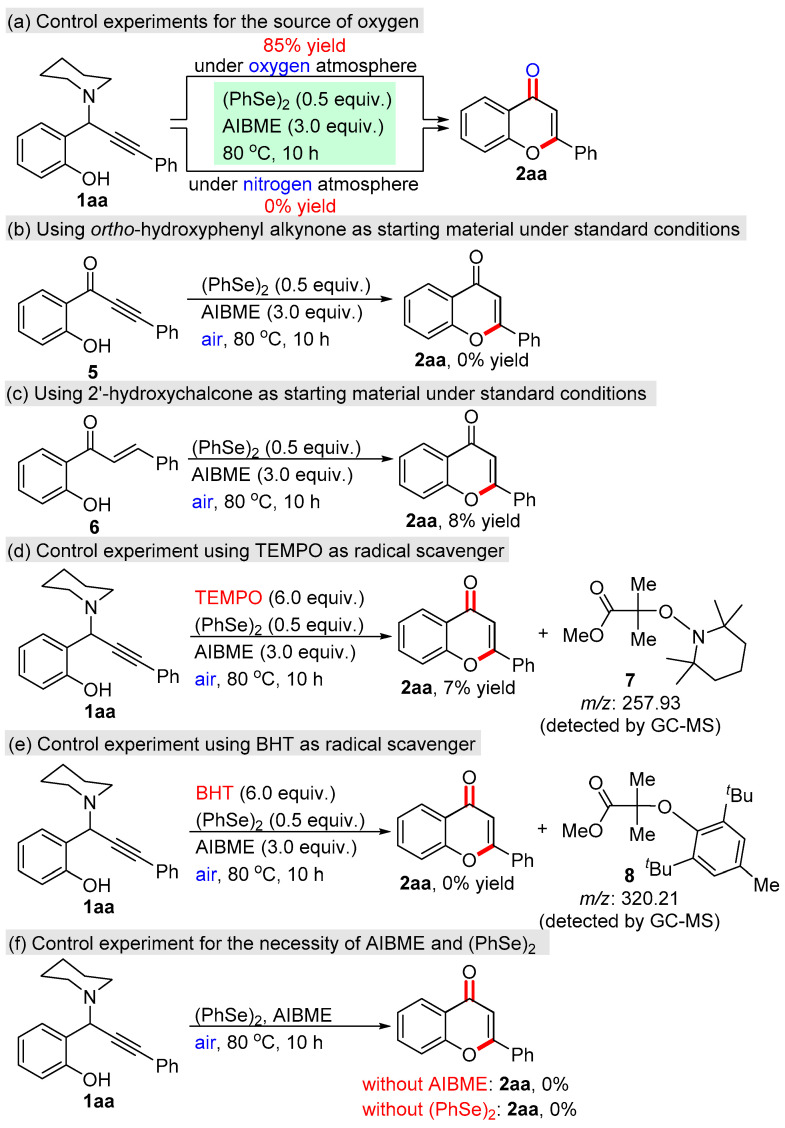
Mechanistic studies.

Insights into this cascade reaction were gained by performing control experiments to clarify the reaction mechanism. To find the source of oxygen, the reaction with propargylamine **1aa** was initially carried out in the presence of an oxygen and nitrogen atmosphere. In both cases, the desired product **2aa** was isolated in 85% and 0% yields, respectively, clearly indicating an oxygen supply from molecular oxygen of air (Scheme 5a). Moreover, the desired product **2aa** was not obtained when the reaction was carried out using *ortho*-hydroxyphenyl alkynone **5** instead of *ortho*-hydroxyphenyl propargylamine **1** under the standard conditions (Scheme 5b). The reaction of 2′-hydroxychalcone **6** was further examined under the standard conditions, providing the desired product **2aa** in an 8% yield (Scheme 5c). Moreover, the addition of radical scavengers, namely (2,2,6,6-tetramethyl-1-piperidinyl)oxyl (TEMPO) and 2,6-di-*tert*-butyl-4-methylphenol (BHT), under the standard conditions significantly inhibited the reaction. The radical-trapping products **7** and **8** were detected by GC-MS, implying that the reaction proceeds via the radical pathway (Scheme 5d,e). Furthermore, the control experiments showed that AIBME and (PhSe)_2_ were both necessary for this transformation (Scheme 5f).

**Scheme 6:**
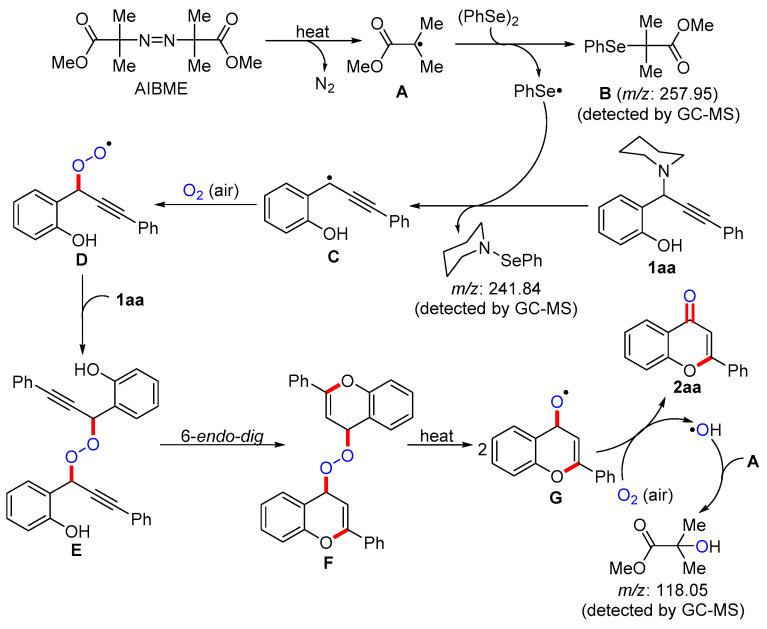
A plausible mechanism for the cascade reaction of proparylamines with air.

Based on the literature reports [65,66,67] and the results of the above control experiments, a plausible mechanism is proposed (Scheme 6). Initially, AIBME releases nitrogen under thermal conditions to form the free radical **A**, which attacks (PhSe)_2_ to generate the phenylselenyl radical (PhSe∙) and compound **B** (detected by CC-Ms) via radical transfer. Phenylselenyl radical then reacts with propargylamine **1aa** to form the radical intermediate **C** and 1-(phenylselanyl)piperidine (detected by GC-MS) through C-N cleavage. The subsequent direct coupling of the intermediate **C** with molecular O_2_ (from air) gives rise to an *O*-radical **D**, which undergoes radical substitution with propargylamine **1aa** to afford intermediate **E**. Then, the intramolecular 6-*endo-dig* annulation of intermediate **E** via nucleophilic addition of the OH group to alkynes gives the intermediate **F**, which undergoes thermal hemolytic cleavage to generate the radical intermediate **G**. Finally, the oxidation of intermediate **G** results in the desired product **2aa** in the presence of air as the sole oxidant, yielding the hydroxyl radical, which could be quenched by the radical intermediate **A** to yield methyl 2-hydroxy-2-methylpropanoate (detected by GC-MS).

## 3. Materials and Methods

The detailed procedures for the synthesis and characterization of the products are given in Appendix A.

## 4. Conclusions

In summary, we established a novel and straightforward metal- and solvent-free cascade reaction of propargylamines with air for the construction of 2-aryl-4*H*-chromen-4-ones with substantial substitution diversity in generally good yields. This cascade process presumably involves a sequence of radical-induced C-N cleavage, followed by C-O coupling, intramolecular 6-*endo-dig* annulation, thermal hemolytic cleavage, and oxidative hydrocarbonylation. The preliminary mechanistic studies suggest that this reaction probably proceeds via a radical pathway. The practical protocol employs air as an oxygen source and represents a simple, economically acceptable, and eco-friendly route toward the straightforward construction of a 2-aryl-4*H*-chromen-4-one skeleton. In addition, the current strategy can be scaled-up to a gram-scale reaction and the synthetic utility of this transformation was also accomplished.

## Figures and Tables

**Table 1:** Optimization of reaction conditions *^a^*.

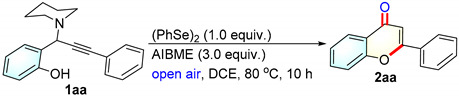		
Entry	Variation from the Standard Conditions	Yield (%) *^b^*
1	none	63
2	MeCN instead of DCE	40
3	toluene instead of DCE	45
4	DMSO instead of DCE	42
5	DMF instead of DCE	35
6	acetone instead of DCE	0
7	0.2 equiv. of (PhSe)_2_ was used	55
8	0.5 equiv. of (PhSe)_2_ was used	71
9	1.5 equiv. of (PhSe)_2_ was used	59
10	2.0 equiv. of (PhSe)_2_ was used	64
11 *^c^*	2.0 equiv. of AIBME was used	44
12 *^c^*	4.0 equiv. of AIBME was used	52
13 *^c^*	AIBN instead of AIBME	37
14 *^c^*	solvent-free	85
15 *^c,d^*	proceeded under blue LED light	78
16 *^c,d^*	At 60 °C	36
17 *^c,d^*	At 100 °C	54
18 *^c,d^*	For 8 h	59
19 *^c,d^*	For 18 h	83

*^a^* Reaction conditions: propargylamine **1aa** (0.2 mmol), (PhSe)_2_ (0.2 mmol), and AIBME (0.6 mmol) in DCE (2 mL) at 80 °C for 10 h under air atmosphere. *^b^* Isolated yields. *^c^* The amount of (PhSe)_2_ was 0.5 equivalent. *^d^* Proceeded under solvent-free conditions.

## Data Availability

The data presented in this study are available in this article, Characterization data for product 3 and 4, including ^1^H- and ^13^C-NMR spectroscopies, are available online. CCDC 2195370 contains the supplementary crystallographic data for this paper. These data can be obtained free of charge via www.ccdc.cam.ac.uk/data_request/cif, or by emailing data_request@ccdc.cam.ac.uk, or by contacting. The Cambridge Crystallographic Data Centre, 12 Union Road, Cambridge CB2 1EZ, UK; Fax: +44-1223-336033.

## Supplementary Materials

The following supporting information can be downloaded at: https://www.mdpi.com/article/10.3390/molecules27217412/s1.

## Sample Availability

Samples of the compounds are available from the authors.

## Appendix A. Experimental Section

Unless otherwise noted, all reagents were purchased from commercial suppliers and used without purification. All cascade reactions were performed in a resealable screw-capped Schlenk flask (approximately a 15 mL volume) in the presence of a Teflon-coated magnetic stirrer bar (4 mm× 10 mm). Reactions were monitored using thin-layer chromatography (TLC) on commercial silica gel plates (GF 254). Visualization of the developed plates was performed under UV lights (GF 254 nm). Flash column chromatography was performed on silica gel (200–300 mesh). ^1^H NMR spectra were recorded on a 400 MHz spectrometer and ^13^C NMR spectra were recorded on a 100 MHz spectrometer. Chemical shifts were expressed in parts per million (*δ*) and the signals were reported as s (singlet), d (doublet), dd (doublet of doublet), t (triplet), q (quartet), and m (multiplet), and coupling constants (*J*) were given in Hz. Chemical shifts as internal standard were referenced to CDCl_3_ (*δ* = 7.26 for ^1^H and *δ* = 77.16 for ^13^C NMR) as internal standard. HRMS analysis with a quadrupole time-of-flight mass spectrometer yielded ion mass/charge (*m/z*) ratios in atomic mass units. The melting points were measured using an SGWX-4 melting point apparatus and were not corrected. The X-ray source used for the single-crystal X-ray diffraction analysis of compound 3na was Mo Kα (λ = 0.71073 Å), and the thermal ellipsoid was drawn at the 30% probability level.

**General procedure for the synthesis of 2-aryl-4*H*-chromen-4-ones 2**. A mixture of propargylamines **1** (0.2 mmol), diphenyl diselenide (0.1 mmol), and dimethyl 2,2′-azobis(2-methylpropionate) (0.6 mmol) were added to a resealable screw-capped Schlenk tube. The resulting mixture was stirred in an oil bath preheated to 80 °C under an open air atmosphere for 10 h (monitored by TLC). Upon completion of the reaction, the reaction mixture was cooled to room temperature, extracted with CH_2_Cl_2_ (3 × 10 mL), and washed with brine. The organic layers were combined, dried over Na_2_SO_4_, filtered, and then evaporated under a vacuum. The residue was purified using flash column chromatography with a silica gel (200–300 mesh), using ethyl acetate and petroleum ether (1:5, *v/v*) as the elution solvent to give the desired products **2**.

**General procedure for the synthesis of compound 4**. A mixture of 2-aryl-4*H*-chromen-4-ones **2** (0.2 mmol), butyl acrylate **3** (0.6 mmol), [Cp*RhCl_2_]_2_ (0.005 mol), AgOTf (0.04 mmol), and AgOAc (0.4 mmol) were added to a resealable screw-capped Schlenk tube. Then 1,2-dichloroethane (2 mL) was added. The tube sealed with a Teflon-coated cap and the resulting mixture was stirred in an oil bath preheated to 60 °C for 48 h (monitored by TLC). Upon completion of the reaction, the reaction mixture was cooled to room temperature, extracted with CH_2_Cl_2_ (3 × 10 mL), and washed with brine. The organic layers were combined, dried over Na_2_SO_4_, filtered, and then evaporated under a vacuum. The residue was purified using flash column chromatography with a silica gel (200–300 mesh) using ethyl acetate and petroleum ether (1:5, *v/v*) as the elution solvent to give the desired products **4aa**, **4ab**, and **4af** in 80%, 75%, and 71% yields, respectively.

*2-Phenyl-4H-chromen-4-one (**2aa**)*. This compound was purified by column chromatography (ethyl acetate/petroleum ether = 1:5, R*_f_* = 0.5) to afford a yellow solid in an 85% yield (38 mg); mp 122–124 °C; ^1^H NMR (400 MHz, CDCl_3_) *δ* 8.24 (dd, *J* = 7.9 Hz, 1.6 Hz, 1H), 7.55–7.93 (m, 2H), 7.74 (td, *J* = 7.7 Hz, 1.7 Hz, 1H), 7.62 (d, *J* = 8.4 Hz, 1H), 7.58–7.53 (m, 3H), 7.46 (t, *J* = 8.0 Hz, 1H), 6.84 (s, 1H); ^13^C NMR (100 MHz, CDCl_3_) *δ* 178.54, 163.45, 156.29, 133.83, 131.80, 131.65, 129.09, 126.33, 125.74, 125.28, 123.98, 118.13, 107.63; HRMS (ESI-TOF) *m/z*: [M + H]^+^ Calcd for C_15_H_11_ O_2_ 223.0754; Found 223.0752.

*6-Methyl-2-phenyl-4H-chromen-4-one (**2ba**)*. This compound was purified by column chromatography (ethyl acetate/petroleum ether = 1:5, R*_f_* = 0.4) to afford a yellow solid in a 72% yield (34mg); mp 100–102 °C; ^1^H NMR (400 MHz, CDCl_3_) *δ* 8.02 (s, 1H), 7.94–7.91 (m, 2H), 7.53 (dd, *J* = 5.3 Hz, 1.8 Hz, 3H), 7.51 (d, *J* = 2.2 Hz, 1H), 7.47 (d, *J* = 8.5 Hz, 1H), 6.82 (s, 1H), 2.47 (s, 3H); ^13^C NMR (100 MHz, CDCl_3_) *δ* 178.60, 163.27, 154.54, 135.20, 134.99, 131.90, 131.49, 129.00, 126.26, 125.04, 123.59, 117.83, 107.44, 20.95; HRMS (ESI-TOF) *m/z*: [M + H]^+^ Calcd for C_16_H_13_O_2_ 237.0910; Found 237.0900.

*6-Methoxy-2-phenyl-4H-chromen-4-one (**2ca**)*. This compound was purified by column chromatography (ethyl acetate/petroleum ether = 1:5, R*_f_* = 0.3) to afford a yellow solid in a 70% yield (35 mg); mp 152–153 °C; ^1^H NMR (400 MHz, CDCl3) *δ* 7.93–7.91 (m, 2H), 7.60 (d, *J* = 3.1 Hz, 1H), 7.53–7.50 (m, 4H), 7.30 (dd, *J* = 9.1 Hz, 3.1 Hz, 1H), 6.82 (s, 1H), 3.91 (s, 3H); ^13^C NMR (100 MHz, CDCl_3_) *δ* 178.34, 163.20, 157.01, 151.10, 131.88, 131.49, 129.02, 126.24, 124.55, 123.83, 119.51, 106.84, 104.83, 55.94; HRMS (ESI-TOF) *m/z*: [M + H]^+^ Calcd for C_16_H_13_O_3_ 253.0859; Found 253.0856.

*6-Fluoro-2-phenyl-4H-chromen-4-one (**2da**)*. This compound was purified by column chromatography (ethyl acetate/petroleum ether = 1:5, R*_f_* = 0.6) to afford a yellow solid in a 65% yield (31 mg); mp 125–127 °C; ^1^H NMR (400 MHz, CDCl_3_) *δ* 7.93–7.91 (m, 2H), 7.87 (dd, *J* = 8.2 Hz, 3.1 Hz, 1H), 7.60–7.51 (m, 4H), 7.45–7.40 (m, 1H), 6.83 (s, 1H); ^13^C NMR (100 MHz, CDCl_3_) *δ* 177.63 (d, *J_C-F_* = 2.4 Hz), 163.69, 159.59 (d, *J_C-F_* = 245.4 Hz), 152.45 (d, *J_C-F_* = 1.7 Hz), 131.79, 131.52, 129.09, 126.31, 125.18 (d, *J_C-F_* = 7.3 Hz), 122.03 (d, *J_C-F_* = 25.3 Hz), 120.19 (d, *J_C-F_* = 8.0 Hz), 110.76 (d, *J_C-F_* = 23.5 Hz), 106.90; ^19^F NMR (376 MHz, CDCl_3_) *δ* -115.08; HRMS (ESI-TOF) *m/z*: [M + H]^+^ Calcd for C_15_H_10_FO_2_ 241.0659; Found 241.0663.

*6-Chloro-2-phenyl-4H-chromen-4-one (**2ea**)*. This compound was purified by column chromatography (ethyl acetate/petroleum ether = 1:5, R*_f_* = 0.6) to afford a yellow solid in a 63% yield (32 mg); mp 181–182 °C; ^1^H NMR (400 MHz, CDCl_3_) *δ* 8.20 (d, *J* = 2.6 Hz, 1H), 7.93–7.90 (m, 2H), 7.65 (dd, *J* = 8.9 Hz, 2.6 Hz, 1H), 7.57–7.52 (m, 4H), 6.84 (s, 1H); ^13^C NMR (100 MHz, CDCl_3_) *δ* 177.22, 163.72, 154.58, 133.97, 131.86, 131.41, 131.21, 129.11, 126.33, 125.19, 124.91, 119.80, 107.48; HRMS (ESI-TOF) *m/z*: [M + H]^+^ Calcd for C_15_H_10_ClO_2_ 257.0364; Found 257.0355.

*6-Bromo-2-phenyl-4H-chromen-4-one (**2fa**)*. This compound was purified by column chromatography (ethyl acetate/petroleum ether = 1:5, R*_f_* = 0.7) to afford a yellow solid in a 70% yield (42 mg); mp 188–190 °C; ^1^H NMR (400 MHz, CDCl_3_) *δ* 8.36 (d, *J* = 2.5 Hz, 1H), 7.91 (dd, *J* = 7.6 Hz, 1.6 Hz, 2H), 7.79 (dd, *J* = 8.9 Hz, 2.5 Hz, 1H), 7.56–7.53 (m, 3H), 7.48 (d, *J* = 8.8 Hz, 1H), 6.83 (s, 1H); ^13^C NMR (100 MHz, CDCl_3_) *δ* 177.01, 163.68, 155.00, 136.71, 131.86, 131.39, 129.11, 128.38, 126.32, 125.28, 120.03, 118.67, 107.56; HRMS (ESI-TOF) *m/z*: [M + H]^+^ Calcd for C_15_H_10_BrO_2_ 300.9859; Found 300.9853.

*6,8-Dichloro-2-phenyl-4H-chromen-4-one (**2ga**)*. This compound was purified by column chromatography (ethyl acetate/petroleum ether = 1:5, R*_f_* = 0.6) to afford a yellow solid in a 51% yield (29 mg); mp 165–167 °C; ^1^H NMR (400 MHz, CDCl_3_) *δ* 8.10 (d, *J* = 2.5 Hz, 1H), 7.99 (dd, *J* = 7.7 Hz, 1.5 Hz, 2H), 7.74 (d, *J* = 2.5 Hz, 1H), 7.68–7.50 (m, 3H), 6.87 (s, 1H); ^13^C NMR (100 MHz, CDCl_3_) *δ* 176.50, 163.50, 150.49, 133.80, 132.20, 130.89, 130.88, 129.23, 126.43, 125.73, 124.50, 123.90, 107.22; HRMS (ESI-TOF) *m/z*: [M + H]^+^ Calcd for C_15_H_9_Cl_2_O_2_ 290.9974; Found 290.9977.

*6,8-Dibromo-2-phenyl-4H-chromen-4-one (**2ha**)*. This compound was purified by column chromatography (ethyl acetate/petroleum ether = 1:5, R*_f_* = 0.4) to afford a yellow solid in a 52% yield (39 mg); mp 166–168 °C; ^1^H NMR (400 MHz, CDCl_3_) *δ* 8.30 (d, *J* = 2.4 Hz, 1H), 8.04 (d, *J* = 2.3 Hz, 1H), 8.01 (dd, *J* = 7.5 Hz, 1.5 Hz, 2H), 7.61–7.52 (m, 3H), 6.87 (s, 1H); ^13^C NMR (100 MHz, CDCl_3_) *δ* 176.45, 163.67, 151.83, 139.36, 132.24, 130.91, 129.27, 127.83, 126.52, 126.04, 118.55, 113.11, 107.18; HRMS (ESI-TOF) *m/z*: [M + H]^+^ Calcd for C_15_H_9_Br_2_O_2_ 378.8964; Found 378.8965.

*6,8-Di-tert-butyl-2-phenyl-4H-chromen-4-one (**2ia**)*. This compound was purified by column chromatography (ethyl acetate/petroleum ether = 1:5, R*_f_* = 0.6) to afford a yellow solid in a 55% yield (36 mg); mp 105–107 °C; ^1^H NMR (400 MHz, CDCl_3_) *δ* 8.14 (d, *J* = 2.5 Hz, 1H), 8.06–7.89 (m, 2H), 7.75 (d, *J* = 2.5 Hz, 1H), 7.55 (t, *J* = 3.2 Hz, 3H), 6.87 (s, 1H), 1.60 (s, 9H), 1.40 (s, 9H); ^13^C NMR (100 MHz, CDCl_3_) *δ* 179.16, 163.08, 153.32, 147.63, 138.40, 132.35, 131.35, 129.17, 128.97, 126.40, 124.18, 119.77, 107.40, 35.27, 35.01, 31.37, 30.29; HRMS (ESI-TOF) *m/z*: [M + H]^+^ Calcd for C_23_H_27_O_2_ 335.2006; Found 335.1996.

*5-Chloro-2-phenyl-4H-chromen-4-one (**2ja**)*. This compound was purified by column chromatography (ethyl acetate/petroleum ether = 1:5, R*_f_* = 0.6) to afford a yellow solid in a 45% yield (23 mg); mp 117–118 °C; ^1^H NMR (400 MHz, CDCl_3_) *δ* 7.92–7.89 (m, 2H), 7.56–7.52 (m, 4H), 7.50 (dd, *J* = 7.6 Hz, 1.4 Hz, 1H), 7.40 (dd, *J* = 7.6 Hz, 1.4 Hz, 1H), 6.79 (s, 1H); ^13^C NMR (100 MHz, CDCl_3_) *δ* 177.23, 161.72, 157.82, 133.51, 132.80, 131.73, 131.05, 129.06, 128.14, 126.16, 121.02, 117.31, 108.86; HRMS (ESI-TOF) *m/z*: [M + H]^+^ Calcd for C_15_H_10_ClO_2_ 257.0364; Found 257.0358.

*7-Chloro-2-phenyl-4H-chromen-4-one (**2ka**)*. This compound was purified by column chromatography (ethyl acetate/petroleum ether = 1:5, R*_f_* = 0.6) to afford a yellow solid in a 67% yield (34 mg); mp 154–156 °C; ^1^H NMR (400 MHz, CDCl_3_) *δ* 8.17 (d, *J* = 8.5 Hz, 1H), 7.91 (dd, *J* = 7.6 Hz, 1.6 Hz, 2H), 7.61 (d, *J* = 1.9 Hz, 1H), 7.56–7.51 (m, 3H), 7.39 (dd, *J* = 8.4 Hz, 2.0 Hz, 1H), 6.82 (s, 1H); ^13^C NMR (100 MHz, CDCl_3_) *δ* 177.52, 163.56, 156.34, 139.76, 131.82, 131.35, 129.10, 127.08, 126.27, 126.07, 122.49, 118.17, 107.77; HRMS (ESI-TOF) *m/z*: [M + H]^+^ Calcd for C_15_H_10_ClO_2_ 257.0364; Found 257.0357.

*8-(tert-Butyl)-2-phenyl-4H-chromen-4-one (**2la**)*. This compound was purified by column chromatography (ethyl acetate/petroleum ether = 1:5, R*_f_* = 0.6) to afford a yellow solid in a 90% yield (50 mg); mp 186–188 °C; ^1^H NMR (400 MHz, CDCl_3_) *δ* 8.16 (dd, *J* = 7.8 Hz, 1.5 Hz, 1H), 8.99–7.96 (m, 2H), 7.69 (dd, *J* = 7.6 Hz, 1.4 Hz, 1H), 7.56 (t, *J* = 3.2 Hz, 3H), 7.36 (t, *J* = 7.7 Hz, 1H), 6.87 (s, 1H), 1.60 (s, 9H); ^13^C NMR (100 MHz, CDCl_3_) *δ* 178.83, 163.36, 155.15, 139.06, 132.23, 131.46, 131.12, 129.19, 126.45, 124.83, 124.80, 124.04, 107.57, 35.11, 30.22; HRMS (ESI-TOF) *m/z*: [M + H]^+^ Calcd for C_19_H_19_O_2_ 279.1380; Found 279.1375.

*2-(p-Tolyl)-4H-chromen-4-one (**2ab**)*. This compound was purified by column chromatography (ethyl acetate/petroleum ether = 1:5, R*_f_* = 0.4) to afford a yellow solid in an 83% yield (39 mg); mp 112–113 °C; ^1^H NMR (400 MHz, CDCl_3_) *δ* 8.25 (dd, *J* = 7.9 Hz, 1.8 Hz, 1H), 7.83 (d, *J* = 8.2 Hz, 2H), 7.71–7.67 (m, 1H), 7.57 (d, *J* = 8.5 Hz, 1H), 7.43–7.39 (m, 1H), 7.33 (d, *J* = 8.0 Hz, 2H), 6.80 (s, 1H), 2.44 (s, 3H); ^13^C NMR (100 MHz, CDCl_3_) *δ* 178.16, 163.30, 155.92, 141.92, 133.32, 129.44, 128.64, 125.91, 125.35, 124.80, 123.66, 117.72, 106.66, 21.21; HRMS (ESI-TOF) *m/z*: [M + H]^+^ Calcd for C_16_H_13_O_2_ 237.0910; Found 237.0920.

*2-(4-Methoxyphenyl)-4H-chromen-4-one (**2ac**)*. This compound was purified by column chromatography (ethyl acetate/petroleum ether = 1:5, R*_f_* = 0.3) to afford a yellow solid in a 65% yield (33 mg); mp 146–147 °C; ^1^H NMR (400 MHz, CDCl_3_) *δ* 8.23 (dd, *J* = 7.9 Hz, 1.7 Hz, 1H), 7.90 (d, *J* = 8.9 Hz, 2H), 7.70–7.66 (m, 1H), 7.55 (d, *J* = 8.4 Hz, 1H), 7.43–7.38 (m, 1H), 7.04 (d, *J* = 8.9 Hz, 2H), 6.75 (s, 1H), 3.89 (s, 3H); ^13^C NMR (100 MHz, CDCl_3_) *δ* 178.42, 163.45, 162.42, 156.21, 133.57, 128.02, 125.69, 125.09, 124.06, 123.95, 117.96, 114.48, 106.21, 55.52; HRMS (ESI-TOF) *m/z*: [M + H]^+^ Calcd for C_16_H_13_O_3_ 253.0859; Found 253.0869.

*2-(4-Fluorophenyl)-4H-chromen-4-one (**2ad**)*. This compound was purified by column chromatography (ethyl acetate/petroleum ether = 1:5, R*_f_* = 0.6) to afford a yellow solid in a 63% yield (30 mg); mp 140–142 °C; ^1^H NMR (400 MHz, CDCl_3_) *δ* 8.23 (dd, *J* = 7.8 Hz, 1.7 Hz, 1H), 7.95–7.90 (m, 2H), 7.72–7.68 (m, 1H), 7.56 (d, *J* = 8.0 Hz, 1H), 7.44–7.40 (m, 1H), 7.23–7.18 (m, 2H), 6.77 (s, 1H); ^13^C NMR (100 MHz, CDCl_3_) *δ* 178.27, 165.99 (d, *J_C-F_* = 251.68 Hz), 162.39, 156.16, 133.82, 128.48 (d, *J_C-F_* = 8.8 Hz), 127.97 (d, *J_C-F_* = 3.3 Hz), 125.73, 125.31, 123.84, 117.99, 116.28 (d, *J_C-F_* = 27.31 Hz), 107.37; ^19^F NMR (376 MHz, CDCl_3_) *δ* −107.48; HRMS (ESI-TOF) *m/z*: [M + H]^+^ Calcd for C_15_H_10_FO_2_ 241.0659; Found 241.0655.

*2-(4-Chlorophenyl)-4H-chromen-4-one (**2ae**)*. This compound was purified by column chromatography (ethyl acetate/petroleum ether = 1:5, R*_f_* = 0.7) to afford a yellow solid in a 65% yield (33 mg); mp 179–180 °C; ^1^H NMR (400 MHz, CDCl_3_) *δ* 8.23 (d, *J* = 7.8 Hz, 1H), 7.86 (d, *J* = 8.4 Hz, 2H), 7.71 (t, *J* = 7.6 Hz, 1H), 7.56 (d, *J* = 8.5 Hz, 1H), 7.50 (d, *J* = 8.5 Hz, 2H), 7.42 (t, *J* = 7.6 Hz, 1H), 6.79 (s, 1H); ^13^C NMR (100 MHz, CDCl_3_) *δ* 178.25, 162.21, 156.14, 137.87, 133.90, 130.22, 129.36, 127.52, 125.73, 125.36, 123.88, 118.02, 107.67; HRMS (ESI-TOF) *m/z*: [M + H]^+^ Calcd for C_15_H_10_ClO_2_ 257.0364; Found 257.0363.

*2-(4-Bromophenyl)-4H-chromen-4-one (**2af**)*. This compound was purified by column chromatography (ethyl acetate/petroleum ether = 1:5, R*_f_* = 0.7) to afford a yellow solid in a 75% yield (45 mg); mp 152–153 °C; ^1^H NMR (400 MHz, CDCl_3_) *δ* 8.23 (dd, *J* = 7.9 Hz, 1.6 Hz, 1H), 7.79 (d, *J* = 8.6 Hz, 2H), 7.73–7.70 (m, 1H), 7.66 (d, *J* = 8.6 Hz, 2H), 7.56 (d, *J* = 8.4 Hz, 1H), 7.45–7.41 (m, 1H), 6.80 (s, 1H); ^13^C NMR (100 MHz, CDCl_3_) *δ* 178.28, 162.30, 156.15, 133.93, 132.34, 130.68, 127.69, 126.31, 125.73, 125.39, 123.88, 118.03, 107.68; HRMS (ESI-TOF) *m/z*: [M + H]^+^ Calcd for C_15_H_10_BrO_2_ 300.9859; Found 300.9866.

*2-(2-Fluorophenyl)-4H-chromen-4-one (**2ag**)*. This compound was purified by column chromatography (ethyl acetate/petroleum ether = 1:5, R*_f_* = 0.4) to afford a yellow solid in a 70% yield (33 mg); mp 97–98 °C; ^1^H NMR (400 MHz, CDCl_3_) *δ* 8.24 (dd, *J* = 7.9 Hz, 1.7 Hz, 1H), 7.95–7.91 (m, 1H), 7.71–7.68 (m, 1H), 7.55–7.50 (m, 2H), 7.45–7.41 (m, 1H), 7.34–7.30 (m, 1H), 7.23–7.20 (m, 1H), 6.94 (s, 1H); ^13^C NMR (100 MHz, CDCl_3_) *δ* 178.42, 161.82 (d, *J_C-F_* = 354.4 Hz), 158.81 (d, *J_C-F_* = 3.8 Hz), 156.37, 133.89, 132.90 (d, *J_C-F_* = 9.0 Hz), 129.08, 129.07, 125.76, 125.30, 124.64 (d, *J_C-F_* = 3.8 Hz), 123.84, 120.38 (d, *J_C-F_* = 10.1 Hz), 118.08, 116.99 (d, *J_C-F_* = 22.4 Hz), 112.44 (d, *J_C-F_* = 11.2 Hz); ^19^F NMR (376 MHz, CDCl_3_) *δ*-110.82; HRMS (ESI-TOF) *m/z*: [M + H]^+^ Calcd for C_15_H_10_FO_2_ 241.0659; Found 241.0669.

*2-(3-Chlorophenyl)-4H-chromen-4-one (**2ah**)*. This compound was purified by column chromatography (ethyl acetate/petroleum ether = 1:5, R*_f_* = 0.5) to afford a yellow solid in a 60% yield (30 mg); mp 110–112 °C; ^1^H NMR (400 MHz, CDCl_3_) *δ* 8.23 (dd, *J* = 7.9 Hz, 1.7 Hz, 1H), 7.93 (t, *J* = 1.9 Hz, 1H), 7.80–7.78 (m, 1H), 7.74–7.70 (m, 1H), 7.59 (d, *J* = 8.3 Hz, 1H), 7.53–7.50 (m, 1H), 7.48–7.42 (m, 2H), 6.81 (s, 1H); ^13^C NMR (100 MHz, CDCl_3_) *δ* 178.24, 161.79, 156.16, 135.26, 134.00, 133.59, 131.51, 130.32, 126.36, 125.76, 125.44, 124.38, 123.92, 118.09, 108.17; HRMS (ESI-TOF) *m/z*: [M + H]^+^ Calcd for C_15_H_10_ClO_2_ 257.0364; Found 257.0366.

*2-(m-Tolyl)-4H-chromen-4-one (**2ai**)*. This compound was purified by column chromatography (ethyl acetate/petroleum ether = 1:5, R*_f_* = 0.6) to afford a yellow solid in a 72% yield (34 mg); mp 108–109 °C; ^1^H NMR (400 MHz, CDCl_3_) *δ* 8.24 (dd, *J* = 8.0 Hz, 1.7 Hz, 1H), 7.74–7.72 (m, 2H), 7.70–7.68 (m, 1H), 7.59 (d, *J* = 8.1 Hz, 1H), 7.44–7.39 (m, 2H), 7.36 (d, *J* = 7.6 Hz, 1H), 6.82 (s, 1H), 2.47 (s, 3H); ^13^C NMR (100 MHz, CDCl_3_) *δ* 178.47, 163.64, 156.29, 138.84, 133.70, 132.40, 131.76, 128.93, 126.86, 125.70, 125.17, 123.99, 123.51, 118.08, 107.57, 21.51; HRMS (ESI-TOF) *m/z*: [M + H]^+^ Calcd for C_16_H_13_O_2_ 237.0910; Found 237.0909.

*2-(3,5-Bis(trifluoromethyl)phenyl)-4H-chromen-4-one (**2aj**)*. This compound was purified by column chromatography (ethyl acetate/petroleum ether = 1:5, R*_f_* = 0.6) to afford a yellow solid in a 59% yield (42 mg); mp 154–155 °C; ^1^H NMR (400 MHz, CDCl_3_) *δ* 8.36 (s, 2H), 8.25 (dd, *J* = 8.0 Hz, 1.6 Hz, 1H), 8.05 (s, 1H), 7.79–7.75 (m, 1H), 7.65 (d, *J* = 8.2 Hz, 1H), 7.50–7.46 (m, 1H), 6.92 (s, 1H); ^13^C NMR (100 MHz, CDCl_3_) *δ* 177.80, 159.81, 156.09, 134.41, 134.14, 133.32 (q, *J_C-F_* = 33.7 Hz), 126.91, 126.26, 126.22, 125.90, 125.88, 124.89 (q, *J_C-F_* = 3.6 Hz), 124.75, 124.20, 123.89, 121.49, 118.77, 118.18, 109.21; ^19^F NMR (376 MHz, CDCl_3_) *δ*-62.96; HRMS (ESI-TOF) *m/z*: [M + H]^+^ Calcd for C_17_H_9_F_6_O_2_ 359.0501; Found 359.0499.

*2-(Thiophen-3-yl)-4H-chromen-4-one (**2ak**)*. This compound was purified by column chromatography (ethyl acetate/petroleum ether = 1:5, R*_f_* = 0.5) to afford a yellow solid in a 65% yield (29 mg); mp 107–108 °C; ^1^H NMR (400 MHz, CDCl_3_) *δ* 8.22 (dd, *J* = 7.9 Hz, 1.8 Hz, 1H), 8.03 (dd, *J* = 3.0 Hz, 1.3 Hz, 1H), 7.70–7.66 (m, 1H), 7.53 (d, *J* = 8.3 Hz, 1H), 7.50 (dd, *J* = 5.2, 1.4 Hz, 1H), 7.46 (dd, *J* = 5.1, 3.0 Hz, 1H), 7.43–7.38 (m, 1H), 6.68 (s, 1H); ^13^C NMR (100 MHz, CDCl_3_) *δ* 178.44, 159.53, 156.04, 134.20, 133.70, 127.36, 126.82, 125.67, 125.14, 125.04, 123.97, 117.94, 107.16; HRMS (ESI-TOF) *m/z*: [M + H]^+^ Calcd for C_13_H_9_O_2_S 229.0318; Found 229.0319.

*2-(Naphthalen-2-yl)-4H-chromen-4-one (**2al**)*. This compound was purified by column chromatography (ethyl acetate/petroleum ether = 1:5, R*_f_* = 0.5) to afford a yellow solid in a 69% yield (37 mg); mp 100–102 °C; ^1^H NMR (400 MHz, CDCl_3_) *δ* 8.32 (dd, *J* = 8.0 Hz, 1.7 Hz, 1H), 8.15–8.13 (m, 1H), 8.04 (d, *J* = 8.2 Hz, 1H), 7.97–7.94 (m, 1H), 7.78 (dd, *J* = 7.2 Hz, 1.2 Hz, 1H), 7.73–7.70 (m, 1H), 7.61–7.56 (m, 3H), 7.54 (d, *J* = 8.1 Hz, 1H), 7.50–7.46 (m, 1H), 6.70 (s, 1H); ^13^C NMR (100 MHz, CDCl_3_) *δ* 178.28, 165.43, 156.72, 133.87, 133.72, 131.50, 130.65, 130.38, 128.72, 127.95, 127.43, 126.58, 125.86, 125.37, 125.06, 124.87, 124.02, 118.24, 113.07; HRMS (ESI-TOF) *m/z*: [M + H]^+^ Calcd for C_19_H_13_O_2_ 273.0910; Found 273.0900.

*2-(Pyren-2-yl)-4H-chromen-4-one (**2am**)*. This compound was purified by column chromatography (ethyl acetate/petroleum ether = 1:5, R*_f_* = 0.6) to afford a yellow solid in a 73% yield (50 mg); mp 216–219 °C; ^1^H NMR (400 MHz, CDCl_3_) *δ* 8.43 (d, *J* = 9.3 Hz, 1H), 8.35 (d, *J* = 7.9 Hz, 1H), 8.27–8.24 (m, 4H), 8.18–8.15 (m, 2H), 8.11–8.05 (m, 2H), 7.75 (t, *J* = 7.8 Hz, 1H), 7.60 (d, *J* = 8.4 Hz, 1H), 7.50 (t, *J* = 7.6 Hz, 1H), 6.85 (s, 1H); ^13^C NMR (100 MHz, CDCl_3_) *δ* 178.17, 165.54, 156.84, 133.86, 133.05, 131.12, 130.49, 129.21, 129.13, 128.93, 127.11, 127.05, 126.70, 126.49, 126.25, 126.00, 125.85, 125.37, 124.77, 124.61, 124.33, 123.96, 123.80, 118.24, 113.69; HRMS (ESI-TOF) *m/z*: [M + H]^+^ Calcd for C_25_H_15_O_2_ 347.1067; Found 347.1070.

*6-Methyl-2-(p-tolyl)-4H-chromen-4-one (**2bb**).* This compound was purified by column chromatography (ethyl acetate/petroleum ether = 1:5, R*_f_* = 0.6) to afford a yellow solid in a 91% yield (45 mg); mp 136–137 °C; ^1^H NMR (400 MHz, CDCl_3_) *δ* 8.04 (s, 1H), 7.84 (d, *J* = 8.3 Hz, 2H), 7.53 (dd, *J* = 8.6 Hz, 1.9 Hz, 1H), 7.49 (d, *J* = 8.5 Hz, 1H), 7.35 (d, *J* = 8.1 Hz, 2H), 6.81 (s, 1H), 2.49 (s, 3H), 2.46 (s, 3H); ^13^C NMR (100 MHz, CDCl_3_) *δ* 178.60, 163.46, 154.50, 142.11, 135.07, 134.86, 129.72, 129.05, 126.18, 125.01, 123.60, 117.79, 106.81, 21.52, 20.94; HRMS (ESI-TOF) *m/z*: [M + H]^+^ Calcd for C_17_H_15_O_2_ 251.1067; Found 251.1072.

*2-(4-Methoxyphenyl)-6-methyl-4H-chromen-4-one (**2bc**)*. This compound was purified by column chromatography (ethyl acetate/petroleum ether = 1:5, R*_f_* = 0.8) to afford a yellow solid in a 76% yield (40 mg); mp 162–163 °C; ^1^H NMR (400 MHz, CDCl_3_) *δ* 8.01 (s, 1H), 7.88 (d, *J* = 8.9 Hz, 2H), 7.49 (dd, *J* = 8.6 Hz, 1.8 Hz, 1H), 7.45 (d, *J* = 8.5 Hz, 1H), 7.02 (d, *J* = 8.9 Hz, 2H), 6.73 (s, 1H), 3.89 (s, 3H), 2.47 (s, 3H); ^13^C NMR (100 MHz, CDCl_3_) *δ* 178.54, 163.31, 162.32, 154.47, 135.03, 134.76, 127.97, 125.03, 124.18, 123.57, 117.70, 114.43, 106.06, 55.50, 20.93; HRMS (ESI-TOF) *m/z*: [M + H]^+^ Calcd for C_17_H_15_O_3_ 267.1016; Found 267.1020.

*2-(4-Fluorophenyl)-6-methyl-4H-chromen-4-one (**2bd**)*. This compound was purified by column chromatography (ethyl acetate/petroleum ether = 1:5, R*_f_* = 0.4) to afford a yellow solid in a 75% yield (38 mg); mp 124–126 °C; ^1^H NMR (400 MHz, CDCl_3_) *δ* 8.01 (s, 1H), 7.95–7.89 (m, 2H), 7.51 (dd, *J* = 8.6 Hz, 2.2 Hz, 1H), 7.46 (d, *J* = 8.5 Hz, 1H), 7.25–7.16 (m, 2H), 6.75 (s, 1H), 2.47 (s, 3H); ^13^C NMR (100 MHz, CDCl_3_) *δ* 178.44, 164.69 (d, *J_C-F_* = 251.9 Hz), 162.28, 154.45, 135.33, 135.06, 128.45 (d, *J_C-F_* = 8.8 Hz), 128.09 (d, *J_C-F_* = 3.3 Hz), 125.07, 123.48, 117.75, 116.25 (d, *J_C-F_* = 22.1 Hz), 107.21, 20.94; ^19^F NMR (376 MHz, CDCl_3_) *δ* -107.67; HRMS (ESI-TOF) *m/z*: [M + H]^+^ Calcd for C_16_H_12_FO_2_ 255.0816; Found 255.0811.

*2-(4-Chlorophenyl)-6-methyl-4H-chromen-4-one (**2be**)*. This compound was purified by column chromatography (ethyl acetate/petroleum ether = 1:5, R*_f_* = 0.7) to afford a yellow solid in a 72% yield (38 mg); mp 165–167 °C; ^1^H NMR (400 MHz, CDCl_3_) *δ* 8.02 (s, 1H), 7.87 (d, *J* = 8.6 Hz, 2H), 7.52 (t, *J* = 8.6 Hz, 2H), 7.49 (s, 1H), 7.46 (d, *J* = 8.6 Hz, 1H), 6.78 (s, 1H), 2.48 (s, 3H); ^13^C NMR (100 MHz, CDCl_3_) *δ* 178.42, 162.12, 154.47, 137.78, 135.41, 135.14, 130.39, 129.35, 127.53, 125.10, 123.56, 117.79, 107.55, 20.95; HRMS (ESI-TOF) *m/z*: [M + H]^+^ Calcd for C_16_H_12_ClO_2_ 271.0520; Found 271.0512.

*2-(4-Bromophenyl)-6-methyl-4H-chromen-4-one (**2bf**)*. This compound was purified by column chromatography (ethyl acetate/petroleum ether = 1:5, R*_f_* = 0.4) to afford a yellow solid in a 69% yield (43 mg); mp 196–198 °C; ^1^H NMR (400 MHz, CDCl_3_) *δ* 8.00 (s, 1H), 7.78 (d, *J* = 8.6 Hz, 2H), 7.65 (d, *J* = 8.7 Hz, 2H), 7.51 (dd, *J* = 8.6 Hz, 2.2 Hz, 1H), 7.45 (d, *J* = 8.6 Hz, 1H), 6.77 (s, 1H), 2.46 (s, 1H); ^13^C NMR (100 MHz, CDCl_3_) *δ* 178.34, 162.10, 154.41, 135.38, 135.11, 132.28, 130.79, 127.64, 126.16, 125.06, 123.53, 117.77, 107.51, 20.93; HRMS (ESI-TOF) *m/z*: [M + H]^+^ Calcd for C_16_H_12_BrO_2_ 315.0015; Found 315.0017.

*6-Methyl-2-(4′-propyl-[1,1′-biphenyl]-4-yl)-4H-chromen-4-one (**2bo**)*. This compound was purified by column chromatography (ethyl acetate/petroleum ether = 1:5, R*_f_* = 0.4) to afford a yellow solid in a 78% yield (55 mg); mp 152–153 °C; ^1^H NMR (400 MHz, CDCl_3_) *δ* 8.02 (s, 1H), 7.98 (d, *J* = 8.4 Hz, 2H), 7.73 (d, *J* = 8.5 Hz, 2H), 7.57 (d, *J* = 8.2 Hz, 2H), 7.54–7.42 (m, 2H), 7.29 (d, *J* = 8.2 Hz, 2H), 6.85 (s, 1H), 2.78–2.55 (m, 2H), 2.47 (s, 3H), 1.70 (d, *J* = 7.5 Hz, 2H), 0.99 (t, *J* = 7.3 Hz, 3H); ^13^C NMR (100 MHz, CDCl_3_) *δ* 178.52, 163.07, 154.52, 144.23, 142.95, 137.04, 135.16, 134.93, 130.25, 129.10, 127.35, 126.92, 126.66, 125.04, 123.63, 117.80, 107.14, 37.70, 24.49, 20.93, 13.84; HRMS (ESI-TOF) *m/z*: [M + H]^+^ Calcd for C_25_H_23_O_2_ 355.1693; Found 355.1691.

*6-Methoxy-2-(p-tolyl)-4H-chromen-4-one (**2cb**)*. This compound was purified by column chromatography (ethyl acetate/petroleum ether = 1:5, R*_f_* = 0.3) to afford a yellow solid in an 80% yield (42 mg); mp 147–148 °C; ^1^H NMR (400 MHz, CDCl_3_) *δ* 7.82 (d, *J* = 8.3 Hz, 2H), 7.60 (d, *J* = 3.1 Hz, 1H), 7.50 (d, *J* = 9.1 Hz, 1H), 7.32 (d, *J* = 8.1 Hz, 2H), 7.29 (dd, *J* = 9.1 Hz, 3.1 Hz, 1H), 6.79 (s, 1H), 3.91 (s, 3H), 2.44 (s, 3H); ^13^C NMR (100 MHz, CDCl_3_) *δ* 178.34, 163.38, 156.92, 151.05, 142.11, 129.74, 129.04, 126.16, 124.56, 123.69, 119.47, 106.25, 104.80, 55.94, 21.53; HRMS (ESI-TOF) *m/z*: [M + H]^+^ Calcd for C_17_H_15_O_3_ 267.1016; Found 267.1026.

*6-Methoxy-2-(4-methoxyphenyl)-4H-chromen-4-one (**2cc**)*. This compound was purified by column chromatography (ethyl acetate/petroleum ether = 1:5, R*_f_* = 0.3) to afford a yellow solid in an 84% yield (48 mg); mp 187–188 °C; ^1^H NMR (400 MHz, CDCl_3_) *δ* 7.87 (d, *J* = 8.9 Hz, 2H), 7.59 (d, *J* = 3.1 Hz, 1H), 7.48 (d, *J* = 9.1 Hz, 1H), 7.33–7.22 (m, 1H), 7.02 (d, *J* = 8.9 Hz, 2H), 6.74 (s, 1H), 3.91 (s, 3H), 3.89 (s, 3H); ^13^C NMR (100 MHz, CDCl_3_) *δ* 178.23, 163.19, 162.30, 156.88, 150.98, 127.91, 124.49, 124.12, 123.53, 119.35, 114.42, 105.47, 104.86, 55.92, 55.48; HRMS (ESI-TOF) *m/z*: [M + H]^+^ Calcd for C_17_H_15_O_4_ 283.0965; Found 283.0971.

*2-(4-Fluorophenyl)-6-methoxy-4H-chromen-4-one (**2cd**)*. This compound was purified by column chromatography (ethyl acetate/petroleum ether = 1:5, R*_f_* = 0.5) to afford a yellow solid in a 78% yield (42 mg); mp 144–146 °C; ^1^H NMR (400 MHz, CDCl_3_) *δ* 7.94–7.89 (m, 2H), 7.59 (d, *J* = 3.1 Hz, 1H), 7.50 (d, *J* = 9.2 Hz, 1H), 7.29 (dd, *J* = 9.2 Hz, 3.2 Hz, 1H), 7.24–7.17 (m, 2H), 6.76 (s, 1H), 3.91 (s, 3H); ^13^C NMR (100 MHz, CDCl_3_) *δ* 178.17, 165.94 (d, *J* = 251.5 Hz), 162.19, 157.06, 150.99, 128.42 (d, *J* = 8.8 Hz), 128.07 (d, *J* = 3.3 Hz), 124.45, 123.87, 119.42, 116.26 (d, *J* = 22.0 Hz), 106.63, 104.87, 55.95; ^19^F NMR (376 MHz, CDCl_3_) *δ*-107.68; HRMS (ESI-TOF) *m/z*: [M + H]^+^ Calcd for C_16_H_12_FO_3_ 271.0765; Found 271.0759.

*2-(4-Chlorophenyl)-6-methoxy-4H-chromen-4-one (**2ce**)*. This compound was purified by column chromatography (ethyl acetate/petroleum ether = 1:5, R*_f_* = 0.5) to afford a yellow solid in a 72% yield (41 mg); mp 167–168 °C; ^1^H NMR (400 MHz, CDCl_3_) *δ* 7.85 (d, *J* = 8.6 Hz, 2H), 7.58 (d, *J* = 3.1 Hz, 1H), 7.49 (d, *J* = 8.8 Hz, 3H), 7.29 (dd, *J* = 9.2 Hz, 3.2 Hz, 1H), 6.78 (s, 1H), 3.91 (s, 3H); ^13^C NMR (100 MHz, CDCl_3_) *δ* 178.14, 161.98, 157.09, 150.96, 137.75, 130.30, 129.33, 127.46, 124.49, 123.94, 119.45, 106.89, 104.84, 55.94; HRMS (ESI-TOF) *m/z*: [M + H]^+^ Calcd for C_16_H_12_ClO_3_ 287.0469; Found 287.0475.

*2-(4-Bromophenyl)-6-methoxy-4H-chromen-4-one (**2cf**)*. This compound was purified by column chromatography (ethyl acetate/petroleum ether = 1:5, R*_f_* = 0.6) to afford a yellow solid in a 75% yield (49 mg); mp 189–190 °C; ^1^H NMR (400 MHz, CDCl_3_) *δ* 7.78 (d, *J* = 8.7 Hz, 2H), 7.65 (d, *J* = 8.7 Hz, 2H), 7.58 (d, *J* = 3.1 Hz, 1H), 7.49 (d, *J* = 9.1 Hz, 1H), 7.29 (dd, *J* = 9.2 Hz, 3.2 Hz, 1H), 6.78 (s, 1H), 3.91 (s, 3H); ^13^C NMR (100 MHz, CDCl_3_) *δ* 178.12, 162.04, 157.09, 150.95, 132.30, 130.77, 127.62, 126.16, 124.50, 123.95, 119.46,106.90, 104.84, 55.94; HRMS (ESI-TOF) *m/z*: [M + H]^+^ Calcd for C_16_H_12_BrO_3_ 330.9964; Found 330.9960.

*6-Chloro-2-(p-tolyl)-4H-chromen-4-one (**2db**)*. This compound was purified by column chromatography (ethyl acetate/petroleum ether = 1:5, R*_f_* = 0.5) to afford a yellow solid in a 72% yield (38 mg); mp 177–178 °C; ^1^H NMR (400 MHz, CDCl_3_) *δ* 8.19 (d, *J* = 2.6 Hz, 1H), 7.81 (d, *J* = 8.3 Hz, 2H), 7.63 (dd, *J* = 8.9 Hz, 2.6 Hz, 1H), 7.52 (d, *J* = 8.9 Hz, 1H), 7.33 (d, *J* = 8.0 Hz, 2H), 6.79 (s, 1H), 2.44 (s, 3H); ^13^C NMR (100 MHz, CDCl_3_) *δ* 177.20, 163.89, 154.53, 142.58, 133.82, 131.08, 129.83, 128.55, 126.25, 125.14, 124.92, 119.75, 106.83, 21.55; HRMS (ESI-TOF) *m/z*: [M + H]^+^ Calcd for C_16_H_12_ClO_2_ 271.0520; Found 271.0527.

*6-Chloro-2-(4-methoxyphenyl)-4H-chromen-4-one (**2dc**)*. This compound was purified by column chromatography (ethyl acetate/petroleum ether = 1:5, R*_f_* = 0.5) to afford a yellow solid in a 62% yield (35 mg); mp 173–174 °C; ^1^H NMR (400 MHz, CDCl_3_) *δ* 8.18 (d, *J* = 2.6 Hz, 1H), 7.87 (d, *J* = 9.0 Hz, 2H), 7.62 (dd, *J* = 8.9, 2.6 Hz, 1H), 7.51 (d, *J* = 8.9 Hz, 1H), 7.03 (d, *J* = 9.0 Hz, 2H), 6.74 (s, 1H), 3.90 (s, 3H); ^13^C NMR (100 MHz, CDCl_3_) *δ* 177.10, 163.70, 162.61, 154.49, 133.71, 131.02, 128.06, 125.15, 124.91, 123.62, 119.66, 114.54, 106.05, 55.52; HRMS (ESI-TOF) *m/z*: [M + H]^+^ Calcd for C_16_H_12_ClO_3_ 287.0469; Found 287.0474.

*6-Chloro-2-(4-chlorophenyl)-4H-chromen-4-one (**2de**)*. This compound was purified by column chromatography (ethyl acetate/petroleum ether = 1:5, R*_f_* = 0.3) to afford a yellow solid in a 68% yield (39 mg); mp 202–203 °C; ^1^H NMR (400 MHz, CDCl_3_) *δ* 8.19 (d, *J* = 2.5 Hz, 1H), 7.85 (d, *J* = 8.7 Hz, 2H), 7.65 (dd, *J* = 8.9 Hz, 2.6 Hz, 1H), 7.52 (dd, *J* = 8.9 Hz, 5.8 Hz, 3H), 6.79 (s, 1H); ^13^C NMR (100 MHz, CDCl_3_) *δ* 176.98, 162.50, 154.46, 138.19, 134.10, 131.38, 129.86, 129.46, 127.56, 125.22, 124.85, 119.75, 107.57; HRMS (ESI-TOF) *m/z*: [M + H]^+^ Calcd for C_15_H_9_Cl_2_O_2_ 290.9974; Found 290.9977.

*2-(4-Bromophenyl)-6-chloro-4H-chromen-4-one (**2df**)*. This compound was purified by column chromatography (ethyl acetate/petroleum ether = 1:5, R*_f_* = 0.4) to afford a yellow solid in a 75% yield (50 mg); mp 200–202 °C; ^1^H NMR (400 MHz, CDCl_3_) *δ* 8.19 (d, *J* = 2.4 Hz, 1H), 7.78 (d, *J* = 8.8 Hz, 2H), 7.72–7.60 (m, 3H), 7.53 (d, *J* = 8.9 Hz, 1H), 6.80 (s, 1H); ^13^C NMR (100 MHz, CDCl_3_) *δ* 176.98, 162.58, 154.46, 134.11, 132.43, 131.39, 130.32, 127.71, 126.62, 125.23, 124.86, 119.76, 107.59; HRMS (ESI-TOF) *m/z*: [M + H]^+^ Calcd for C_15_H_9_BrClO_2_ 334.9469; Found 334.9462.

*6-Bromo-2-(p-tolyl)-4H-chromen-4-one (**2eb**)*. This compound was purified by column chromatography (ethyl acetate/petroleum ether = 1:5, R*_f_* = 0.5) to afford a yellow solid in a 75% yield (47 mg); mp 185–186 °C; ^1^H NMR (400 MHz, CDCl_3_) *δ* 8.35 (d, *J* = 2.5 Hz, 1H), 7.81 (d, *J* = 8.2 Hz, 2H), 7.77 (dd, *J* = 8.8 Hz, 2.5 Hz, 1H), 7.46 (d, *J* = 8.8 Hz, 1H), 7.33 (d, *J* = 8.0 Hz, 2H), 6.80 (s, 1H), 2.44 (s, 3H); ^13^C NMR (100 MHz, CDCl_3_) *δ* 177.05, 163.90, 155.00, 142.61, 136.60, 129.85, 128.57, 128.37, 126.27, 125.33, 120.01, 118.57, 106.95, 21.58; HRMS (ESI-TOF) *m/z*: [M + H]^+^ Calcd for C_16_H_12_BrO_2_ 315.0015; Found 315.0024.

*6-Bromo-2-(4-methoxyphenyl)-4H-chromen-4-one (**2ec**)*. This compound was purified by column chromatography (ethyl acetate/petroleum ether = 1:5, R*_f_* = 0.5) to afford a yellow solid in a 69% yield (45 mg); mp 187–188 °C; ^1^H NMR (400 MHz, CDCl_3_) *δ* 8.34 (d, *J* = 2.5 Hz, 1H), 7.88–7.84 (m, 2H), 7.76 (dd, *J* = 8.8 Hz, 2.5 Hz, 1H), 7.44 (d, *J* = 8.8 Hz, 1H), 7.04–7.00 (m, 2H), 6.74 (s, 1H), 3.89 (s, 1H); ^13^C NMR (100 MHz, CDCl_3_) *δ* 176.94, 163.70, 162.62, 154.92, 136.48, 128.34, 128.06, 125.27, 123.59, 119.89, 118.49, 114.54, 106.11, 55.52; HRMS (ESI-TOF) *m/z*: [M + H]^+^ Calcd for C_16_H_12_BrO_3_ 330.9964; Found 330.9970.

*6-Bromo-2-(4-chlorophenyl)-4H-chromen-4-one (**2ee**)*. This, 136.87, 129.85, 129.47, 128.43, 127.57, 125.22, 119.99, 118.84, 107.67. HRMS (ESI-TO compound was purified by column chromatography (ethyl acetate/petroleum ether = 1:5, R*_f_* = 0.6) to afford a yellow solid in a 72% yield (48 mg); mp 214–215 °C; ^1^H NMR (400 MHz, CDCl_3_) *δ* 8.35 (d, *J* = 2.5 Hz, 1H), 7.85 (d, *J* = 8.5 Hz, 2H), 7.79 (dd, *J* = 8.8 Hz, 2.5 Hz, 1H), 7.51 (d, *J* = 8.6 Hz, 2H), 7.47 (d, *J* = 8.9 Hz, 1H), 6.80 (s, 1H); ^13^C NMR (100 MHz, CDCl_3_) *δ* 176.84, 162.51, 154.91, 138.20, 136.87, 129.85, 129.47, 128.43, 127.57, 125.22, 119.99, 118.84, 107.67. HRMS (ESI-TOF) *m/z*: [M + H]^+^ Calcd for C_15_H_9_ClBrO_2_ 334.9469; Found 334.9474.

*6-Bromo-2-(4-bromophenyl)-4H-chromen-4-one (**2ef**)*. This compound was purified by column chromatography (ethyl acetate/petroleum ether = 1:5, R*_f_* = 0.6) to afford a yellow solid in a 66% yield (49 mg); mp 219–220 °C; ^1^H NMR (400 MHz, CDCl_3_) *δ* 8.35 (d, *J* = 2.5 Hz, 1H), 7.80–7.77 (m, 2H), 7.76 (s, 1H), 7.67 (d, *J* = 8.7 Hz, 2H), 7.46 (d, *J* = 8.9 Hz, 1H), 6.80 (s, 1H); ^13^C NMR (100 MHz, CDCl_3_) *δ* 176.83, 162.57, 154.89, 136.87, 132.43, 130.30, 128.42, 127.70, 126.64, 125.22, 119.99, 118.85, 107.67; HRMS (ESI-TOF) *m/z*: [M + H]^+^ Calcd for C_15_H_9_Br_2_O_2_ 378.8964; Found 378.8970.

*6-Bromo-2-(4-ethylphenyl)-4H-chromen-4-one (**2en**).* This compound was purified by column chromatography (ethyl acetate/petroleum ether = 1:5, R*_f_* = 0.5) to afford a yellow solid in an 85% yield (55 mg); mp 129–130 °C; ^1^H NMR (400 MHz, CDCl_3_) *δ* 8.36 (d, *J* = 2.4 Hz, 1H), 7.84 (d, *J* = 8.4 Hz, 2H), 7.78 (dd, *J* = 8.8 Hz, 2.5 Hz, 1H), 7.47 (d, *J* = 8.8 Hz, 1H), 7.36 (d, *J* = 8.4 Hz, 2H), 6.81 (s, 1H), 2.74 (d, *J* = 7.6 Hz, 2H), 1.29 (d, *J* = 7.6 Hz, 3H); ^13^C NMR (100 MHz, CDCl_3_) *δ* 177.08, 163.95, 155.01, 148.84, 136.60, 128.78, 128.67, 128.36, 126.40, 125.32, 120.01, 118.57, 106.98, 28.85, 15.23; HRMS (ESI-TOF) *m/z*: [M + H]^+^ Calcd for C_17_H_14_BrO_2_ 329.0172; Found 329.0169.

*Butyl (E)-3-(4-oxo-2-phenyl-4H-chromen-5-yl)acrylate (**4aa**)*. This compound was purified by column chromatography (ethyl acetate/petroleum ether = 1:5, R*_f_* = 0.6) to afford a yellow solid in an 80% yield (55 mg); mp 93–94 °C; ^1^H NMR (400 MHz, CDCl_3_) *δ* 9.03 (d, *J* = 15.9 Hz, 1H), 7.92 (d, *J* = 8.0 Hz, 2H), 7.66 (t, *J* = 7.9 Hz, 1H), 7.60 (d, *J* = 8.3 Hz, 1H), 7.54 (d, *J* = 6.3 Hz, 3H), 7.47 (d, *J* = 7.2 Hz, 1H), 6.81 (s, 1H), 6.27 (d, *J* = 15.9 Hz, 1H), 4.24 (t, *J* = 6.6 Hz, 2H), 1.91–1.67 (m, 2H), 1.55–1.38 (m, 2H), 0.97 (t, *J* = 7.3 Hz, 3H); ^13^C NMR (100 MHz, CDCl_3_) *δ* 179.58, 166.61, 162.21, 157.21, 144.58, 137.06, 133.03, 131.70, 131.23, 129.06, 126.22, 124.74, 121.67, 121.43, 119.54, 108.72, 64.52, 30.74, 19.21, 13.80; HRMS (ESI-TOF) *m/z*: [M + H]^+^ Calcd for C_22_H_21_O_4_ 349.1434; Found 349.1435.

*Butyl €-3-(4-oxo-2-(p-tolyl)-4H-chromen-5-yl)acrylate (**4ab**)*. This compound was purified by column chromatography (ethyl acetate/petroleum ether = 1:5, R*_f_* = 0.6) to afford a yellow solid in a 75% yield (54 mg); mp 128–129 °C; ^1^H NMR (400 MHz, CDCl_3_) *δ* 9.04 (d, *J* = 15.9 Hz, 1H), 7.81 (d, *J* = 8.2 Hz, 2H), 7.69–7.62 (m, 1H), 7.59 (dd, *J* = 8.3 Hz, 1.0 Hz, 1H), 7.46 (d, *J* = 7.3 Hz, 1H), 7.32 (d, *J* = 8.0 Hz, 2H), 6.76 (s, 1H), 6.26 (d, *J* = 15.9 Hz, 1H), 4.24 (t, *J* = 6.7 Hz, 2H), 2.44 (s, 3H), 1.80–1.70 (m, 2H), 1.55–1.36 (m, 2H), 0.97 (t, *J* = 7.4 Hz, 3H); ^13^C NMR (100 MHz, CDCl_3_) *δ* 179.58, 166.63, 162.39, 157.16, 144.66, 142.36, 136.99, 132.89, 129.76, 128.35, 126.13, 124.63, 121.55, 121.43, 119.52, 108.07, 64.50, 30.73, 21.55, 19.20, 13.79; HRMS (ESI-TOF) *m/z*: [M + H]^+^ Calcd for C_23_H_23_O_4_ 363.1591; Found 363.1593.

*Butyl (E)-3-(2-(4-bromophenyl)-4-oxo-4H-chromen-5-yl)acrylate (**4af**)*. This compound was purified by column chromatography (ethyl acetate/petroleum ether = 1:5, R*_f_* = 0.6) to afford a yellow solid in a 71% yield (60 mg); mp 156–157 °C; ^1^H NMR (400 MHz, CDCl_3_) *δ* 9.01 (d, *J* = 15.9 Hz, 1H), 7.78 (d, *J* = 8.6 Hz, 2H), 7.72–7.64 (m, 3H), 7.59 (d, *J* = 7.6 Hz, 1H), 7.48 (d, *J* = 7.4 Hz, 1H), 6.77 (s, 1H), 6.27 (d, *J* = 15.9 Hz, 1H), 4.24 (t, *J* = 6.7 Hz, 2H), 1.84–1.67 (m, 2H), 1.54–1.40 (m, 2H), 0.97 (t, *J* = 7.4 Hz, 3H); ^13^C NMR (100 MHz, CDCl_3_) *δ* 179.37, 166.57, 161.13, 157.10, 144.41, 137.11, 133.18, 132.36, 130.15, 127.62, 126.41, 124.88, 121.80, 121.37, 119.48, 108.81, 64.55, 30.73, 19.21, 13.79; HRMS (ESI-TOF) *m/z*: [M + H]^+^ Calcd for C_22_H_22_BrO_4_ 427.0539; Found 427.0547.
