# Supplementary material for: Radical-Induced Cascade Annulation/Hydrocarbonylation for Construction of 2-Aryl-4H-chromen-4-ones
Source: Molecules. 2022 Nov 1;27(21):7412. doi: 10.3390/molecules27217412 (PMC9654733; doi:10.3390/molecules27217412)
Supplement: Supplementary file 1 [file molecules-27-07412-s001.zip › molecules-1987694-supplementary.pdf]

Supporting Information

Radical-Induced Cascade Annulation/Hydrocarbonylation for Construction of 2-Aryl-4*H*-chromen-4-ones

Xinwei He *, Keke Xu, Yanan Liu, Demao Wang, Qiang Tang, Wenjie Hui, Haoyu Chen and Yongjia Shang

Key Laboratory of Functional Molecular Solids, Ministry of Education, Anhui Laboratory of Molecule-Based Materials (State Key Laboratory Cultivation Base), College of Chemistry and Materials Science, Anhui Normal University, Wuhu 241000, China
* Correspondence: xinweihe@mail.ahnu.edu.cn

Table of contents

1. X-ray crystallographic data of compound **2bo**--S2

2. NMR spectra for all compounds--S3

3. GC-MS spectra for mechanistic investigations--S56

1. X-ray crystallographic data of compound 2bo

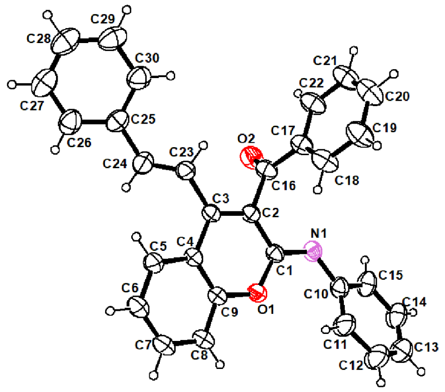

The purified compound **2bo** is dissolved in a mixed solvent of ethyl acetate and petroleum ether, and placed in a dark cabinet to slowly evaporate. After several days, a colourless bulk crystal was obtained. The X-ray crystal-structure determinations were obtained on a Bruker Smart CCDC APEX-2 diffractometer (graphite- monochromated Mo *Kα* radiation, λ=0.71073 nm) at 296(2) K.

Figure S1. ORTEP drawing of compound **2bo** (30% probability for the thermal ellipsoid).

Table S1. Crystal data and structure refinement for compound **2bo**.

CCDC number	2195370	
Identification code	210920b_0m_a	
Empirical formula	C25 H22 O2	
Formula weight	354.42	
Temperature	293.15 K	
Wavelength	0.71073 Å	
Crystal system	Monoclinic	
Space group	$P2_1/c$	
Unit cell dimensions	a = 10.684(3) Å	α= 90°.
	b = 9.951(2) Å	β= 93.881(3)°.
	c = 18.236(4) Å	γ= 90°.
Volume	1934.3(8) $Å^3$	
Z	4	
Density (calculated)	1.217 g/cm^3	
Absorption coefficient	0.076 mm^{-1}	
F(000)	752.0	
Crystal size	0.21 × 0.2 × 0.19 mm^3	
2Θ range for data collection	3.82 to 55.092°	
Index ranges	$-13 \leq h \leq 13$, $-12 \leq k \leq 12$, $-23 \leq l \leq 23$	
Reflections collected	21949	
Independent reflections	4407 [R(int) = 0.0427, R(sigma) = 0.0413]	
Data / restraints / parameters	4407 / 0 / 246	
Goodness-of-fit on F^2	1.049	
Final R indices [I>2sigma(I)]	R1 = 0.0507, wR2 = 0.1302	
Final R indices (all data)	R1 = 0.0946, wR2 = 0.1521	
Largest diff. peak and hole	0.20 and -0.17 $eÅ^{-3}$	

2. NMR spectra for all compounds

2-Phenyl-4*H*-chromen-4-one (2aa)

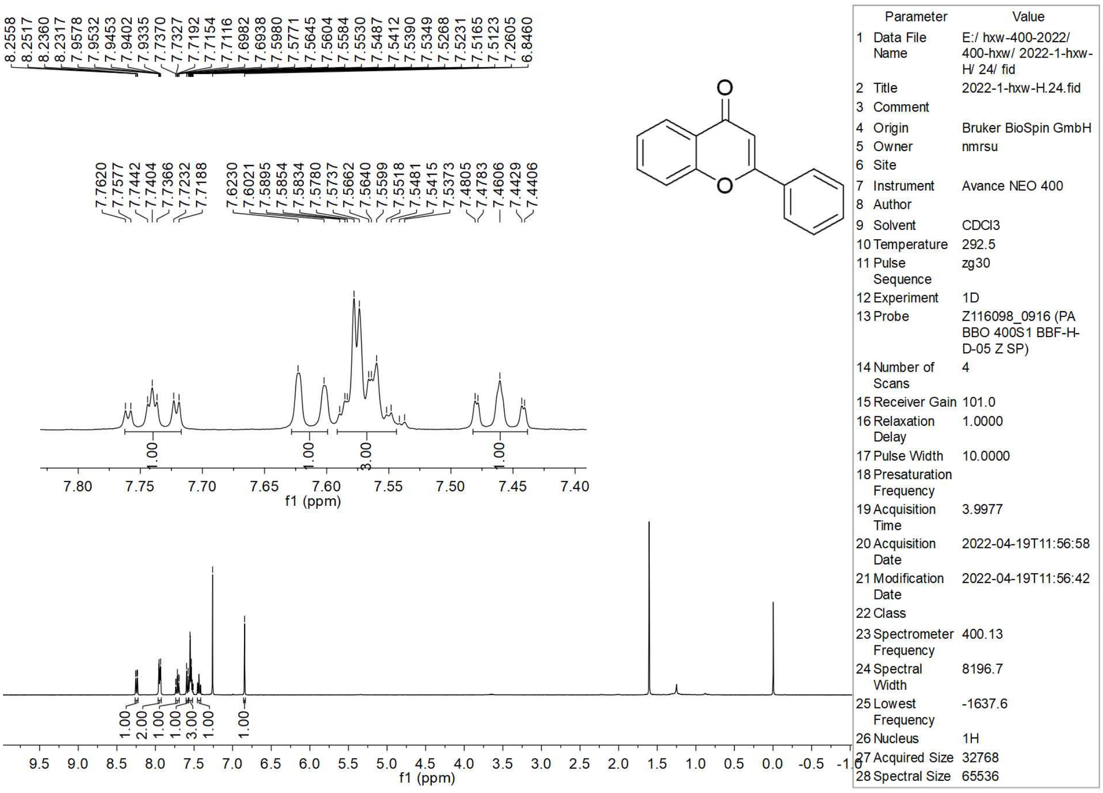

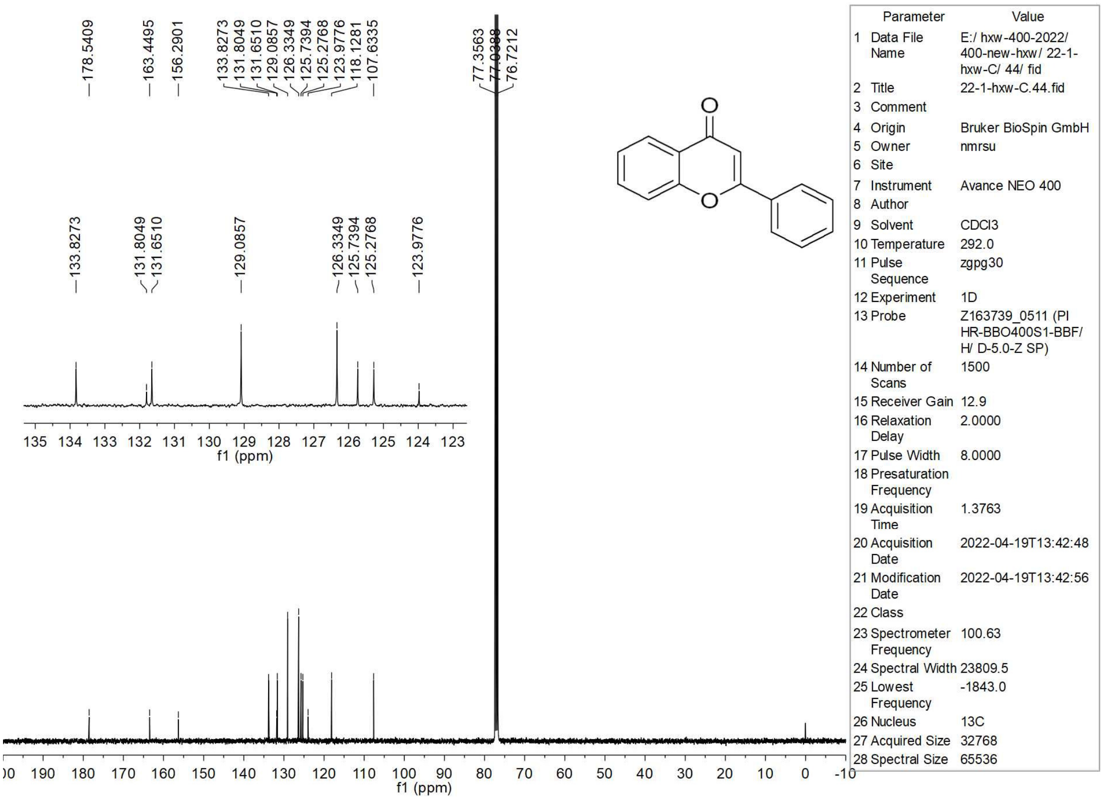

6-Methyl-2-phenyl-4*H*-chromen-4-one (2ba)

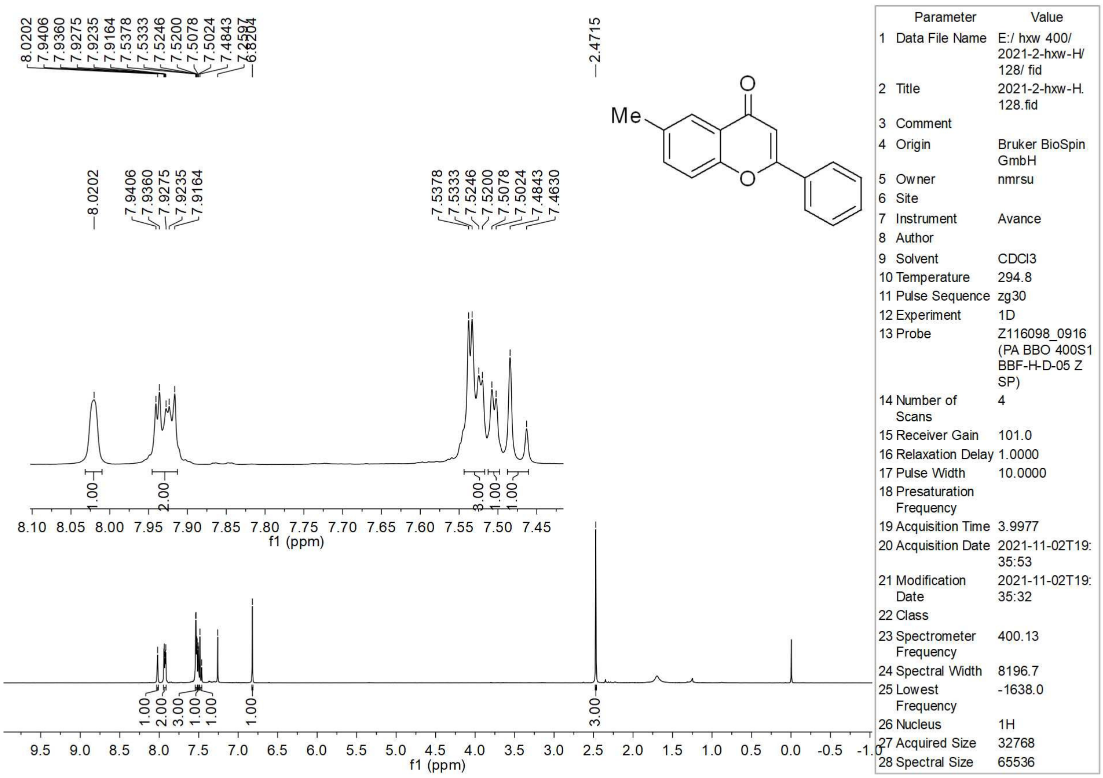

	Parameter	Value
1	Data File Name	E:/ hxw 400/ 2021-2-hxw-H/ 128/ fid
2	Title	2021-2-hxw-H.128.fid
3	Comment	
4	Origin	Bruker BioSpin GmbH
5	Owner	nmrsu
6	Site	
7	Instrument	Avance
8	Author	
9	Solvent	CDCl3
10	Temperature	294.8
11	Pulse Sequence	zg30
12	Experiment	1D
13	Probe	Z116098_0916 (PA BBO 400S1 BBF-H-D-05 Z SP)
14	Number of Scans	4
15	Receiver Gain	101.0
16	Relaxation Delay	1.0000
17	Pulse Width	10.0000
18	Presaturation Frequency	
19	Acquisition Time	3.9977
20	Acquisition Date	2021-11-02T19:35:53
21	Modification Date	2021-11-02T19:35:32
22	Class	
23	Spectrometer Frequency	400.13
24	Spectral Width	8196.7
25	Lowest Frequency	-1638.0
26	Nucleus	1H
27	Acquired Size	32768
28	Spectral Size	65536

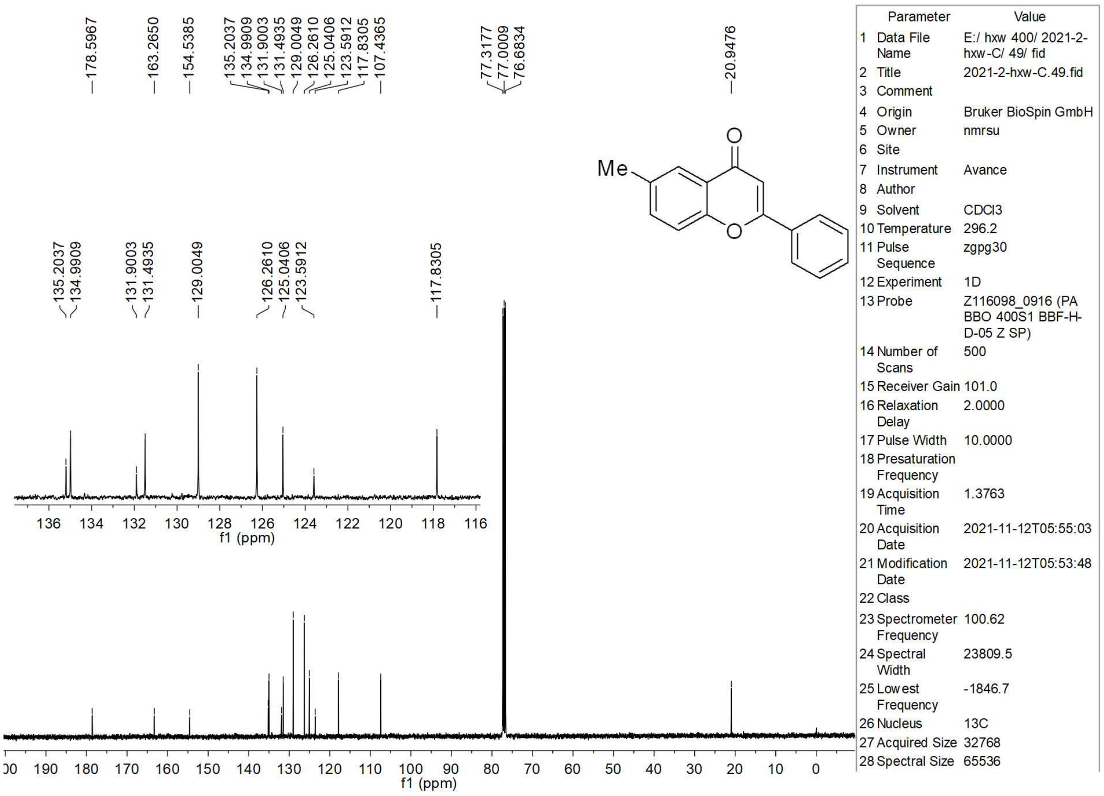

	Parameter	Value
1	Data File Name	E:/ hxw 400/ 2021-2-hxw-C/ 49/ fid
2	Title	2021-2-hxw-C.49.fid
3	Comment	
4	Origin	Bruker BioSpin GmbH
5	Owner	nmrsu
6	Site	
7	Instrument	Avance
8	Author	
9	Solvent	CDCl3
10	Temperature	296.2
11	Pulse Sequence	zgpg30
12	Experiment	1D
13	Probe	Z116098_0916 (PA BBO 400S1 BBF-H-D-05 Z SP)
14	Number of Scans	500
15	Receiver Gain	101.0
16	Relaxation Delay	2.0000
17	Pulse Width	10.0000
18	Presaturation Frequency	
19	Acquisition Time	1.3763
20	Acquisition Date	2021-11-12T05:55:03
21	Modification Date	2021-11-12T05:53:48
22	Class	
23	Spectrometer Frequency	100.62
24	Spectral Width	23809.5
25	Lowest Frequency	-1846.7
26	Nucleus	13C
27	Acquired Size	32768
28	Spectral Size	65536

6-Methoxy-2-phenyl-4*H*-chromen-4-one (2ca)

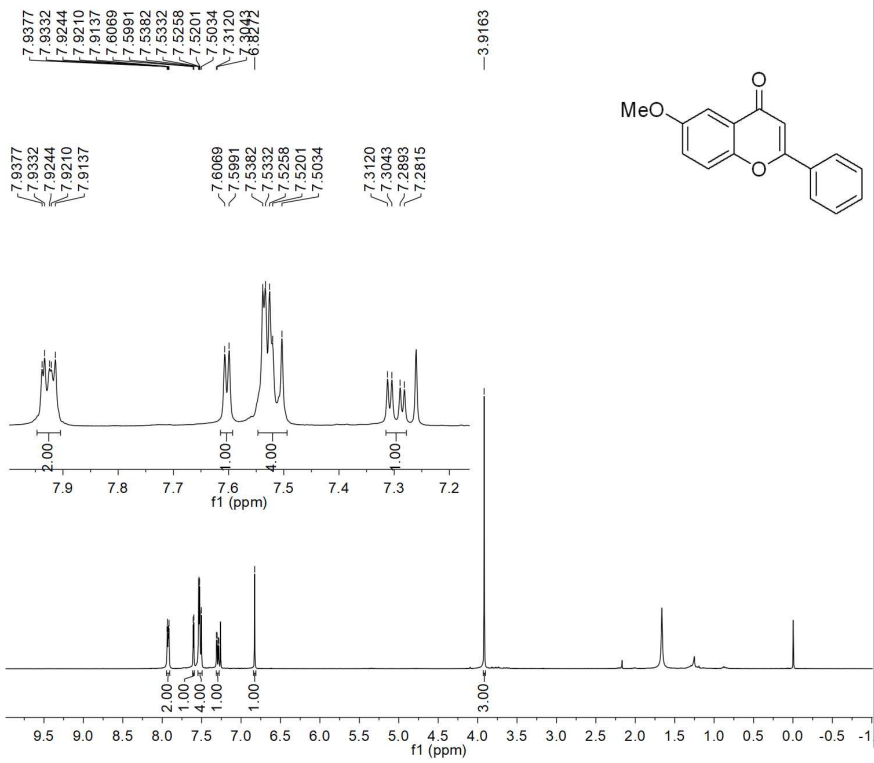

Parameter	Value
1 Data File Name	E:/ hxw 400/ 2021-2-hxw-H/ 5/ fid
2 Title	2021-2-hxw-H. 5.fid
3 Comment	
4 Origin	Bruker BioSpin GmbH
5 Owner	nmrsu
6 Site	
7 Instrument	Avance
8 Author	
9 Solvent	CDCl3
10 Temperature	296.9
11 Pulse Sequence	zg30
12 Experiment	1D
13 Probe	Z163739_0032 (PI HR-400-S1-BBF/ H/ D-5.0-Z SP)
14 Number of Scans	4
15 Receiver Gain	101.0
16 Relaxation Delay	1.0000
17 Pulse Width	10.0000
18 Presaturation Frequency	
19 Acquisition Time	3.9977
20 Acquisition Date	2021-07-28T11:41:08
21 Modification Date	2021-07-28T11:40:22
22 Class	
23 Spectrometer Frequency	400.13
24 Spectral Width	8196.7
25 Lowest Frequency	-1637.2
26 Nucleus	1H
27 Acquired Size	32768
28 Spectral Size	65536

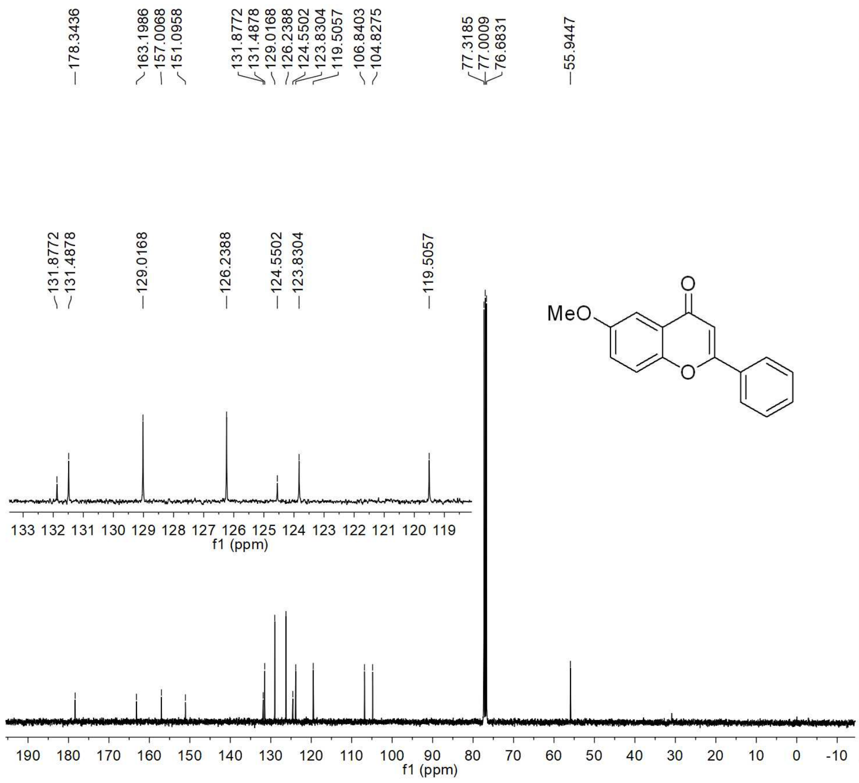

Parameter	Value
1 Data File Name	E:/ hxw 400/ 2021-2-hxw-C/ 4/ fid
2 Title	2021-2-hxw-C.4.fid
3 Comment	
4 Origin	Bruker BioSpin GmbH
5 Owner	nmrsu
6 Site	
7 Instrument	Avance
8 Author	
9 Solvent	CDCl3
10 Temperature	297.9
11 Pulse Sequence	zgpg30
12 Experiment	1D
13 Probe	Z163739_0032 (PI HR-400-S1-BBF/ H/ D-5.0-Z SP)
14 Number of Scans	353
15 Receiver Gain	47.3
16 Relaxation Delay	2.0000
17 Pulse Width	10.0000
18 Presaturation Frequency	
19 Acquisition Time	1.3763
20 Acquisition Date	2021-07-28T14:56:37
21 Modification Date	2021-07-28T14:55:52
22 Class	
23 Spectrometer Frequency	100.62
24 Spectral Width	23809.5
25 Lowest Frequency	-1846.1
26 Nucleus	13C
27 Acquired Size	32768
28 Spectral Size	65536

6-Fluoro-2-phenyl-4*H*-chromen-4-one (2da)

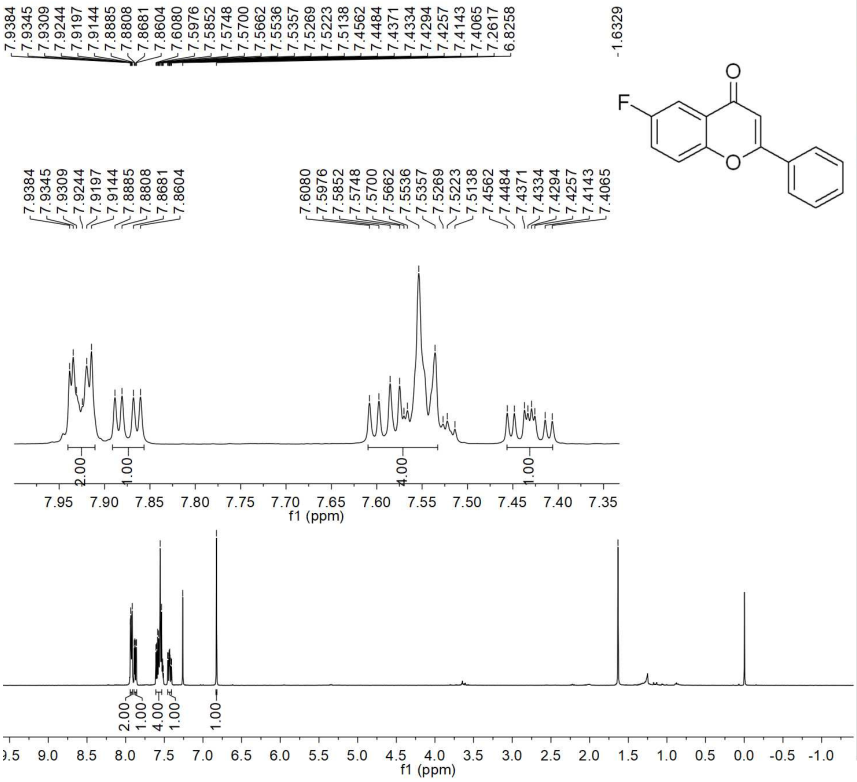

Parameter		Value
1	Data File Name	E:/ hxw 400/ 2021-2-hxw-H/ 117/ fid
2	Title	2021-2-hxw-H.117.fid
3	Comment	
4	Origin	Bruker BioSpin GmbH
5	Owner	nmrsu
6	Site	
7	Instrument	Avance
8	Author	
9	Solvent	CDCl3
10	Temperature	294.7
11	Pulse Sequence	zg30
12	Experiment	1D
13	Probe	Z116098_0916 (PA BBO 400S1 BBF-H-D-05 Z SP)
14	Number of Scans	4
15	Receiver Gain	101.0
16	Relaxation Delay	1.0000
17	Pulse Width	10.0000
18	Presaturation Frequency	
19	Acquisition Time	3.9977
20	Acquisition Date	2021-10-29T20:30:46
21	Modification Date	2021-10-29T20:30:46
22	Class	
23	Spectrometer Frequency	400.13
24	Spectral Width	8196.7
25	Lowest Frequency	-1637.2
26	Nucleus	1H
27	Acquired Size	32768
28	Spectral Size	65536

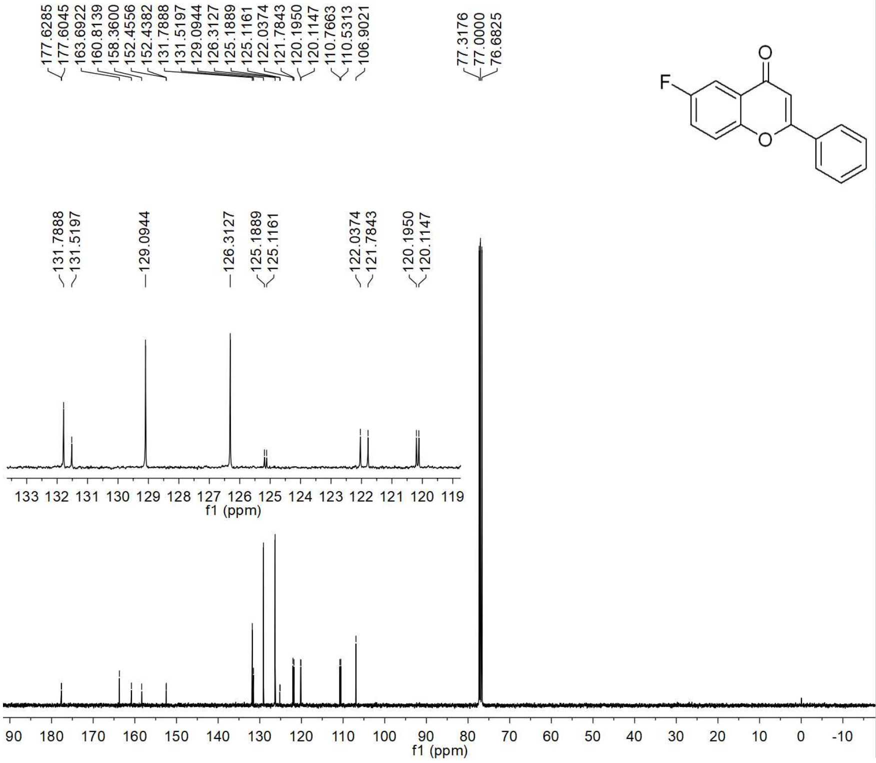

Parameter		Value
1	Data File Name	E:/ hxw 400/ 2021-2-hxw-C/ 46/ fid
2	Title	2021-2-hxw-C.46.fid
3	Comment	
4	Origin	Bruker BioSpin GmbH
5	Owner	nmrsu
6	Site	
7	Instrument	Avance
8	Author	
9	Solvent	CDCl3
10	Temperature	296.3
11	Pulse Sequence	zgpg30
12	Experiment	1D
13	Probe	Z116098_0916 (PA BBO 400S1 BBF-H-D-05 Z SP)
14	Number of Scans	800
15	Receiver Gain	101.0
16	Relaxation Delay	2.0000
17	Pulse Width	10.0000
18	Presaturation Frequency	
19	Acquisition Time	1.3763
20	Acquisition Date	2021-11-12T04:17:25
21	Modification Date	2021-11-12T04:16:10
22	Class	
23	Spectrometer Frequency	100.62
24	Spectral Width	23809.5
25	Lowest Frequency	-1846.6
26	Nucleus	13C
27	Acquired Size	32768
28	Spectral Size	65536

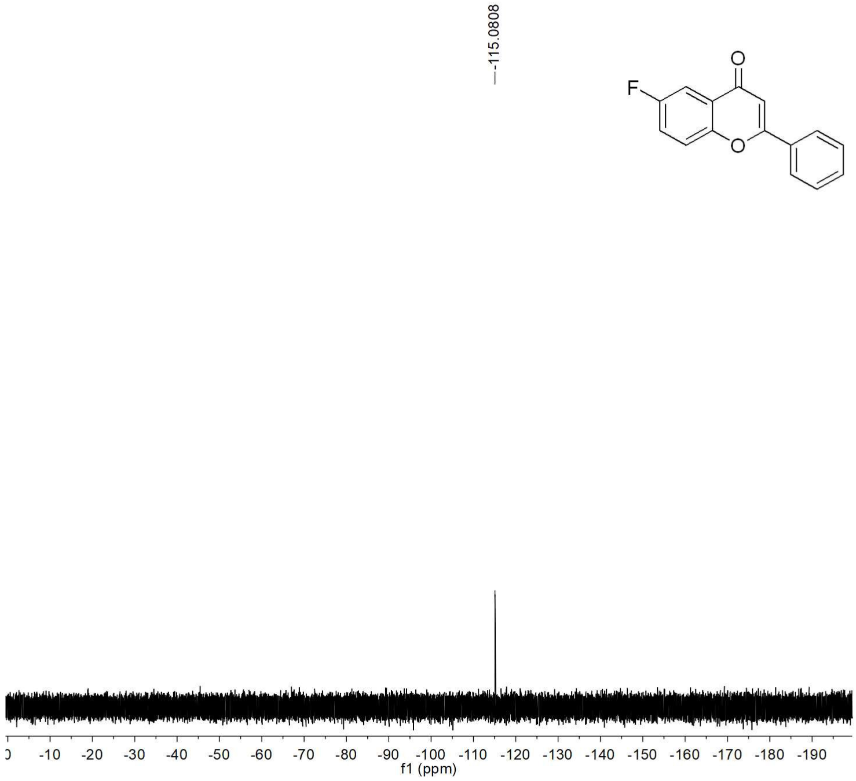

	Parameter	Value
1	Data File Name	E:/ hxw 400/ 2021-2-hxw-F/ 4/ fid
2	Title	2021-2-hxw-F.4.fid
3	Comment	
4	Origin	Bruker BioSpin GmbH
5	Owner	nmrsu
6	Site	
7	Instrument	Avance
8	Author	
9	Solvent	CDCl3
10	Temperature	294.8
11	Pulse Sequence	zgig
12	Experiment	1D
13	Probe	Z116098_0916 (PA BBO 400S1 BBF-H-D-05 Z SP)
14	Number of Scans	16
15	Receiver Gain	11.3
16	Relaxation Delay	1.0000
17	Pulse Width	19.7500
18	Presaturation Frequency	
19	Acquisition Time	0.7209
20	Acquisition Date	2021-10-29T20:52:13
21	Modification Date	2021-10-29T20:52:14
22	Class	
23	Spectrometer Frequency	376.46
24	Spectral Width	90909.1
25	Lowest Frequency	-83104.4
26	Nucleus	19F
27	Acquired Size	65536
28	Spectral Size	131072

6-Chloro-2-phenyl-4*H*-chromen-4-one (2ea)

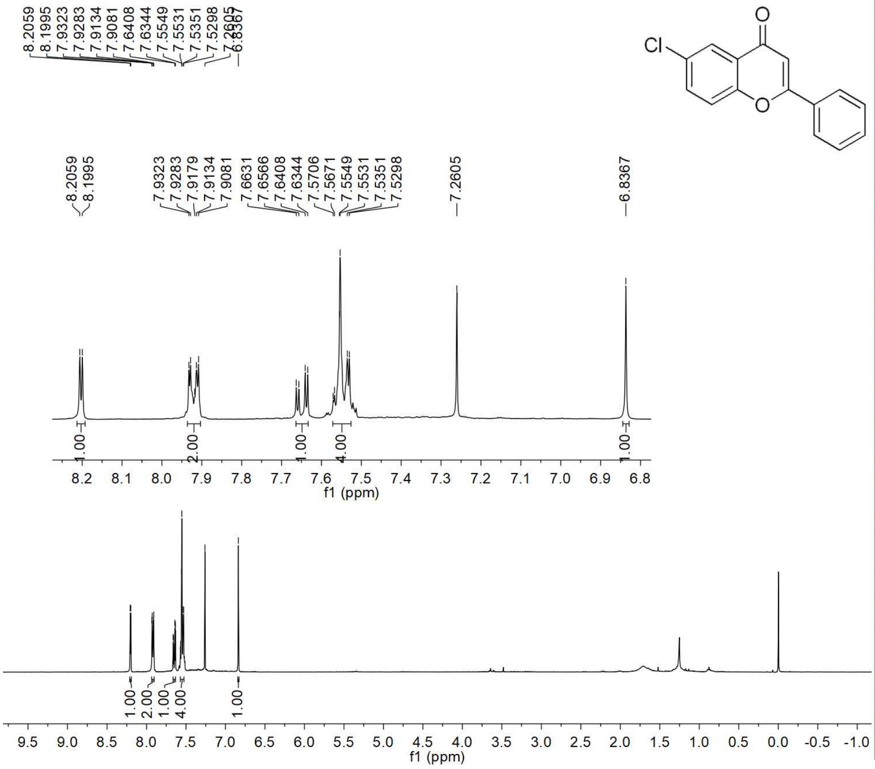

	Parameter	Value
1	Data File Name	E:/ hxw 400/ 2021-2-hxw-H/ 52/ fid
2	Title	2021-2-hxw-H. 52.fid
3	Comment	
4	Origin	Bruker BioSpin GmbH
5	Owner	nmrsu
6	Site	
7	Instrument	Avance
8	Author	
9	Solvent	CDCl3
10	Temperature	297.7
11	Pulse Sequence	zg30
12	Experiment	1D
13	Probe	Z116098_0916 (PA BBO 400S1 BBF-H-D-05 Z SP)
14	Number of Scans	4
15	Receiver Gain	101.0
16	Relaxation Delay	1.0000
17	Pulse Width	10.0000
18	Presaturation Frequency	
19	Acquisition Time	3.9977
20	Acquisition Date	2021-09-22T18:45:47
21	Modification Date	2021-09-22T18:45:32
22	Class	
23	Spectrometer Frequency	400.13
24	Spectral Width	8196.7
25	Lowest Frequency	-1637.6
26	Nucleus	1H
27	Acquired Size	32768
28	Spectral Size	65536

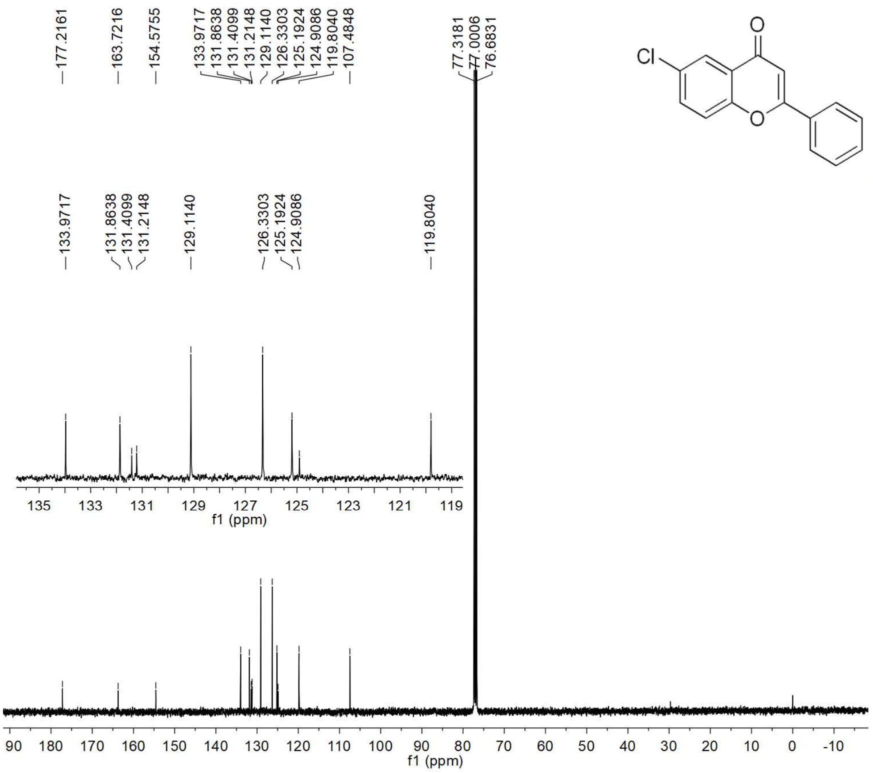

	Parameter	Value
1	Data File Name	E:/ hxw 400/ 2021-2-hxw-C/ 12/ fid
2	Title	2021-2-hxw-C. 12.fid
3	Comment	
4	Origin	Bruker BioSpin GmbH
5	Owner	nmrsu
6	Site	
7	Instrument	Avance
8	Author	
9	Solvent	CDCl3
10	Temperature	298.2
11	Pulse Sequence	zgpg30
12	Experiment	1D
13	Probe	Z116098_0916 (PA BBO 400S1 BBF-H-D-05 Z SP)
14	Number of Scans	500
15	Receiver Gain	101.0
16	Relaxation Delay	2.0000
17	Pulse Width	10.0000
18	Presaturation Frequency	
19	Acquisition Time	1.3763
20	Acquisition Date	2021-09-29T23:50:10
21	Modification Date	2021-09-29T23:49:14
22	Class	
23	Spectrometer Frequency	100.62
24	Spectral Width	23809.5
25	Lowest Frequency	-1845.9
26	Nucleus	13C
27	Acquired Size	32768
28	Spectral Size	65536

6-Bromo-2-phenyl-4*H*-chromen-4-one (2fa)

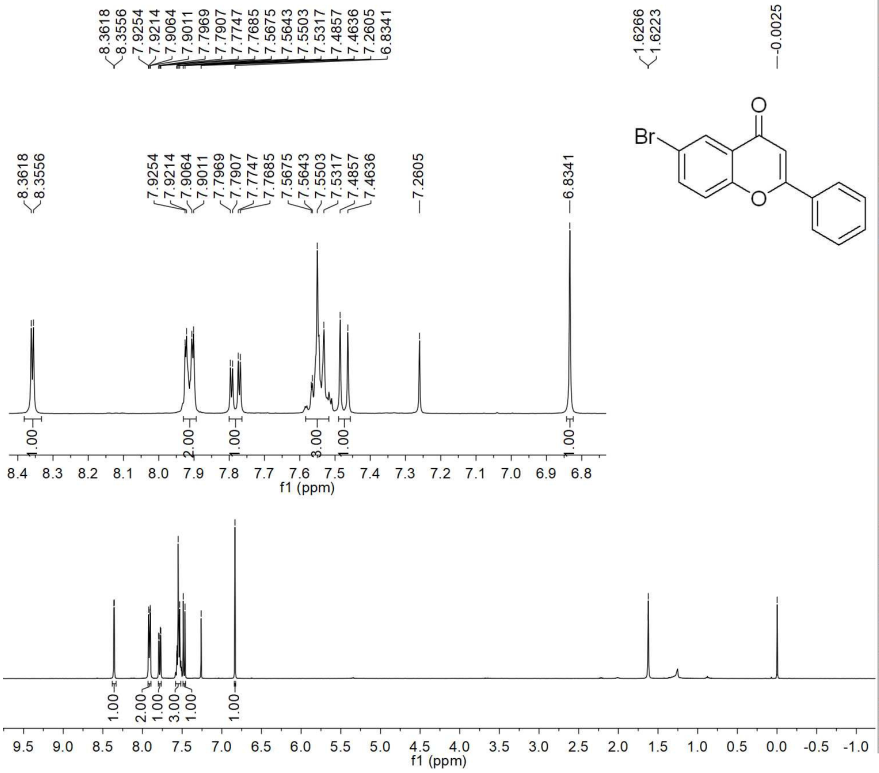

	Parameter	Value
1	Data File Name	E:/ hxw 400/ 2021-2-hxw-H/ 101/ fid
2	Title	2021-2-hxw-H. 101.fid
3	Comment	
4	Origin	Bruker BioSpin GmbH
5	Owner	nmrsu
6	Site	
7	Instrument	Avance
8	Author	
9	Solvent	CDCl3
10	Temperature	297.8
11	Pulse Sequence	zg30
12	Experiment	1D
13	Probe	Z116098_0916 (PA BBO 400S1 BBF-H-D-05 Z SP)
14	Number of Scans	4
15	Receiver Gain	101.0
16	Relaxation Delay	1.0000
17	Pulse Width	10.0000
18	Presaturation Frequency	
19	Acquisition Time	3.9977
20	Acquisition Date	2021-10-23T10:54:40
21	Modification Date	2021-10-23T10:53:58
22	Class	
23	Spectrometer Frequency	400.13
24	Spectral Width	8196.7
25	Lowest Frequency	-1637.6
26	Nucleus	1H
27	Acquired Size	32768
28	Spectral Size	65536

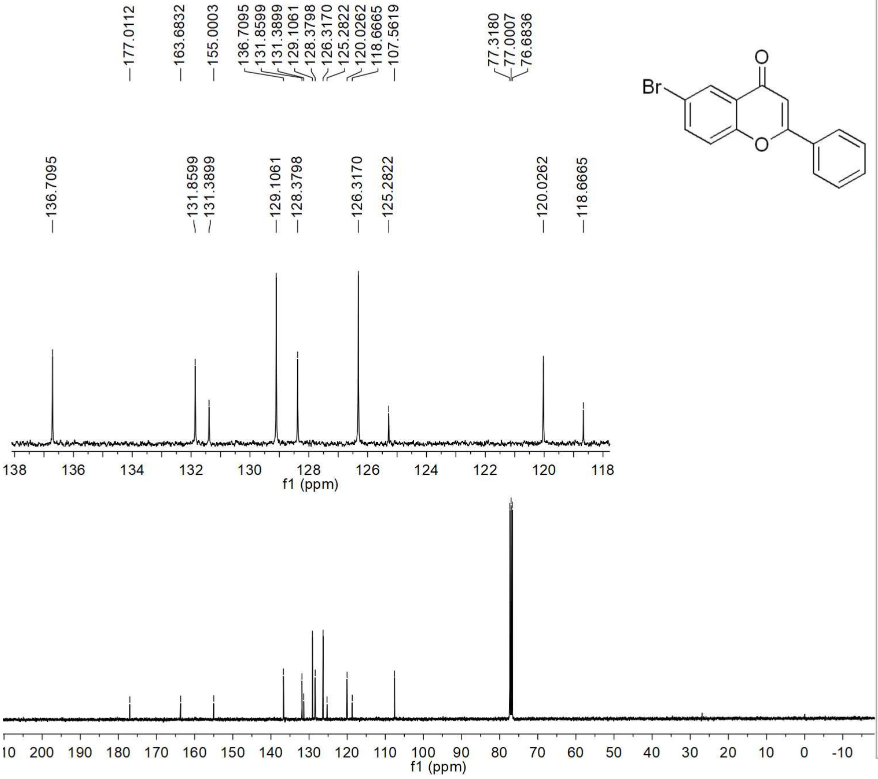

	Parameter	Value
1	Data File Name	E:/ hxw 400/ 2021-2-hxw-C/ 36/ fid
2	Title	2021-2-hxw-C. 36.fid
3	Comment	
4	Origin	Bruker BioSpin GmbH
5	Owner	nmrsu
6	Site	
7	Instrument	Avance
8	Author	
9	Solvent	CDCl3
10	Temperature	298.7
11	Pulse Sequence	zgpg30
12	Experiment	1D
13	Probe	Z116098_0916 (PA BBO 400S1 BBF-H-D-05 Z SP)
14	Number of Scans	400
15	Receiver Gain	101.0
16	Relaxation Delay	2.0000
17	Pulse Width	10.0000
18	Presaturation Frequency	
19	Acquisition Time	1.3763
20	Acquisition Date	2021-10-27T03:23:33
21	Modification Date	2021-10-27T03:22:28
22	Class	
23	Spectrometer Frequency	100.62
24	Spectral Width	23809.5
25	Lowest Frequency	-1846.4
26	Nucleus	13C
27	Acquired Size	32768
28	Spectral Size	65536

6,8-Dichloro-2-phenyl-4*H*-chromen-4-one (2ga)

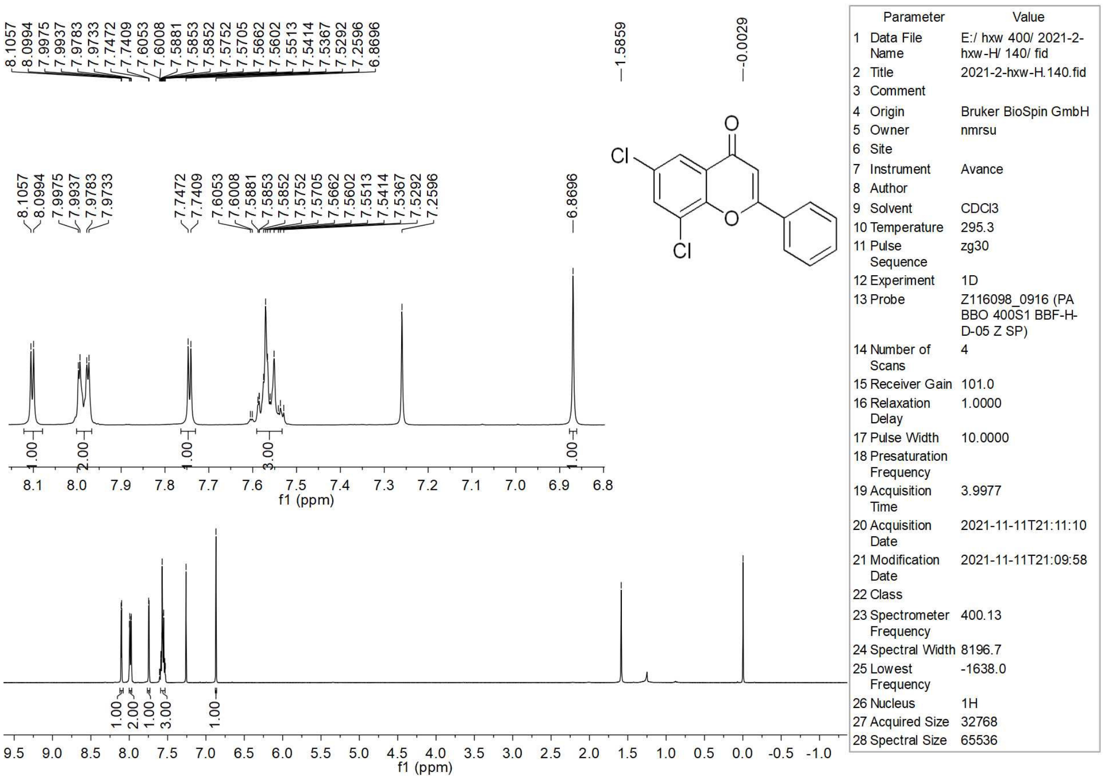

Parameter	Value
1 Data File Name	E:/ hxw 400/ 2021-2-hxw-H/ 140/ fid
2 Title	2021-2-hxw-H.140.fid
3 Comment	
4 Origin	Bruker BioSpin GmbH
5 Owner	nmrsu
6 Site	
7 Instrument	Avance
8 Author	
9 Solvent	CDCl3
10 Temperature	295.3
11 Pulse Sequence	zg30
12 Experiment	1D
13 Probe	Z116098_0916 (PA BBO 400S1 BBF-H-D-05 Z SP)
14 Number of Scans	4
15 Receiver Gain	101.0
16 Relaxation Delay	1.0000
17 Pulse Width	10.0000
18 Presaturation Frequency	
19 Acquisition Time	3.9977
20 Acquisition Date	2021-11-11T21:11:10
21 Modification Date	2021-11-11T21:09:58
22 Class	
23 Spectrometer Frequency	400.13
24 Spectral Width	8196.7
25 Lowest Frequency	-1638.0
26 Nucleus	1H
27 Acquired Size	32768
28 Spectral Size	65536

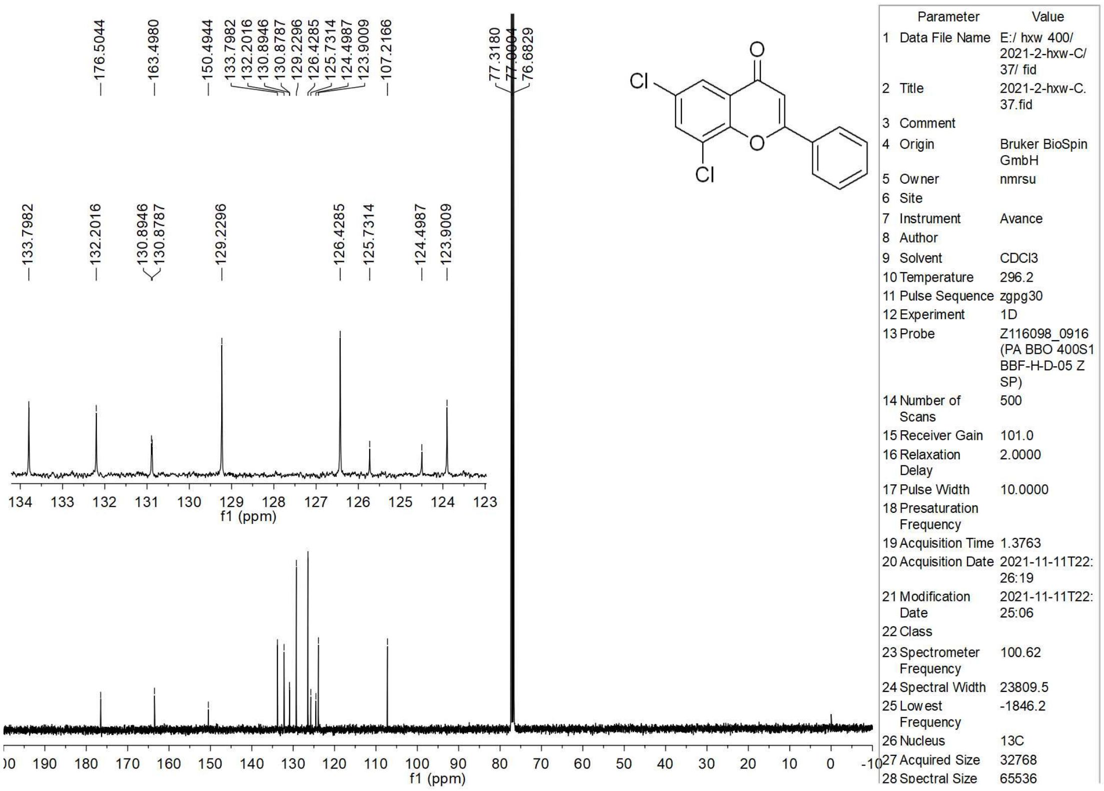

Parameter	Value
1 Data File Name	E:/ hxw 400/ 2021-2-hxw-C/ 37/ fid
2 Title	2021-2-hxw-C.37.fid
3 Comment	
4 Origin	Bruker BioSpin GmbH
5 Owner	nmrsu
6 Site	
7 Instrument	Avance
8 Author	
9 Solvent	CDCl3
10 Temperature	296.2
11 Pulse Sequence	zgpg30
12 Experiment	1D
13 Probe	Z116098_0916 (PA BBO 400S1 BBF-H-D-05 Z SP)
14 Number of Scans	500
15 Receiver Gain	101.0
16 Relaxation Delay	2.0000
17 Pulse Width	10.0000
18 Presaturation Frequency	
19 Acquisition Time	1.3763
20 Acquisition Date	2021-11-11T22:26:19
21 Modification Date	2021-11-11T22:25:06
22 Class	
23 Spectrometer Frequency	100.62
24 Spectral Width	23809.5
25 Lowest Frequency	-1846.2
26 Nucleus	13C
27 Acquired Size	32768
28 Spectral Size	65536

6,8-Dibromo-2-phenyl-4*H*-chromen-4-one (2ha)

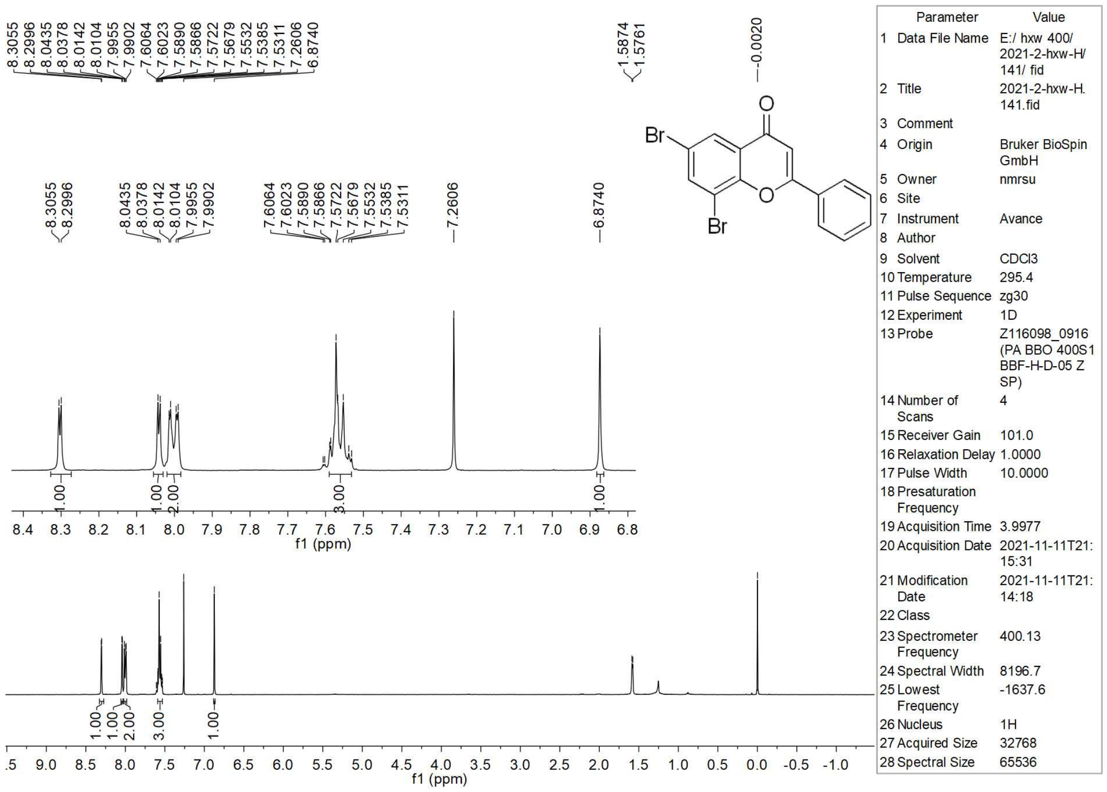

	Parameter	Value
1	Data File Name	E:/ hxw 400/ 2021-2-hxw-H/ 141/ fid
2	Title	2021-2-hxw-H. 141.fid
3	Comment	
4	Origin	Bruker BioSpin GmbH
5	Owner	nmrsu
6	Site	
7	Instrument	Avance
8	Author	
9	Solvent	CDCl3
10	Temperature	295.4
11	Pulse Sequence	zg30
12	Experiment	1D
13	Probe	Z116098_0916 (PA BBO 400S1 BBF-H-D-05 Z SP)
14	Number of Scans	4
15	Receiver Gain	101.0
16	Relaxation Delay	1.0000
17	Pulse Width	10.0000
18	Presaturation Frequency	
19	Acquisition Time	3.9977
20	Acquisition Date	2021-11-11T21:15:31
21	Modification Date	2021-11-11T21:14:18
22	Class	
23	Spectrometer Frequency	400.13
24	Spectral Width	8196.7
25	Lowest Frequency	-1637.6
26	Nucleus	1H
27	Acquired Size	32768
28	Spectral Size	65536

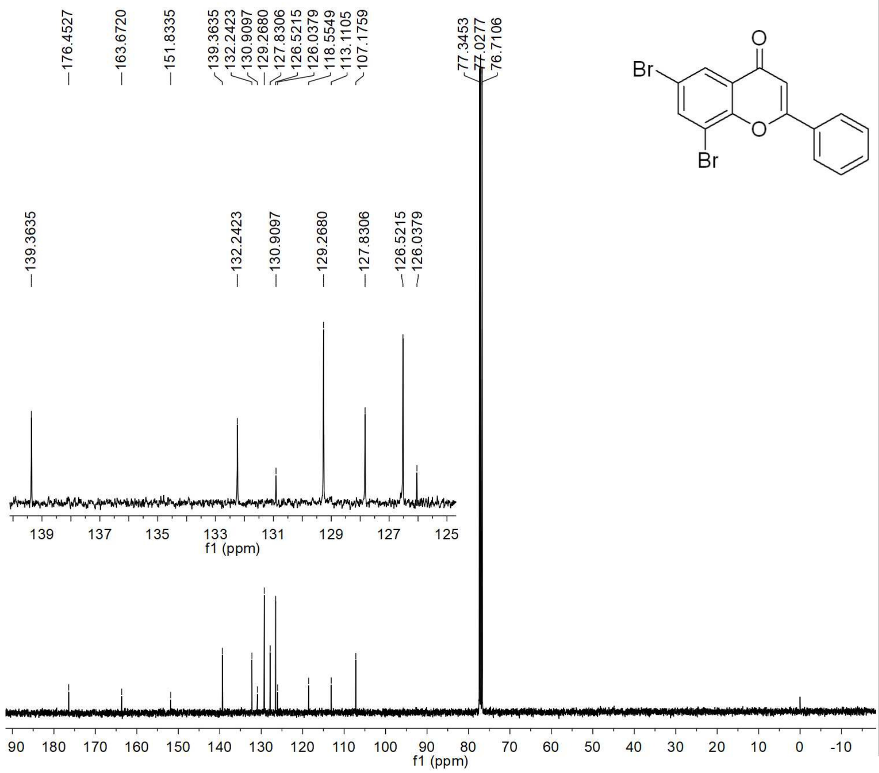

	Parameter	Value
1	Data File Name	E:/ hxw 400/ 2021-2-hxw-C/ 38/ fid
2	Title	2021-2-hxw-C. 38.fid
3	Comment	
4	Origin	Bruker BioSpin GmbH
5	Owner	nmrsu
6	Site	
7	Instrument	Avance
8	Author	
9	Solvent	CDCl3
10	Temperature	296.2
11	Pulse Sequence	zgpg30
12	Experiment	1D
13	Probe	Z116098_0916 (PA BBO 400S1 BBF-H-D-05 Z SP)
14	Number of Scans	500
15	Receiver Gain	101.0
16	Relaxation Delay	2.0000
17	Pulse Width	10.0000
18	Presaturation Frequency	
19	Acquisition Time	1.3763
20	Acquisition Date	2021-11-11T22:59:00
21	Modification Date	2021-11-11T22:57:46
22	Class	
23	Spectrometer Frequency	100.62
24	Spectral Width	23809.5
25	Lowest Frequency	-1843.5
26	Nucleus	13C
27	Acquired Size	32768
28	Spectral Size	65536

6,8-Di-*tert*-butyl-2-phenyl-4*H*-chromen-4-one(2ia)

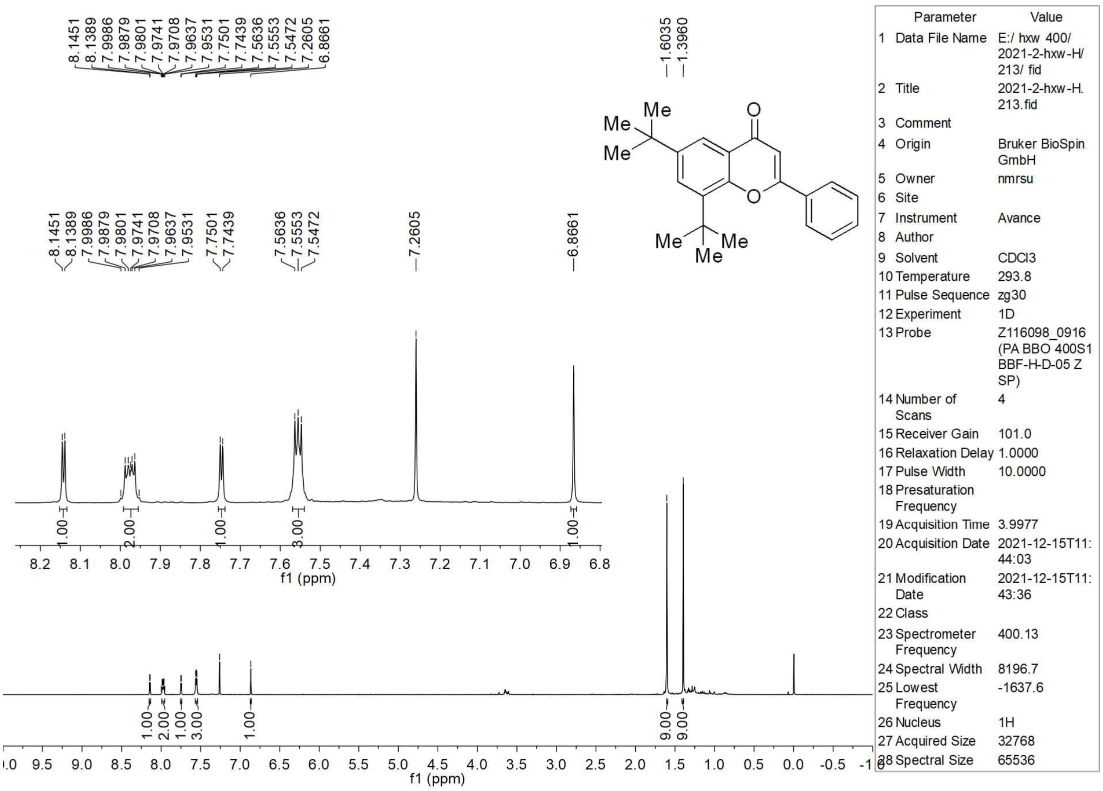

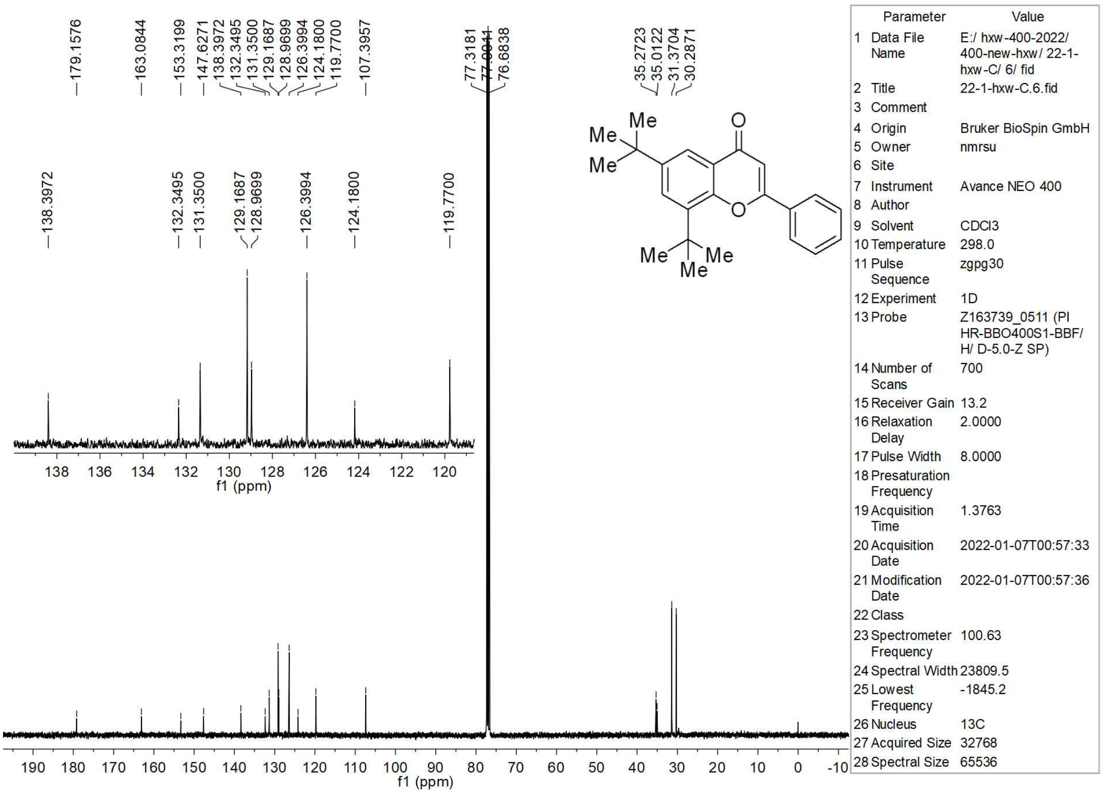

5-Chloro-2-phenyl-4*H*-chromen-4-one (2ja)

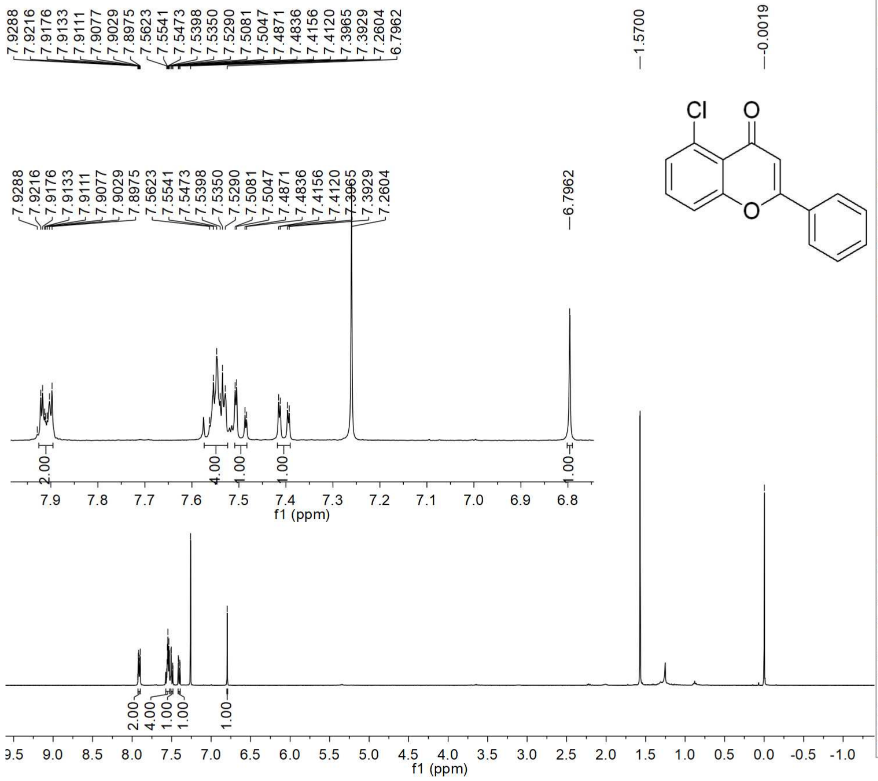

	Parameter	Value
1	Data File Name	E:/ hxw 400/ 2021-2-hxw-H/ 207/ fid
2	Title	2021-2-hxw-H. 207.fid
3	Comment	
4	Origin	Bruker BioSpin GmbH
5	Owner	nmrsu
6	Site	
7	Instrument	Avance
8	Author	
9	Solvent	CDCl3
10	Temperature	295.7
11	Pulse Sequence	zg30
12	Experiment	1D
13	Probe	Z116098_0916 (PA BBO 400S1 BBF-H-D-05 Z SP)
14	Number of Scans	4
15	Receiver Gain	101.0
16	Relaxation Delay	1.0000
17	Pulse Width	10.0000
18	Presaturation Frequency	
19	Acquisition Time	3.9977
20	Acquisition Date	2021-12-11T18:16:20
21	Modification Date	2021-12-11T18:16:14
22	Class	
23	Spectrometer Frequency	400.13
24	Spectral Width	8196.7
25	Lowest Frequency	-1637.6
26	Nucleus	1H
27	Acquired Size	32768
28	Spectral Size	65536

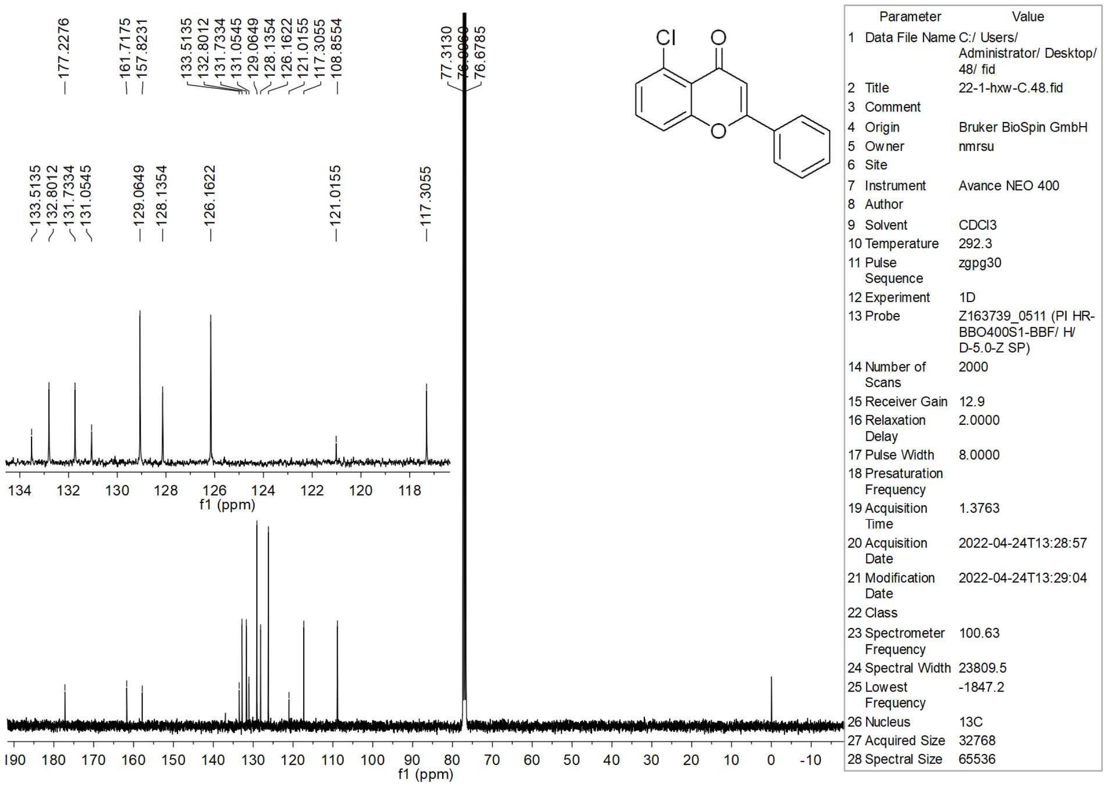

	Parameter	Value
1	Data File Name	C:/ Users/ Administrator/ Desktop/ 48/ fid
2	Title	22-1-hxw-C.48.fid
3	Comment	
4	Origin	Bruker BioSpin GmbH
5	Owner	nmrsu
6	Site	
7	Instrument	Avance NEO 400
8	Author	
9	Solvent	CDCl3
10	Temperature	292.3
11	Pulse Sequence	zgpg30
12	Experiment	1D
13	Probe	Z163739_0511 (PI HR-BBO400S1-BBF/ H/ D-5.0-Z SP)
14	Number of Scans	2000
15	Receiver Gain	12.9
16	Relaxation Delay	2.0000
17	Pulse Width	8.0000
18	Presaturation Frequency	
19	Acquisition Time	1.3763
20	Acquisition Date	2022-04-24T13:28:57
21	Modification Date	2022-04-24T13:29:04
22	Class	
23	Spectrometer Frequency	100.63
24	Spectral Width	23809.5
25	Lowest Frequency	-1847.2
26	Nucleus	13C
27	Acquired Size	32768
28	Spectral Size	65536

7-Chloro-2-phenyl-4*H*-chromen-4-one (2ka)

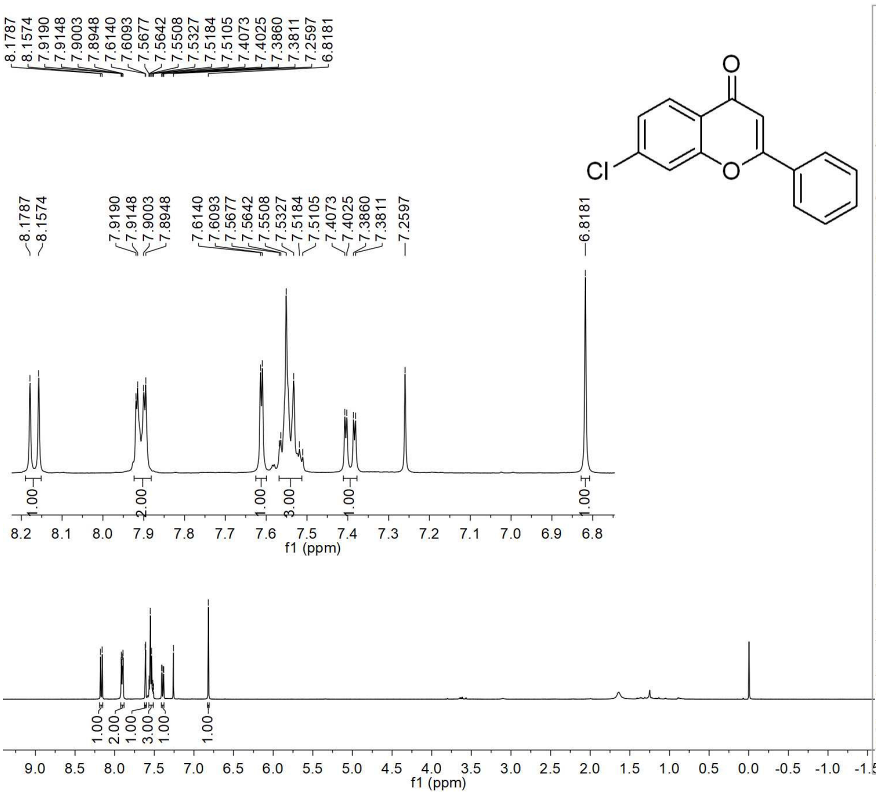

	Parameter	Value
1	Data File Name	E:/ hxw 400/ 2021-2-hxw-H/ 208/ fid
2	Title	2021-2-hxw-H. 208.fid
3	Comment	
4	Origin	Bruker BioSpin GmbH
5	Owner	nmrsu
6	Site	
7	Instrument	Avance
8	Author	
9	Solvent	CDCl3
10	Temperature	295.7
11	Pulse Sequence	zg30
12	Experiment	1D
13	Probe	Z116098_0916 (PA BBO 400S1 BBF-H-D-05 Z SP)
14	Number of Scans	4
15	Receiver Gain	101.0
16	Relaxation Delay	1.0000
17	Pulse Width	10.0000
18	Presaturation Frequency	
19	Acquisition Time	3.9977
20	Acquisition Date	2021-12-11T18:21:00
21	Modification Date	2021-12-11T18:20:54
22	Class	
23	Spectrometer Frequency	400.13
24	Spectral Width	8196.7
25	Lowest Frequency	-1638.0
26	Nucleus	1H
27	Acquired Size	32768
28	Spectral Size	65536

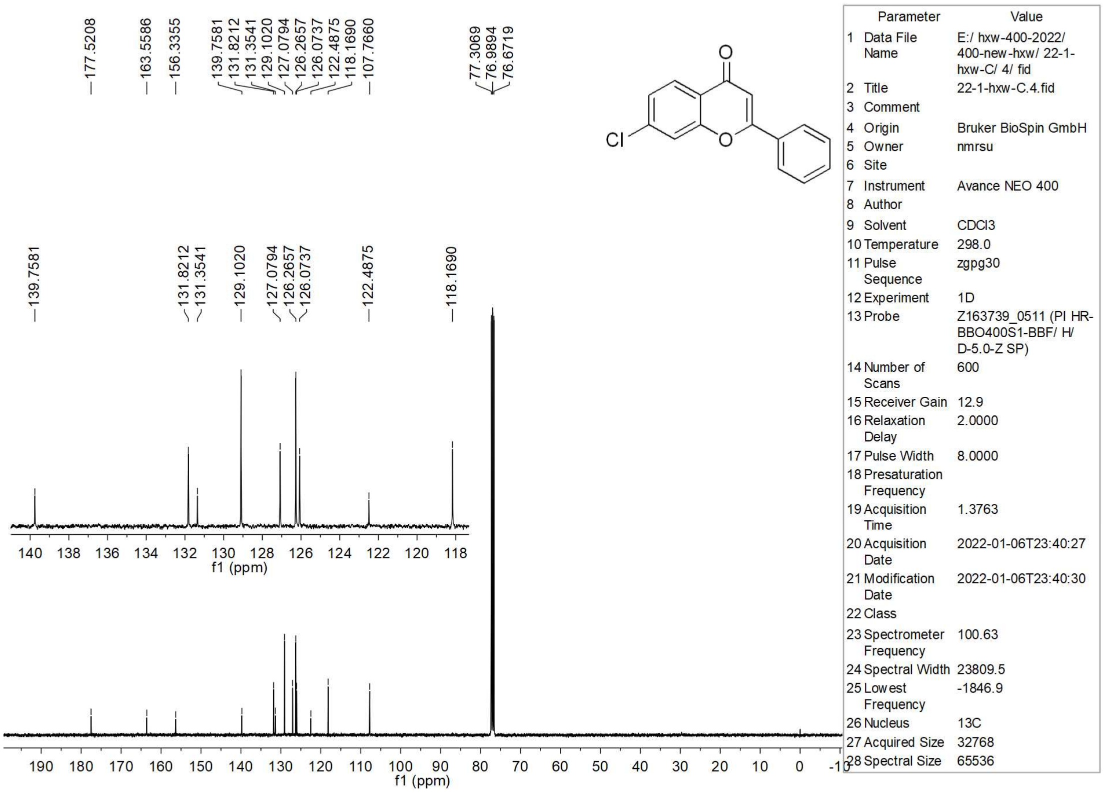

	Parameter	Value
1	Data File Name	E:/ hxw-400-2022/ 400-new-hxw/ 22-1-hxw-C/ 4/ fid
2	Title	22-1-hxw-C.4.fid
3	Comment	
4	Origin	Bruker BioSpin GmbH
5	Owner	nmrsu
6	Site	
7	Instrument	Avance NEO 400
8	Author	
9	Solvent	CDCl3
10	Temperature	298.0
11	Pulse Sequence	zgpg30
12	Experiment	1D
13	Probe	Z163739_0511 (PI HR-BBO400S1-BBF/ H/ D-5.0-Z SP)
14	Number of Scans	600
15	Receiver Gain	12.9
16	Relaxation Delay	2.0000
17	Pulse Width	8.0000
18	Presaturation Frequency	
19	Acquisition Time	1.3763
20	Acquisition Date	2022-01-06T23:40:27
21	Modification Date	2022-01-06T23:40:30
22	Class	
23	Spectrometer Frequency	100.63
24	Spectral Width	23809.5
25	Lowest Frequency	-1846.9
26	Nucleus	13C
27	Acquired Size	32768
28	Spectral Size	65536

8-(*tert*-Butyl)-2-phenyl-4*H*-chromen-4-one(2la)

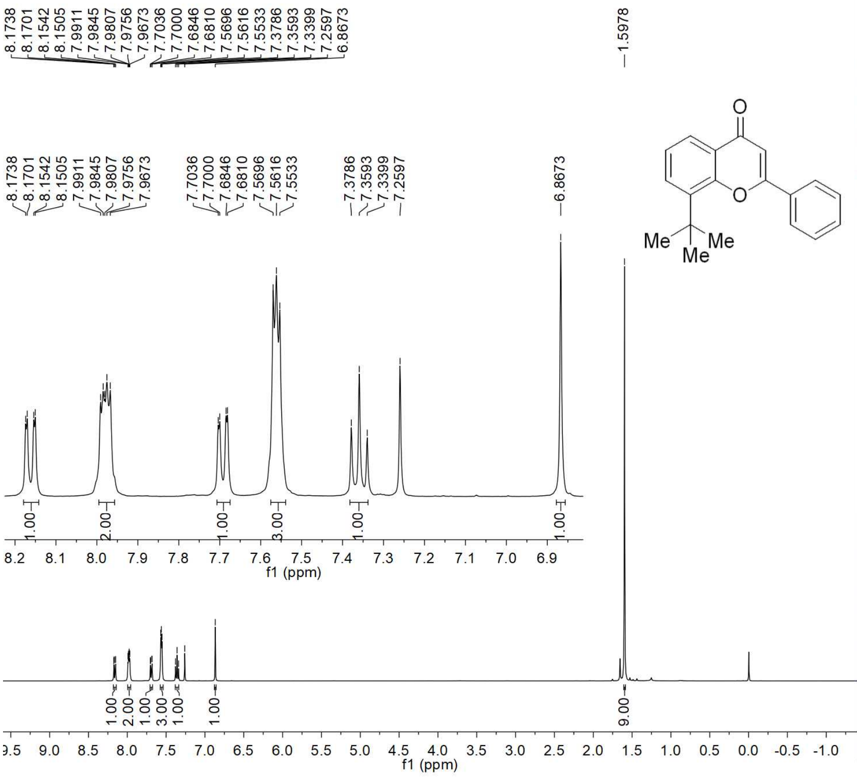

Parameter	Value
1 Data File Name	E:/ hxw 400/ 2021-2-hxw-H/ 205/ fid
2 Title	2021-2-hxw-H.205.fid
3 Comment	
4 Origin	Bruker BioSpin GmbH
5 Owner	nmrsu
6 Site	
7 Instrument	Avance
8 Author	
9 Solvent	CDCl3
10 Temperature	295.8
11 Pulse Sequence	zg30
12 Experiment	1D
13 Probe	Z116098_0916 (PA BBO 400S1 BBF-H-D-05 Z SP)
14 Number of Scans	4
15 Receiver Gain	101.0
16 Relaxation Delay	1.0000
17 Pulse Width	10.0000
18 Presaturation Frequency	
19 Acquisition Time	3.9977
20 Acquisition Date	2021-12-09T19:03:53
21 Modification Date	2021-12-09T19:02:28
22 Class	
23 Spectrometer Frequency	400.13
24 Spectral Width	8196.7
25 Lowest Frequency	-1638.0
26 Nucleus	1H
27 Acquired Size	32768
28 Spectral Size	65536

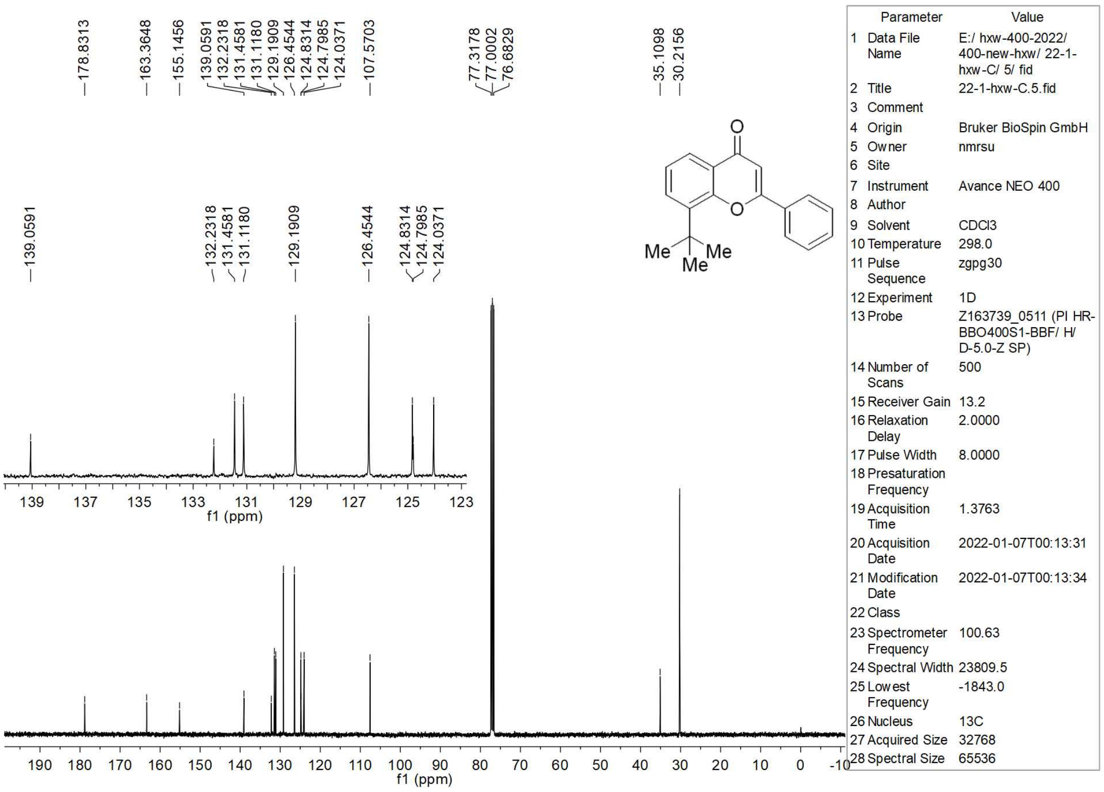

Parameter	Value
1 Data File Name	E:/ hxw-400-2022/ 400-new-hxw/ 22-1-hxw-C/ 5/ fid
2 Title	22-1-hxw-C.5.fid
3 Comment	
4 Origin	Bruker BioSpin GmbH
5 Owner	nmrsu
6 Site	
7 Instrument	Avance NEO 400
8 Author	
9 Solvent	CDCl3
10 Temperature	298.0
11 Pulse Sequence	zgpg30
12 Experiment	1D
13 Probe	Z163739_0511 (PI HR-BBO400S1-BBF/ H/ D-5.0-Z SP)
14 Number of Scans	500
15 Receiver Gain	13.2
16 Relaxation Delay	2.0000
17 Pulse Width	8.0000
18 Presaturation Frequency	
19 Acquisition Time	1.3763
20 Acquisition Date	2022-01-07T00:13:31
21 Modification Date	2022-01-07T00:13:34
22 Class	
23 Spectrometer Frequency	100.63
24 Spectral Width	23809.5
25 Lowest Frequency	-1843.0
26 Nucleus	13C
27 Acquired Size	32768
28 Spectral Size	65536

2-(*p*-Tolyl)-4*H*-chromen-4-one (2ab)

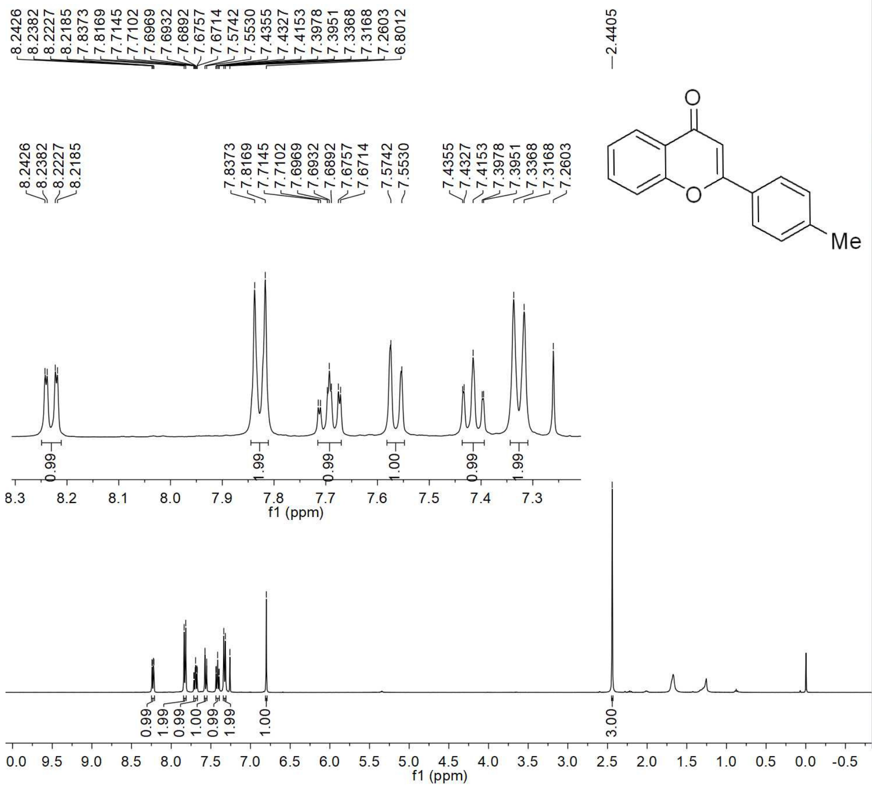

	Parameter	Value
1	Data File Name	E:/ hxw 400/ 2021-1-hxw-H/ 98/ fid
2	Title	2021-1-hxw-H. 98.fid
3	Comment	
4	Origin	Bruker BioSpin GmbH
5	Owner	nmrsu
6	Site	
7	Instrument	Avance
8	Author	
9	Solvent	CDCl3
10	Temperature	298.3
11	Pulse Sequence	zg30
12	Experiment	1D
13	Probe	Z163739_0032 (PI HR-400-S1-BBF/ H/ D-5.0-Z SP)
14	Number of Scans	4
15	Receiver Gain	101.0
16	Relaxation Delay	1.0000
17	Pulse Width	10.0000
18	Presaturation Frequency	
19	Acquisition Time	3.9977
20	Acquisition Date	2021-06-30T12:11:44
21	Modification Date	2021-06-30T12:11:18
22	Class	
23	Spectrometer Frequency	400.13
24	Spectral Width	8196.7
25	Lowest Frequency	-1637.2
26	Nucleus	1H
27	Acquired Size	32768
28	Spectral Size	65536

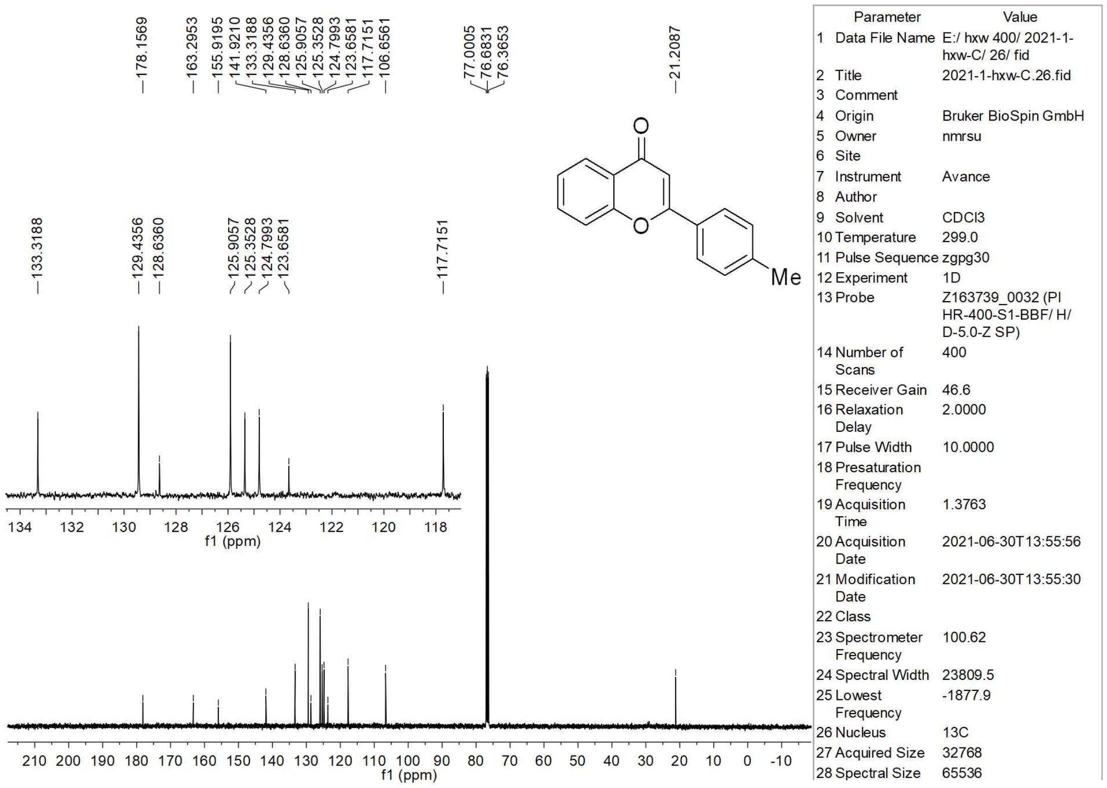

	Parameter	Value
1	Data File Name	E:/ hxw 400/ 2021-1-hxw-C/ 26/ fid
2	Title	2021-1-hxw-C.26.fid
3	Comment	
4	Origin	Bruker BioSpin GmbH
5	Owner	nmrsu
6	Site	
7	Instrument	Avance
8	Author	
9	Solvent	CDCl3
10	Temperature	299.0
11	Pulse Sequence	zgpg30
12	Experiment	1D
13	Probe	Z163739_0032 (PI HR-400-S1-BBF/ H/ D-5.0-Z SP)
14	Number of Scans	400
15	Receiver Gain	46.6
16	Relaxation Delay	2.0000
17	Pulse Width	10.0000
18	Presaturation Frequency	
19	Acquisition Time	1.3763
20	Acquisition Date	2021-06-30T13:55:56
21	Modification Date	2021-06-30T13:55:30
22	Class	
23	Spectrometer Frequency	100.62
24	Spectral Width	23809.5
25	Lowest Frequency	-1877.9
26	Nucleus	13C
27	Acquired Size	32768
28	Spectral Size	65536

2-(4-Methoxyphenyl)-4*H*-chromen-4-one (2ac)

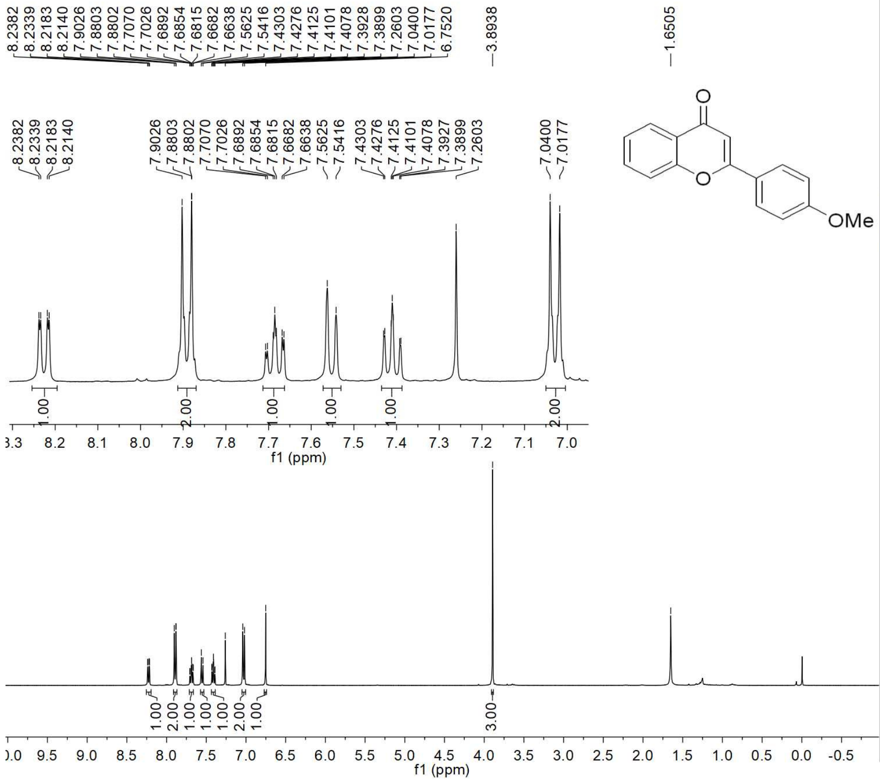

	Parameter	Value
1	Data File Name	E:/ hxw 400/ 2021-1-hxw-H/ 119/ fid
2	Title	2021-1-hxw-H.119.fid
3	Comment	
4	Origin	Bruker BioSpin GmbH
5	Owner	nmrsu
6	Site	
7	Instrument	Avance
8	Author	
9	Solvent	CDCl3
10	Temperature	298.3
11	Pulse Sequence	zg30
12	Experiment	1D
13	Probe	Z163739_0032 (PI HR-400-S1-BBF/ H/ D-5.0-Z SP)
14	Number of Scans	4
15	Receiver Gain	101.0
16	Relaxation Delay	1.0000
17	Pulse Width	10.0000
18	Presaturation Frequency	
19	Acquisition Time	3.9977
20	Acquisition Date	2021-07-09T13:55:55
21	Modification Date	2021-07-09T13:55:24
22	Class	
23	Spectrometer Frequency	400.13
24	Spectral Width	8196.7
25	Lowest Frequency	-1637.2
26	Nucleus	1H
27	Acquired Size	32768
28	Spectral Size	65536

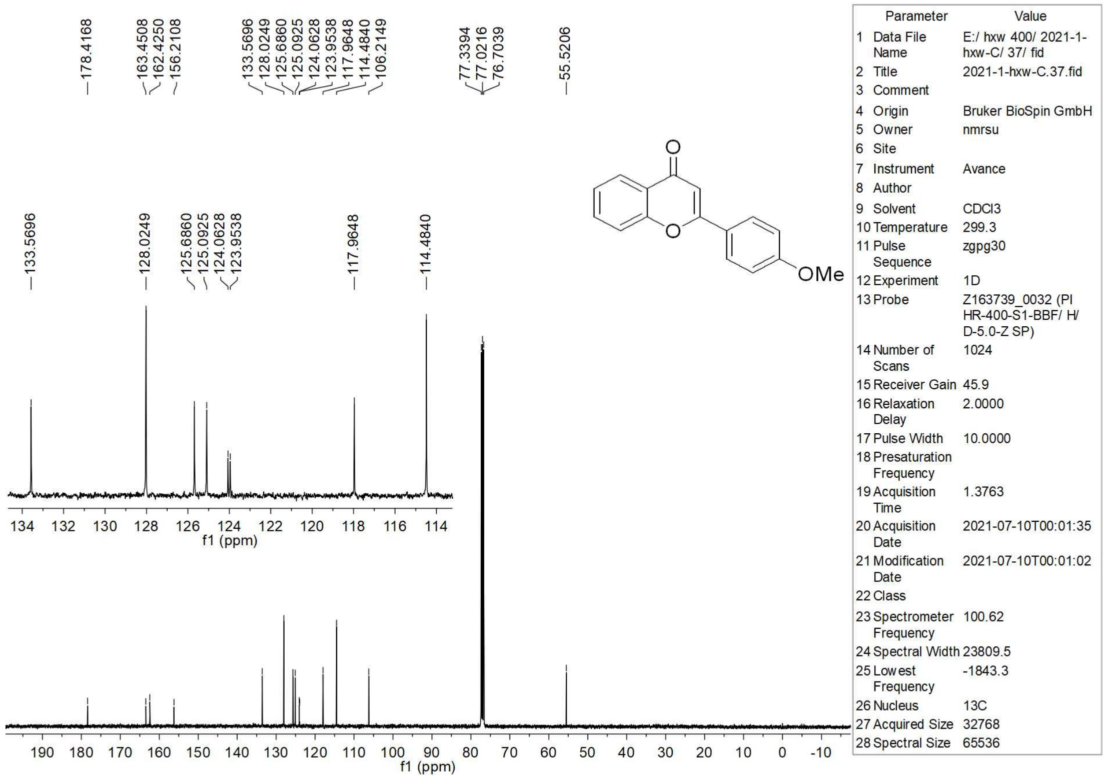

	Parameter	Value
1	Data File Name	E:/ hxw 400/ 2021-1-hxw-C/ 37/ fid
2	Title	2021-1-hxw-C.37.fid
3	Comment	
4	Origin	Bruker BioSpin GmbH
5	Owner	nmrsu
6	Site	
7	Instrument	Avance
8	Author	
9	Solvent	CDCl3
10	Temperature	299.3
11	Pulse Sequence	zgpg30
12	Experiment	1D
13	Probe	Z163739_0032 (PI HR-400-S1-BBF/ H/ D-5.0-Z SP)
14	Number of Scans	1024
15	Receiver Gain	45.9
16	Relaxation Delay	2.0000
17	Pulse Width	10.0000
18	Presaturation Frequency	
19	Acquisition Time	1.3763
20	Acquisition Date	2021-07-10T00:01:35
21	Modification Date	2021-07-10T00:01:02
22	Class	
23	Spectrometer Frequency	100.62
24	Spectral Width	23809.5
25	Lowest Frequency	-1843.3
26	Nucleus	13C
27	Acquired Size	32768
28	Spectral Size	65536

2-(4-Fluorophenyl)-4*H*-chromen-4-one (2ad)

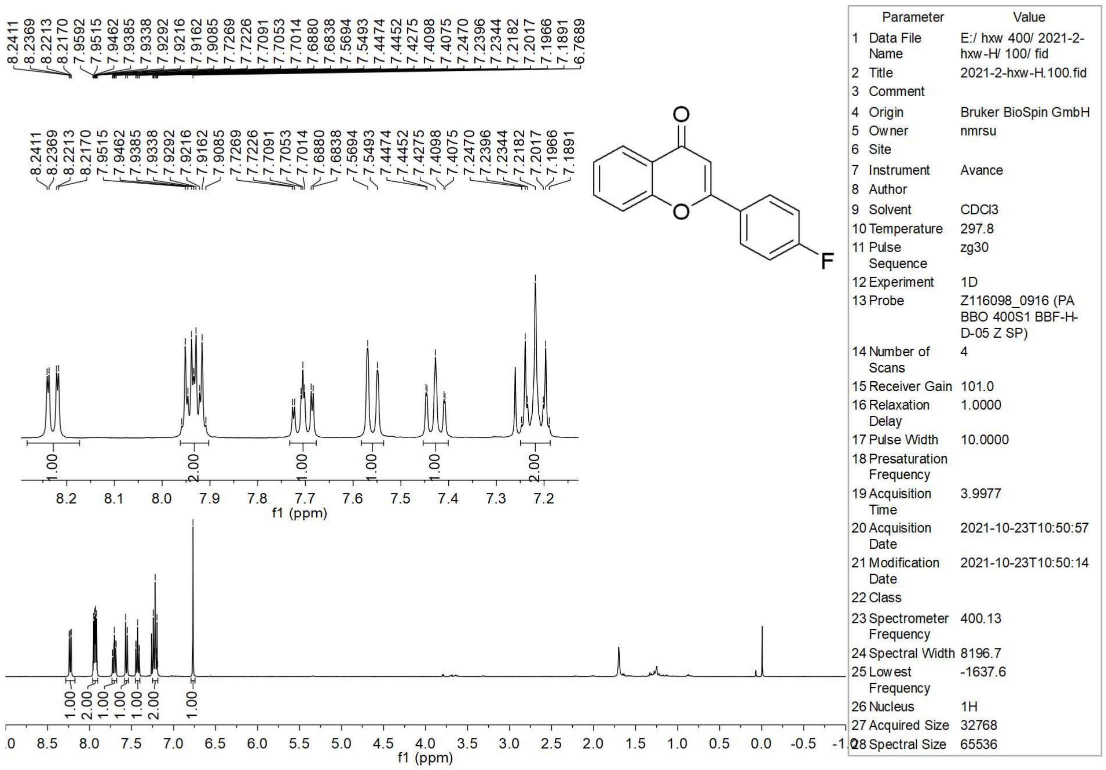

Parameter		Value
1	Data File Name	E:/ hxw 400/ 2021-2-hxw-H/ 100/ fid
2	Title	2021-2-hxw-H.100.fid
3	Comment	
4	Origin	Bruker BioSpin GmbH
5	Owner	nmrsu
6	Site	
7	Instrument	Avance
8	Author	
9	Solvent	CDCl3
10	Temperature	297.8
11	Pulse Sequence	zg30
12	Experiment	1D
13	Probe	Z116098_0916 (PA BBO 400S1 BBF-H-D-05 Z SP)
14	Number of Scans	4
15	Receiver Gain	101.0
16	Relaxation Delay	1.0000
17	Pulse Width	10.0000
18	Presaturation Frequency	
19	Acquisition Time	3.9977
20	Acquisition Date	2021-10-23T10:50:57
21	Modification Date	2021-10-23T10:50:14
22	Class	
23	Spectrometer Frequency	400.13
24	Spectral Width	8196.7
25	Lowest Frequency	-1637.6
26	Nucleus	1H
27	Acquired Size	32768
28	Spectral Size	65536

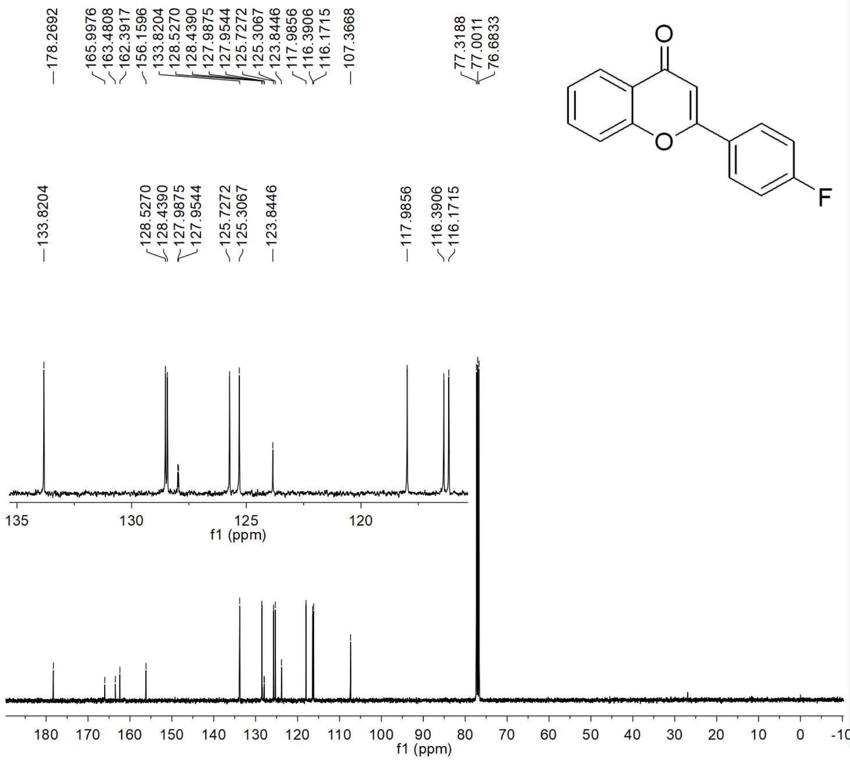

Parameter		Value
1	Data File Name	E:/ hxw 400/ 2021-2-hxw-C/ 33/ fid
2	Title	2021-2-hxw-C.33.fid
3	Comment	
4	Origin	Bruker BioSpin GmbH
5	Owner	nmrsu
6	Site	
7	Instrument	Avance
8	Author	
9	Solvent	CDCl3
10	Temperature	298.8
11	Pulse Sequence	zgpg30
12	Experiment	1D
13	Probe	Z116098_0916 (PA BBO 400S1 BBF-H-D-05 Z SP)
14	Number of Scans	400
15	Receiver Gain	101.0
16	Relaxation Delay	2.0000
17	Pulse Width	10.0000
18	Presaturation Frequency	
19	Acquisition Time	1.3763
20	Acquisition Date	2021-10-27T01:55:08
21	Modification Date	2021-10-27T01:54:04
22	Class	
23	Spectrometer Frequency	100.62
24	Spectral Width	23809.5
25	Lowest Frequency	-1846.8
26	Nucleus	13C
27	Acquired Size	32768
28	Spectral Size	65536

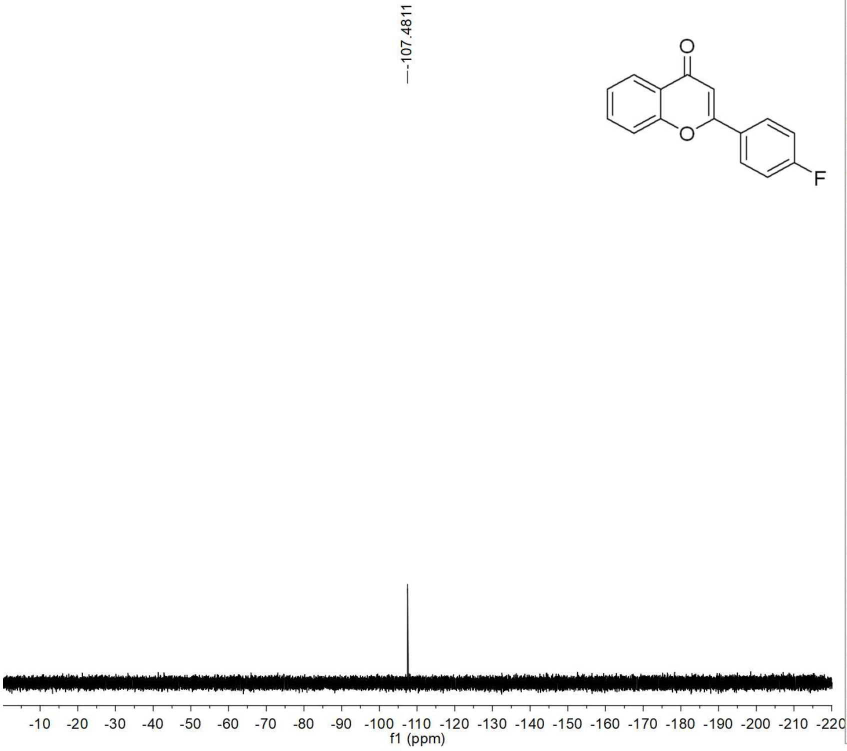

	Parameter	Value
1	Data File Name	E:/ hxw 400/ 2021-2-hxw-F/ 3/ fid
2	Title	2021-2-hxw-F.3.fid
3	Comment	
4	Origin	Bruker BioSpin GmbH
5	Owner	nmrsu
6	Site	
7	Instrument	Avance
8	Author	
9	Solvent	CDCl3
10	Temperature	298.4
11	Pulse Sequence	zgig
12	Experiment	1D
13	Probe	Z116098_0916 (PA BBO 400S1 BBF-H-D-05 Z SP)
14	Number of Scans	16
15	Receiver Gain	13.1
16	Relaxation Delay	1.0000
17	Pulse Width	19.7500
18	Presaturation Frequency	
19	Acquisition Time	0.7209
20	Acquisition Date	2021-10-27T01:30:39
21	Modification Date	2021-10-27T01:29:36
22	Class	
23	Spectrometer Frequency	376.46
24	Spectral Width	90909.1
25	Lowest Frequency	-83104.4
26	Nucleus	19F
27	Acquired Size	65536
28	Spectral Size	131072

2-(4-Chlorophenyl)-4*H*-chromen-4-one (2ae)

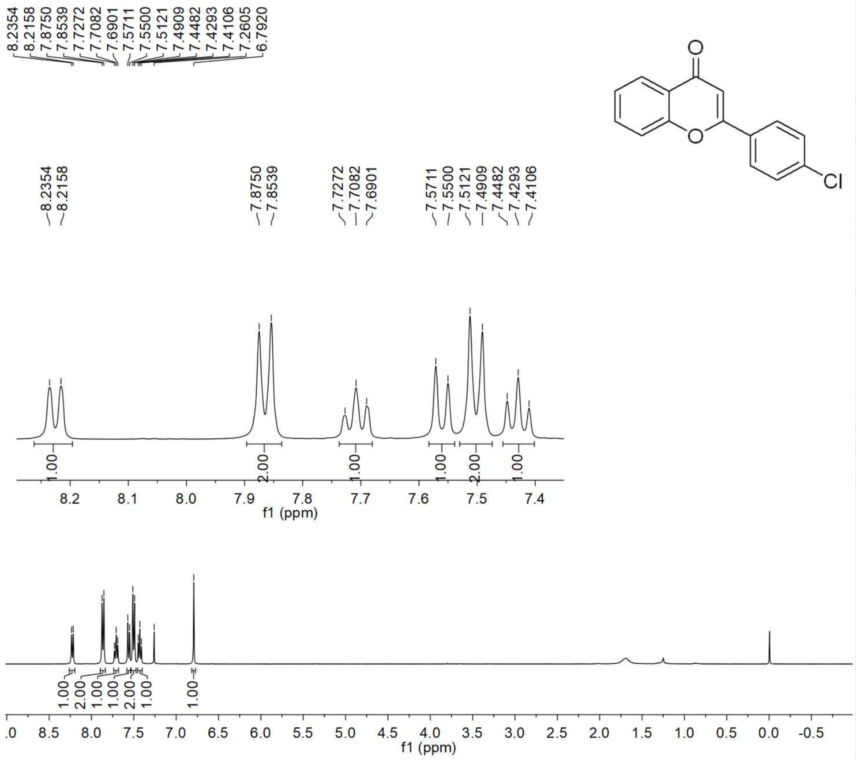

Parameter	Value
1 Data File Name	E:/ hxw 400/ 2021-2-hxw-H/ 69/ fid
2 Title	2021-2-hxw-H.69.fid
3 Comment	
4 Origin	Bruker BioSpin GmbH
5 Owner	nmrsu
6 Site	
7 Instrument	Avance
8 Author	
9 Solvent	CDCl3
10 Temperature	297.6
11 Pulse Sequence	zg30
12 Experiment	1D
13 Probe	Z116098_0916 (PA BBO 400S1 BBF-H-D-05 Z SP)
14 Number of Scans	4
15 Receiver Gain	101.0
16 Relaxation Delay	1.0000
17 Pulse Width	10.0000
18 Presaturation Frequency	
19 Acquisition Time	3.9977
20 Acquisition Date	2021-10-11T19:03:46
21 Modification Date	2021-10-11T19:01:42
22 Class	
23 Spectrometer Frequency	400.13
24 Spectral Width	8196.7
25 Lowest Frequency	-1637.6
26 Nucleus	1H
27 Acquired Size	32768
28 Spectral Size	65536

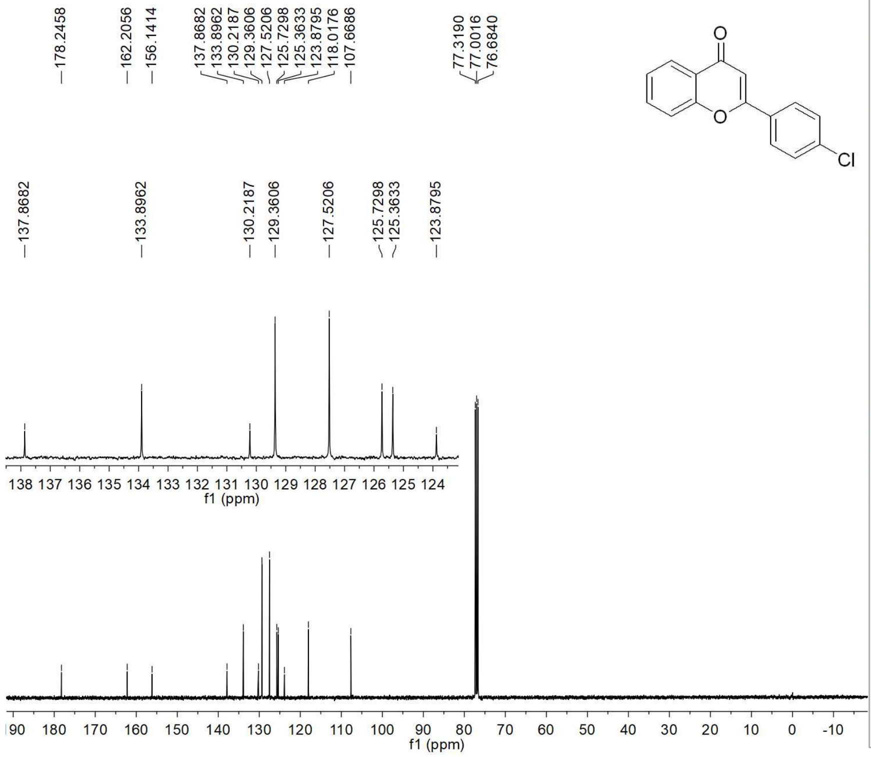

Parameter	Value
1 Data File Name	E:/ hxw 400/ 2021-2-hxw-C/ 19/ fid
2 Title	2021-2-hxw-C.19.fid
3 Comment	
4 Origin	Bruker BioSpin GmbH
5 Owner	nmrsu
6 Site	
7 Instrument	Avance
8 Author	
9 Solvent	CDCl3
10 Temperature	298.2
11 Pulse Sequence	zgpg30
12 Experiment	1D
13 Probe	Z116098_0916 (PA BBO 400S1 BBF-H-D-05 Z SP)
14 Number of Scans	400
15 Receiver Gain	101.0
16 Relaxation Delay	2.0000
17 Pulse Width	10.0000
18 Presaturation Frequency	
19 Acquisition Time	1.3763
20 Acquisition Date	2021-10-11T22:49:58
21 Modification Date	2021-10-11T22:47:54
22 Class	
23 Spectrometer Frequency	100.62
24 Spectral Width	23809.5
25 Lowest Frequency	-1847.1
26 Nucleus	13C
27 Acquired Size	32768
28 Spectral Size	65536

2-(4-Bromophenyl)-4*H*-chromen-4-one (2af)

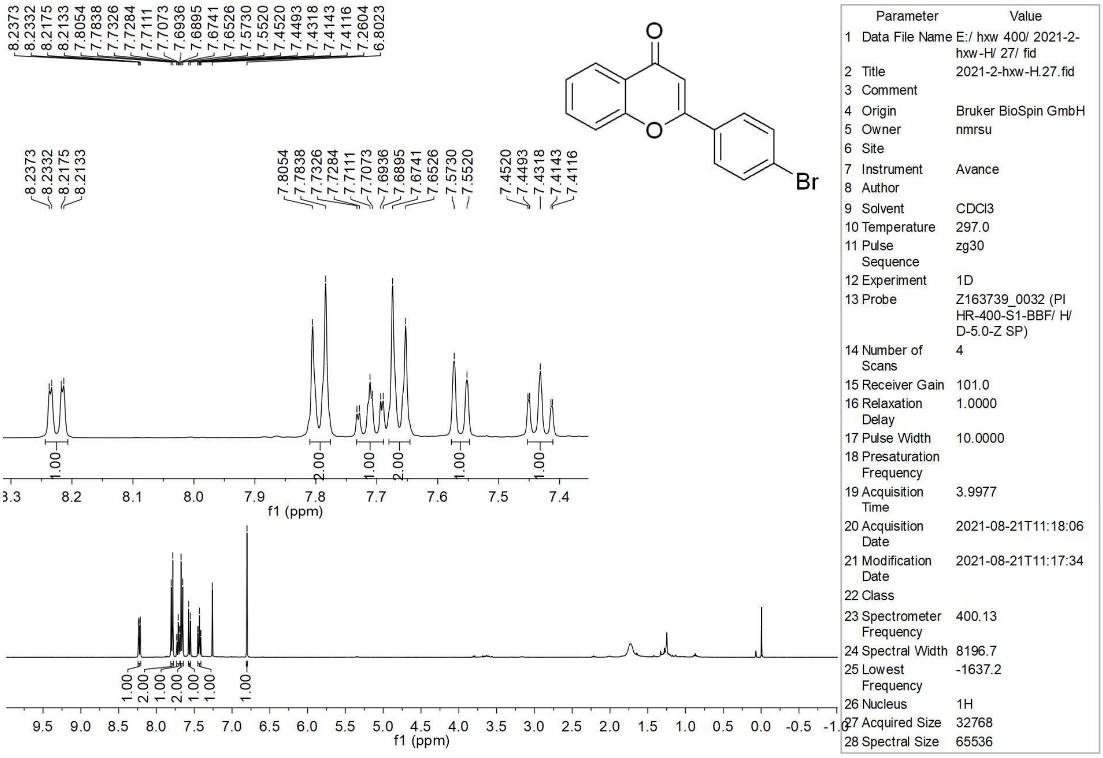

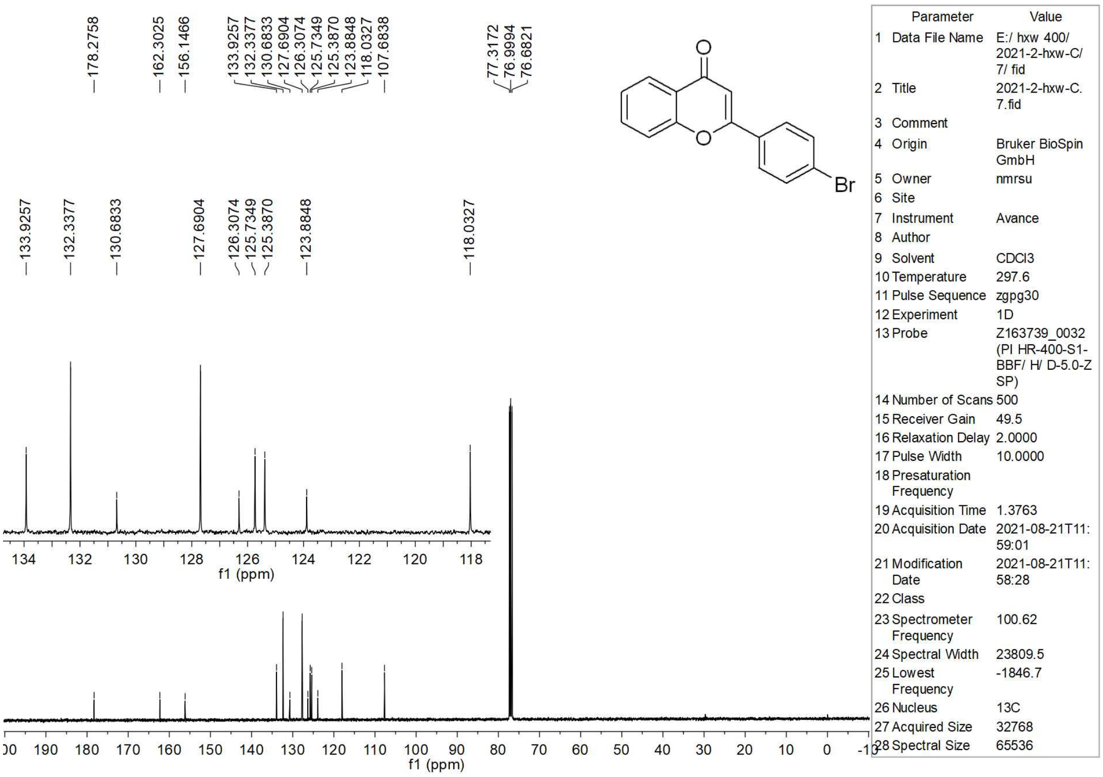

2-(2-Fluorophenyl)-4*H*-chromen-4-one (2ag)

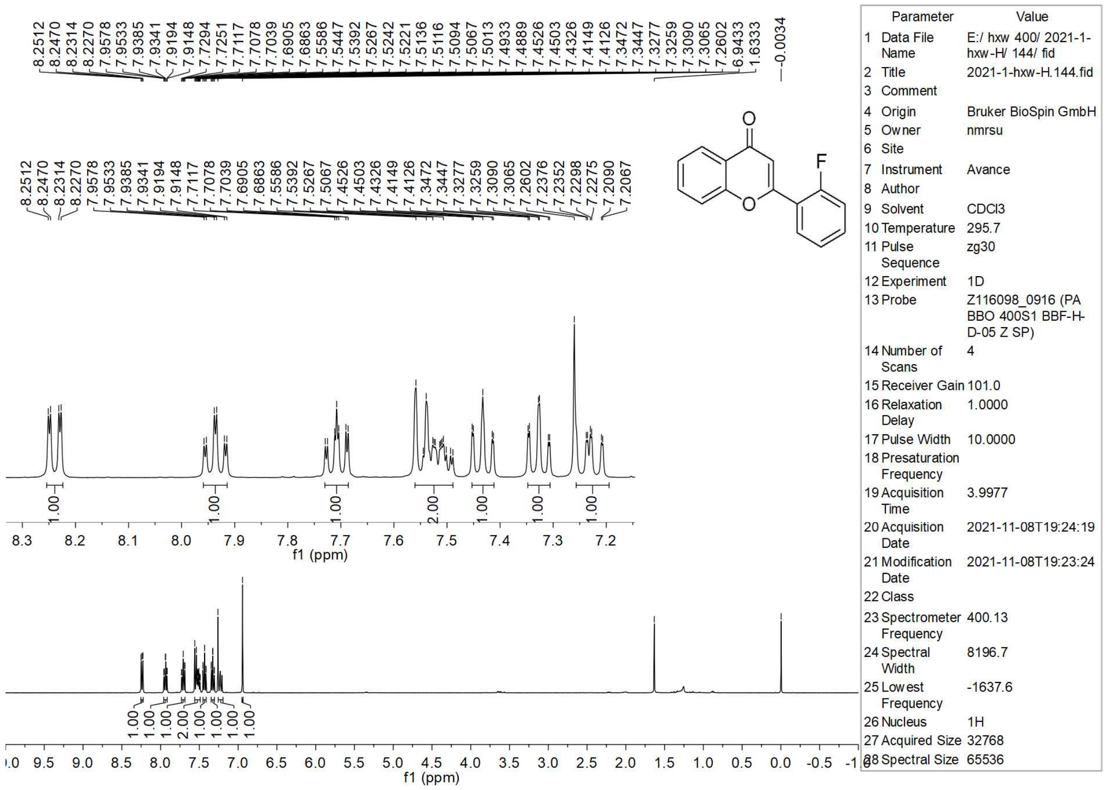

	Parameter	Value
1	Data File Name	E:/ hxw 400/ 2021-1-hxw-H/ 144/ fid
2	Title	2021-1-hxw-H.144.fid
3	Comment	
4	Origin	Bruker BioSpin GmbH
5	Owner	nmrsu
6	Site	
7	Instrument	Avance
8	Author	
9	Solvent	CDCl3
10	Temperature	295.7
11	Pulse Sequence	zg30
12	Experiment	1D
13	Probe	Z116098_0916 (PA BBO 400S1 BBF-H-D-05 Z SP)
14	Number of Scans	4
15	Receiver Gain	101.0
16	Relaxation Delay	1.0000
17	Pulse Width	10.0000
18	Presaturation Frequency	
19	Acquisition Time	3.9977
20	Acquisition Date	2021-11-08T19:24:19
21	Modification Date	2021-11-08T19:23:24
22	Class	
23	Spectrometer Frequency	400.13
24	Spectral Width	8196.7
25	Lowest Frequency	-1637.6
26	Nucleus	1H
27	Acquired Size	32768
28	Spectral Size	65536

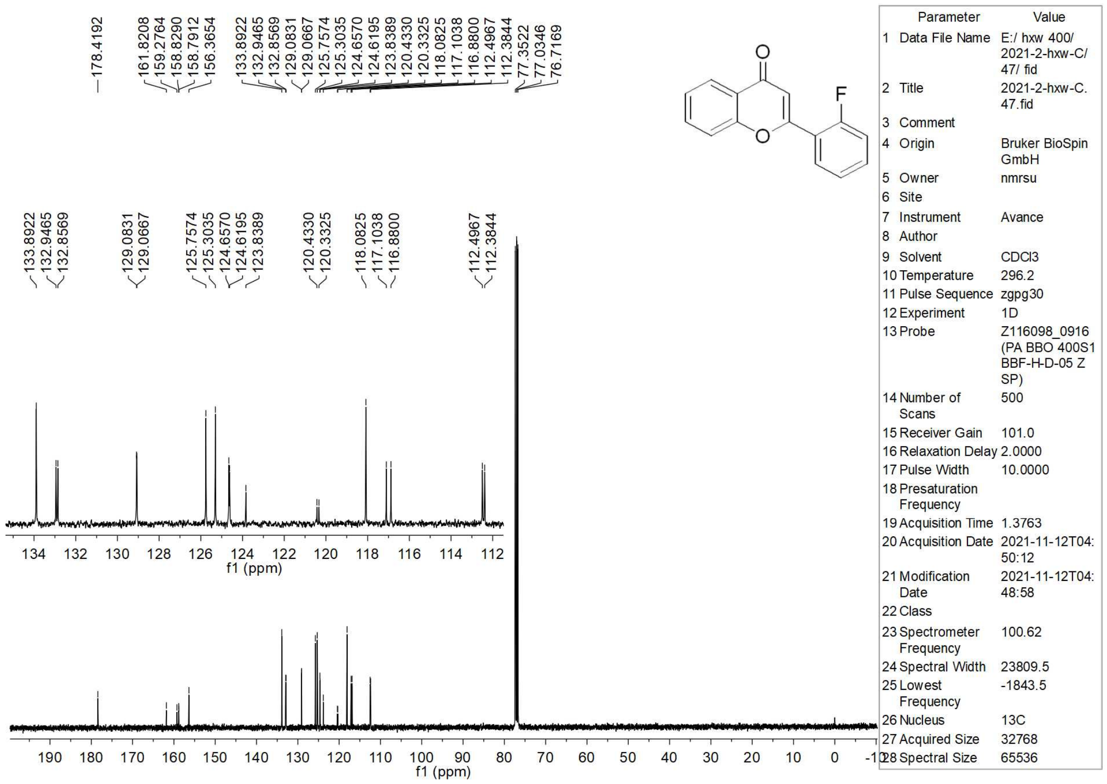

	Parameter	Value
1	Data File Name	E:/ hxw 400/ 2021-2-hxw-C/ 47/ fid
2	Title	2021-2-hxw-C.47.fid
3	Comment	
4	Origin	Bruker BioSpin GmbH
5	Owner	nmrsu
6	Site	
7	Instrument	Avance
8	Author	
9	Solvent	CDCl3
10	Temperature	296.2
11	Pulse Sequence	zgpg30
12	Experiment	1D
13	Probe	Z116098_0916 (PA BBO 400S1 BBF-H-D-05 Z SP)
14	Number of Scans	500
15	Receiver Gain	101.0
16	Relaxation Delay	2.0000
17	Pulse Width	10.0000
18	Presaturation Frequency	
19	Acquisition Time	1.3763
20	Acquisition Date	2021-11-12T04:50:12
21	Modification Date	2021-11-12T04:48:58
22	Class	
23	Spectrometer Frequency	100.62
24	Spectral Width	23809.5
25	Lowest Frequency	-1843.5
26	Nucleus	13C
27	Acquired Size	32768
28	Spectral Size	65536

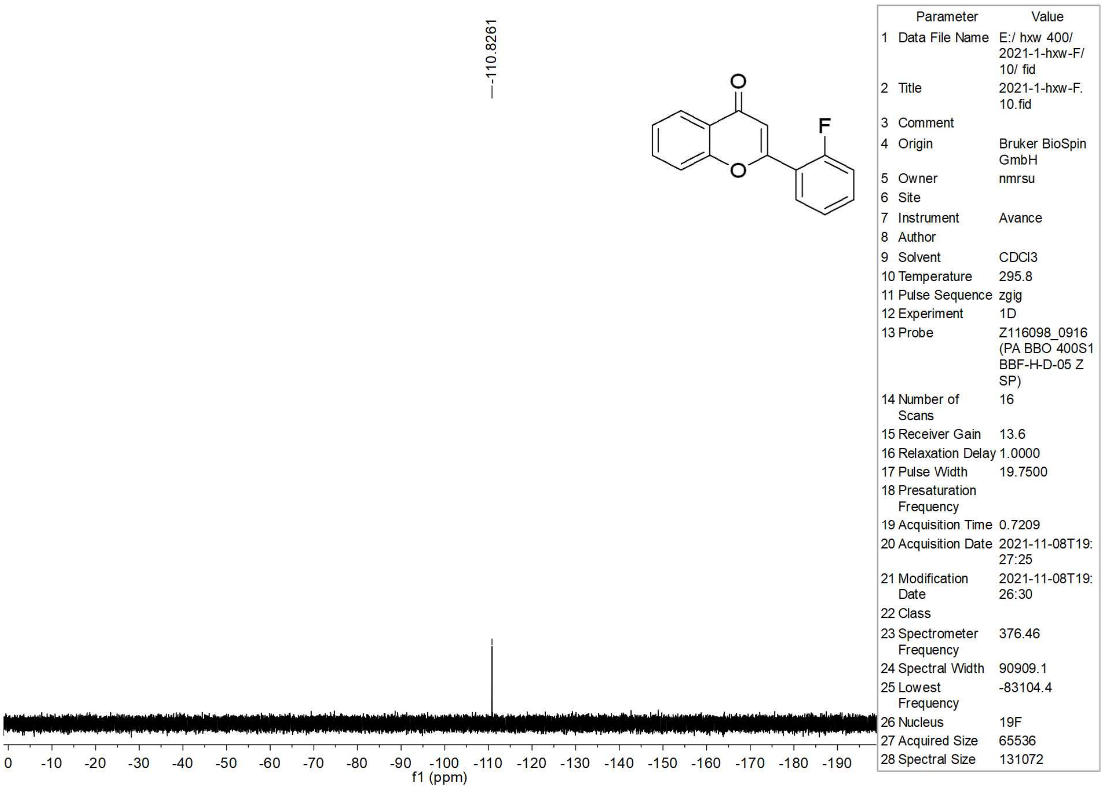

	Parameter	Value
1	Data File Name	E:/ hxw 400/ 2021-1-hxw-F/ 10/ fid
2	Title	2021-1-hxw-F. 10.fid
3	Comment	
4	Origin	Bruker BioSpin GmbH
5	Owner	nmrsu
6	Site	
7	Instrument	Avance
8	Author	
9	Solvent	CDCl3
10	Temperature	295.8
11	Pulse Sequence	zgig
12	Experiment	1D
13	Probe	Z116098_0916 (PA BBO 400S1 BBF-H-D-05 Z SP)
14	Number of Scans	16
15	Receiver Gain	13.6
16	Relaxation Delay	1.0000
17	Pulse Width	19.7500
18	Presaturation Frequency	
19	Acquisition Time	0.7209
20	Acquisition Date	2021-11-08T19:27:25
21	Modification Date	2021-11-08T19:26:30
22	Class	
23	Spectrometer Frequency	376.46
24	Spectral Width	90909.1
25	Lowest Frequency	-83104.4
26	Nucleus	19F
27	Acquired Size	65536
28	Spectral Size	131072

2-(3-Chlorophenyl)-4*H*-chromen-4-one (2ah)

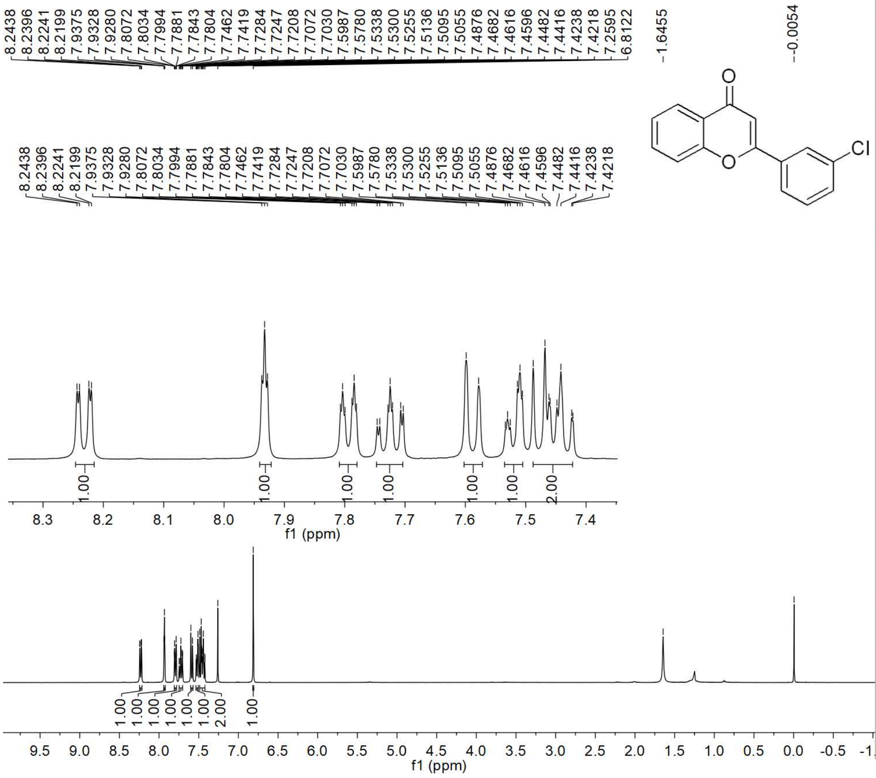

	Parameter	Value
1	Data File Name	E:/ hxw 400/ 2021-2-hxw-H/ 130/ fid
2	Title	2021-2-hxw-H. 130.fid
3	Comment	
4	Origin	Bruker BioSpin GmbH
5	Owner	nmrsu
6	Site	
7	Instrument	Avance
8	Author	
9	Solvent	CDCl3
10	Temperature	294.7
11	Pulse Sequence	zg30
12	Experiment	1D
13	Probe	Z116098_0916 (PA BBO 400S1 BBF-H-D-05 Z SP)
14	Number of Scans	4
15	Receiver Gain	101.0
16	Relaxation Delay	1.0000
17	Pulse Width	10.0000
18	Presaturation Frequency	
19	Acquisition Time	3.9977
20	Acquisition Date	2021-11-02T19:43:44
21	Modification Date	2021-11-02T19:43:22
22	Class	
23	Spectrometer Frequency	400.13
24	Spectral Width	8196.7
25	Lowest Frequency	-1638.0
26	Nucleus	1H
27	Acquired Size	32768
28	Spectral Size	65536

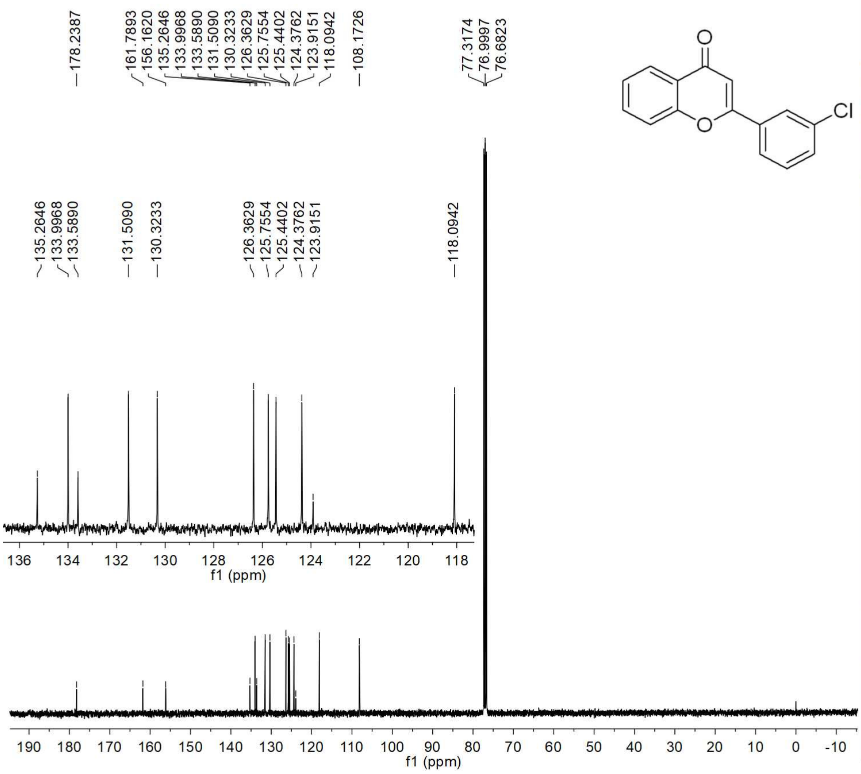

	Parameter	Value
1	Data File Name	E:/ hxw 400/ 2021-2-hxw-C/ 50/ fid
2	Title	2021-2-hxw-C.50.fid
3	Comment	
4	Origin	Bruker BioSpin GmbH
5	Owner	nmrsu
6	Site	
7	Instrument	Avance
8	Author	
9	Solvent	CDCl3
10	Temperature	296.2
11	Pulse Sequence	zgpg30
12	Experiment	1D
13	Probe	Z116098_0916 (PA BBO 400S1 BBF-H-D-05 Z SP)
14	Number of Scans	500
15	Receiver Gain	101.0
16	Relaxation Delay	2.0000
17	Pulse Width	10.0000
18	Presaturation Frequency	
19	Acquisition Time	1.3763
20	Acquisition Date	2021-11-12T06:27:55
21	Modification Date	2021-11-12T06:26:40
22	Class	
23	Spectrometer Frequency	100.62
24	Spectral Width	23809.5
25	Lowest Frequency	-1846.6
26	Nucleus	13C
27	Acquired Size	32768
28	Spectral Size	65536

2-(*m*-Tolyl)-4*H*-chromen-4-one (2ai)

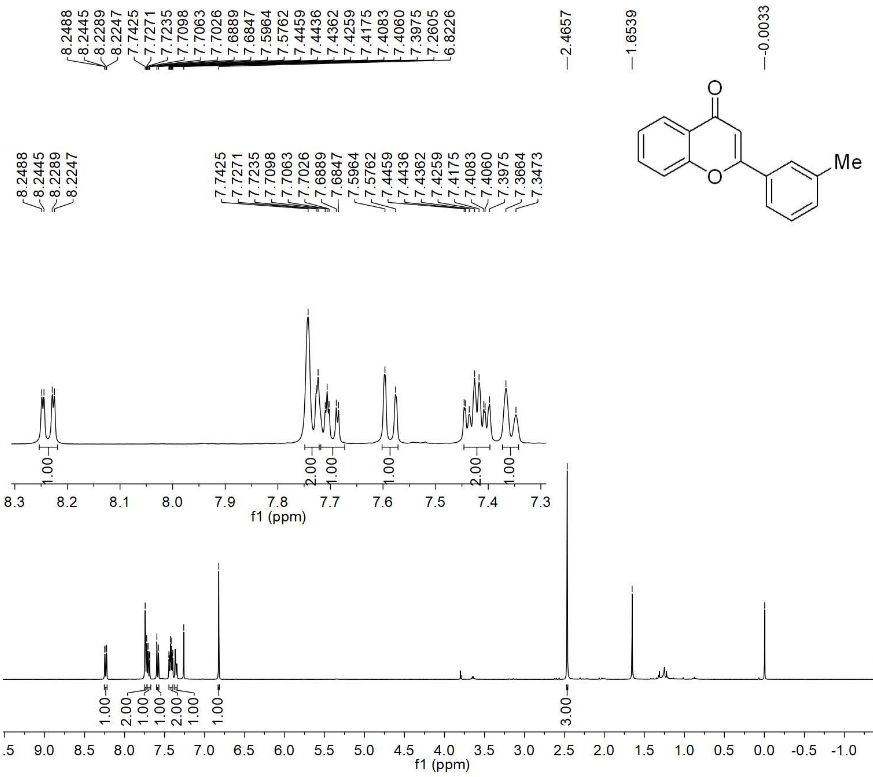

Parameter		Value
1	Data File Name	E:/ hxw 400/ 2021-2-hxw-H/ 214/ fid
2	Title	2021-2-hxw-H. 214.fid
3	Comment	
4	Origin	Bruker BioSpin GmbH
5	Owner	nmrsu
6	Site	
7	Instrument	Avance
8	Author	
9	Solvent	CDCl3
10	Temperature	293.6
11	Pulse Sequence	zg30
12	Experiment	1D
13	Probe	Z116098_0916 (PA BBO 400S1 BBF-H-D-05 Z SP)
14	Number of Scans	4
15	Receiver Gain	101.0
16	Relaxation Delay	1.0000
17	Pulse Width	10.0000
18	Presaturation Frequency	
19	Acquisition Time	3.9977
20	Acquisition Date	2021-12-15T11:47:50
21	Modification Date	2021-12-15T11:47:22
22	Class	
23	Spectrometer Frequency	400.13
24	Spectral Width	8196.7
25	Lowest Frequency	-1637.6
26	Nucleus	1H
27	Acquired Size	32768
28	Spectral Size	65536

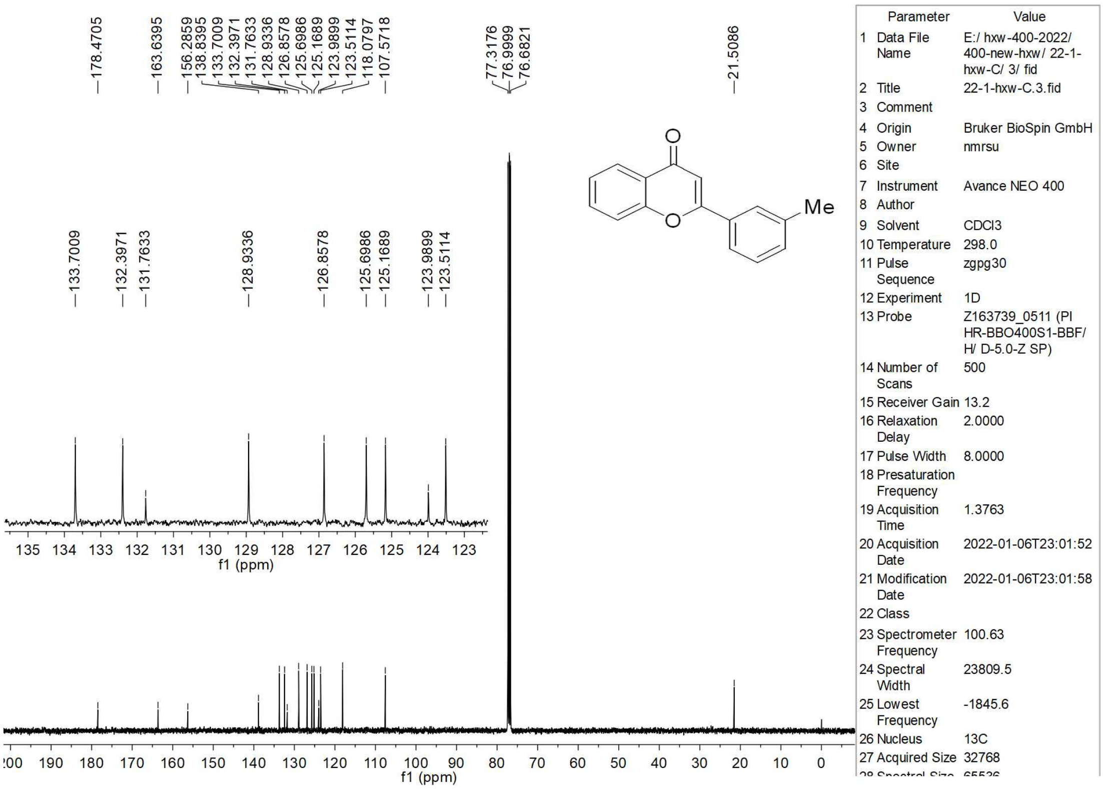

Parameter		Value
1	Data File Name	E:/ hxw-400-2022/ 400-new-hxw/ 22-1-hxw-C/ 3/ fid
2	Title	22-1-hxw-C.3.fid
3	Comment	
4	Origin	Bruker BioSpin GmbH
5	Owner	nmrsu
6	Site	
7	Instrument	Avance NEO 400
8	Author	
9	Solvent	CDCl3
10	Temperature	298.0
11	Pulse Sequence	zgpg30
12	Experiment	1D
13	Probe	Z163739_0511 (PI HR-BBO400S1-BBF/ H/ D-5.0-Z SP)
14	Number of Scans	500
15	Receiver Gain	13.2
16	Relaxation Delay	2.0000
17	Pulse Width	8.0000
18	Presaturation Frequency	
19	Acquisition Time	1.3763
20	Acquisition Date	2022-01-06T23:01:52
21	Modification Date	2022-01-06T23:01:58
22	Class	
23	Spectrometer Frequency	100.63
24	Spectral Width	23809.5
25	Lowest Frequency	-1845.6
26	Nucleus	13C
27	Acquired Size	32768
28	[illegible]	[illegible]

2-(3,5-Bis(trifluoromethyl)phenyl)-4*H*-chromen-4-one (2aj)

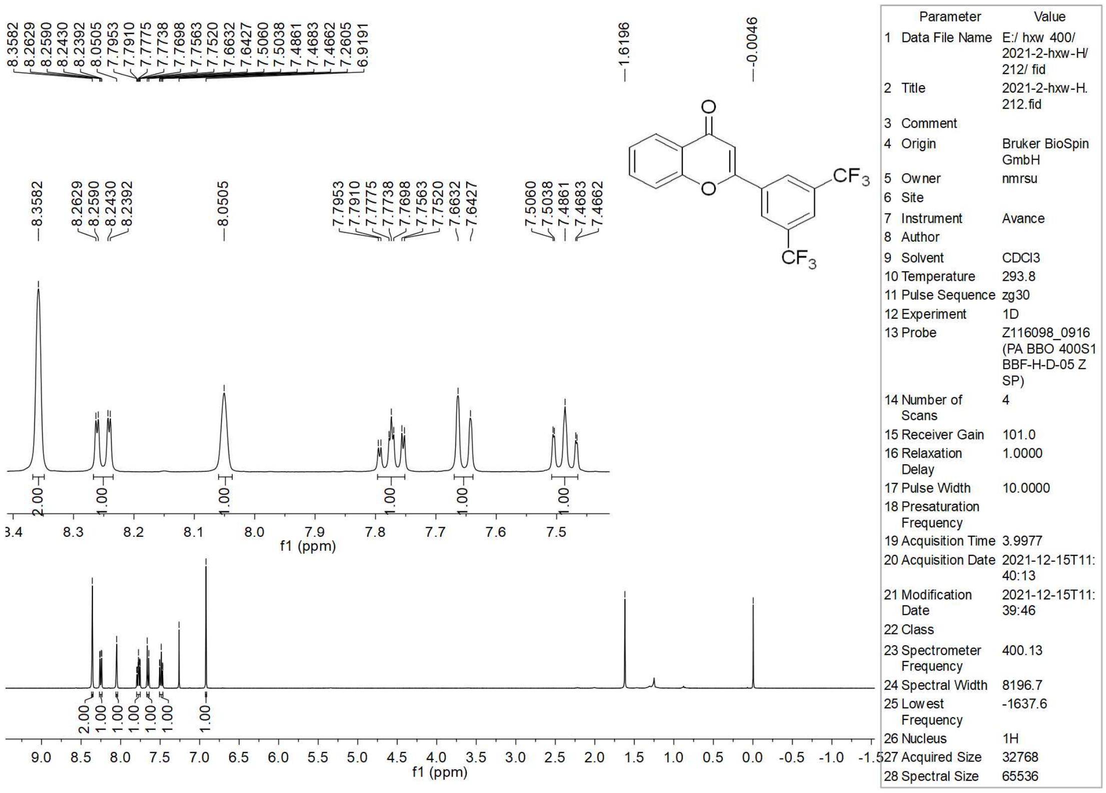

	Parameter	Value
1	Data File Name	E:/ hxw 400/ 2021-2-hxw-H/ 212/ fid
2	Title	2021-2-hxw-H. 212.fid
3	Comment	
4	Origin	Bruker BioSpin GmbH
5	Owner	nmrsu
6	Site	
7	Instrument	Avance
8	Author	
9	Solvent	CDCl3
10	Temperature	293.8
11	Pulse Sequence	zg30
12	Experiment	1D
13	Probe	Z116098_0916 (PA BBO 400S1 BBF-H-D-05 Z SP)
14	Number of Scans	4
15	Receiver Gain	101.0
16	Relaxation Delay	1.0000
17	Pulse Width	10.0000
18	Presaturation Frequency	
19	Acquisition Time	3.9977
20	Acquisition Date	2021-12-15T11:40:13
21	Modification Date	2021-12-15T11:39:46
22	Class	
23	Spectrometer Frequency	400.13
24	Spectral Width	8196.7
25	Lowest Frequency	-1637.6
26	Nucleus	1H
27	Acquired Size	32768
28	Spectral Size	65536

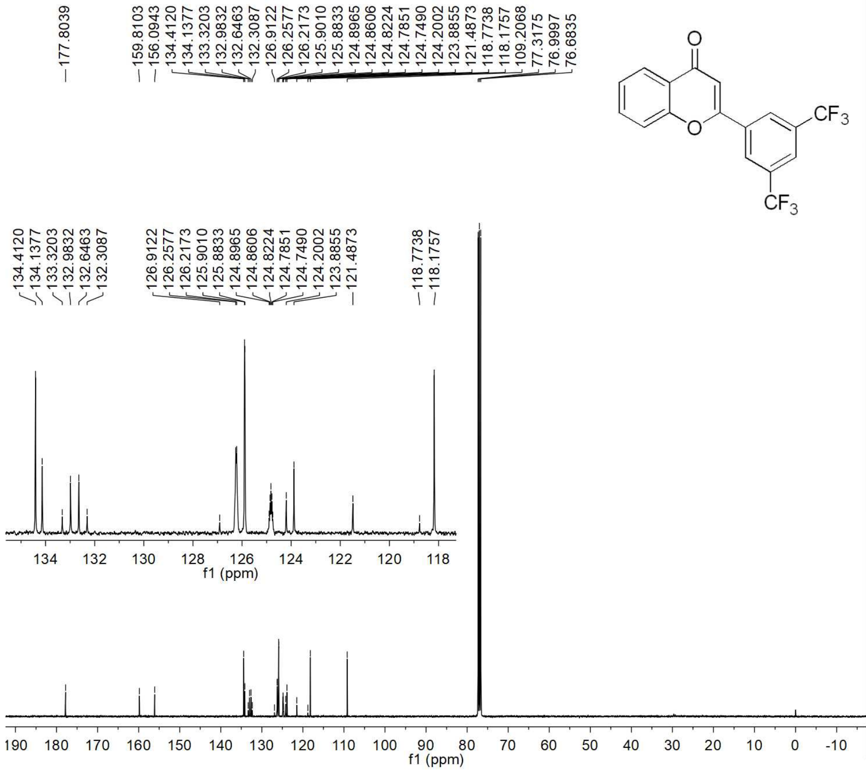

	Parameter	Value
1	Data File Name	E:/ hxw-400-2022/ 400-new-hxw/ 22-1-hxw-C/ 7/ fid
2	Title	22-1-hxw-C.7.fid
3	Comment	
4	Origin	Bruker BioSpin GmbH
5	Owner	nmrsu
6	Site	
7	Instrument	Avance NEO 400
8	Author	
9	Solvent	CDCl3
10	Temperature	298.0
11	Pulse Sequence	zgpg30
12	Experiment	1D
13	Probe	Z163739_0511 (PI HR-BBO400S1-BBF/H/ D-5.0-Z SP)
14	Number of Scans	3000
15	Receiver Gain	13.2
16	Relaxation Delay	2.0000
17	Pulse Width	8.0000
18	Presaturation Frequency	
19	Acquisition Time	1.3763
20	Acquisition Date	2022-01-07T03:53:23
21	Modification Date	2022-01-07T03:53:28
22	Class	
23	Spectrometer Frequency	100.63
24	Spectral Width	23809.5
25	Lowest Frequency	-1844.9
26	Nucleus	13C
27	Acquired Size	32768
28	Spectral Size	65536

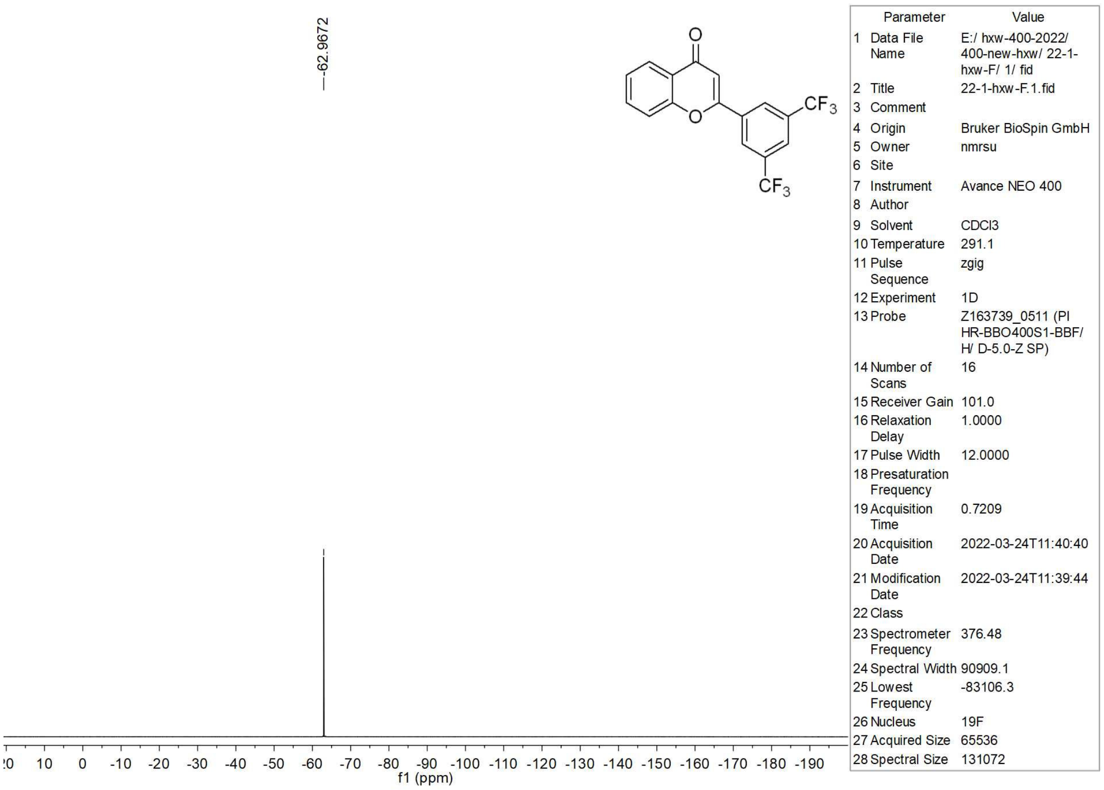

	Parameter	Value
1	Data File Name	E:/ hxw-400-2022/ 400-new-hxw/ 22-1-hxw-F/ 1/ fid
2	Title	22-1-hxw-F.1.fid
3	Comment	
4	Origin	Bruker BioSpin GmbH
5	Owner	nmrsu
6	Site	
7	Instrument	Avance NEO 400
8	Author	
9	Solvent	CDCl3
10	Temperature	291.1
11	Pulse Sequence	zgig
12	Experiment	1D
13	Probe	Z163739_0511 (PI HR-BBO400S1-BBF/ H/ D-5.0-Z SP)
14	Number of Scans	16
15	Receiver Gain	101.0
16	Relaxation Delay	1.0000
17	Pulse Width	12.0000
18	Presaturation Frequency	
19	Acquisition Time	0.7209
20	Acquisition Date	2022-03-24T11:40:40
21	Modification Date	2022-03-24T11:39:44
22	Class	
23	Spectrometer Frequency	376.48
24	Spectral Width	90909.1
25	Lowest Frequency	-83106.3
26	Nucleus	19F
27	Acquired Size	65536
28	Spectral Size	131072

2-(Thiophen-3-yl)-4*H*-chromen-4-one (2ak)

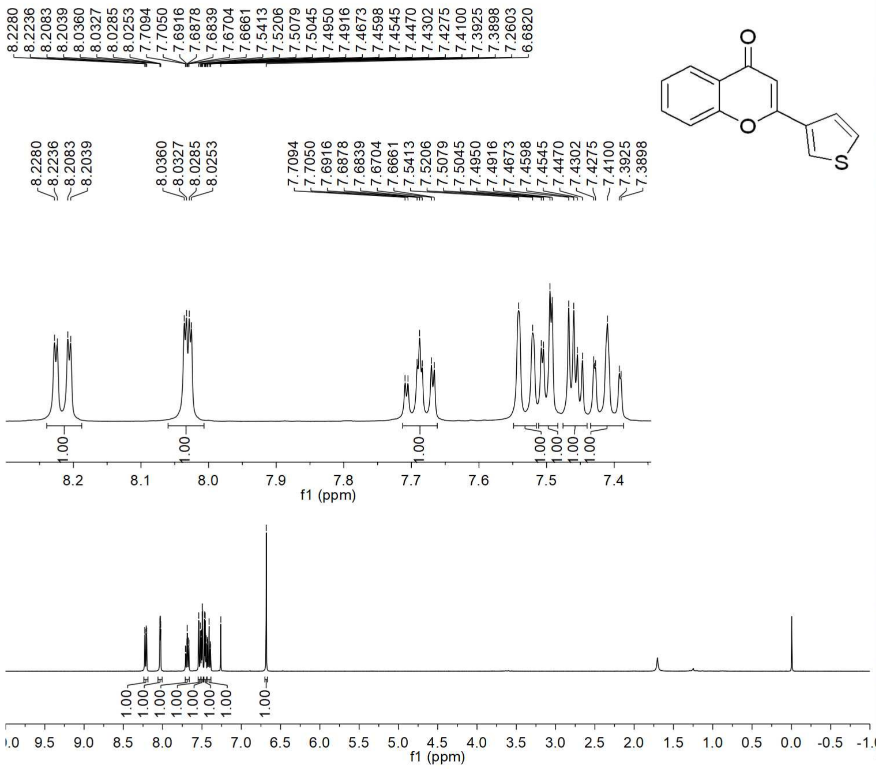

Parameter	Value
1 Data File Name	E:/ hxw 400/ 2021-2-hxw-H/ 115/ fid
2 Title	2021-2-hxw-H. 115.fid
3 Comment	
4 Origin	Bruker BioSpin GmbH
5 Owner	nmrsu
6 Site	
7 Instrument	Avance
8 Author	
9 Solvent	CDCl3
10 Temperature	294.8
11 Pulse Sequence	zg30
12 Experiment	1D
13 Probe	Z116098_0916 (PA BBO 400S1 BBF-H-D-05 Z SP)
14 Number of Scans	4
15 Receiver Gain	101.0
16 Relaxation Delay	1.0000
17 Pulse Width	10.0000
18 Presaturation Frequency	
19 Acquisition Time	3.9977
20 Acquisition Date	2021-10-28T19:30:20
21 Modification Date	2021-10-28T19:29:06
22 Class	
23 Spectrometer Frequency	400.13
24 Spectral Width	8196.7
25 Lowest Frequency	-1637.6
26 Nucleus	1H
27 Acquired Size	32768
28 Spectral Size	65536

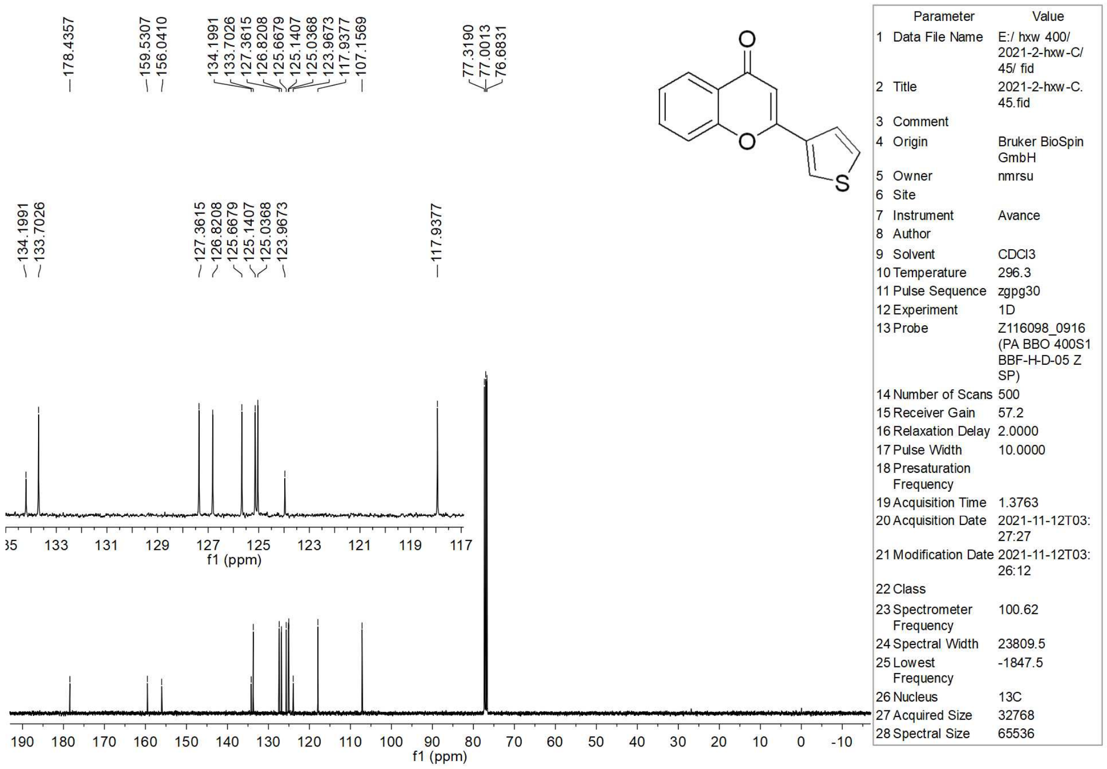

Parameter	Value
1 Data File Name	E:/ hxw 400/ 2021-2-hxw-C/ 45/ fid
2 Title	2021-2-hxw-C. 45.fid
3 Comment	
4 Origin	Bruker BioSpin GmbH
5 Owner	nmrsu
6 Site	
7 Instrument	Avance
8 Author	
9 Solvent	CDCl3
10 Temperature	296.3
11 Pulse Sequence	zgpg30
12 Experiment	1D
13 Probe	Z116098_0916 (PA BBO 400S1 BBF-H-D-05 Z SP)
14 Number of Scans	500
15 Receiver Gain	57.2
16 Relaxation Delay	2.0000
17 Pulse Width	10.0000
18 Presaturation Frequency	
19 Acquisition Time	1.3763
20 Acquisition Date	2021-11-12T03:27:27
21 Modification Date	2021-11-12T03:26:12
22 Class	
23 Spectrometer Frequency	100.62
24 Spectral Width	23809.5
25 Lowest Frequency	-1847.5
26 Nucleus	13C
27 Acquired Size	32768
28 Spectral Size	65536

2-(Naphthalen-2-yl)-4*H*-chromen-4-one (2al)

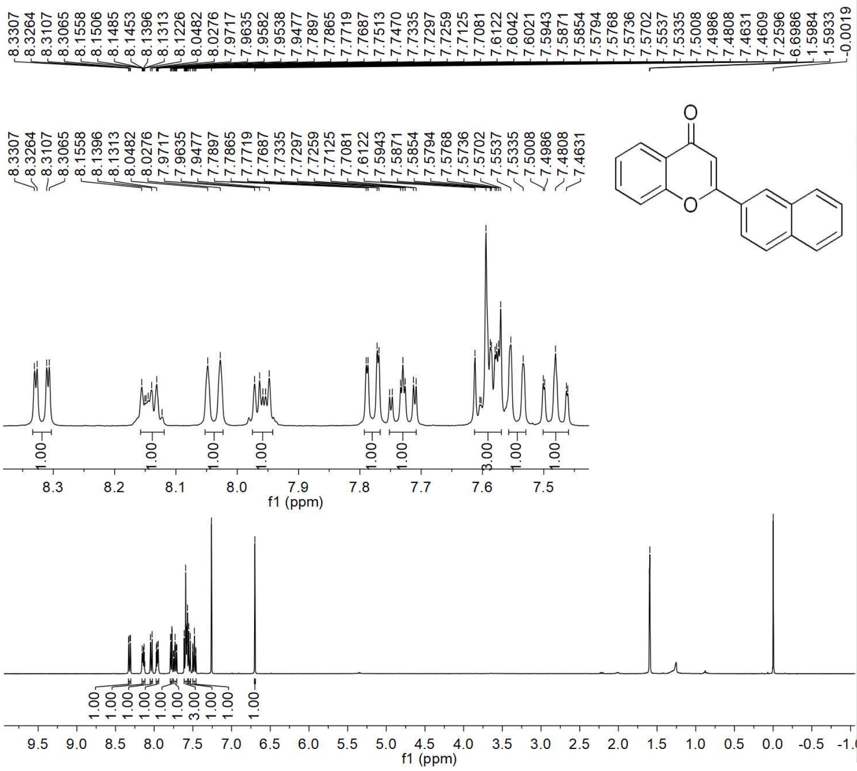

	Parameter	Value
1	Data File Name	E:/ hxw 400/ 2021-2-hxw-H/ 112/ fid
2	Title	2021-2-hxw-H.112.fid
3	Comment	
4	Origin	Bruker BioSpin GmbH
5	Owner	nmrsu
6	Site	
7	Instrument	Avance
8	Author	
9	Solvent	CDCl3
10	Temperature	294.9
11	Pulse Sequence	zg30
12	Experiment	1D
13	Probe	Z116098_0916 (PA BBO 400S1 BBF-H-D-05 Z SP)
14	Number of Scans	4
15	Receiver Gain	101.0
16	Relaxation Delay	1.0000
17	Pulse Width	10.0000
18	Presaturation Frequency	
19	Acquisition Time	3.9977
20	Acquisition Date	2021-10-28T19:18:10
21	Modification Date	2021-10-28T19:16:56
22	Class	
23	Spectrometer Frequency	400.13
24	Spectral Width	8196.7
25	Lowest Frequency	-1638.0
26	Nucleus	1H
27	Acquired Size	32768
28	Spectral Size	65536

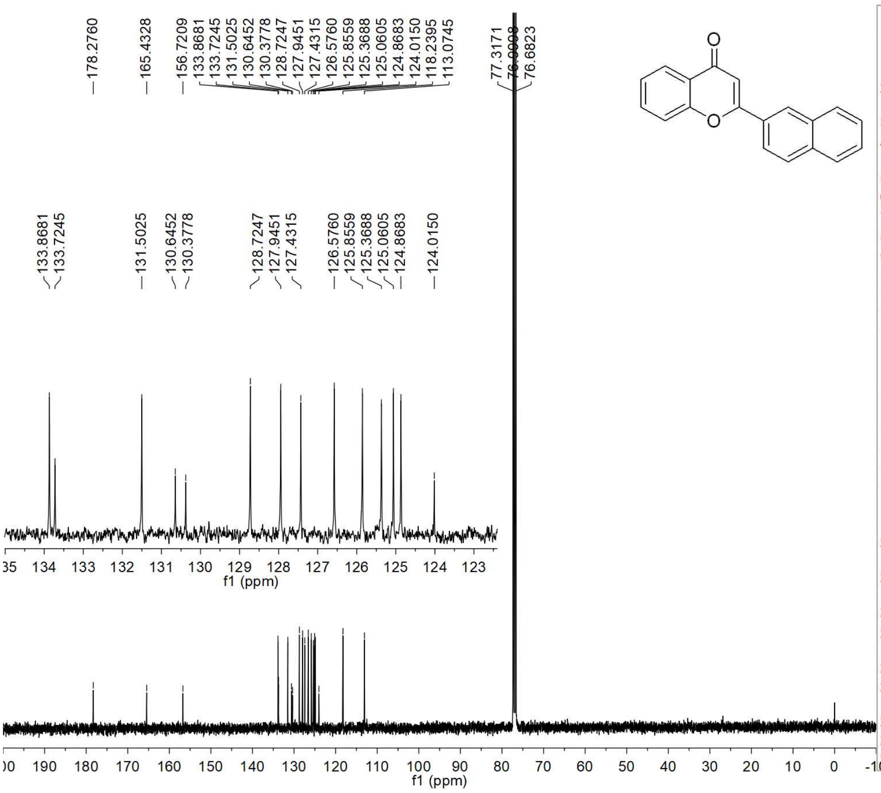

	Parameter	Value
1	Data File Name	E:/ hxw 400/ 2021-2-hxw-C/ 41/ fid
2	Title	2021-2-hxw-C.41.fid
3	Comment	
4	Origin	Bruker BioSpin GmbH
5	Owner	nmrsu
6	Site	
7	Instrument	Avance
8	Author	
9	Solvent	CDCl3
10	Temperature	296.4
11	Pulse Sequence	zgpg30
12	Experiment	1D
13	Probe	Z116098_0916 (PA BBO 400S1 BBF-H-D-05 Z SP)
14	Number of Scans	800
15	Receiver Gain	101.0
16	Relaxation Delay	2.0000
17	Pulse Width	10.0000
18	Presaturation Frequency	
19	Acquisition Time	1.3763
20	Acquisition Date	2021-11-12T01:42:41
21	Modification Date	2021-11-12T01:41:28
22	Class	
23	Spectrometer Frequency	100.62
24	Spectral Width	23809.5
25	Lowest Frequency	-1846.2
26	Nucleus	13C
27	Acquired Size	32768
28	Spectral Size	65536

2-(Pyren-2-yl)-4*H*-chromen-4-one (2am)

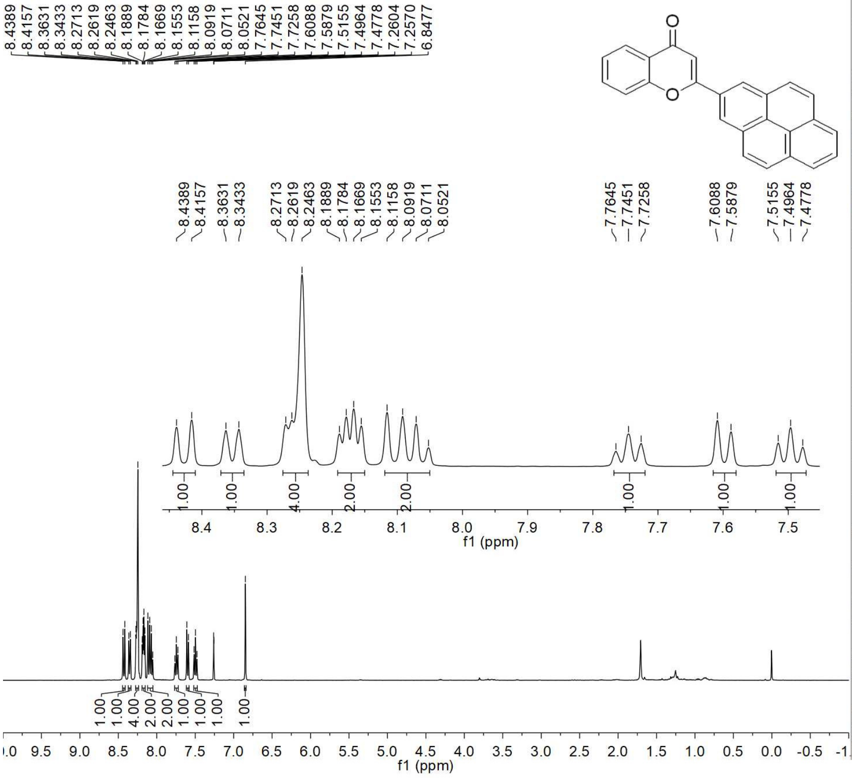

Parameter		Value
1	Data File Name	E:/ hxw-400-2022/ 400-new-hxw/ 22-1-hxw-H/ 41/ fid
2	Title	22-1-hxw-H.41.fid
3	Comment	
4	Origin	Bruker BioSpin GmbH
5	Owner	nmrsu
6	Site	
7	Instrument	Avance NEO 400
8	Author	
9	Solvent	CDCl3
10	Temperature	290.9
11	Pulse Sequence	zg30
12	Experiment	1D
13	Probe	Z163739_0511 (PI HR-BBO400S1-BBF/ H/ D-5.0-Z SP)
14	Number of Scans	4
15	Receiver Gain	101.0
16	Relaxation Delay	1.0000
17	Pulse Width	8.0000
18	Presaturation Frequency	
19	Acquisition Time	3.9977
20	Acquisition Date	2022-03-22T19:20:20
21	Modification Date	2022-03-22T19:19:24
22	Class	
23	Spectrometer Frequency	400.15
24	Spectral Width	8196.7
25	Lowest Frequency	-1638.2
26	Nucleus	1H
27	Acquired Size	32768
28	Spectral Size	65536

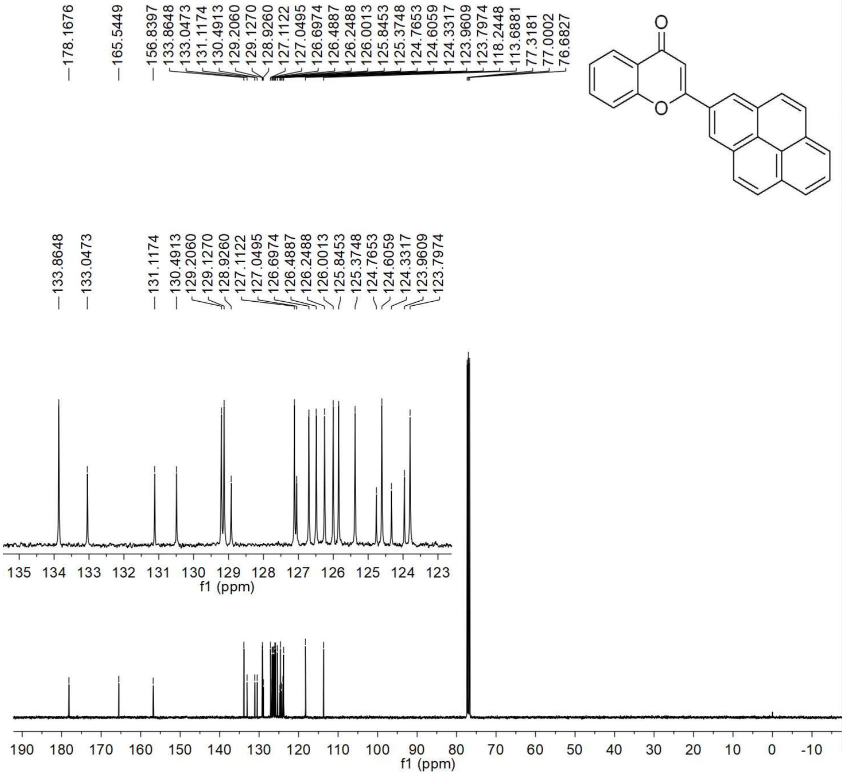

Parameter		Value
1	Data File Name	E:/ hxw-400-2022/ 400-new-hxw/ 22-1-hxw-C/ 27/ fid
2	Title	22-1-hxw-C.27.fid
3	Comment	
4	Origin	Bruker BioSpin GmbH
5	Owner	nmrsu
6	Site	
7	Instrument	Avance NEO 400
8	Author	
9	Solvent	CDCl3
10	Temperature	291.7
11	Pulse Sequence	zgpg30
12	Experiment	1D
13	Probe	Z163739_0511 (PI HR-BBO400S1-BBF/ H/ D-5.0-Z SP)
14	Number of Scans	600
15	Receiver Gain	13.2
16	Relaxation Delay	2.0000
17	Pulse Width	8.0000
18	Presaturation Frequency	
19	Acquisition Time	1.3763
20	Acquisition Date	2022-03-23T11:47:16
21	Modification Date	2022-03-23T11:46:20
22	Class	
23	Spectrometer Frequency	100.63
24	Spectral Width	23809.5
25	Lowest Frequency	-1849.8
26	Nucleus	13C
27	Acquired Size	32768
28	Spectral Size	65536

6-Methyl-2-(*p*-tolyl)-4*H*-chromen-4-one (2bb)

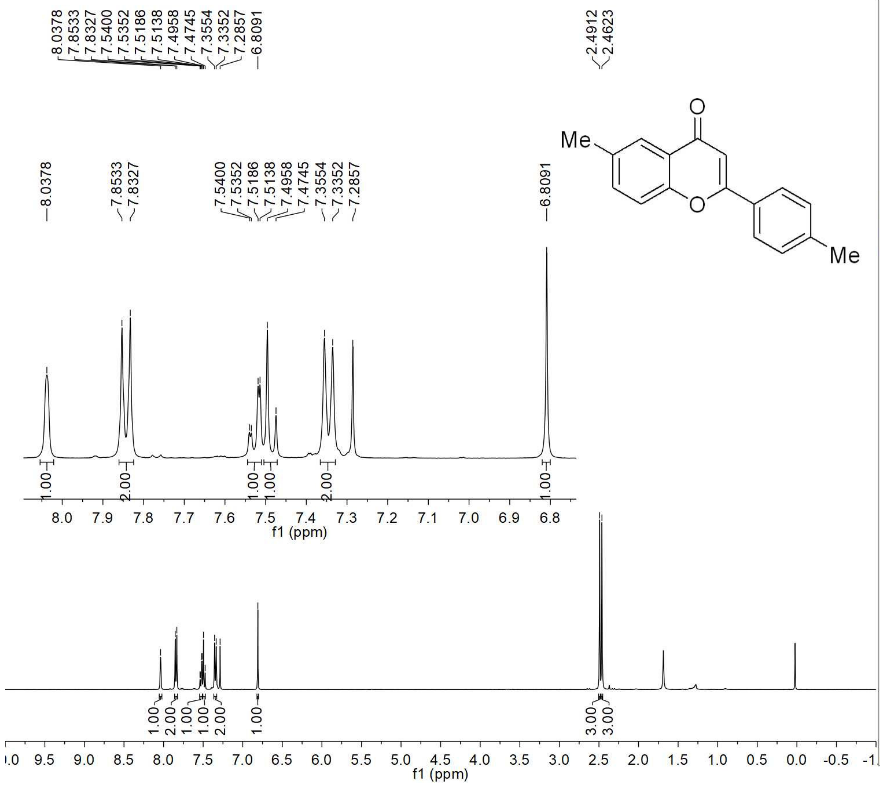

Parameter		Value
1	Data File Name	E:/ hxw 400/ 2021-2-hxw-H/ 127/ fid
2	Title	2021-2-hxw-H.127.fid
3	Comment	
4	Origin	Bruker BioSpin GmbH
5	Owner	nmrsu
6	Site	
7	Instrument	Avance
8	Author	
9	Solvent	CDCl3
10	Temperature	294.8
11	Pulse Sequence	zg30
12	Experiment	1D
13	Probe	Z116098_0916 (PA BBO 400S1 BBF-H-D-05 Z SP)
14	Number of Scans	4
15	Receiver Gain	101.0
16	Relaxation Delay	1.0000
17	Pulse Width	10.0000
18	Presaturation Frequency	
19	Acquisition Time	3.9977
20	Acquisition Date	2021-11-02T19:31:52
21	Modification Date	2021-11-02T19:31:30
22	Class	
23	Spectrometer Frequency	400.13
24	Spectral Width	8196.7
25	Lowest Frequency	-1627.6
26	Nucleus	1H
27	Acquired Size	32768
28	Spectral Size	65536

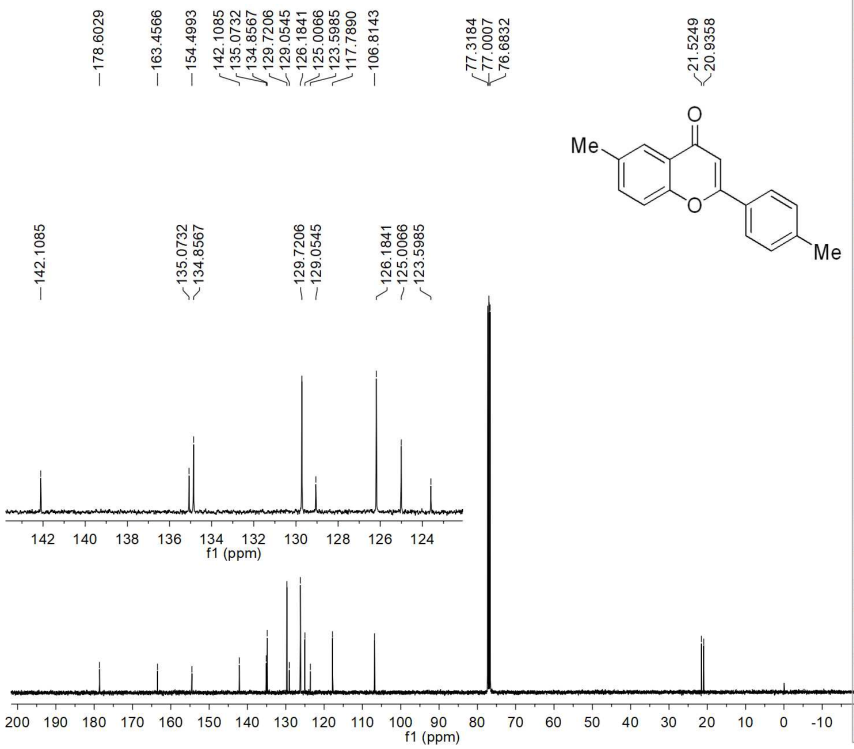

Parameter		Value
1	Data File Name	E:/ hxw 400/ 2021-2-hxw-C/ 48/ fid
2	Title	2021-2-hxw-C.48.fid
3	Comment	
4	Origin	Bruker BioSpin GmbH
5	Owner	nmrsu
6	Site	
7	Instrument	Avance
8	Author	
9	Solvent	CDCl3
10	Temperature	296.3
11	Pulse Sequence	zgpg30
12	Experiment	1D
13	Probe	Z116098_0916 (PA BBO 400S1 BBF-H-D-05 Z SP)
14	Number of Scans	500
15	Receiver Gain	101.0
16	Relaxation Delay	2.0000
17	Pulse Width	10.0000
18	Presaturation Frequency	
19	Acquisition Time	1.3763
20	Acquisition Date	2021-11-12T05:22:37
21	Modification Date	2021-11-12T05:21:22
22	Class	
23	Spectrometer Frequency	100.62
24	Spectral Width	23809.5
25	Lowest Frequency	-1846.8
26	Nucleus	13C
27	Acquired Size	32768
28	Spectral Size	65536

2-(4-Methoxyphenyl)-6-methyl-4*H*-chromen-4-one (2bc)

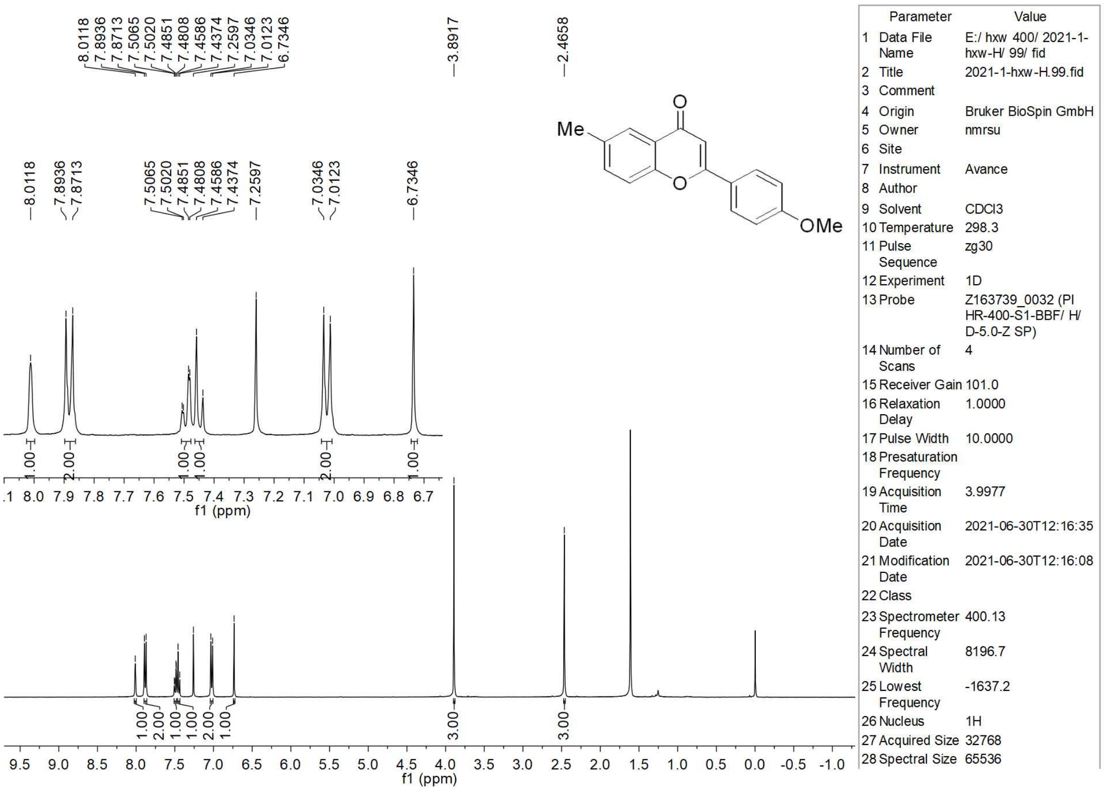

Parameter	Value
1 Data File Name	E:/ hxw 400/ 2021-1-hxw-H/ 99/ fid
2 Title	2021-1-hxw-H.99.fid
3 Comment	
4 Origin	Bruker BioSpin GmbH
5 Owner	nmrsu
6 Site	
7 Instrument	Avance
8 Author	
9 Solvent	CDCl3
10 Temperature	298.3
11 Pulse Sequence	zg30
12 Experiment	1D
13 Probe	Z163739_0032 (PI HR-400-S1-BBF/ H/ D-5.0-Z SP)
14 Number of Scans	4
15 Receiver Gain	101.0
16 Relaxation Delay	1.0000
17 Pulse Width	10.0000
18 Presaturation Frequency	
19 Acquisition Time	3.9977
20 Acquisition Date	2021-06-30T12:16:35
21 Modification Date	2021-06-30T12:16:08
22 Class	
23 Spectrometer Frequency	400.13
24 Spectral Width	8196.7
25 Lowest Frequency	-1637.2
26 Nucleus	1H
27 Acquired Size	32768
28 Spectral Size	65536

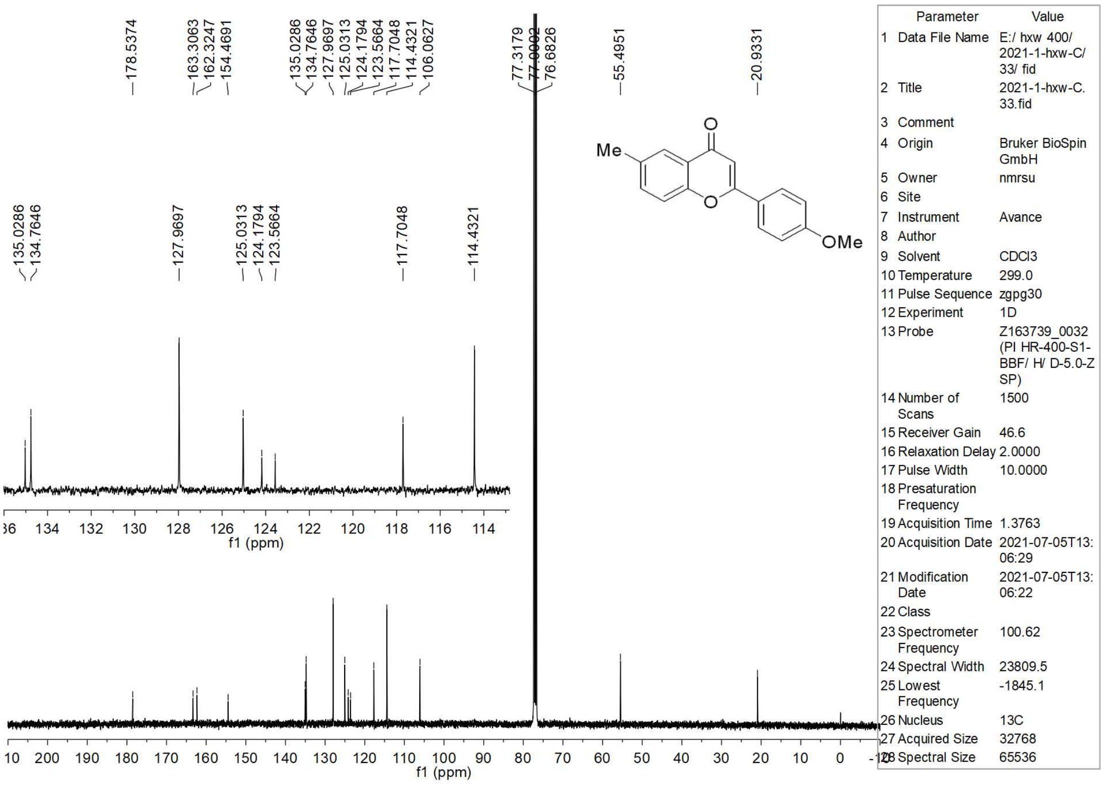

Parameter	Value
1 Data File Name	E:/ hxw 400/ 2021-1-hxw-C/ 33/ fid
2 Title	2021-1-hxw-C.33.fid
3 Comment	
4 Origin	Bruker BioSpin GmbH
5 Owner	nmrsu
6 Site	
7 Instrument	Avance
8 Author	
9 Solvent	CDCl3
10 Temperature	299.0
11 Pulse Sequence	zgpg30
12 Experiment	1D
13 Probe	Z163739_0032 (PI HR-400-S1-BBF/ H/ D-5.0-Z SP)
14 Number of Scans	1500
15 Receiver Gain	46.6
16 Relaxation Delay	2.0000
17 Pulse Width	10.0000
18 Presaturation Frequency	
19 Acquisition Time	1.3763
20 Acquisition Date	2021-07-05T13:06:29
21 Modification Date	2021-07-05T13:06:22
22 Class	
23 Spectrometer Frequency	100.62
24 Spectral Width	23809.5
25 Lowest Frequency	-1845.1
26 Nucleus	13C
27 Acquired Size	32768
28 Spectral Size	65536

2-(4-Fluorophenyl)-6-methyl-4*H*-chromen-4-one (2bd)

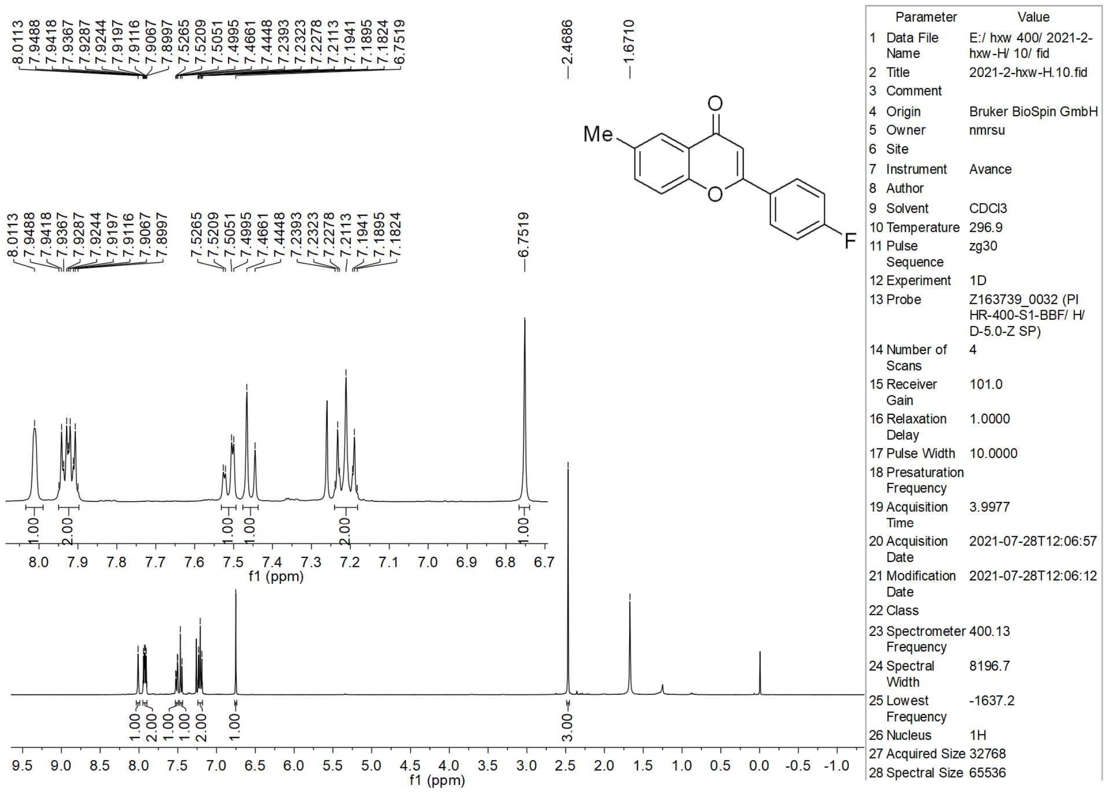

Parameter	Value
1 Data File Name	E:/ hxw 400/ 2021-2-hxw-H/ 10/ fid
2 Title	2021-2-hxw-H.10.fid
3 Comment	
4 Origin	Bruker BioSpin GmbH
5 Owner	nmrsu
6 Site	
7 Instrument	Avance
8 Author	
9 Solvent	CDCl3
10 Temperature	296.9
11 Pulse Sequence	zg30
12 Experiment	1D
13 Probe	Z163739_0032 (PI HR-400-S1-BBF/ H/ D-5.0-Z SP)
14 Number of Scans	4
15 Receiver Gain	101.0
16 Relaxation Delay	1.0000
17 Pulse Width	10.0000
18 Presaturation Frequency	
19 Acquisition Time	3.9977
20 Acquisition Date	2021-07-28T12:06:57
21 Modification Date	2021-07-28T12:06:12
22 Class	
23 Spectrometer Frequency	400.13
24 Spectral Width	8196.7
25 Lowest Frequency	-1637.2
26 Nucleus	1H
27 Acquired Size	32768
28 Spectral Size	65536

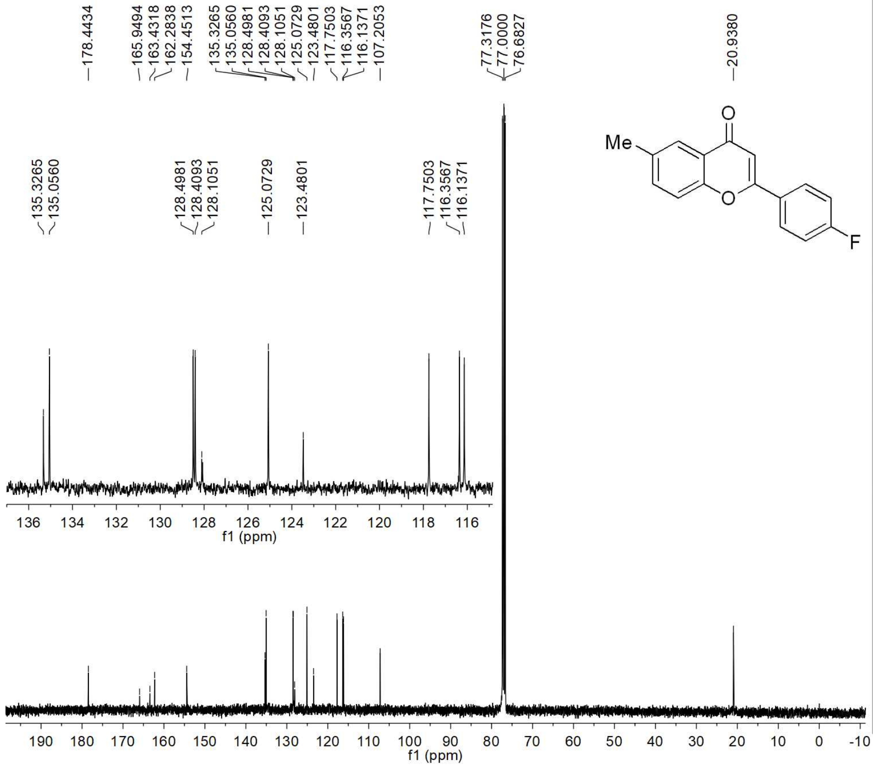

Parameter	Value
1 Data File Name	E:/ hxw 400/ 2021-2-hxw-C/ 5/ fid
2 Title	2021-2-hxw-C.5.fid
3 Comment	
4 Origin	Bruker BioSpin GmbH
5 Owner	nmrsu
6 Site	
7 Instrument	Avance
8 Author	
9 Solvent	CDCl3
10 Temperature	297.8
11 Pulse Sequence	zgpg30
12 Experiment	1D
13 Probe	Z163739_0032 (PI HR-400-S1-BBF/ H/ D-5.0-Z SP)
14 Number of Scans	391
15 Receiver Gain	45.9
16 Relaxation Delay	2.0000
17 Pulse Width	10.0000
18 Presaturation Frequency	
19 Acquisition Time	1.3763
20 Acquisition Date	2021-07-28T15:30:40
21 Modification Date	2021-07-28T15:29:54
22 Class	
23 Spectrometer Frequency	100.62
24 Spectral Width	23809.5
25 Lowest Frequency	-1846.0
26 Nucleus	13C
27 Acquired Size	32768
28 Spectral Size	65536

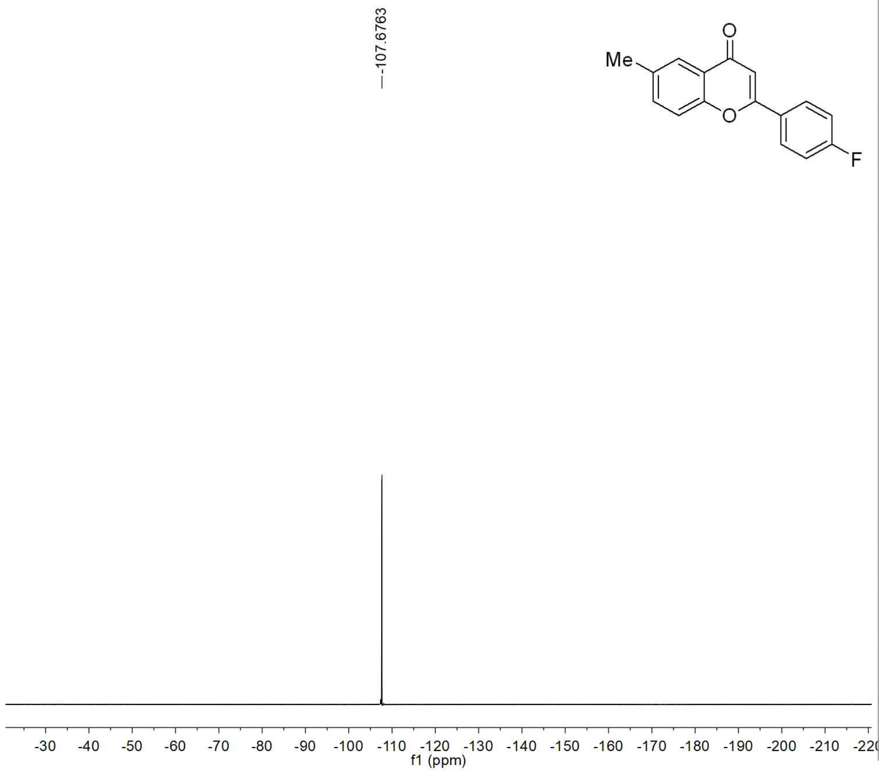

	Parameter	Value
1	Data File Name	E:/ hxw 400/ 2021-1-hxw-F/ 9/ fid
2	Title	2021-1-hxw-F. 9.fid
3	Comment	
4	Origin	Bruker BioSpin GmbH
5	Owner	nmrsu
6	Site	
7	Instrument	Avance
8	Author	
9	Solvent	CDCl3
10	Temperature	297.2
11	Pulse Sequence	zgig
12	Experiment	1D
13	Probe	Z163739_0032 (PI HR-400-S1-BBF/ H/ D-5.0-Z SP)
14	Number of Scans	16
15	Receiver Gain	101.0
16	Relaxation Delay	1.0000
17	Pulse Width	18.0000
18	Presaturation Frequency	
19	Acquisition Time	0.7209
20	Acquisition Date	2021-07-30T09:03:13
21	Modification Date	2021-07-30T09:03:10
22	Class	
23	Spectrometer Frequency	376.46
24	Spectral Width	90909.1
25	Lowest Frequency	-83104.4
26	Nucleus	19F
27	Acquired Size	65536
28	Spectral Size	131072

2-(4-Chlorophenyl)-6-methyl-4*H*-chromen-4-one (2be)

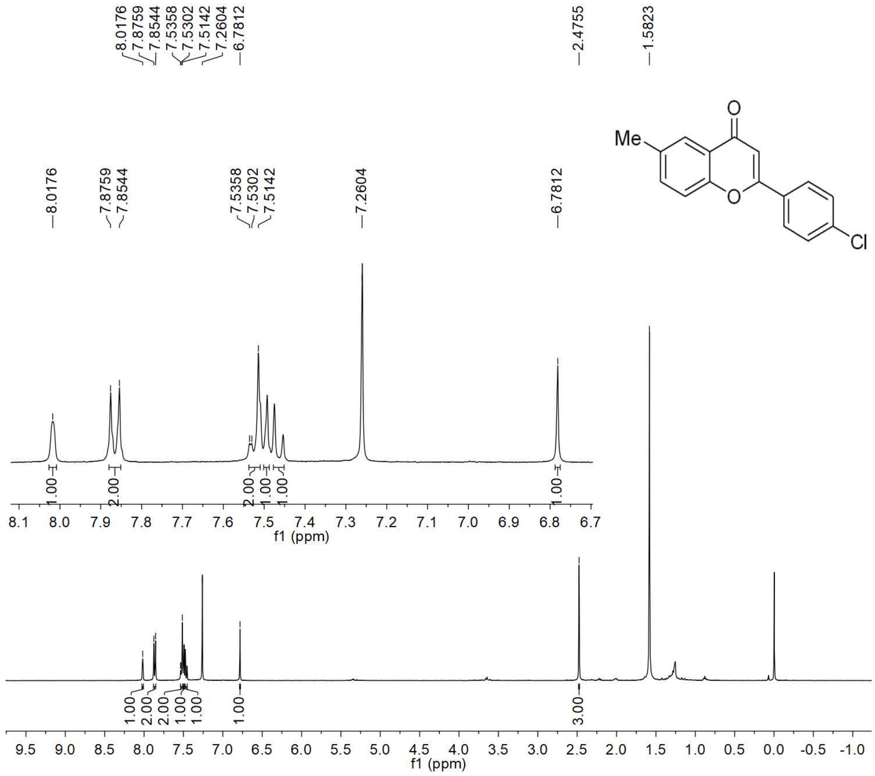

Parameter	Value
1 Data File Name	E:/ hxw 400/ 2021-1-hxw-H/ 106/ fid
2 Title	2021-1-hxw-H. 106.fid
3 Comment	
4 Origin	Bruker BioSpin GmbH
5 Owner	nmrsu
6 Site	
7 Instrument	Avance
8 Author	
9 Solvent	CDCl3
10 Temperature	298.4
11 Pulse Sequence	zg30
12 Experiment	1D
13 Probe	Z163739_0032 (PI HR-400-S1-BBF/ H/ D-5.0-Z SP)
14 Number of Scans	4
15 Receiver Gain	101.0
16 Relaxation Delay	1.0000
17 Pulse Width	10.0000
18 Presaturation Frequency	
19 Acquisition Time	3.9977
20 Acquisition Date	2021-07-06T18:48:41
21 Modification Date	2021-07-06T18:48:26
22 Class	
23 Spectrometer Frequency	400.13
24 Spectral Width	8196.7
25 Lowest Frequency	-1637.2
26 Nucleus	1H
27 Acquired Size	32768
28 Spectral Size	65536

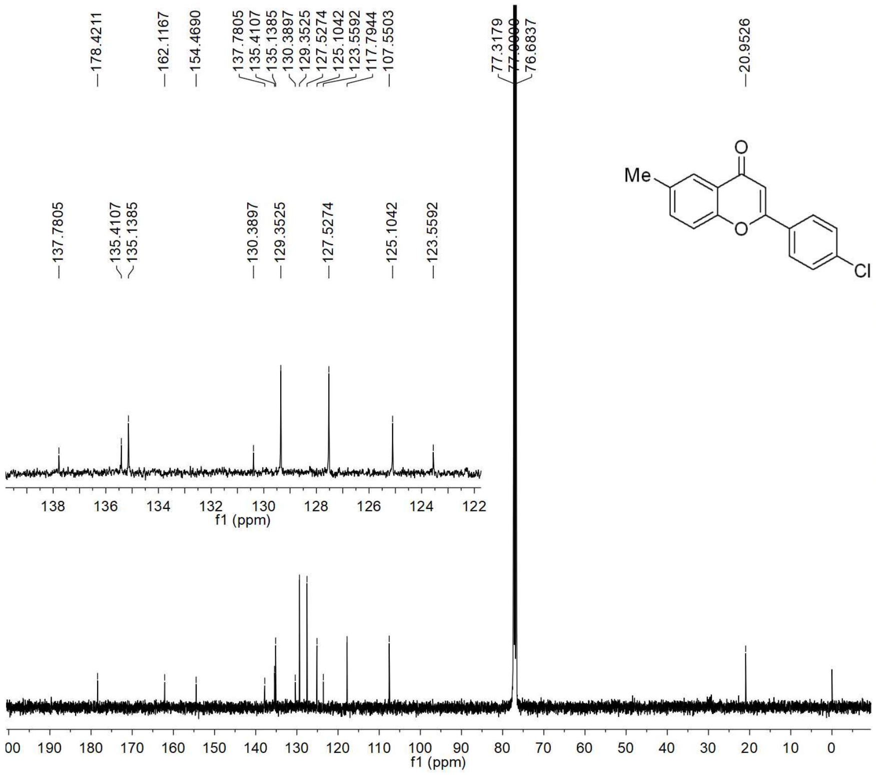

Parameter	Value
1 Data File Name	E:/ hxw 400/ 2021-1-hxw-C/ 38/ fid
2 Title	2021-1-hxw-C. 38.fid
3 Comment	
4 Origin	Bruker BioSpin GmbH
5 Owner	nmrsu
6 Site	
7 Instrument	Avance
8 Author	
9 Solvent	CDCl3
10 Temperature	299.3
11 Pulse Sequence	zgpg30
12 Experiment	1D
13 Probe	Z163739_0032 (PI HR-400-S1-BBF/ H/ D-5.0-Z SP)
14 Number of Scans	3000
15 Receiver Gain	44.6
16 Relaxation Delay	2.0000
17 Pulse Width	10.0000
18 Presaturation Frequency	
19 Acquisition Time	1.3763
20 Acquisition Date	2021-07-10T02:57:59
21 Modification Date	2021-07-10T02:57:26
22 Class	
23 Spectrometer Frequency	100.62
24 Spectral Width	23809.5
25 Lowest Frequency	-1844.6
26 Nucleus	13C
27 Acquired Size	32768
28 Spectral Size	65536

2-(4-Bromophenyl)-6-methyl-4*H*-chromen-4-one (2bf)

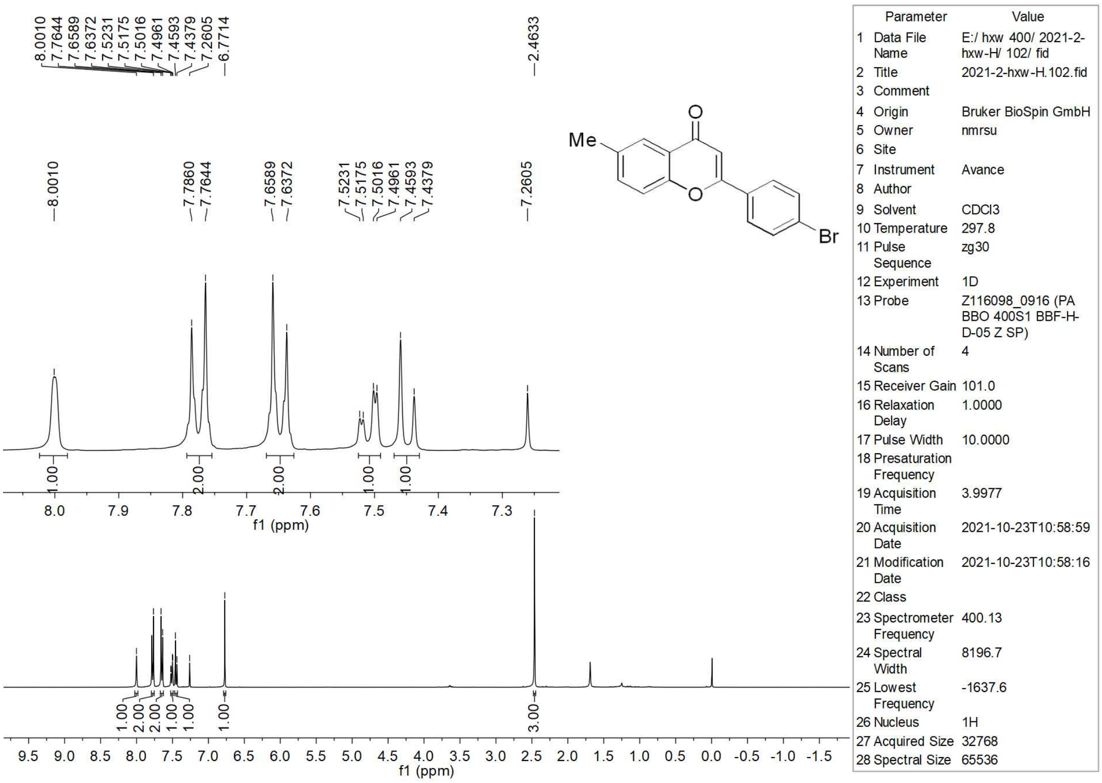

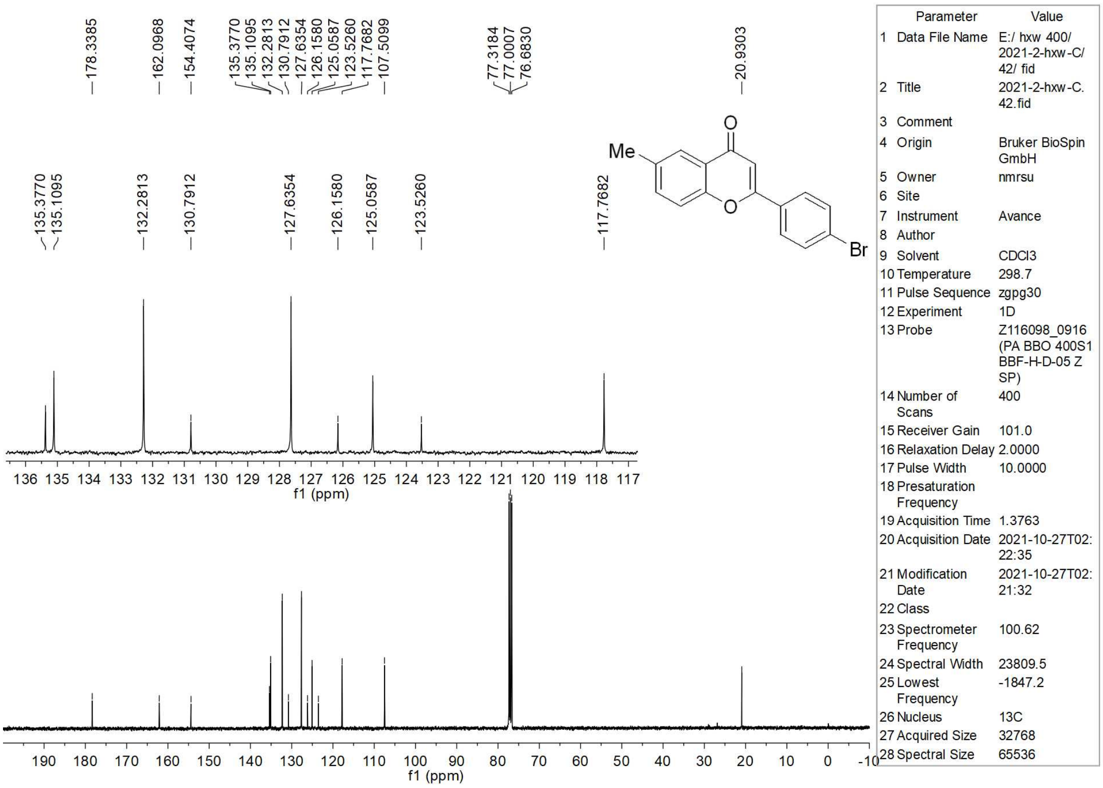

6-Methyl-2-(4'-propyl-[1,1'-biphenyl]-4-yl)-4*H*-chromen-4-one (2bo)

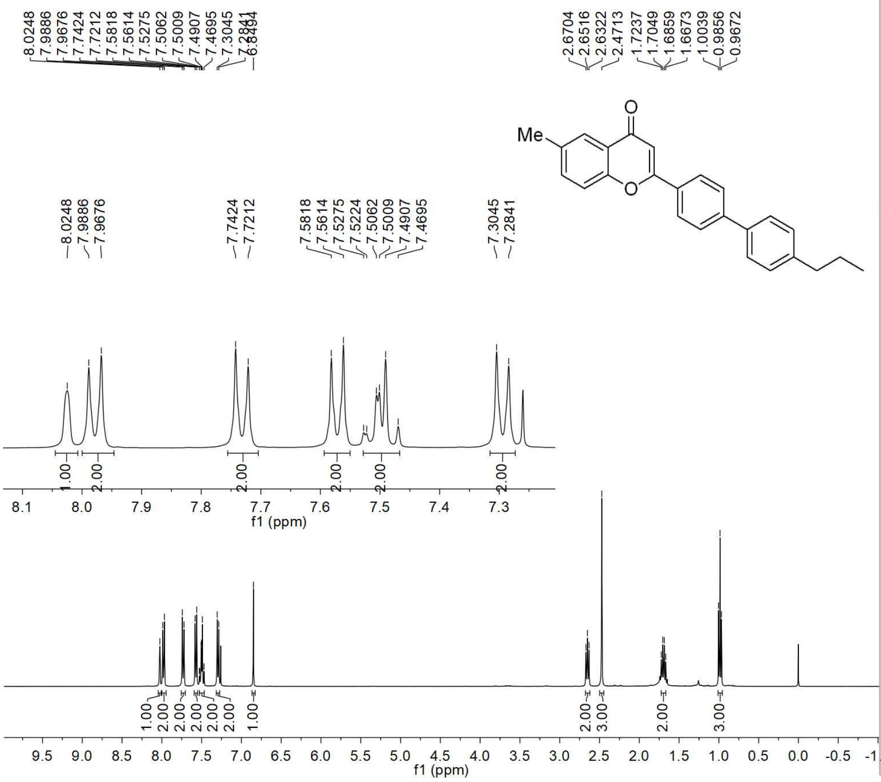

	Parameter	Value
1	Data File Name	E:/ hxw 400/ 2021-2-hxw-H/ 78/ fid
2	Title	2021-2-hxw-H. 78.fid
3	Comment	
4	Origin	Bruker BioSpin GmbH
5	Owner	nmrsu
6	Site	
7	Instrument	Avance
8	Author	
9	Solvent	CDCl3
10	Temperature	297.5
11	Pulse Sequence	zg30
12	Experiment	1D
13	Probe	Z116098_0916 (PA BBO 400S1 BBF-H-D-05 Z SP)
14	Number of Scans	4
15	Receiver Gain	101.0
16	Relaxation Delay	1.0000
17	Pulse Width	10.0000
18	Presaturation Frequency	
19	Acquisition Time	3.9977
20	Acquisition Date	2021-10-11T19:11:39
21	Modification Date	2021-10-11T19:09:36
22	Class	
23	Spectrometer Frequency	400.13
24	Spectral Width	8196.7
25	Lowest Frequency	-1637.6
26	Nucleus	1H
27	Acquired Size	32768
28	Spectral Size	65536

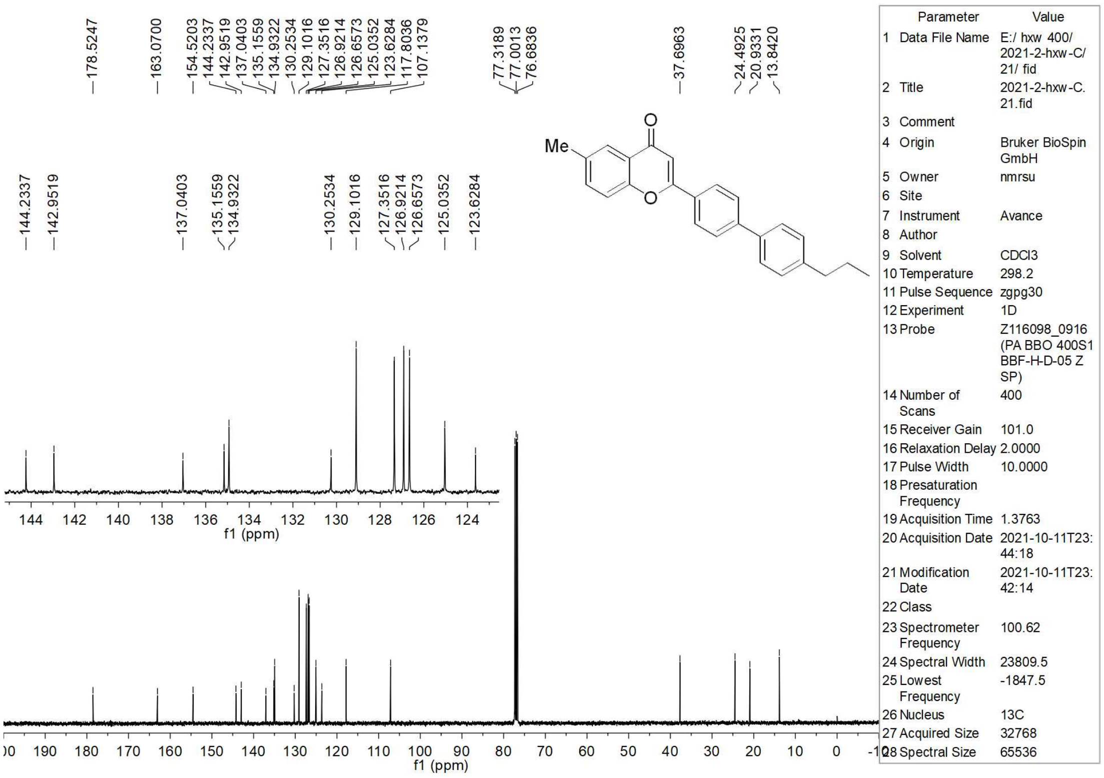

	Parameter	Value
1	Data File Name	E:/ hxw 400/ 2021-2-hxw-C/ 21/ fid
2	Title	2021-2-hxw-C. 21.fid
3	Comment	
4	Origin	Bruker BioSpin GmbH
5	Owner	nmrsu
6	Site	
7	Instrument	Avance
8	Author	
9	Solvent	CDCl3
10	Temperature	298.2
11	Pulse Sequence	zgpg30
12	Experiment	1D
13	Probe	Z116098_0916 (PA BBO 400S1 BBF-H-D-05 Z SP)
14	Number of Scans	400
15	Receiver Gain	101.0
16	Relaxation Delay	2.0000
17	Pulse Width	10.0000
18	Presaturation Frequency	
19	Acquisition Time	1.3763
20	Acquisition Date	2021-10-11T23:44:18
21	Modification Date	2021-10-11T23:42:14
22	Class	
23	Spectrometer Frequency	100.62
24	Spectral Width	23809.5
25	Lowest Frequency	-1847.5
26	Nucleus	13C
27	Acquired Size	32768
28	Spectral Size	65536

6-Methoxy-2-(*p*-tolyl)-4*H*-chromen-4-one (2cb)

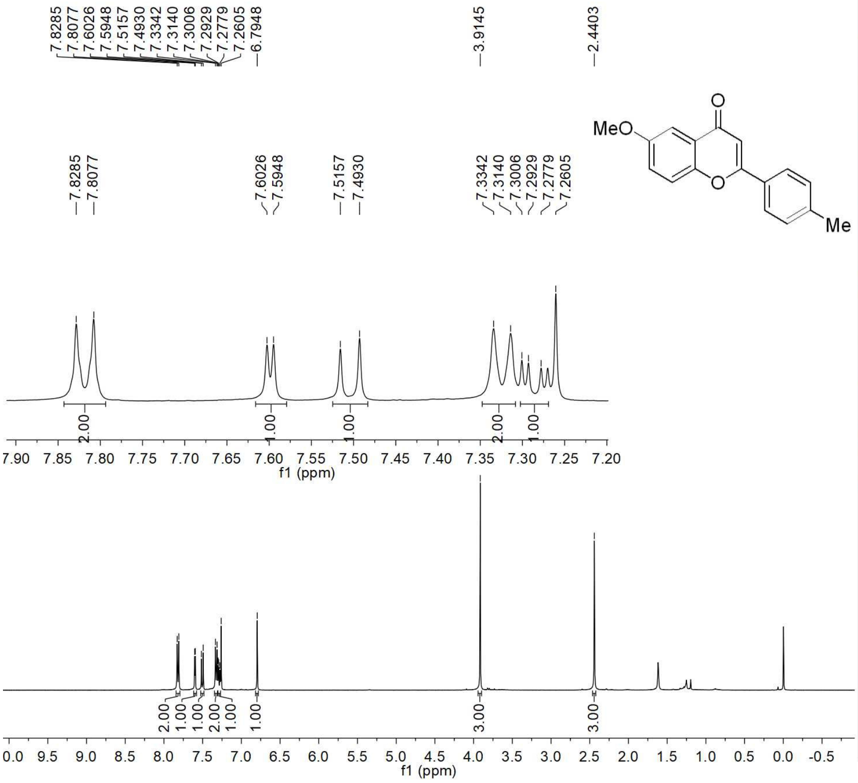

Parameter		Value
1	Data File Name	E:/ hxw 400/ 2021-2-hxw-H/ 142/ fid
2	Title	2021-2-hxw-H.142.fid
3	Comment	
4	Origin	Bruker BioSpin GmbH
5	Owner	nmrsu
6	Site	
7	Instrument	Avance
8	Author	
9	Solvent	CDCl3
10	Temperature	295.4
11	Pulse Sequence	zg30
12	Experiment	1D
13	Probe	Z116098_0916 (PA BBO 400S1 BBF-H-D-05 Z SP)
14	Number of Scans	4
15	Receiver Gain	101.0
16	Relaxation Delay	1.0000
17	Pulse Width	10.0000
18	Presaturation Frequency	
19	Acquisition Time	3.9977
20	Acquisition Date	2021-11-11T21:19:47
21	Modification Date	2021-11-11T21:18:34
22	Class	
23	Spectrometer Frequency	400.13
24	Spectral Width	8196.7
25	Lowest Frequency	-1637.6
26	Nucleus	1H
27	Acquired Size	32768
28	Spectral Size	65536

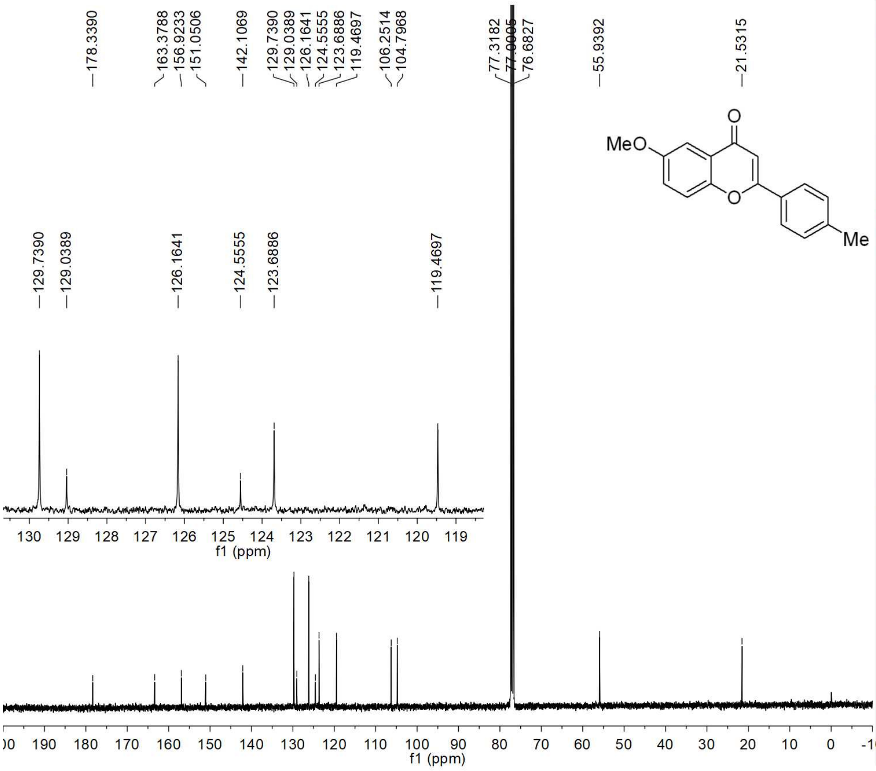

Parameter		Value
1	Data File Name	E:/ hxw 400/ 2021-2-hxw-C/ 39/ fid
2	Title	2021-2-hxw-C.39.fid
3	Comment	
4	Origin	Bruker BioSpin GmbH
5	Owner	nmrsu
6	Site	
7	Instrument	Avance
8	Author	
9	Solvent	CDCl3
10	Temperature	296.2
11	Pulse Sequence	zgpg30
12	Experiment	1D
13	Probe	Z116098_0916 (PA BBO 400S1 BBF-H-D-05 Z SP)
14	Number of Scans	800
15	Receiver Gain	101.0
16	Relaxation Delay	2.0000
17	Pulse Width	10.0000
18	Presaturation Frequency	
19	Acquisition Time	1.3763
20	Acquisition Date	2021-11-11T23:49:49
21	Modification Date	2021-11-11T23:48:36
22	Class	
23	Spectrometer Frequency	100.62
24	Spectral Width	23809.5
25	Lowest Frequency	-1846.5
26	Nucleus	13C
27	Acquired Size	32768
28	Spectral Size	65536

6-Methoxy-2-(4-methoxyphenyl)-4*H*-chromen-4-one (2cc)

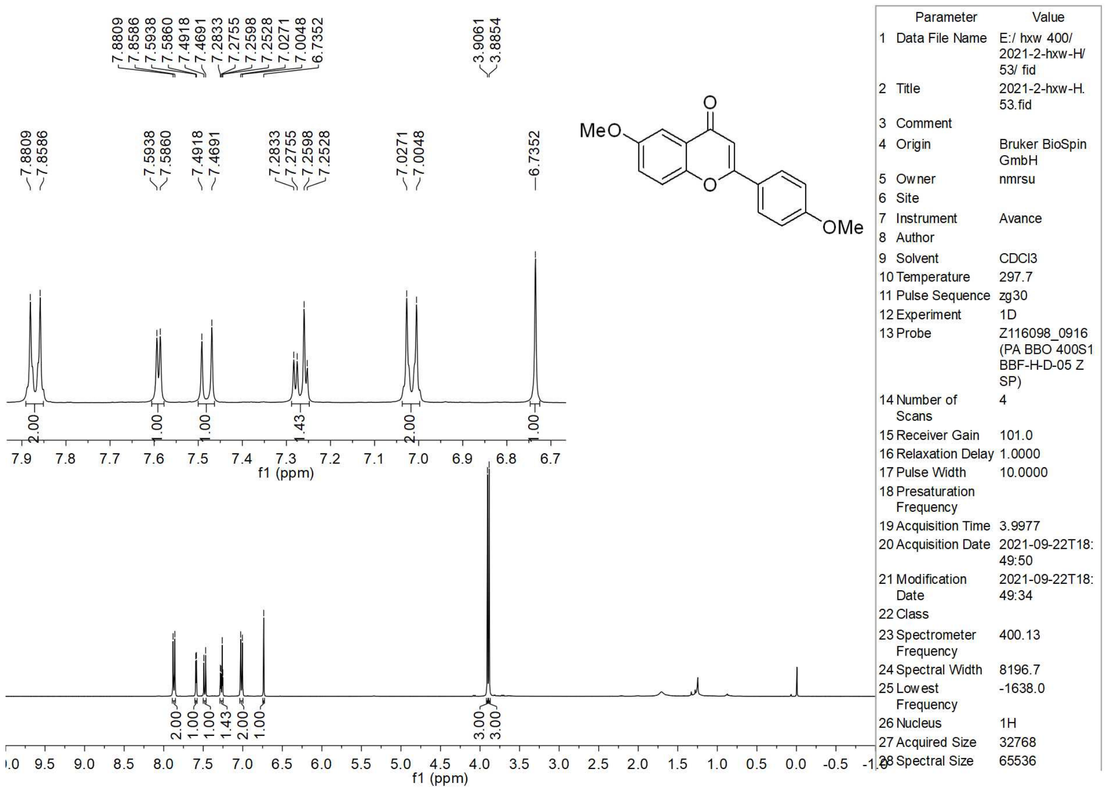

Parameter	Value
1 Data File Name	E:/ hxw 400/ 2021-2-hxw-H/ 53/ fid
2 Title	2021-2-hxw-H. 53.fid
3 Comment	
4 Origin	Bruker BioSpin GmbH
5 Owner	nmrsu
6 Site	
7 Instrument	Avance
8 Author	
9 Solvent	CDCl3
10 Temperature	297.7
11 Pulse Sequence	zg30
12 Experiment	1D
13 Probe	Z116098_0916 (PA BBO 400S1 BBF-H-D-05 Z SP)
14 Number of Scans	4
15 Receiver Gain	101.0
16 Relaxation Delay	1.0000
17 Pulse Width	10.0000
18 Presaturation Frequency	
19 Acquisition Time	3.9977
20 Acquisition Date	2021-09-22T18:49:50
21 Modification Date	2021-09-22T18:49:34
22 Class	
23 Spectrometer Frequency	400.13
24 Spectral Width	8196.7
25 Lowest Frequency	-1638.0
26 Nucleus	1H
27 Acquired Size	32768
28 Spectral Size	65536

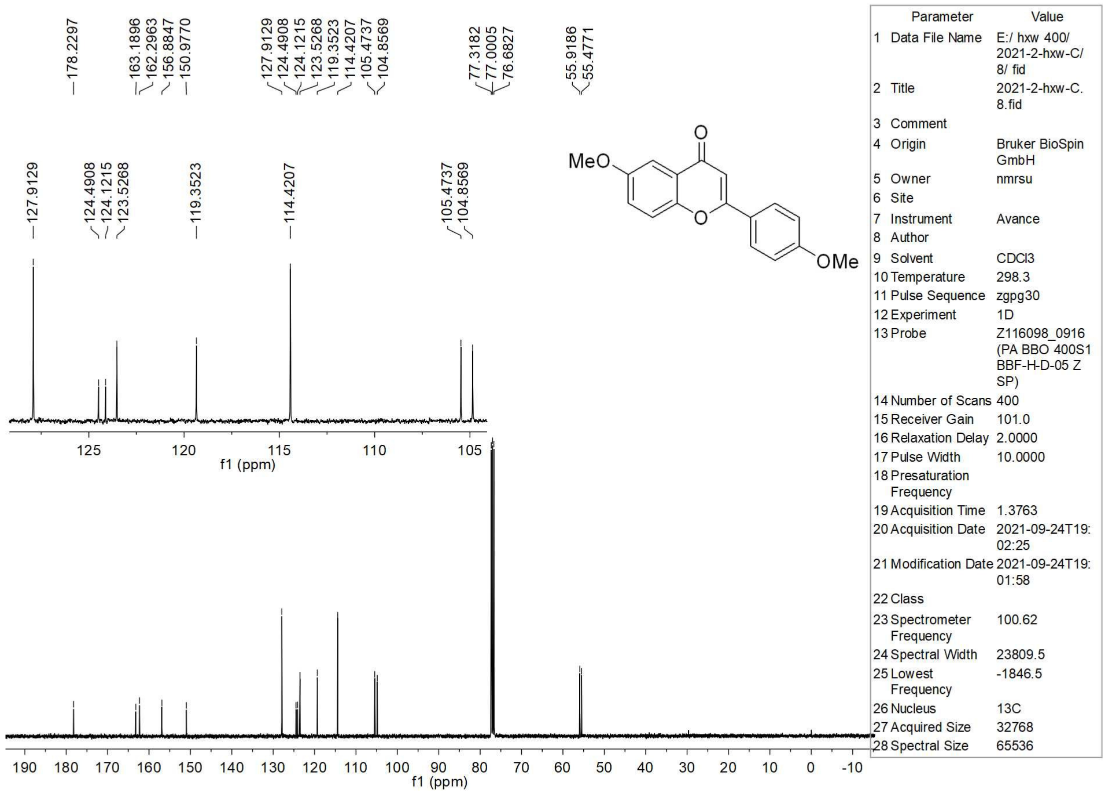

Parameter	Value
1 Data File Name	E:/ hxw 400/ 2021-2-hxw-C/ 8/ fid
2 Title	2021-2-hxw-C. 8.fid
3 Comment	
4 Origin	Bruker BioSpin GmbH
5 Owner	nmrsu
6 Site	
7 Instrument	Avance
8 Author	
9 Solvent	CDCl3
10 Temperature	298.3
11 Pulse Sequence	zgpg30
12 Experiment	1D
13 Probe	Z116098_0916 (PA BBO 400S1 BBF-H-D-05 Z SP)
14 Number of Scans	400
15 Receiver Gain	101.0
16 Relaxation Delay	2.0000
17 Pulse Width	10.0000
18 Presaturation Frequency	
19 Acquisition Time	1.3763
20 Acquisition Date	2021-09-24T19:02:25
21 Modification Date	2021-09-24T19:01:58
22 Class	
23 Spectrometer Frequency	100.62
24 Spectral Width	23809.5
25 Lowest Frequency	-1846.5
26 Nucleus	13C
27 Acquired Size	32768
28 Spectral Size	65536

2-(4-Fluorophenyl)-6-methoxy-4*H*-chromen-4-one (2cd)

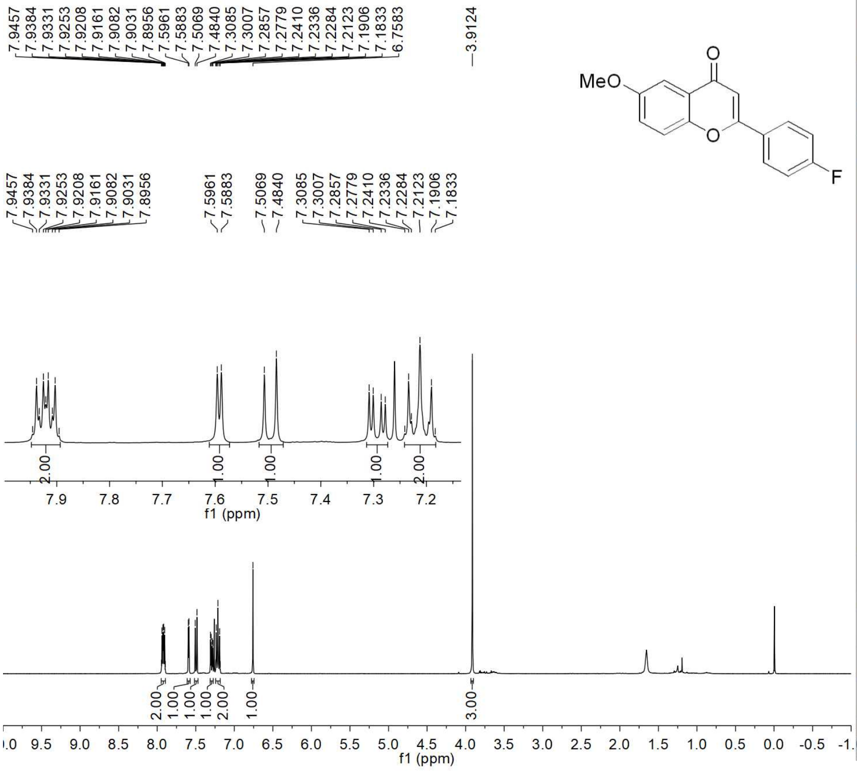

Parameter		Value
1	Data File Name	E:/ hxw 400/ 2021-2-hxw-H/ 74/ fid
2	Title	2021-2-hxw-H.74.fid
3	Comment	
4	Origin	Bruker BioSpin GmbH
5	Owner	nmrsu
6	Site	
7	Instrument	Avance
8	Author	
9	Solvent	CDCl3
10	Temperature	297.5
11	Pulse Sequence	zg30
12	Experiment	1D
13	Probe	Z116098_0916 (PA BBO 400S1 BBF-H-D-05 Z SP)
14	Number of Scans	4
15	Receiver Gain	101.0
16	Relaxation Delay	1.0000
17	Pulse Width	10.0000
18	Presaturation Frequency	
19	Acquisition Time	3.9977
20	Acquisition Date	2021-10-11T19:07:40
21	Modification Date	2021-10-11T19:05:38
22	Class	
23	Spectrometer Frequency	400.13
24	Spectral Width	8196.7
25	Lowest Frequency	-1637.6
26	Nucleus	1H
27	Acquired Size	32768
28	Spectral Size	65536

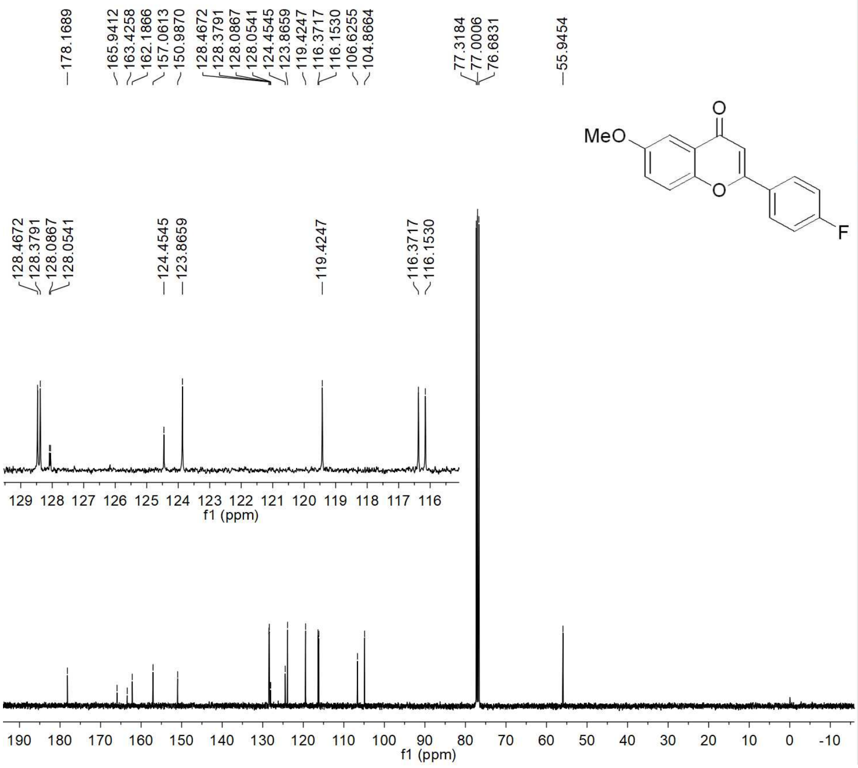

Parameter		Value
1	Data File Name	E:/ hxw 400/ 2021-2-hxw-C/ 20/ fid
2	Title	2021-2-hxw-C.20.fid
3	Comment	
4	Origin	Bruker BioSpin GmbH
5	Owner	nmrsu
6	Site	
7	Instrument	Avance
8	Author	
9	Solvent	CDCl3
10	Temperature	298.2
11	Pulse Sequence	zgpg30
12	Experiment	1D
13	Probe	Z116098_0916 (PA BBO 400S1 BBF-H-D-05 Z SP)
14	Number of Scans	400
15	Receiver Gain	101.0
16	Relaxation Delay	2.0000
17	Pulse Width	10.0000
18	Presaturation Frequency	
19	Acquisition Time	1.3763
20	Acquisition Date	2021-10-11T23:17:06
21	Modification Date	2021-10-11T23:15:02
22	Class	
23	Spectrometer Frequency	100.62
24	Spectral Width	23809.5
25	Lowest Frequency	-1846.1
26	Nucleus	13C
27	Acquired Size	32768
28	Spectral Size	65536

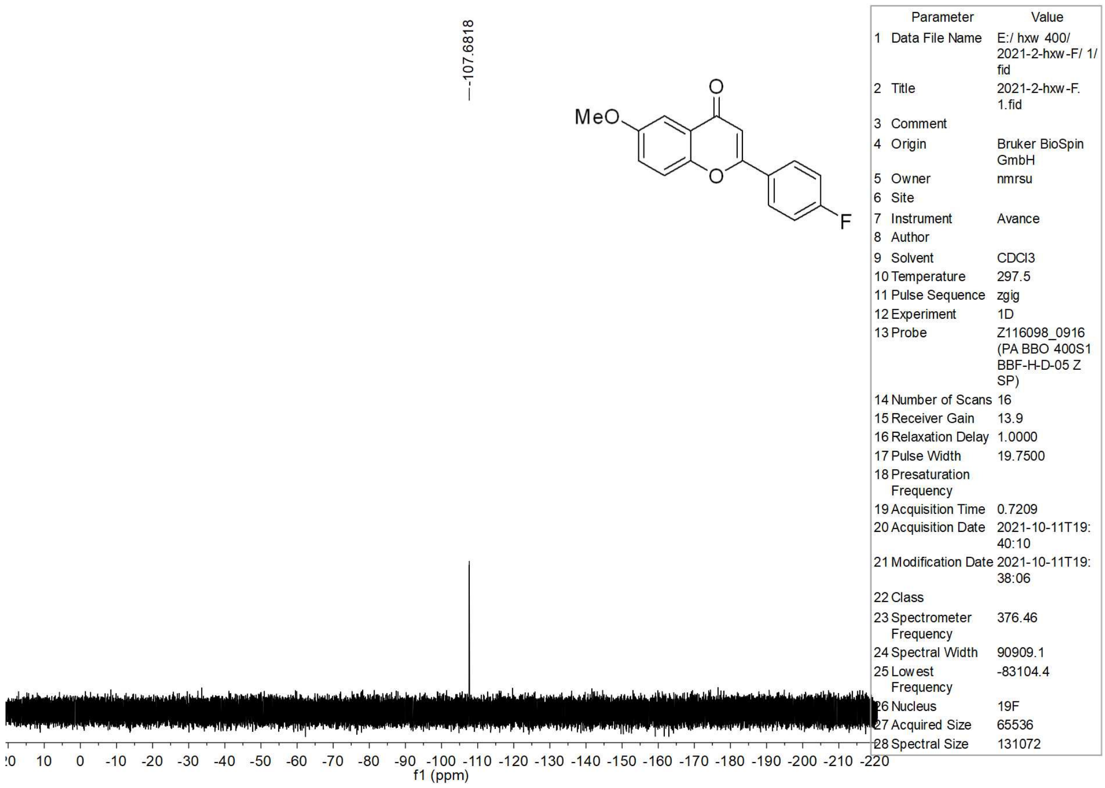

	Parameter	Value
1	Data File Name	E:/ hxw 400/ 2021-2-hxw-F/ 1/ fid
2	Title	2021-2-hxw-F. 1.fid
3	Comment	
4	Origin	Bruker BioSpin GmbH
5	Owner	nmrsu
6	Site	
7	Instrument	Avance
8	Author	
9	Solvent	CDCl3
10	Temperature	297.5
11	Pulse Sequence	zgig
12	Experiment	1D
13	Probe	Z116098_0916 (PA BBO 400S1 BBF-H-D-05 Z SP)
14	Number of Scans	16
15	Receiver Gain	13.9
16	Relaxation Delay	1.0000
17	Pulse Width	19.7500
18	Presaturation Frequency	
19	Acquisition Time	0.7209
20	Acquisition Date	2021-10-11T19:40:10
21	Modification Date	2021-10-11T19:38:06
22	Class	
23	Spectrometer Frequency	376.46
24	Spectral Width	90909.1
25	Lowest Frequency	-83104.4
26	Nucleus	19F
27	Acquired Size	65536
28	Spectral Size	131072

2-(4-Chlorophenyl)-6-methoxy-4*H*-chromen-4-one (2ce)

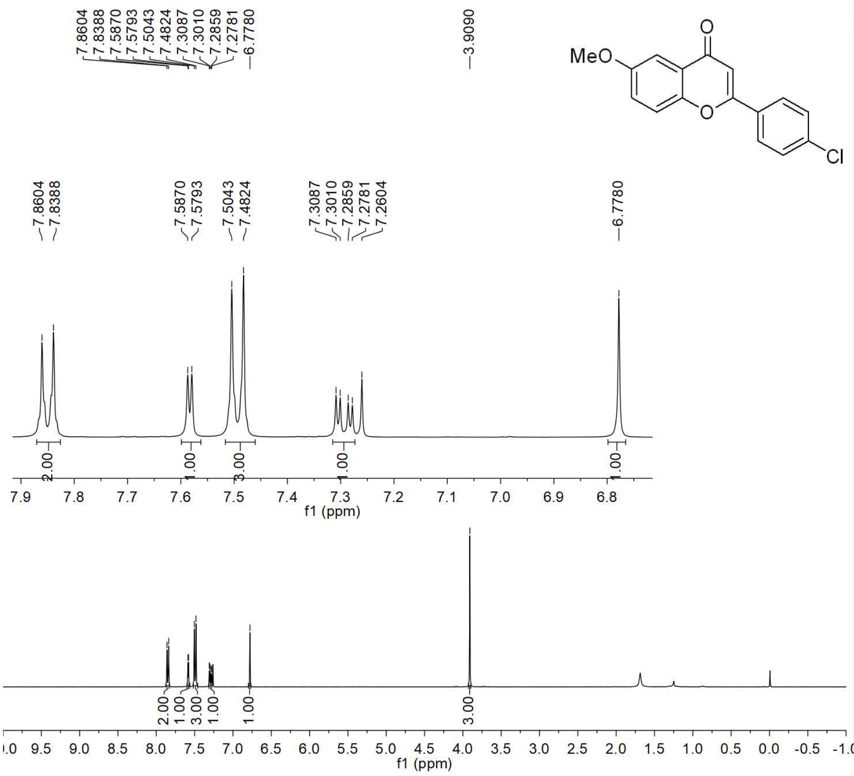

	Parameter	Value
1	Data File Name	E:/ hxw 400/ 2021-1-hxw-H/ 142/ fid
2	Title	2021-1-hxw-H.142.fid
3	Comment	
4	Origin	Bruker BioSpin GmbH
5	Owner	nmrsu
6	Site	
7	Instrument	Avance
8	Author	
9	Solvent	CDCl3
10	Temperature	297.0
11	Pulse Sequence	zg30
12	Experiment	1D
13	Probe	Z163739_0032 (PI HR-400-S1-BBF/ H/ D-5.0-Z SP)
14	Number of Scans	4
15	Receiver Gain	101.0
16	Relaxation Delay	1.0000
17	Pulse Width	10.0000
18	Presaturation Frequency	
19	Acquisition Time	3.9977
20	Acquisition Date	2021-07-26T18:24:09
21	Modification Date	2021-07-26T18:23:34
22	Class	
23	Spectrometer Frequency	400.13
24	Spectral Width	8196.7
25	Lowest Frequency	-1637.2
26	Nucleus	1H
27	Acquired Size	32768
28	Spectral Size	65536

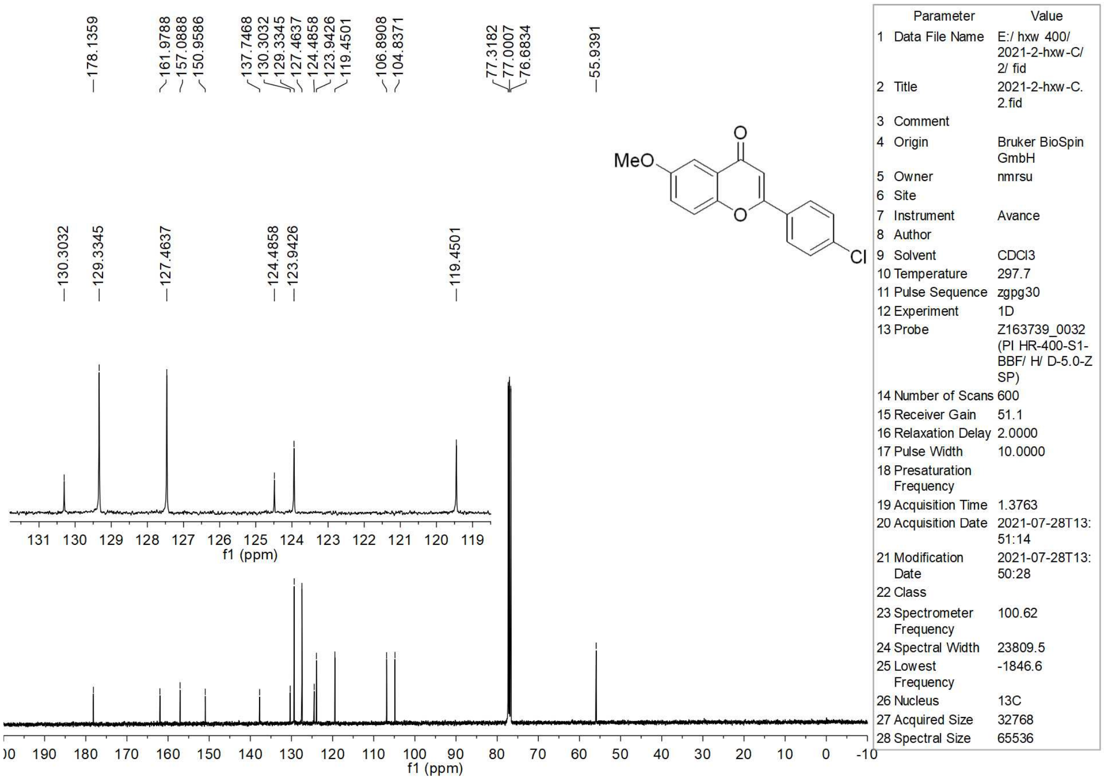

	Parameter	Value
1	Data File Name	E:/ hxw 400/ 2021-2-hxw-C/ 2/ fid
2	Title	2021-2-hxw-C. 2.fid
3	Comment	
4	Origin	Bruker BioSpin GmbH
5	Owner	nmrsu
6	Site	
7	Instrument	Avance
8	Author	
9	Solvent	CDCl3
10	Temperature	297.7
11	Pulse Sequence	zgpg30
12	Experiment	1D
13	Probe	Z163739_0032 (PI HR-400-S1-BBF/ H/ D-5.0-Z SP)
14	Number of Scans	600
15	Receiver Gain	51.1
16	Relaxation Delay	2.0000
17	Pulse Width	10.0000
18	Presaturation Frequency	
19	Acquisition Time	1.3763
20	Acquisition Date	2021-07-28T13:51:14
21	Modification Date	2021-07-28T13:50:28
22	Class	
23	Spectrometer Frequency	100.62
24	Spectral Width	23809.5
25	Lowest Frequency	-1846.6
26	Nucleus	13C
27	Acquired Size	32768
28	Spectral Size	65536

2-(4-Bromophenyl)-6-methoxy-4*H*-chromen-4-one (2cf)

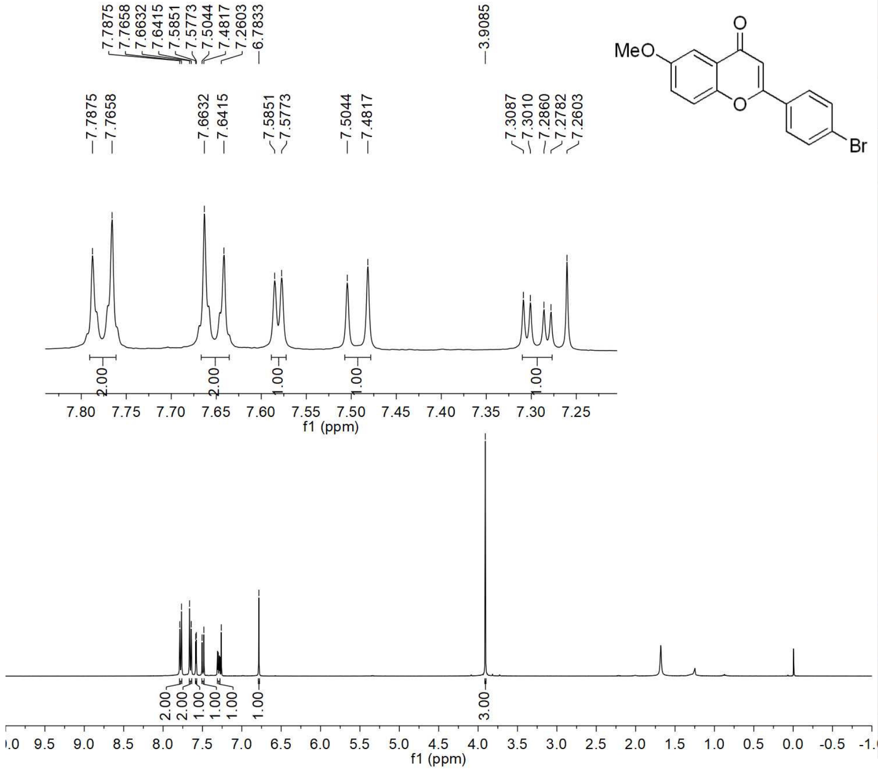

Parameter	Value
1 Data File Name	E:/ hxw 400/ 2021-1-hxw-H/ 139/ fid
2 Title	2021-1-hxw-H. 139.fid
3 Comment	
4 Origin	Bruker BioSpin GmbH
5 Owner	nmrsu
6 Site	
7 Instrument	Avance
8 Author	
9 Solvent	CDCl3
10 Temperature	297.0
11 Pulse Sequence	zg30
12 Experiment	1D
13 Probe	Z163739_0032 (PI HR-400-S1-BBF/ H/ D-5.0-Z SP)
14 Number of Scans	4
15 Receiver Gain	101.0
16 Relaxation Delay	1.0000
17 Pulse Width	10.0000
18 Presaturation Frequency	
19 Acquisition Time	3.9977
20 Acquisition Date	2021-07-26T18:10:11
21 Modification Date	2021-07-26T18:09:36
22 Class	
23 Spectrometer Frequency	400.13
24 Spectral Width	8196.7
25 Lowest Frequency	-1637.2
26 Nucleus	1H
27 Acquired Size	32768
28 Spectral Size	65536

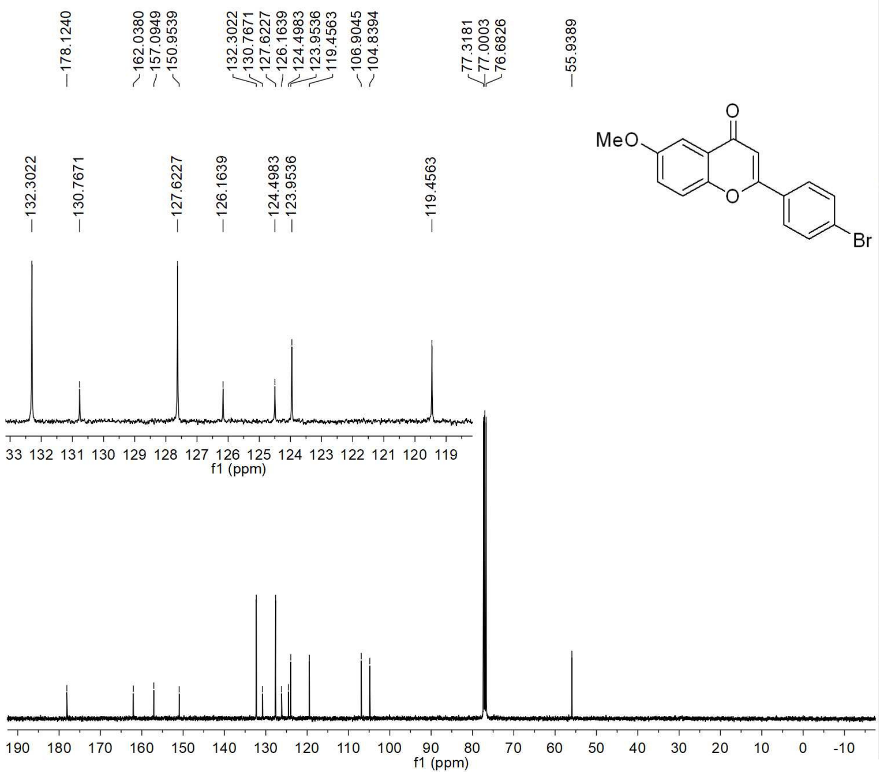

Parameter	Value
1 Data File Name	E:/ hxw 400/ 2021-2-hxw-C/ 3/ fid
2 Title	2021-2-hxw-C. 3.fid
3 Comment	
4 Origin	Bruker BioSpin GmbH
5 Owner	nmrsu
6 Site	
7 Instrument	Avance
8 Author	
9 Solvent	CDCl3
10 Temperature	298.1
11 Pulse Sequence	zgpg30
12 Experiment	1D
13 Probe	Z163739_0032 (PI HR-400-S1-BBF/ H/ D-5.0-Z SP)
14 Number of Scans	600
15 Receiver Gain	45.9
16 Relaxation Delay	2.0000
17 Pulse Width	10.0000
18 Presaturation Frequency	
19 Acquisition Time	1.3763
20 Acquisition Date	2021-07-28T14:31:28
21 Modification Date	2021-07-28T14:30:42
22 Class	
23 Spectrometer Frequency	100.62
24 Spectral Width	23809.5
25 Lowest Frequency	-1846.7
26 Nucleus	13C
27 Acquired Size	32768
28 Spectral Size	65536

6-Chloro-2-(*p*-tolyl)-4*H*-chromen-4-one (2db)

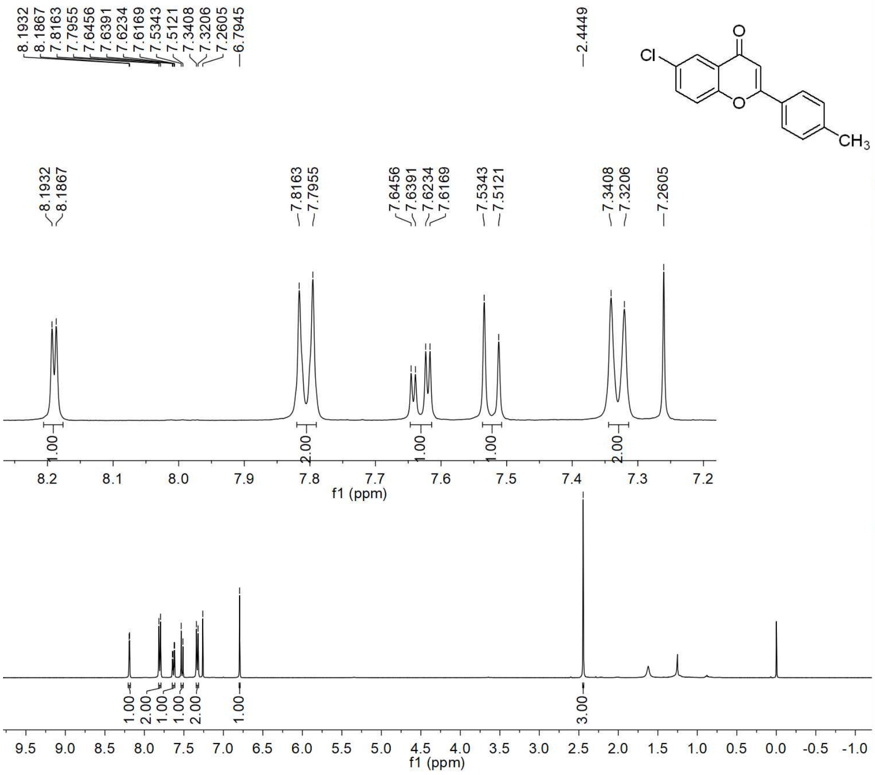

	Parameter	Value
1	Data File Name	E:/ hxw 400/ 2021-2-hxw-H/ 60/ fid
2	Title	2021-2-hxw-H.60.fid
3	Comment	
4	Origin	Bruker BioSpin GmbH
5	Owner	nmrsu
6	Site	
7	Instrument	Avance
8	Author	
9	Solvent	CDCl3
10	Temperature	297.7
11	Pulse Sequence	zg30
12	Experiment	1D
13	Probe	Z116098_0916 (PA BBO 400S1 BBF-H-D-05 Z SP)
14	Number of Scans	4
15	Receiver Gain	101.0
16	Relaxation Delay	1.0000
17	Pulse Width	10.0000
18	Presaturation Frequency	
19	Acquisition Time	3.9977
20	Acquisition Date	2021-09-23T18:42:49
21	Modification Date	2021-09-23T18:42:28
22	Class	
23	Spectrometer Frequency	400.13
24	Spectral Width	8196.7
25	Lowest Frequency	-1637.6
26	Nucleus	1H
27	Acquired Size	32768
28	Spectral Size	65536

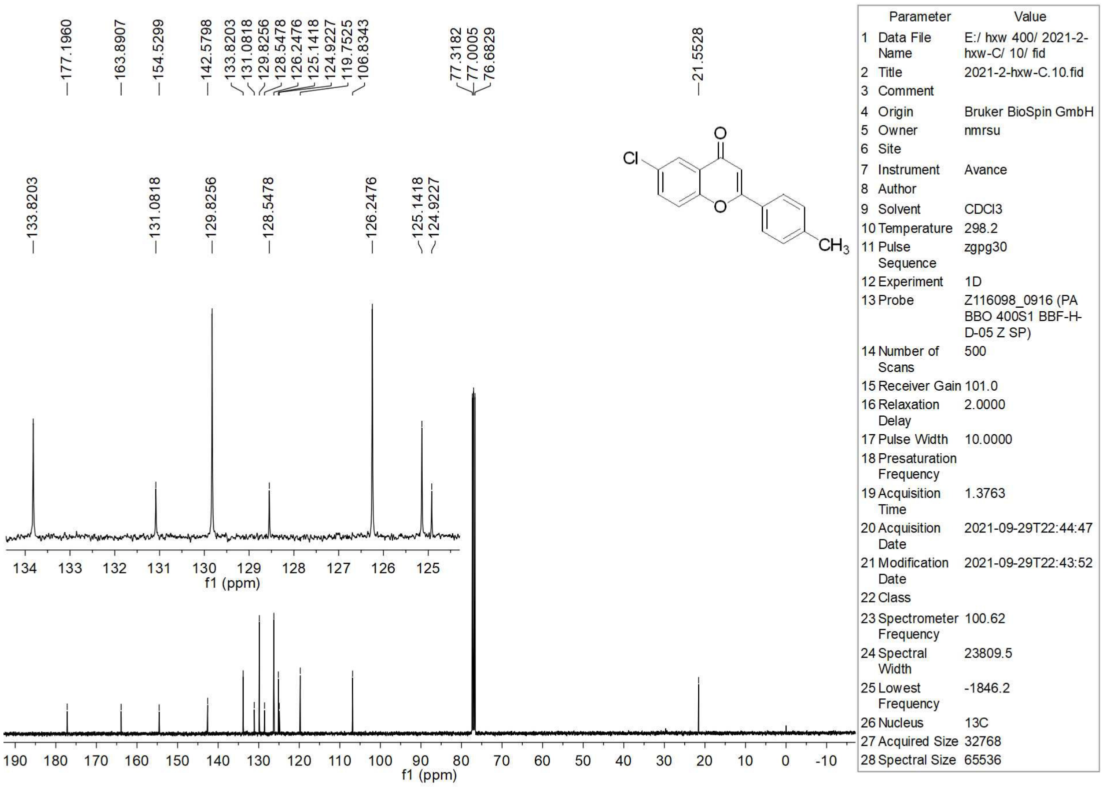

	Parameter	Value
1	Data File Name	E:/ hxw 400/ 2021-2-hxw-C/ 10/ fid
2	Title	2021-2-hxw-C.10.fid
3	Comment	
4	Origin	Bruker BioSpin GmbH
5	Owner	nmrsu
6	Site	
7	Instrument	Avance
8	Author	
9	Solvent	CDCl3
10	Temperature	298.2
11	Pulse Sequence	zgpg30
12	Experiment	1D
13	Probe	Z116098_0916 (PA BBO 400S1 BBF-H-D-05 Z SP)
14	Number of Scans	500
15	Receiver Gain	101.0
16	Relaxation Delay	2.0000
17	Pulse Width	10.0000
18	Presaturation Frequency	
19	Acquisition Time	1.3763
20	Acquisition Date	2021-09-29T22:44:47
21	Modification Date	2021-09-29T22:43:52
22	Class	
23	Spectrometer Frequency	100.62
24	Spectral Width	23809.5
25	Lowest Frequency	-1846.2
26	Nucleus	13C
27	Acquired Size	32768
28	Spectral Size	65536

6-Chloro-2-(4-methoxyphenyl)-4*H*-chromen-4-one (2dc)

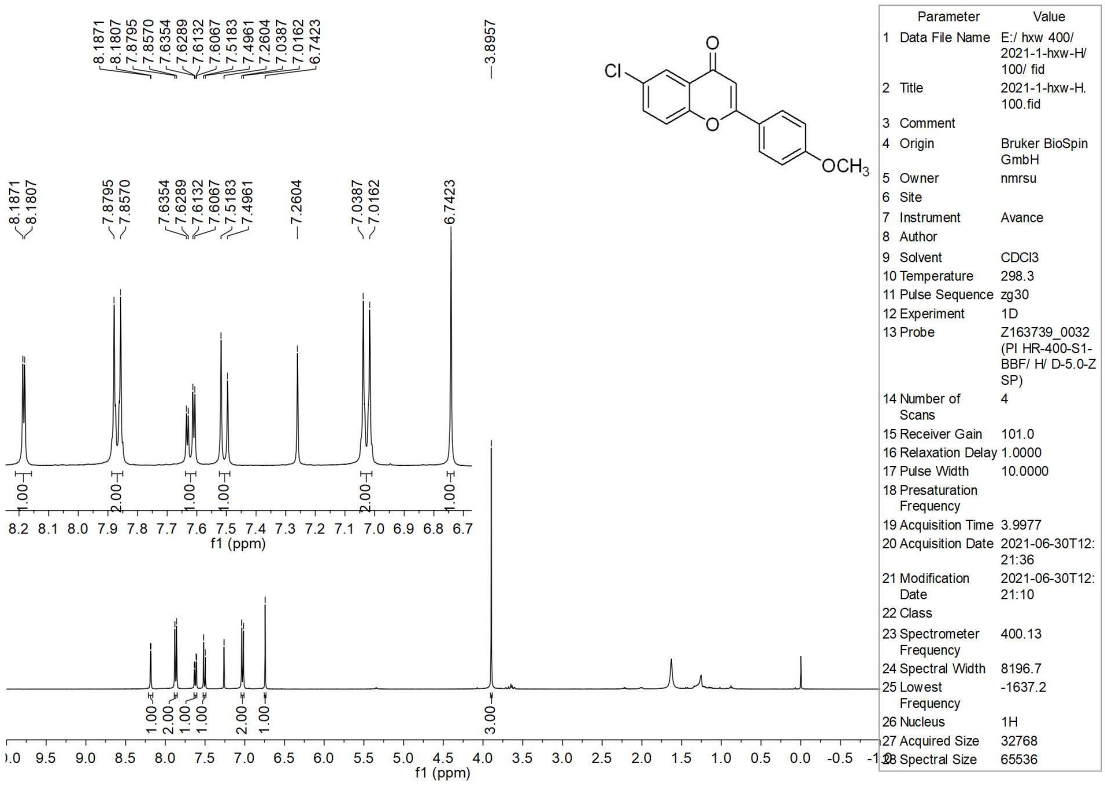

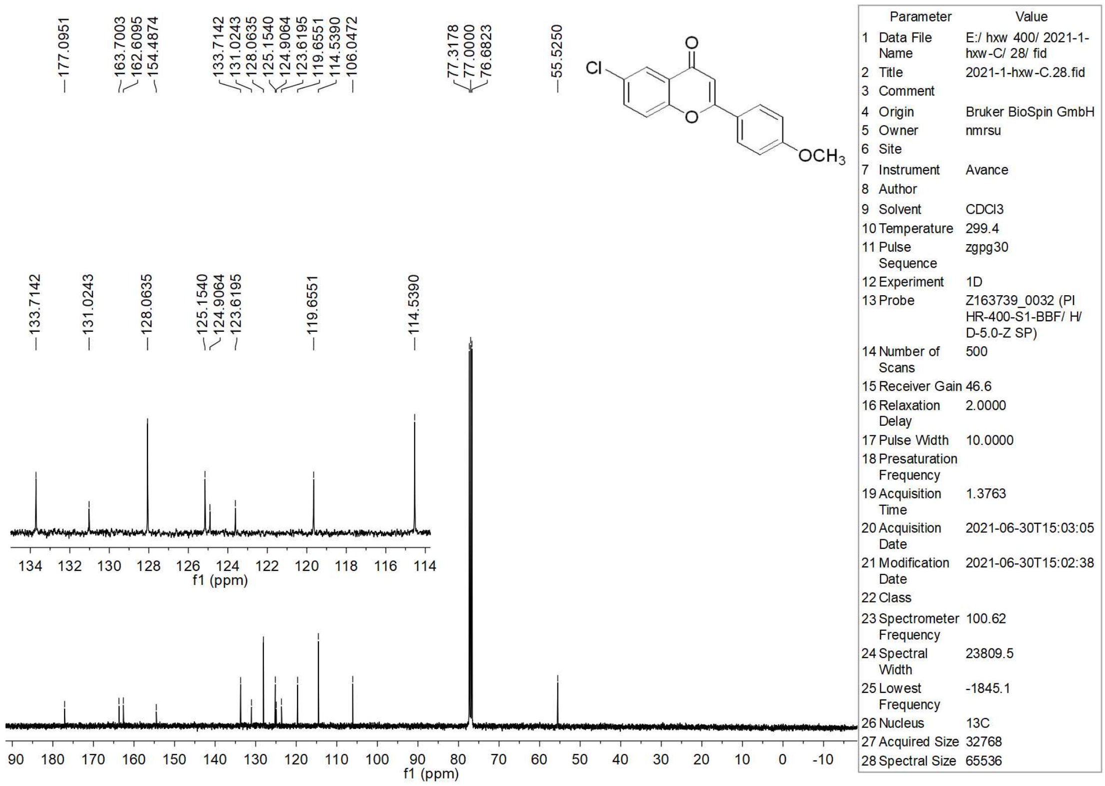

6-Chloro-2-(4-chlorophenyl)-4*H*-chromen-4-one (2de)

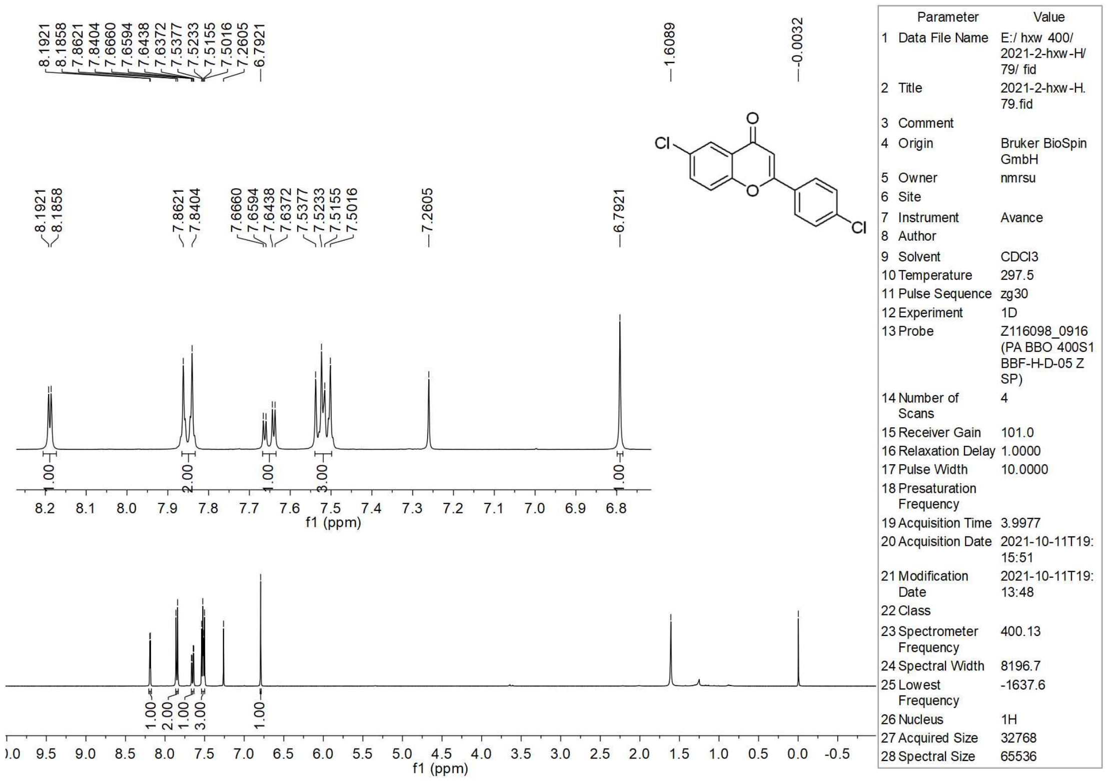

	Parameter	Value
1	Data File Name	E:/ hxw 400/ 2021-2-hxw-H/ 79/ fid
2	Title	2021-2-hxw-H.79.fid
3	Comment	
4	Origin	Bruker BioSpin GmbH
5	Owner	nmrsu
6	Site	
7	Instrument	Avance
8	Author	
9	Solvent	CDCl3
10	Temperature	297.5
11	Pulse Sequence	zg30
12	Experiment	1D
13	Probe	Z116098_0916 (PA BBO 400S1 BBF-H-D-05 Z SP)
14	Number of Scans	4
15	Receiver Gain	101.0
16	Relaxation Delay	1.0000
17	Pulse Width	10.0000
18	Presaturation Frequency	
19	Acquisition Time	3.9977
20	Acquisition Date	2021-10-11T19:15:51
21	Modification Date	2021-10-11T19:13:48
22	Class	
23	Spectrometer Frequency	400.13
24	Spectral Width	8196.7
25	Lowest Frequency	-1637.6
26	Nucleus	1H
27	Acquired Size	32768
28	Spectral Size	65536

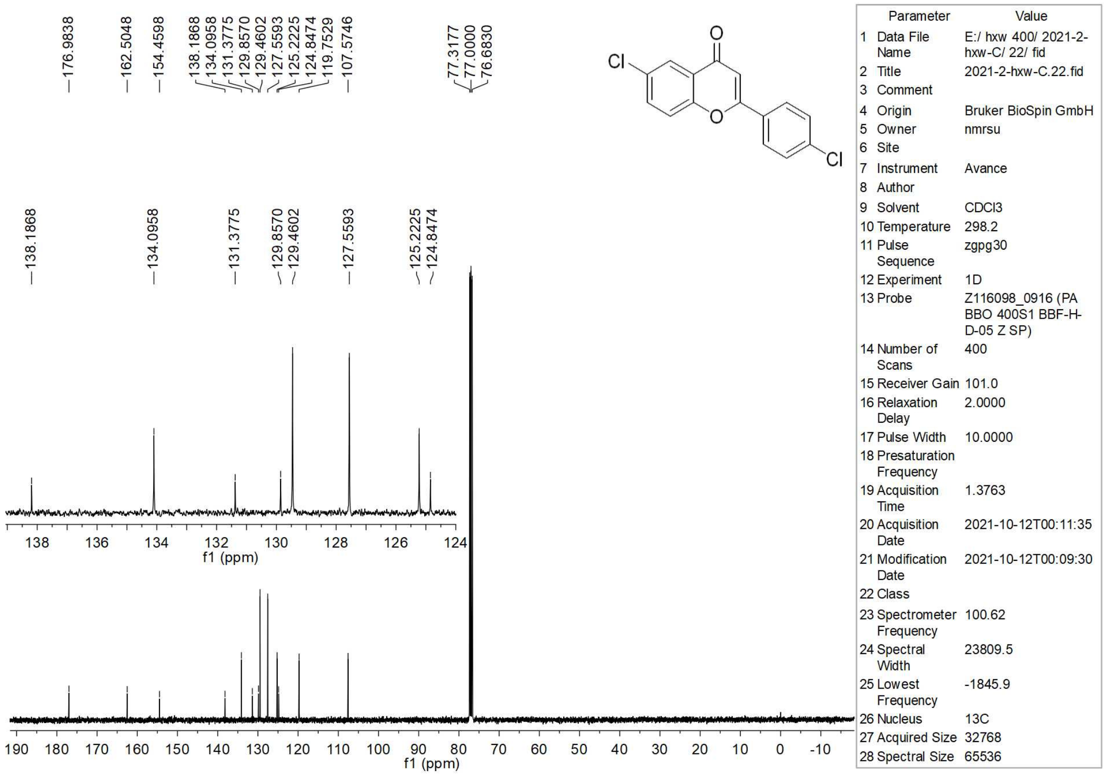

	Parameter	Value
1	Data File Name	E:/ hxw 400/ 2021-2-hxw-C/ 22/ fid
2	Title	2021-2-hxw-C.22.fid
3	Comment	
4	Origin	Bruker BioSpin GmbH
5	Owner	nmrsu
6	Site	
7	Instrument	Avance
8	Author	
9	Solvent	CDCl3
10	Temperature	298.2
11	Pulse Sequence	zgpg30
12	Experiment	1D
13	Probe	Z116098_0916 (PA BBO 400S1 BBF-H-D-05 Z SP)
14	Number of Scans	400
15	Receiver Gain	101.0
16	Relaxation Delay	2.0000
17	Pulse Width	10.0000
18	Presaturation Frequency	
19	Acquisition Time	1.3763
20	Acquisition Date	2021-10-12T00:11:35
21	Modification Date	2021-10-12T00:09:30
22	Class	
23	Spectrometer Frequency	100.62
24	Spectral Width	23809.5
25	Lowest Frequency	-1845.9
26	Nucleus	13C
27	Acquired Size	32768
28	Spectral Size	65536

2-(4-Bromophenyl)-6-chloro-4*H*-chromen-4-one (2df)

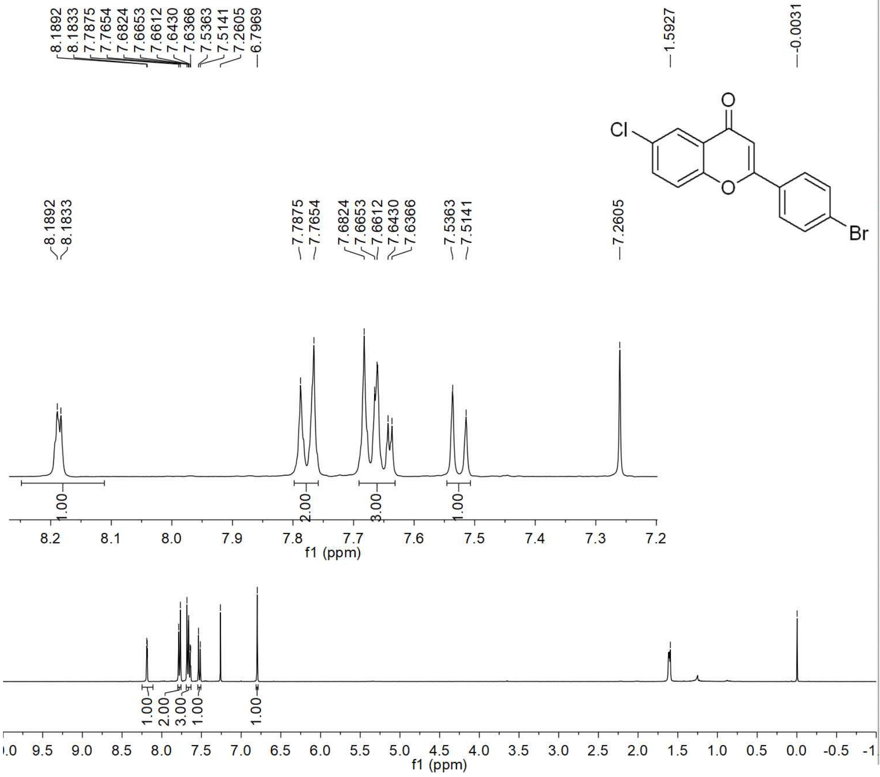

Parameter	Value
1 Data File Name	E:/ hxw 400/ 2021-2-hxw-H/ 80/ fid
2 Title	2021-2-hxw-H. 80.fid
3 Comment	
4 Origin	Bruker BioSpin GmbH
5 Owner	nmrsu
6 Site	
7 Instrument	Avance
8 Author	
9 Solvent	CDCl3
10 Temperature	297.5
11 Pulse Sequence	zg30
12 Experiment	1D
13 Probe	Z116098_0916 (PA BBO 400S1 BBF-H-D-05 Z SP)
14 Number of Scans	4
15 Receiver Gain	101.0
16 Relaxation Delay	1.0000
17 Pulse Width	10.0000
18 Presaturation Frequency	
19 Acquisition Time	3.9977
20 Acquisition Date	2021-10-11T19:19:47
21 Modification Date	2021-10-11T19:17:44
22 Class	
23 Spectrometer Frequency	400.13
24 Spectral Width	8196.7
25 Lowest Frequency	-1637.6
26 Nucleus	1H
27 Acquired Size	32768
28 Spectral Size	65536

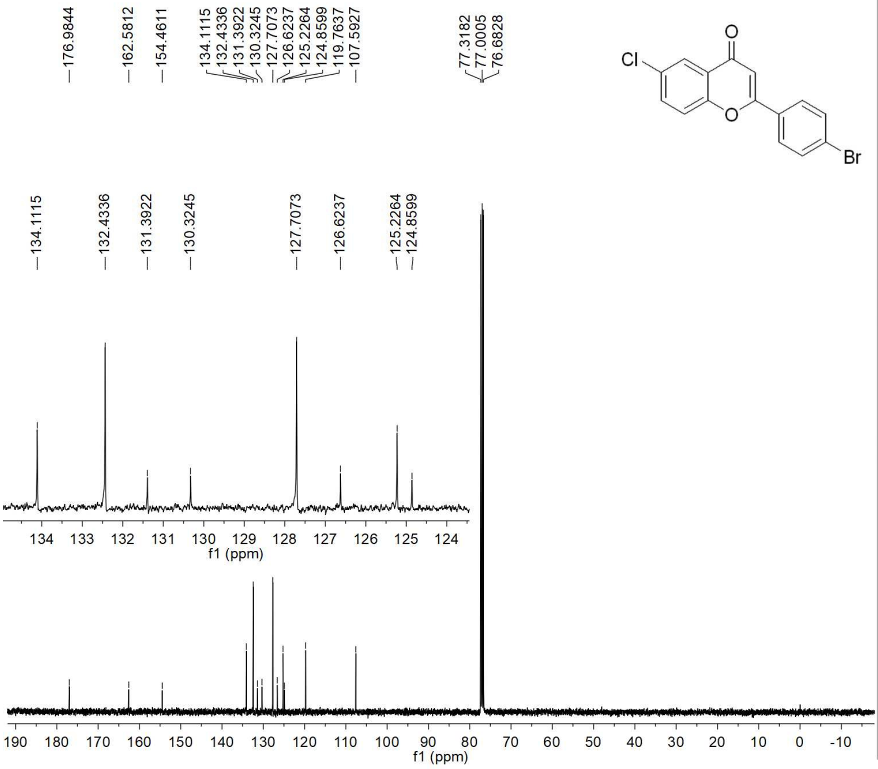

Parameter	Value
1 Data File Name	E:/ hxw 400/ 2021-2-hxw-C/ 23/ fid
2 Title	2021-2-hxw-C. 23.fid
3 Comment	
4 Origin	Bruker BioSpin GmbH
5 Owner	nmrsu
6 Site	
7 Instrument	Avance
8 Author	
9 Solvent	CDCl3
10 Temperature	298.1
11 Pulse Sequence	zgpg30
12 Experiment	1D
13 Probe	Z116098_0916 (PA BBO 400S1 BBF-H-D-05 Z SP)
14 Number of Scans	400
15 Receiver Gain	101.0
16 Relaxation Delay	2.0000
17 Pulse Width	10.0000
18 Presaturation Frequency	
19 Acquisition Time	1.3763
20 Acquisition Date	2021-10-12T00:38:13
21 Modification Date	2021-10-12T00:36:08
22 Class	
23 Spectrometer Frequency	100.62
24 Spectral Width	23809.5
25 Lowest Frequency	-1845.8
26 Nucleus	13C
27 Acquired Size	32768
28 Spectral Size	65536

6-Bromo-2-(*p*-tolyl)-4*H*-chromen-4-one (2eb)

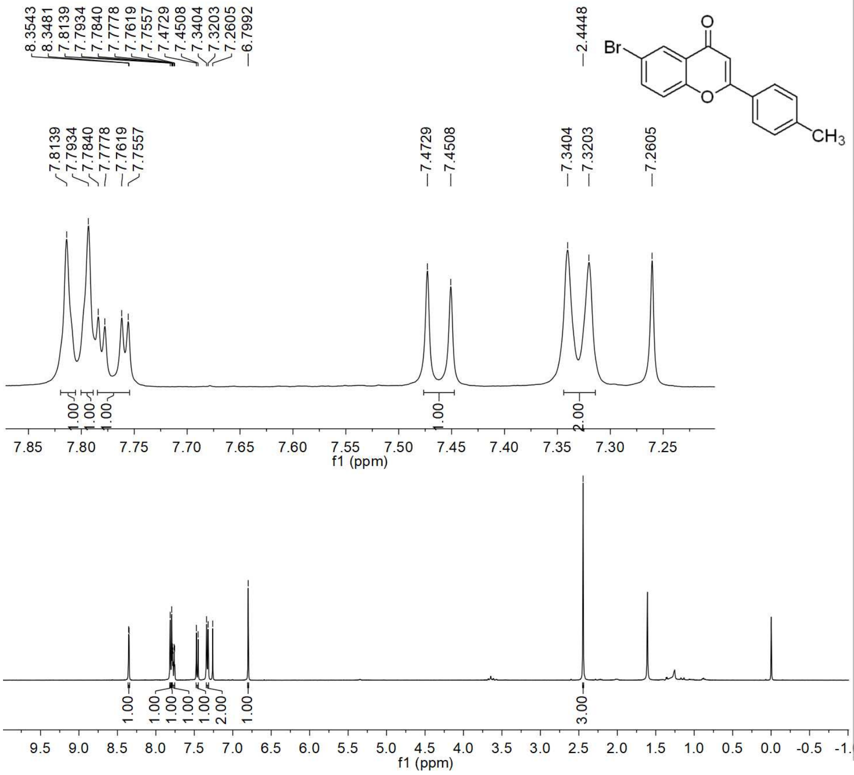

	Parameter	Value
1	Data File Name	E:/ hxw 400/ 2021-2-hxw-H/ 103/ fid
2	Title	2021-2-hxw-H.103.fid
3	Comment	
4	Origin	Bruker BioSpin GmbH
5	Owner	nmrsu
6	Site	
7	Instrument	Avance
8	Author	
9	Solvent	CDCl3
10	Temperature	297.8
11	Pulse Sequence	zg30
12	Experiment	1D
13	Probe	Z116098_0916 (PA BBO 400S1 BBF-H-D-05 Z SP)
14	Number of Scans	4
15	Receiver Gain	101.0
16	Relaxation Delay	1.0000
17	Pulse Width	10.0000
18	Presaturation Frequency	
19	Acquisition Time	3.9977
20	Acquisition Date	2021-10-23T11:02:57
21	Modification Date	2021-10-23T11:02:14
22	Class	
23	Spectrometer Frequency	400.13
24	Spectral Width	8196.7
25	Lowest Frequency	-1637.6
26	Nucleus	1H
27	Acquired Size	32768
28	Spectral Size	65536

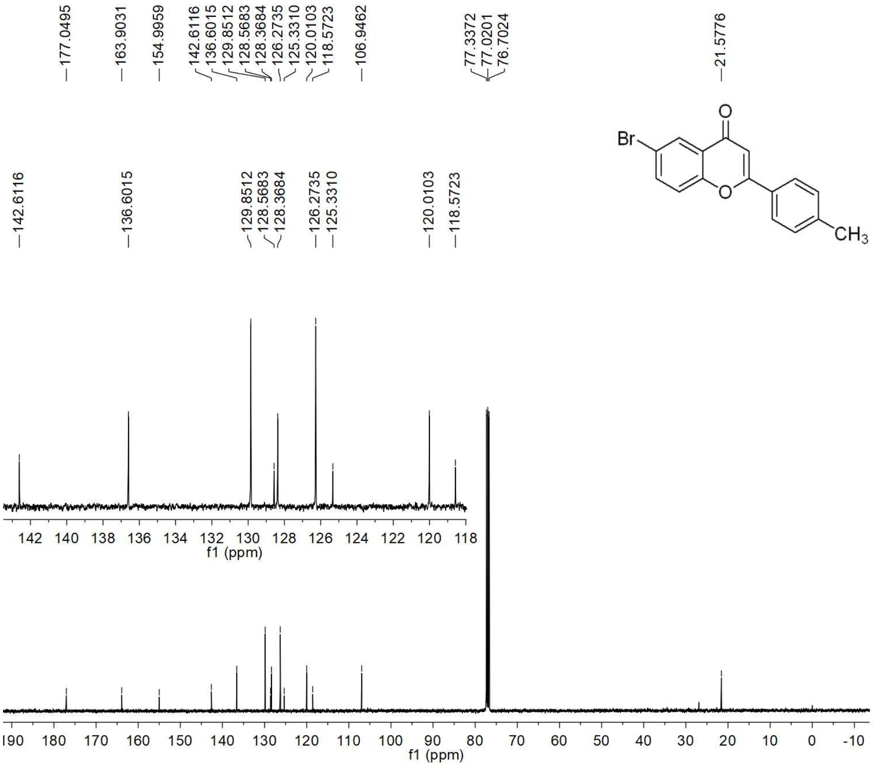

	Parameter	Value
1	Data File Name	E:/ hxw 400/ 2021-2-hxw-C/ 35/ fid
2	Title	2021-2-hxw-C. 35.fid
3	Comment	
4	Origin	Bruker BioSpin GmbH
5	Owner	nmrsu
6	Site	
7	Instrument	Avance
8	Author	
9	Solvent	CDCl3
10	Temperature	298.7
11	Pulse Sequence	zgpg30
12	Experiment	1D
13	Probe	Z116098_0916 (PA BBO 400S1 BBF-H-D-05 Z SP)
14	Number of Scans	500
15	Receiver Gain	101.0
16	Relaxation Delay	2.0000
17	Pulse Width	10.0000
18	Presaturation Frequency	
19	Acquisition Time	1.3763
20	Acquisition Date	2021-10-27T02:55:32
21	Modification Date	2021-10-27T02:54:28
22	Class	
23	Spectrometer Frequency	100.62
24	Spectral Width	23809.5
25	Lowest Frequency	-1844.0
26	Nucleus	13C
27	Acquired Size	32768
28	Spectral Size	65536

6-Bromo-2-(4-methoxyphenyl)-4*H*-chromen-4-one (2ec)

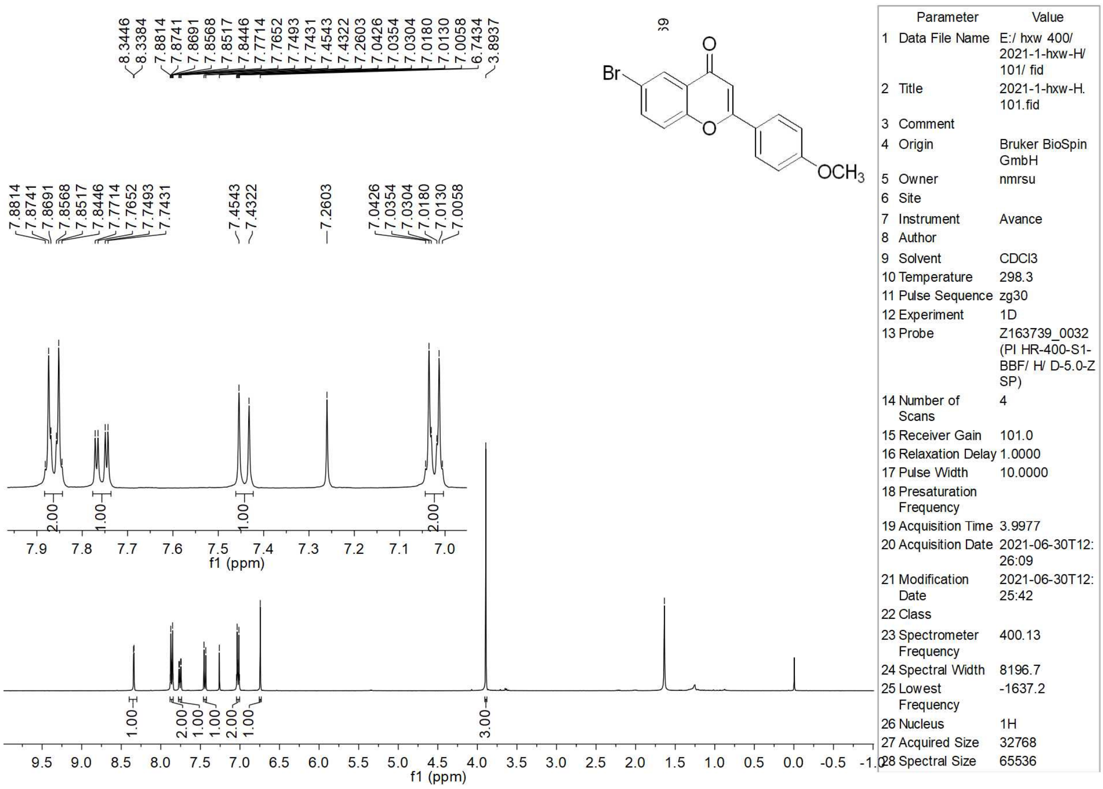

	Parameter	Value
1	Data File Name	E:/ hxw 400/ 2021-1-hxw-H/ 101/ fid
2	Title	2021-1-hxw-H. 101.fid
3	Comment	
4	Origin	Bruker BioSpin GmbH
5	Owner	nmrsu
6	Site	
7	Instrument	Avance
8	Author	
9	Solvent	CDCl3
10	Temperature	298.3
11	Pulse Sequence	zg30
12	Experiment	1D
13	Probe	Z163739_0032 (PI HR-400-S1-BBF/ H/ D-5.0-Z SP)
14	Number of Scans	4
15	Receiver Gain	101.0
16	Relaxation Delay	1.0000
17	Pulse Width	10.0000
18	Presaturation Frequency	
19	Acquisition Time	3.9977
20	Acquisition Date	2021-06-30T12:26:09
21	Modification Date	2021-06-30T12:25:42
22	Class	
23	Spectrometer Frequency	400.13
24	Spectral Width	8196.7
25	Lowest Frequency	-1637.2
26	Nucleus	1H
27	Acquired Size	32768
28	Spectral Size	65536

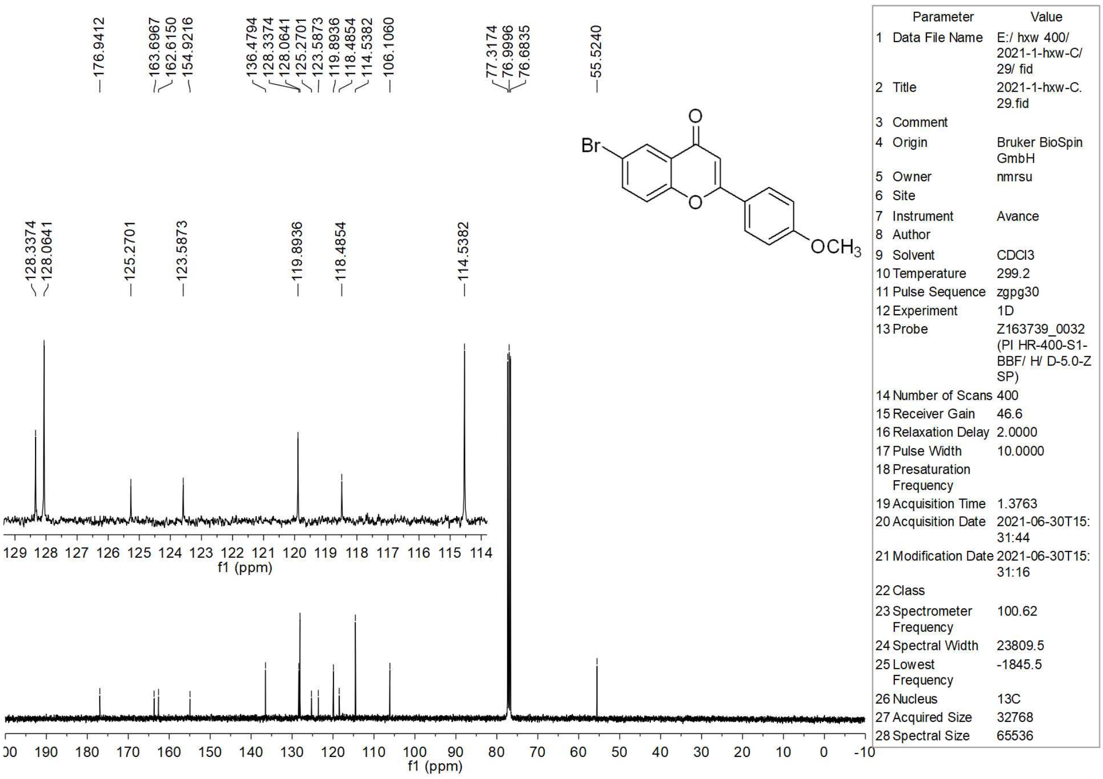

	Parameter	Value
1	Data File Name	E:/ hxw 400/ 2021-1-hxw-C/ 29/ fid
2	Title	2021-1-hxw-C. 29.fid
3	Comment	
4	Origin	Bruker BioSpin GmbH
5	Owner	nmrsu
6	Site	
7	Instrument	Avance
8	Author	
9	Solvent	CDCl3
10	Temperature	299.2
11	Pulse Sequence	zgpg30
12	Experiment	1D
13	Probe	Z163739_0032 (PI HR-400-S1-BBF/ H/ D-5.0-Z SP)
14	Number of Scans	400
15	Receiver Gain	46.6
16	Relaxation Delay	2.0000
17	Pulse Width	10.0000
18	Presaturation Frequency	
19	Acquisition Time	1.3763
20	Acquisition Date	2021-06-30T15:31:44
21	Modification Date	2021-06-30T15:31:16
22	Class	
23	Spectrometer Frequency	100.62
24	Spectral Width	23809.5
25	Lowest Frequency	-1845.5
26	Nucleus	13C
27	Acquired Size	32768
28	Spectral Size	65536

6-Bromo-2-(4-chlorophenyl)-4*H*-chromen-4-one (2ee)

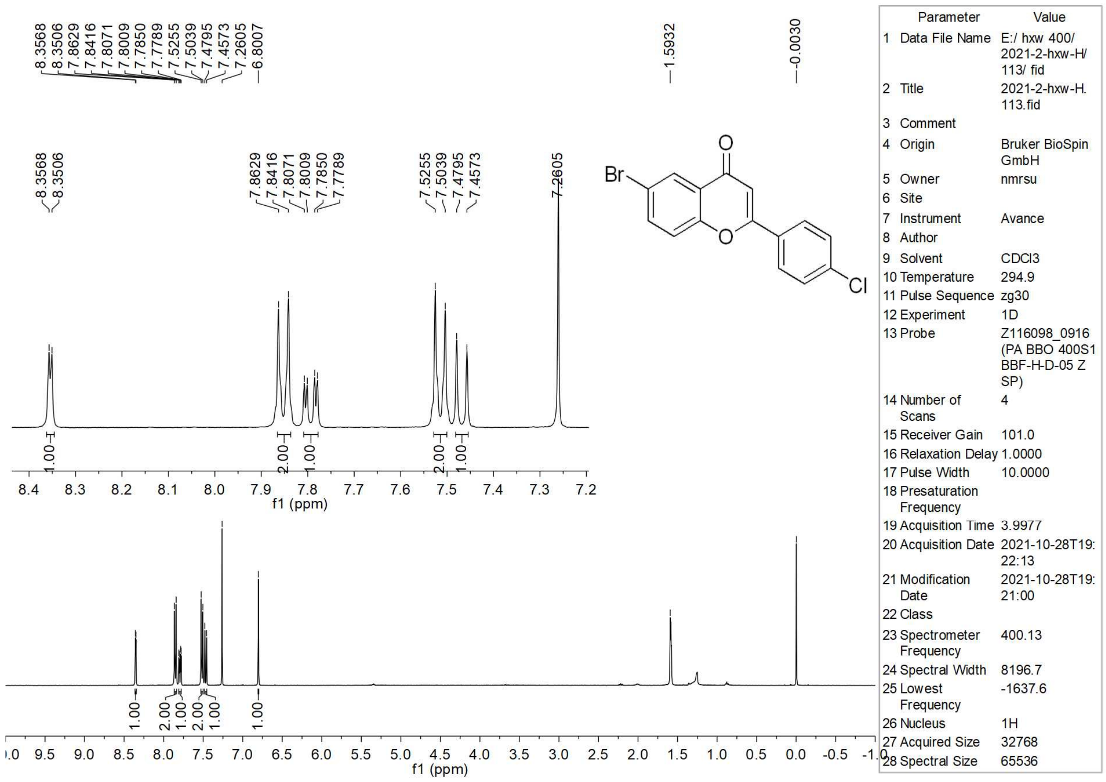

Parameter		Value
1	Data File Name	E:/ hxw 400/ 2021-2-hxw-H/ 113/ fid
2	Title	2021-2-hxw-H. 113.fid
3	Comment	
4	Origin	Bruker BioSpin GmbH
5	Owner	nmrsu
6	Site	
7	Instrument	Avance
8	Author	
9	Solvent	CDCl3
10	Temperature	294.9
11	Pulse Sequence	zg30
12	Experiment	1D
13	Probe	Z116098_0916 (PA BBO 400S1 BBF-H-D-05 Z SP)
14	Number of Scans	4
15	Receiver Gain	101.0
16	Relaxation Delay	1.0000
17	Pulse Width	10.0000
18	Presaturation Frequency	
19	Acquisition Time	3.9977
20	Acquisition Date	2021-10-28T19:22:13
21	Modification Date	2021-10-28T19:21:00
22	Class	
23	Spectrometer Frequency	400.13
24	Spectral Width	8196.7
25	Lowest Frequency	-1637.6
26	Nucleus	1H
27	Acquired Size	32768
28	Spectral Size	65536

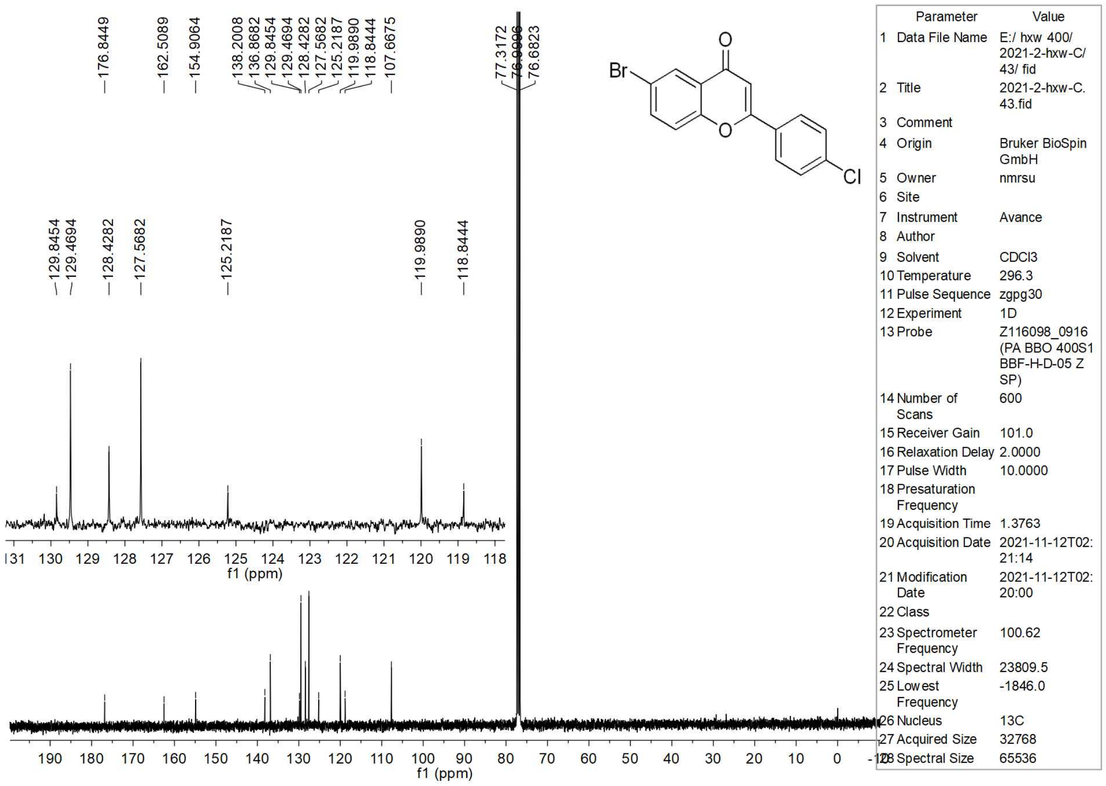

Parameter		Value
1	Data File Name	E:/ hxw 400/ 2021-2-hxw-C/ 43/ fid
2	Title	2021-2-hxw-C. 43.fid
3	Comment	
4	Origin	Bruker BioSpin GmbH
5	Owner	nmrsu
6	Site	
7	Instrument	Avance
8	Author	
9	Solvent	CDCl3
10	Temperature	296.3
11	Pulse Sequence	zgpg30
12	Experiment	1D
13	Probe	Z116098_0916 (PA BBO 400S1 BBF-H-D-05 Z SP)
14	Number of Scans	600
15	Receiver Gain	101.0
16	Relaxation Delay	2.0000
17	Pulse Width	10.0000
18	Presaturation Frequency	
19	Acquisition Time	1.3763
20	Acquisition Date	2021-11-12T02:21:14
21	Modification Date	2021-11-12T02:20:00
22	Class	
23	Spectrometer Frequency	100.62
24	Spectral Width	23809.5
25	Lowest Frequency	-1846.0
26	Nucleus	13C
27	Acquired Size	32768
28	Spectral Size	65536

6-Bromo-2-(4-bromophenyl)-4*H*-chromen-4-one (2ef)

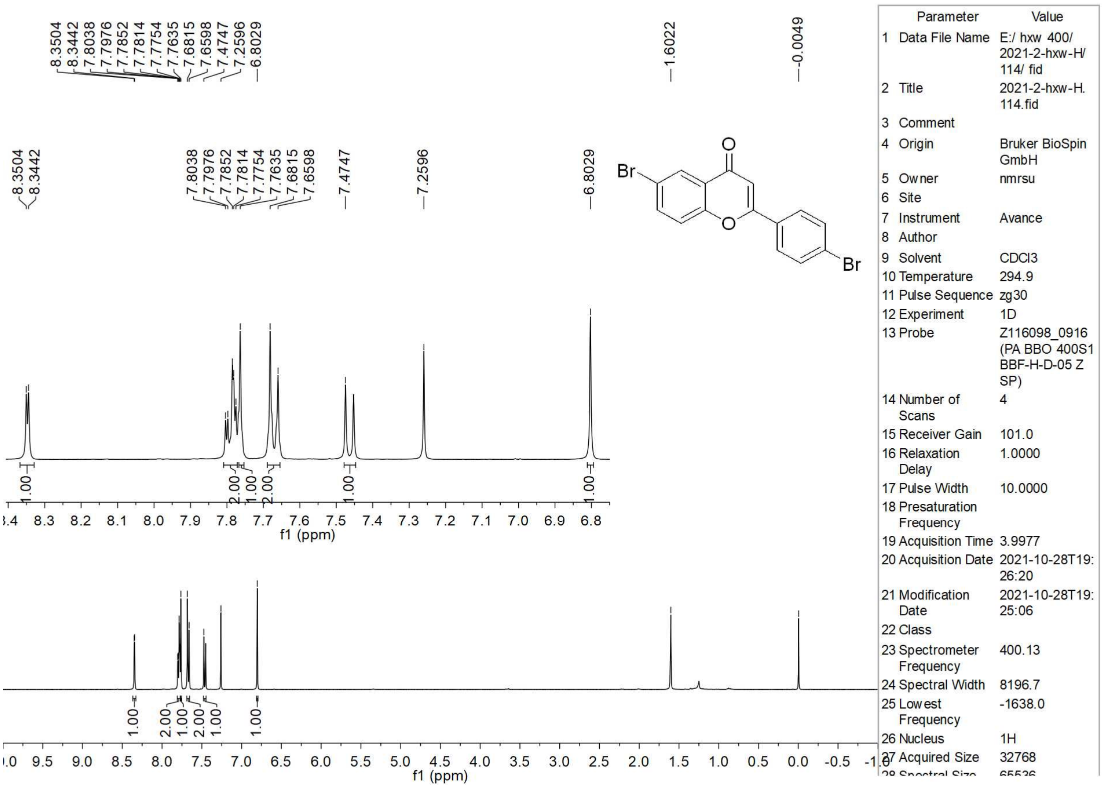

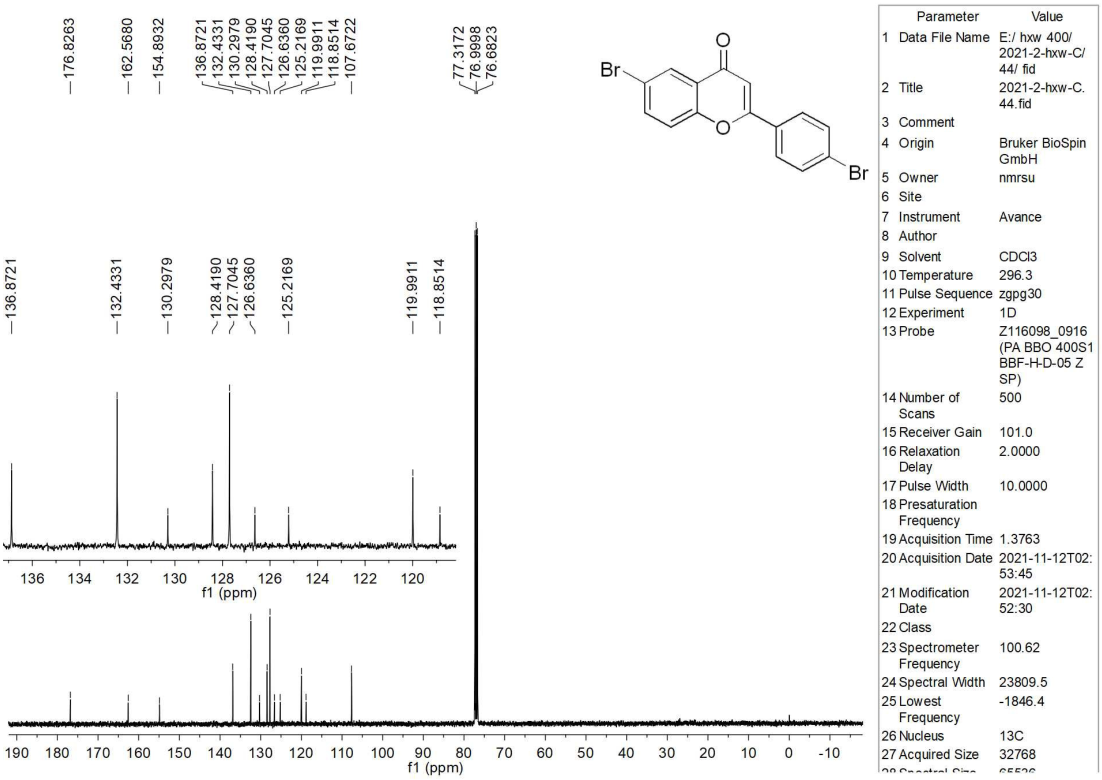

6-Bromo-2-(4-ethylphenyl)-4*H*-chromen-4-one (2en)

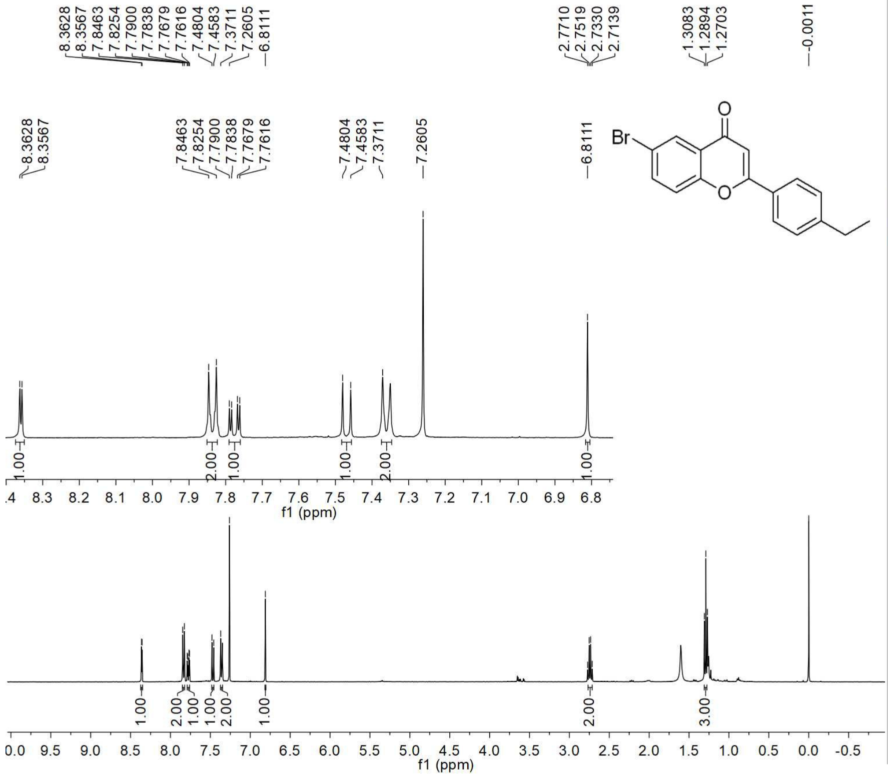

Parameter	Value
1 Data File Name	E:/ hxw 400/ 2021-2-hxw-H/ 59/ fid
2 Title	2021-2-hxw-H. 59.fid
3 Comment	
4 Origin	Bruker BioSpin GmbH
5 Owner	nmrsu
6 Site	
7 Instrument	Avance
8 Author	
9 Solvent	CDCl3
10 Temperature	297.7
11 Pulse Sequence	zg30
12 Experiment	1D
13 Probe	Z116098_0916 (PA BBO 400S1 BBF-H-D-05 Z SP)
14 Number of Scans	4
15 Receiver Gain	101.0
16 Relaxation Delay	1.0000
17 Pulse Width	10.0000
18 Presaturation Frequency	
19 Acquisition Time	3.9977
20 Acquisition Date	2021-09-23T18:38:54
21 Modification Date	2021-09-23T18:38:32
22 Class	
23 Spectrometer Frequency	400.13
24 Spectral Width	8196.7
25 Lowest Frequency	-1637.6
26 Nucleus	1H
27 Acquired Size	32768
28 Spectral Size	65536

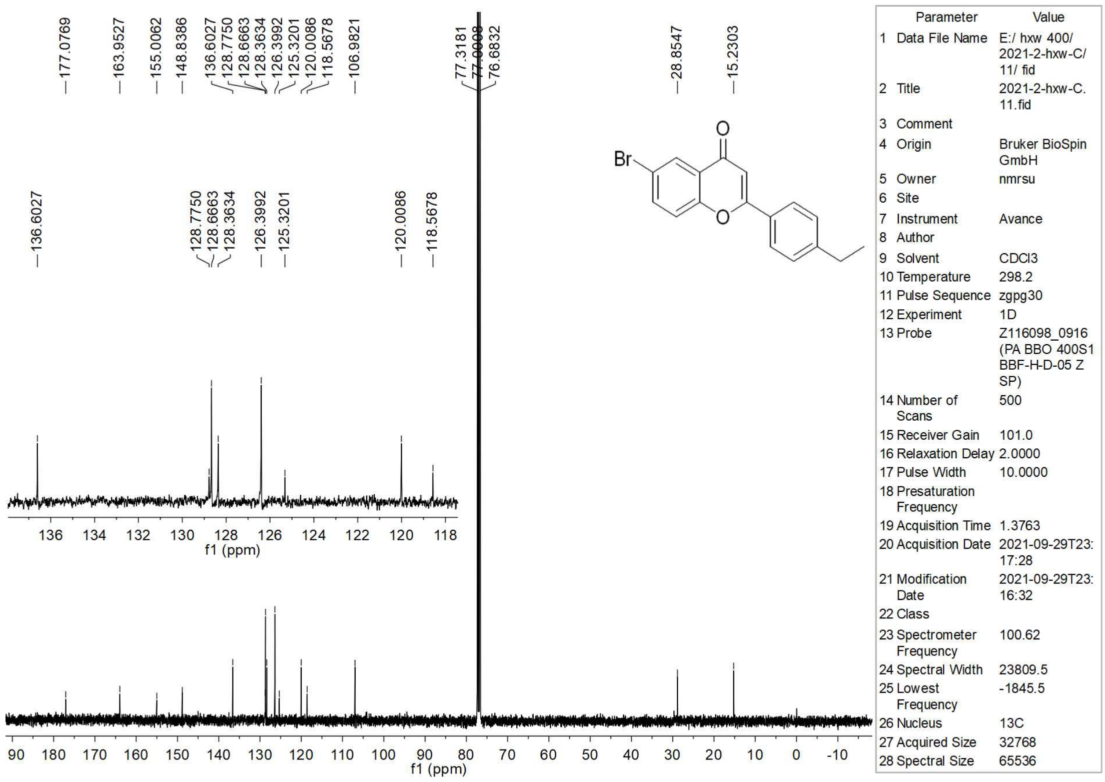

Parameter	Value
1 Data File Name	E:/ hxw 400/ 2021-2-hxw-C/ 11/ fid
2 Title	2021-2-hxw-C. 11.fid
3 Comment	
4 Origin	Bruker BioSpin GmbH
5 Owner	nmrsu
6 Site	
7 Instrument	Avance
8 Author	
9 Solvent	CDCl3
10 Temperature	298.2
11 Pulse Sequence	zgpg30
12 Experiment	1D
13 Probe	Z116098_0916 (PA BBO 400S1 BBF-H-D-05 Z SP)
14 Number of Scans	500
15 Receiver Gain	101.0
16 Relaxation Delay	2.0000
17 Pulse Width	10.0000
18 Presaturation Frequency	
19 Acquisition Time	1.3763
20 Acquisition Date	2021-09-29T23:17:28
21 Modification Date	2021-09-29T23:16:32
22 Class	
23 Spectrometer Frequency	100.62
24 Spectral Width	23809.5
25 Lowest Frequency	-1845.5
26 Nucleus	13C
27 Acquired Size	32768
28 Spectral Size	65536

Butyl (*E*)-3-(4-oxo-2-phenyl-4*H*-chromen-5-yl)acrylate (4aa)

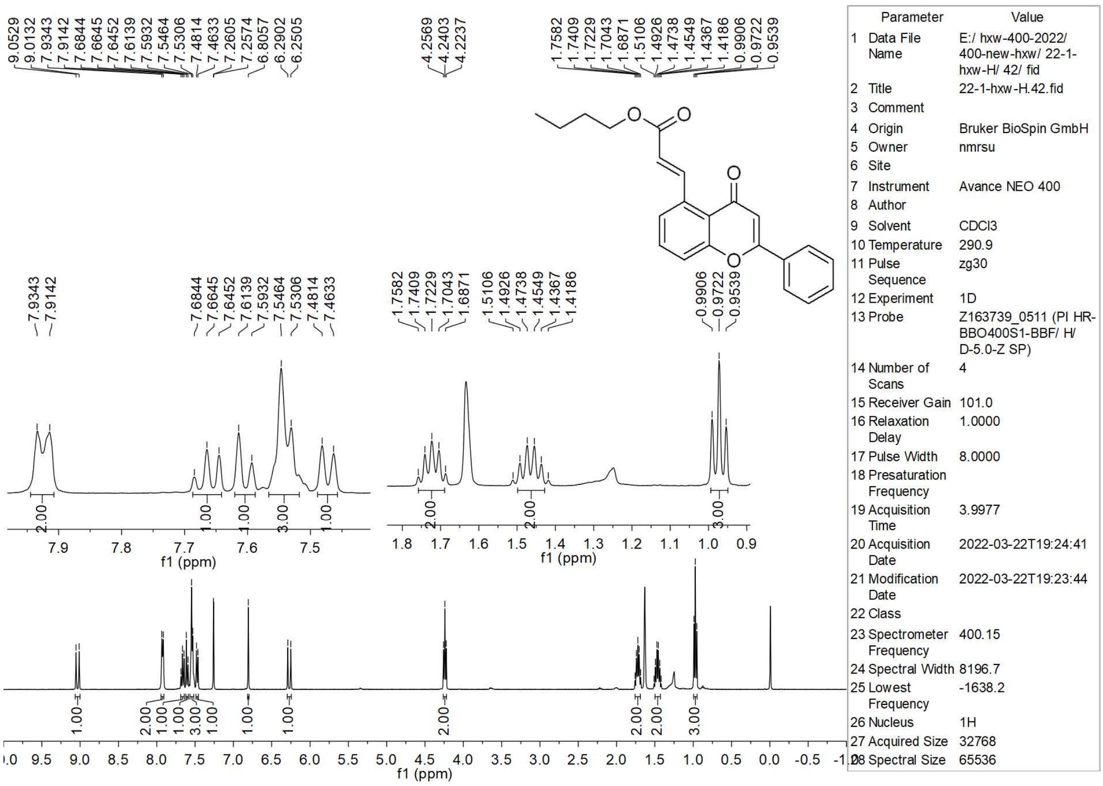

Parameter	Value
1 Data File Name	E:/ hxw-400-2022/ 400-new-hxw/ 22-1-hxw-H/ 42/ fid
2 Title	22-1-hxw-H.42.fid
3 Comment	
4 Origin	Bruker BioSpin GmbH
5 Owner	nmrsu
6 Site	
7 Instrument	Avance NEO 400
8 Author	
9 Solvent	CDCl3
10 Temperature	290.9
11 Pulse Sequence	zg30
12 Experiment	1D
13 Probe	Z163739_0511 (PI HR-BBO400S1-BBF/ H/ D-5.0-Z SP)
14 Number of Scans	4
15 Receiver Gain	101.0
16 Relaxation Delay	1.0000
17 Pulse Width	8.0000
18 Presaturation Frequency	
19 Acquisition Time	3.9977
20 Acquisition Date	2022-03-22T19:24:41
21 Modification Date	2022-03-22T19:23:44
22 Class	
23 Spectrometer Frequency	400.15
24 Spectral Width	8196.7
25 Lowest Frequency	-1638.2
26 Nucleus	1H
27 Acquired Size	32768
28 Spectral Size	65536

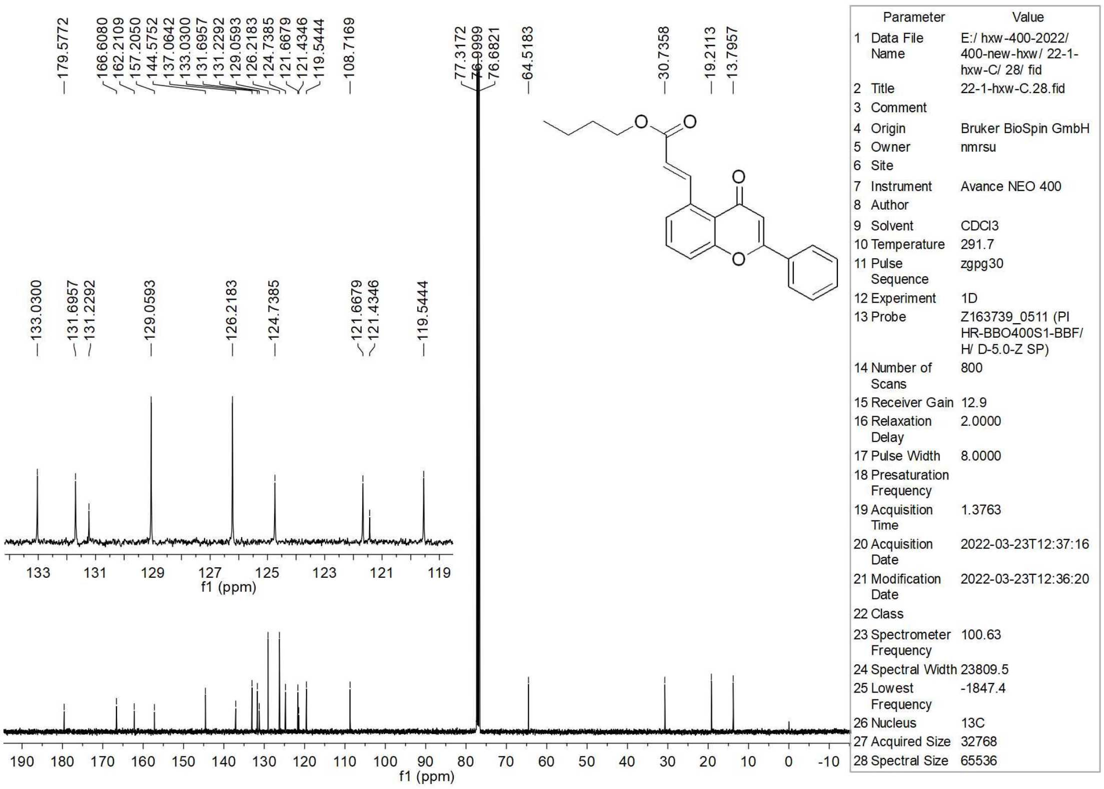

Parameter	Value
1 Data File Name	E:/ hxw-400-2022/ 400-new-hxw/ 22-1-hxw-C/ 28/ fid
2 Title	22-1-hxw-C.28.fid
3 Comment	
4 Origin	Bruker BioSpin GmbH
5 Owner	nmrsu
6 Site	
7 Instrument	Avance NEO 400
8 Author	
9 Solvent	CDCl3
10 Temperature	291.7
11 Pulse Sequence	zgpg30
12 Experiment	1D
13 Probe	Z163739_0511 (PI HR-BBO400S1-BBF/ H/ D-5.0-Z SP)
14 Number of Scans	800
15 Receiver Gain	12.9
16 Relaxation Delay	2.0000
17 Pulse Width	8.0000
18 Presaturation Frequency	
19 Acquisition Time	1.3763
20 Acquisition Date	2022-03-23T12:37:16
21 Modification Date	2022-03-23T12:36:20
22 Class	
23 Spectrometer Frequency	100.63
24 Spectral Width	23809.5
25 Lowest Frequency	-1847.4
26 Nucleus	13C
27 Acquired Size	32768
28 Spectral Size	65536

Butyl (*E*)-3-(4-oxo-2-(*p*-tolyl)-4*H*-chromen-5-yl)acrylate (4ab)

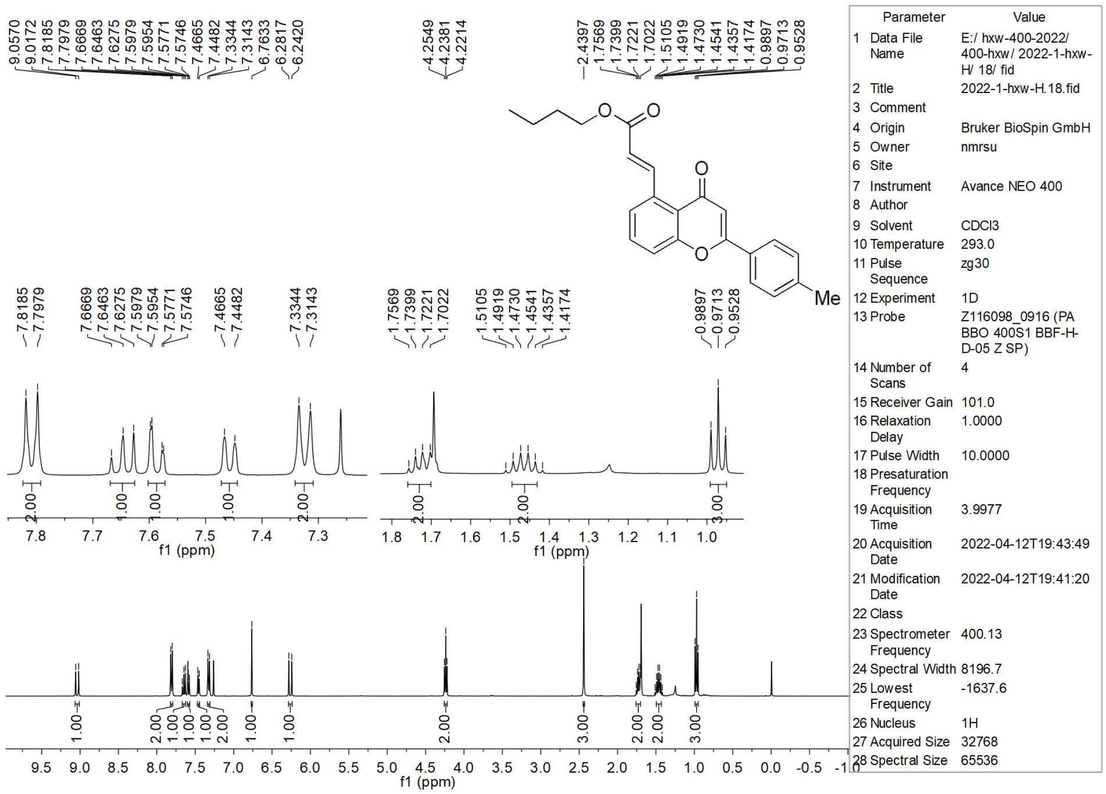

Parameter	Value
1 Data File Name	E:/ hxw-400-2022/ 400-hxw/ 2022-1-hxw-H/ 18/ fid
2 Title	2022-1-hxw-H.18.fid
3 Comment	
4 Origin	Bruker BioSpin GmbH
5 Owner	nmrsu
6 Site	
7 Instrument	Avance NEO 400
8 Author	
9 Solvent	CDCl3
10 Temperature	293.0
11 Pulse Sequence	zg30
12 Experiment	1D
13 Probe	Z116098_0916 (PA BBO 400S1 BBF-H-D-05 Z SP)
14 Number of Scans	4
15 Receiver Gain	101.0
16 Relaxation Delay	1.0000
17 Pulse Width	10.0000
18 Presaturation Frequency	
19 Acquisition Time	3.9977
20 Acquisition Date	2022-04-12T19:43:49
21 Modification Date	2022-04-12T19:41:20
22 Class	
23 Spectrometer Frequency	400.13
24 Spectral Width	8196.7
25 Lowest Frequency	-1637.6
26 Nucleus	1H
27 Acquired Size	32768
28 Spectral Size	65536

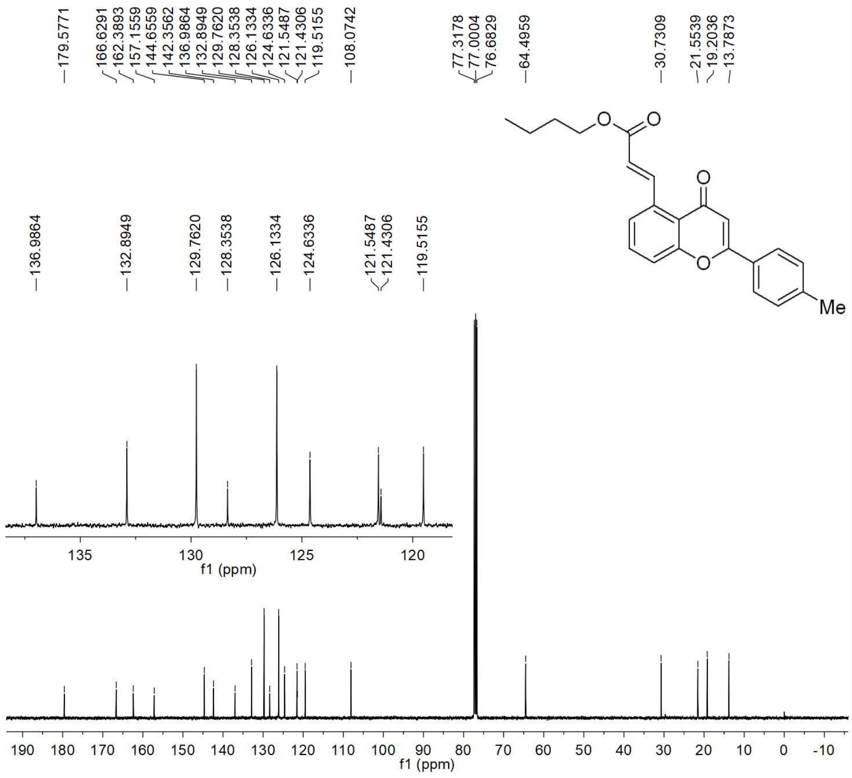

Parameter	Value
1 Data File Name	E:/ hxw-400-2022/ 400-new-hxw/ 22-1-hxw-C/ 41/ fid
2 Title	22-1-hxw-C.41.fid
3 Comment	
4 Origin	Bruker BioSpin GmbH
5 Owner	nmrsu
6 Site	
7 Instrument	Avance NEO 400
8 Author	
9 Solvent	CDCl3
10 Temperature	292.1
11 Pulse Sequence	zgpg30
12 Experiment	1D
13 Probe	Z163739_0511 (PI HR-BBO400S1-BBF/ H/ D-5.0-Z SP)
14 Number of Scans	800
15 Receiver Gain	13.2
16 Relaxation Delay	2.0000
17 Pulse Width	8.0000
18 Presaturation Frequency	
19 Acquisition Time	1.3763
20 Acquisition Date	2022-04-15T19:16:51
21 Modification Date	2022-04-15T19:17:00
22 Class	
23 Spectrometer Frequency	100.63
24 Spectral Width	23809.5
25 Lowest Frequency	-1848.1
26 Nucleus	13C
27 Acquired Size	32768
28 Spectral Size	65536

Butyl (*E*)-3-(2-(4-bromophenyl)-4-oxo-4*H*-chromen-5-yl)acrylate (4af)

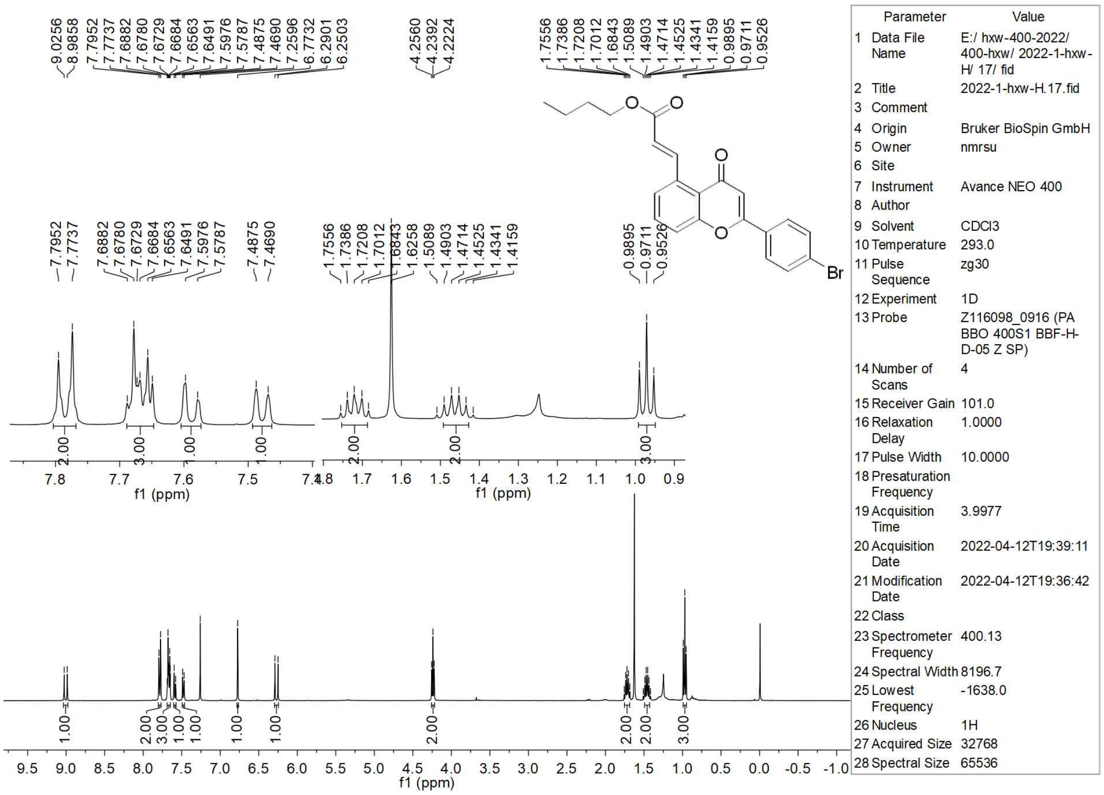

Parameter	Value
1 Data File Name	E:/ hxw-400-2022/ 400-hxw/ 2022-1-hxw-H/ 17/ fid
2 Title	2022-1-hxw-H.17.fid
3 Comment	
4 Origin	Bruker BioSpin GmbH
5 Owner	nmrsu
6 Site	
7 Instrument	Avance NEO 400
8 Author	
9 Solvent	CDCl3
10 Temperature	293.0
11 Pulse Sequence	zg30
12 Experiment	1D
13 Probe	Z116098_0916 (PA BBO 400S1 BBF-H-D-05 Z SP)
14 Number of Scans	4
15 Receiver Gain	101.0
16 Relaxation Delay	1.0000
17 Pulse Width	10.0000
18 Presaturation Frequency	
19 Acquisition Time	3.9977
20 Acquisition Date	2022-04-12T19:39:11
21 Modification Date	2022-04-12T19:36:42
22 Class	
23 Spectrometer Frequency	400.13
24 Spectral Width	8196.7
25 Lowest Frequency	-1638.0
26 Nucleus	1H
27 Acquired Size	32768
28 Spectral Size	65536

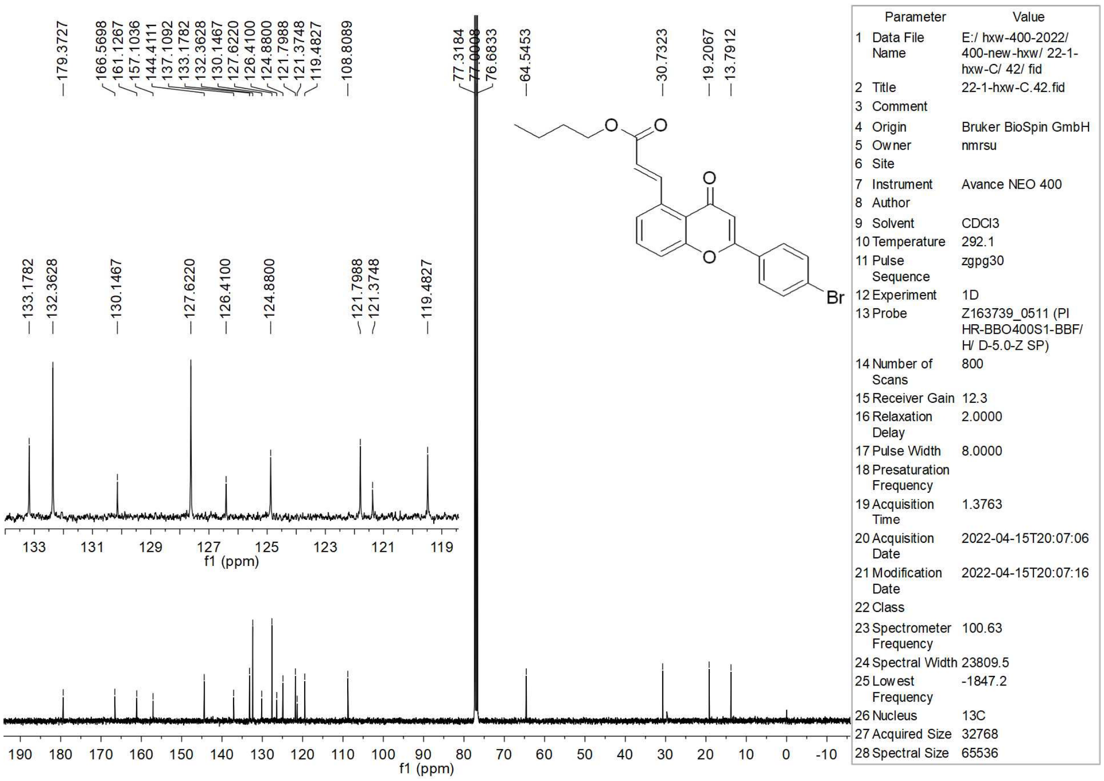

Parameter	Value
1 Data File Name	E:/ hxw-400-2022/ 400-new-hxw/ 22-1-hxw-C/ 42/ fid
2 Title	22-1-hxw-C.42.fid
3 Comment	
4 Origin	Bruker BioSpin GmbH
5 Owner	nmrsu
6 Site	
7 Instrument	Avance NEO 400
8 Author	
9 Solvent	CDCl3
10 Temperature	292.1
11 Pulse Sequence	zgpg30
12 Experiment	1D
13 Probe	Z163739_0511 (PI HR-BBO400S1-BBF/H/ D-5.0-Z SP)
14 Number of Scans	800
15 Receiver Gain	12.3
16 Relaxation Delay	2.0000
17 Pulse Width	8.0000
18 Presaturation Frequency	
19 Acquisition Time	1.3763
20 Acquisition Date	2022-04-15T20:07:06
21 Modification Date	2022-04-15T20:07:16
22 Class	
23 Spectrometer Frequency	100.63
24 Spectral Width	23809.5
25 Lowest Frequency	-1847.2
26 Nucleus	13C
27 Acquired Size	32768
28 Spectral Size	65536

9. GC-MS spectra for mechanistic investigations

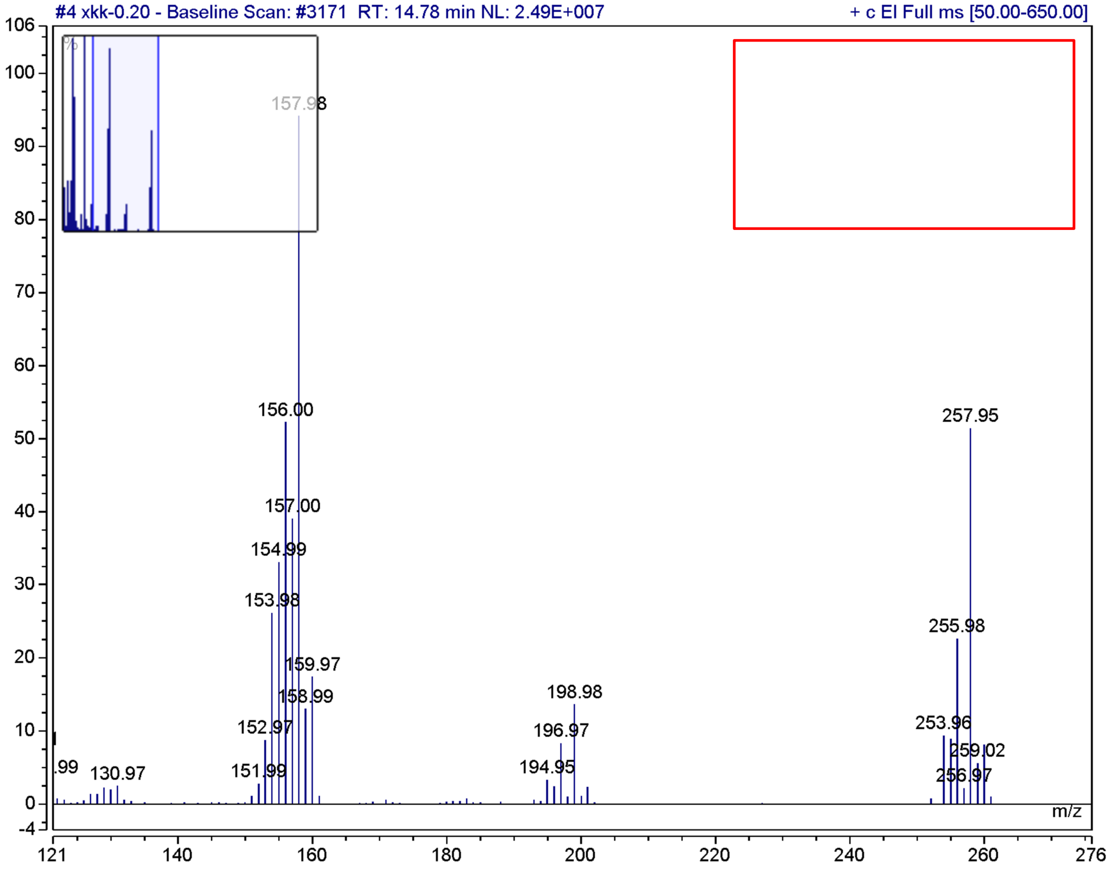

Figure S2. GC-MS spectra of possible intermediate **B**

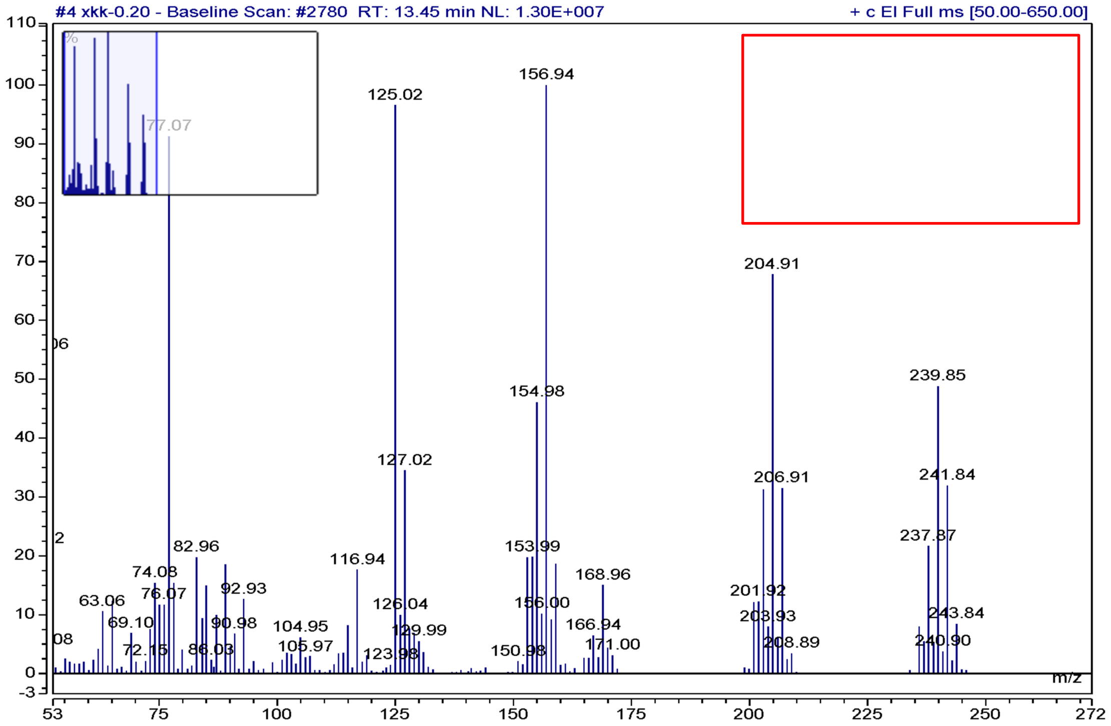

Figure S3. GC-MS spectra of 1-(phenylselanyl)piperidine

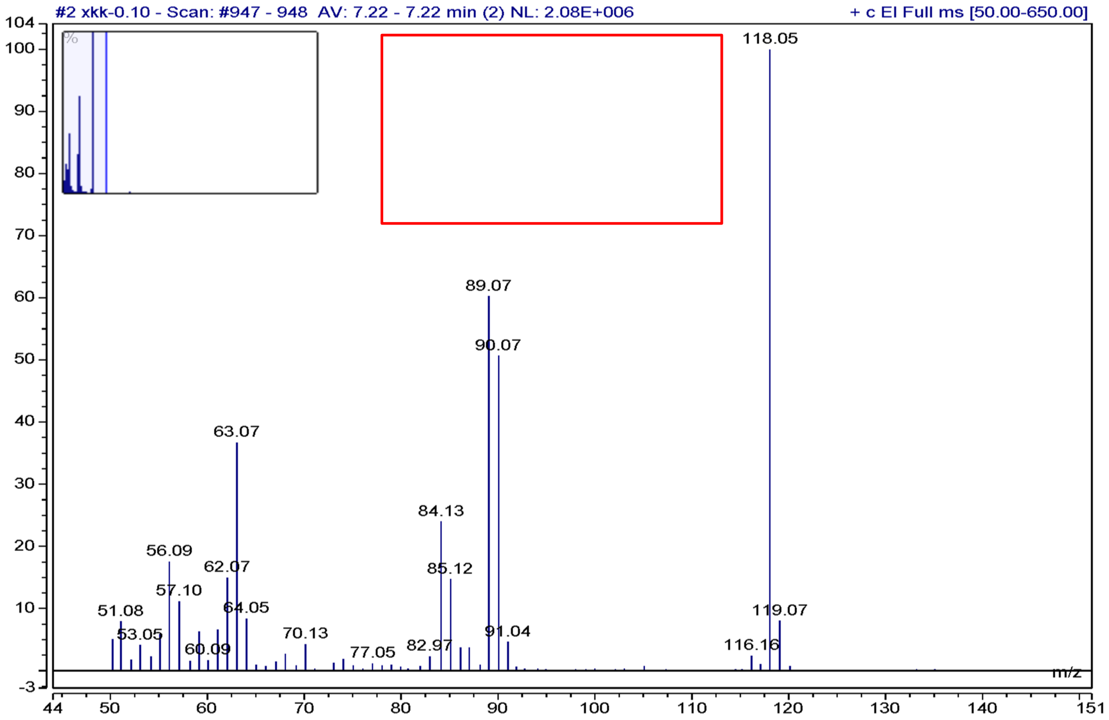

Figure S4. GC-MS spectra of methyl 2-hydroxy-2-methylpropanoate

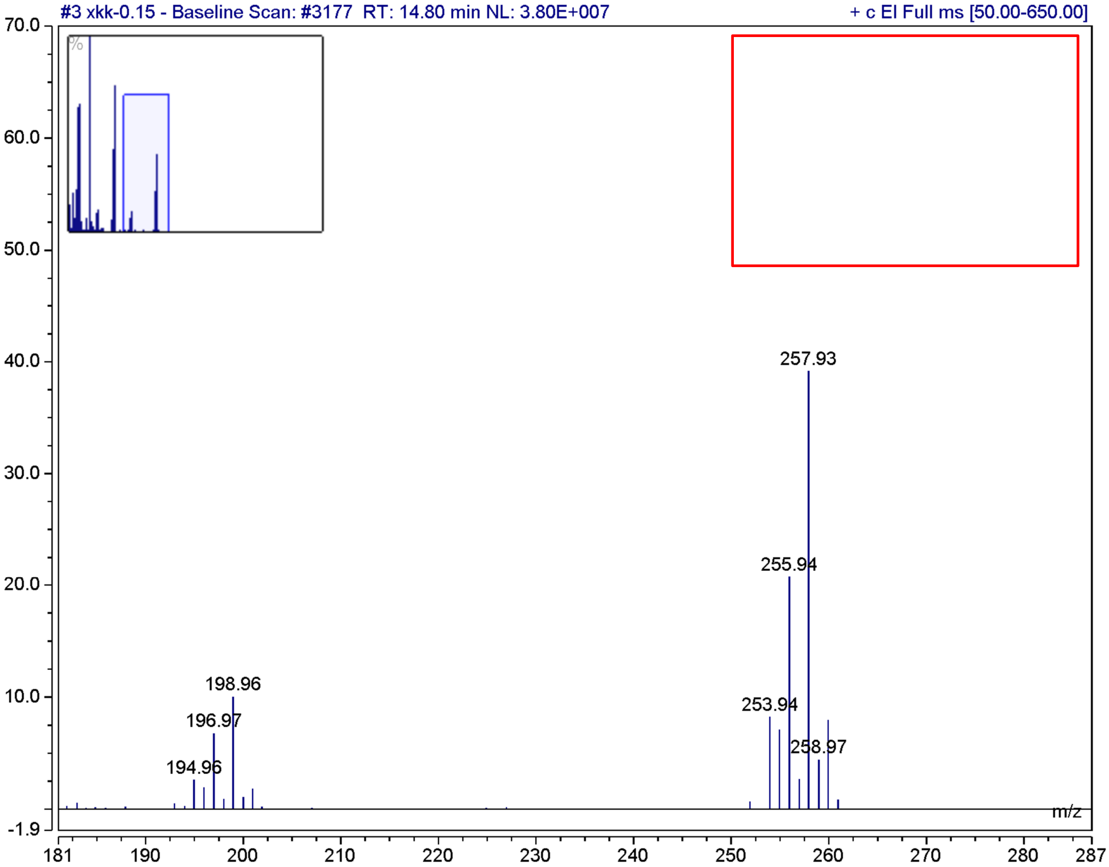

Figure S5. GC-MS spectra of the control experiment in the presence of TEMPO.

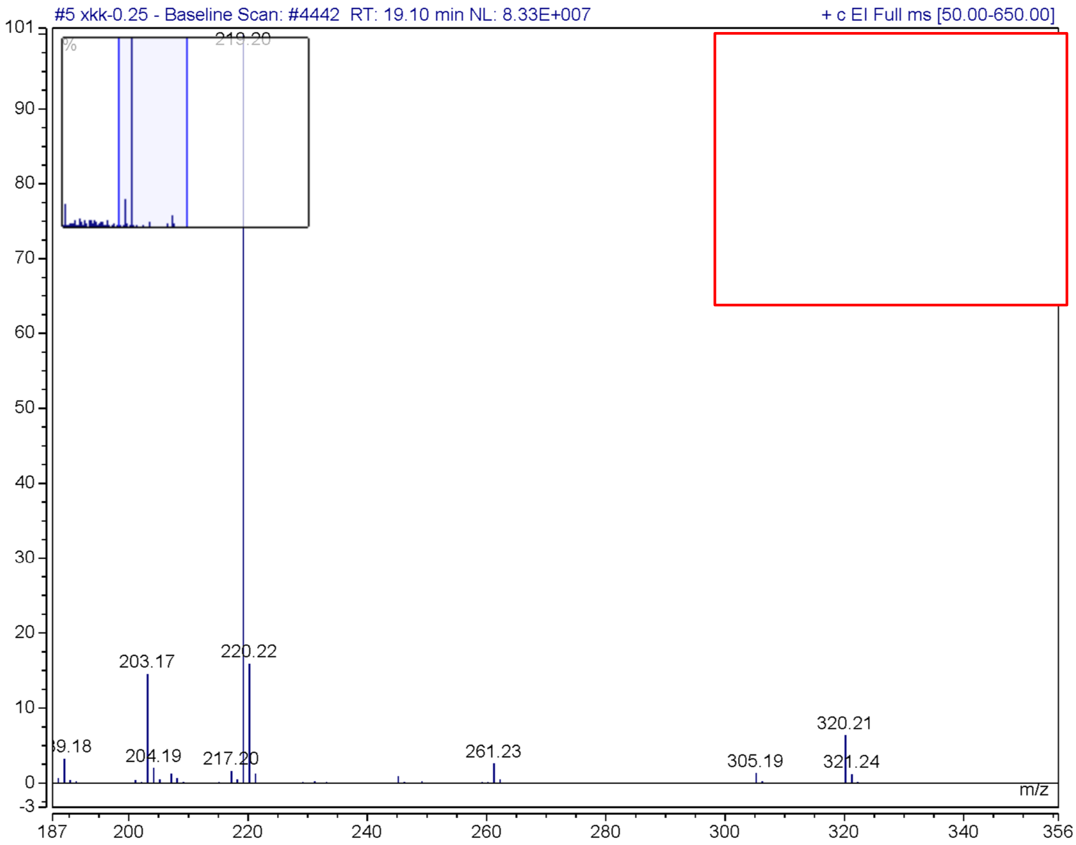

Figure S6. GC-MS spectra of the control experiment in the presence of BHT.
